# NEK6 dampens FOXO3 nuclear translocation to stabilize C-MYC and promotes subsequent de novo purine synthesis to support ovarian cancer chemoresistance

**DOI:** 10.1038/s41419-024-07045-2

**Published:** 2024-09-10

**Authors:** Jingchun Liu, Haoyu Wang, Huanzhi Wan, Jiang Yang, Likun Gao, Zhi Wang, Xiaoyi Zhang, Wuyue Han, Jiaxin Peng, Lian Yang, Li Hong

**Affiliations:** 1 Department of Gynecology and Obstetrics, Renmin Hospital of Wuhan University, Wuhan, Hubei China; 2 The First Clinical School of Wuhan University, Wuhan University, Wuhan, Hubei China; 3 Department of Obstetrics and Gynecology, Xiangyang Central Hospital, Xiangyang, Hubei China; 4 Department of Pathology, Shenzhen People’s Hospital, The Second Clinical Medical College of Jinan University, Shenzhen, 518020 China

**Keywords:** Ovarian cancer, Cancer metabolism, Protein translocation

## Abstract

De novo purine synthesis metabolism plays a crucial role in tumor cell survival and malignant progression. However, the specific impact of this metabolic pathway on chemoresistance in ovarian cancer remains unclear. This study aims to elucidate the influence of de novo purine synthesis on chemoresistance in ovarian cancer and its underlying regulatory mechanisms. We analyzed metabolic differences between chemosensitive and chemoresistant ovarian cancer tissues using mass spectrometry-based metabolomics. Cell growth, metabolism, chemoresistance, and DNA damage repair characteristics were assessed in vitro using cell line models. Tumor growth and chemoresistance were assessed in vivo using ovarian cancer xenograft tumors. Intervention of purines and NEK6-mediated purine metabolism on chemoresistance was investigated at multiple levels. Chemoresistant ovarian cancers exhibited higher purine abundance and NEK6 expression. Inhibiting NEK6 led to decreased de novo purine synthesis, resulting in diminished chemoresistance in ovarian cancer cells. Mechanistically, NEK6 directly interacted with FOXO3, contributing to the phosphorylation of FOXO3 at S7 through its kinase activity, thereby inhibiting its nuclear translocation. Nuclear FOXO3 promoted FBXW7 transcription, leading to c-MYC ubiquitination and suppression of de novo purine synthesis. Paeonol, by inhibiting NEK6, suppressed de novo purine synthesis and enhanced chemosensitivity. The NEK6-mediated reprogramming of de novo purine synthesis emerges as a critical pathway influencing chemoresistance in ovarian cancer. Paeonol exhibits the potential to interfere with NEK6, thereby inhibiting chemoresistance.

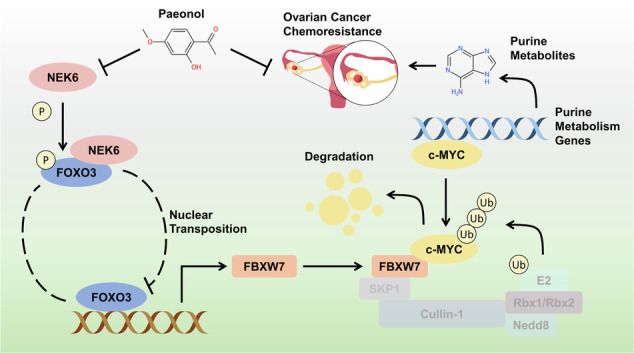

## Introduction

Purine metabolism stands as a pivotal branch within nucleotide metabolism, playing a crucial role in maintaining the homeostasis of DNA and RNA, essential for normal cell growth. Furthermore, purines contribute to immunomodulation and energy metabolism, and potentially serve as vital cofactors in biochemical reactions [1]. Disruptions in purine metabolism can trigger uncontrolled cell growth and disrupt normal cellular processes, impacting proliferation, migration, and cell death. De novo purine synthesis is frequently heightened in tumor cells, fostering accelerated proliferation and the acquisition of malignant properties.

Ovarian cancer stands as an extremely perilous malignant gynecologic neoplasm, impacting women globally and imposing a substantial social burden. Statistically, ovarian cancer ranks as the fifth leading cause of cancer-related deaths among women worldwide, with a relative 5-year survival rate of 49% [2, 3]. The profoundly malignant and chemoresistant nature of ovarian cancer presents a formidable global challenge. Previous studies have indicated that abnormalities in several metabolic pathways, including glucose metabolism, lipid metabolism, iron metabolism, and amino acid metabolism, are associated with chemoresistance in ovarian cancer [4–8]. However, the correlation between de novo purine synthesis and chemoresistance in ovarian cancer remains to be elucidated.

NEK6, a member of the NIMA-related kinase family, is widely distributed in human tissues and intricately linked to cell mitosis. Concurrently, NEK6 possesses serine/threonine kinase activity with the potential to influence protein synthesis [9], regulate cell viability [10], and inhibit cellular autophagy [11]. Overexpression of NEK6 has been observed in several solid human tumors [12–14], promoting tumor proliferation, invasion, and metastasis [15–17]. Recent studies have revealed a potential role for NEK6 in conferring resistance to tumor therapy. However, its function in ovarian cancer has not yet been fully characterized [18].

In this study, we demonstrated that purine supplementation contributed to the repair of DNA damage after chemotherapy. The overactivation of de novo purine synthesis is evident in chemoresistant ovarian cancer, with NEK6 identified as a key molecule. We further revealed that NEK6 directly interacts with FOXO3, inducing phosphorylation of FOXO3 at S7, inhibiting its nuclear translocation, slowing FBXW7 transcription to prevent C-MYC ubiquitination degradation, and ensuring robust de novo purine synthesis. Additionally, we identified paeonol (PAE) as a promising agent for inhibiting NEK6-mediated chemoresistance in ovarian cancer. Thus, our study provides a new perspective for combating chemoresistance in ovarian cancer.

## Materials and methods

### Cell culture

Human ovarian cancer cell lines SKOV3 and SKOV3/DDP were obtained from the China Center for Type Culture Collection (Wuhan, China). OVCAR8 cells were generously provided by Huazhong University of Science and Technology. The NCI/ADR-RES cell line was kindly donated by iCell Bioscience (China). HEK293T cells were maintained at Renmin Hospital of Wuhan University. Notably, NCI/ADR-RES is a stable chemoresistant cell line derived from OVCAR8, and SKOV3/DDP is a stable chemoresistant cell line developed from SKOV3. All cell lines used in this study underwent characterization through short tandem repeat analysis and passed mycoplasma contamination detection. Unless specified otherwise, cells were cultured in RPMI-1640 (Gibco) supplemented with 10% fetal bovine serum (Yeason) at 37 °C and 5% CO2. For purine addition, 60 µM adenosine (MCE, HY-B0228), guanosine (MCE, HY-N0097), and inosine (MCE, HY-N0092) were co-cultured with cells for 24 h to enrich the intracellular purine pool. Purine depletion was induced in the presence of 10% dialyzed FBS and 50 nM Methotrexate for seven days to minimize purine nucleosides and other intracellular purines.

### Lentivirus, plasmid and reagents

The lentivirus utilized for NEK6 knockdown was sourced from GeneChem (China). In brief, cells were infected at 10 MOI and subjected to screening with 4 µg/ml puromycin. Stable polyclonal cell lines, shCtrl and shNEK6#1-3, were established after two weeks, and subsequent maintenance culture employed 1 µg/ml puromycin. The targeting sequences were as follows: shCtrl, TTCTCCGAACGTGTCACGT; shNEK6#1, ccCGGAGAGGACAGTATGGAA; shNEK6#2, cgAAGACAACGAGCTGAACAT; shNEK6#3, gaACCACCCAAATATCATCAA.

Plasmids Flag, Flag-NEK6-WT, Flag-NEK6-K74/75 M, His, His-FOXO3-WT, His-FOXO3-S7A, and C-MYC used for overexpression or control were present in pCMV vectors, procured from Miaoling Biology (China) and verified by sequencing. pDR-GFP (Addgene, # 26475) and pCBASceI (Addgene, # 26477) were gifts from Maria Jasin. PFAS-mCherry, utilized to characterize purinosomes, was acquired from Addgene (#166024). A stable mixed system of serum-free medium, DNA transfection reagent (Neofect, TF201201), and plasmid was added to the cells according to the manufacturer’s instructions, co-cultivated for 24 hours, and then replaced with fresh medium. Other interventions or assays can be performed after 48 h.

The GST, GST-NEK6-WT, and GST-NEK6-K74/75M plasmids used for the production of purified proteins were loaded into pTac, and, His-FOXO3-WT and His-FOXO3-S7A was loaded in pT7, sourced from Miaoling Biology (China) and verified by sequencing.

Doxorubicin hydrochloride (DOX, Sigma, D1515) and PAE (Sigma, H35803) used in the study were obtained from Sigma. 10058-F4 (MCE, HY-12702), cycloheximide (CHX, MCE, HY- 12320), MG132 (MCE, HY-13259), and ZINC05007751(MCE, HY-122639), IZTZ-1 (MCE, HY-147973) were procured from MCE.

### Sample collection from ovarian cancer patients

Tumor samples and paired paraneoplastic samples were collected from ovarian cancer patients who underwent surgery at Wuhan University People’s Hospital between 2020 and 2023. Exclusion criteria included (1) patients who already suffered from hypertension, diabetes mellitus, or other metabolic diseases (2) patients who had undergone neoadjuvant chemotherapy prior to surgery. The final selection comprised six pairs of samples from ovarian cancer patients sensitive to chemotherapy and six pairs from ovarian cancer patients resistant to chemotherapy. All patients provided informed consent. Tumor tissues were histopathologically confirmed by a pathologist. Chemosensitivity was defined as recurrence of ovarian cancer beyond 6 months or absence of recurrence, while chemoresistance was defined as tumor recurrence within 6 months of initial chemotherapy or continued disease progression during postoperative chemotherapy. This study was approved by the Ethics Committee of the Renmin Hospital of Wuhan University in accordance with the principles set forth in the Helsinki Declaration (WDRY2023-K137).

### Experimental mice, xenograft tumor growth and therapy

Female BALB/c nude mice (4 weeks old) were housed in a specific-pathogen-free environment at the animal facility of the Renmin Hospital of Wuhan University. Mice without intervention were randomly grouped according to the random number method, with five mice assigned to each group. No statistical method was used to estimate the sample size for animal experiments. Experimental manipulation, data collection and processing were carried out using the double-blind method. Unless specified otherwise, the mice were provided with irradiated normal food and clean water. For purine supplementation, the mice received a high-adenine purified diet (Dyets, D191101) and consumed clean water containing 0.2% guanosine and 0.2% inosine. A total of 1 × 10^7^ cells were injected into the right flank. Tumor length and width were measured twice a week, and tumor volume was calculated based on length × width^2^ × 0.5. Once the tumor volume reached 100 mm^3^, specific subgroups were administered DOX or PAE. For DOX treatment, 10 mg/ml DOX was injected intraperitoneally into the mice once every seven days. In PAE treatment, 40 mg/kg PAE was injected intraperitoneally once every three days. The experiment concluded on day 28 after the initial administration, and the mice were sacrificed. Tumor tissues and corresponding organs were used for subsequent analyses. The study was approved by the Laboratory Animal Ethics Committee of the Renmin Hospital of Wuhan University in accordance with Basel Declaration (NO. WDRM-20230410D).

### Metabolomics analysis

The obtained samples were then dried and quenched in liquid nitrogen. The samples were weighed and ground, and metabolites were extracted using pre-cooled methanol, acetonitrile, and water (v/v/v, 2:2:1). Metabolomic profiling was performed using a UPLC-ESI-Q-Orbitrap-MS system (UHPLC, Shimadzu Nexera X2 LC-30AD, Shimadzu, Japan) coupled with Q-Exactive Plus (Thermo Scientific, San Jose, USA). The metabolites were identified by accuracy mass (mass tolerance < 10 ppm) and MS/MS data (mass tolerance < 0.02 Da) which were matched with HMDB, Massbank, and other public databases, and a self-built metabolite standard library from Bioprofile (China). Multivariate data analysis was performed using R software (version 4.0.3). Metabolites with a fold change greater than 2, a VIP value greater than 1.0, and a p-value less than 0.05 were considered statistically significant. The heat map is visualized using the ComplexHeatmap R package. The Scale function is used for normalization. The differential metabolite data were analyzed for KEGG pathways, which were enriched and statistically significant at p < 0.05. KEGG-based pathway enrichment analysis of metabolites was performed using the clusterProfiler R package.

### RNA extraction and quantitative real-time PCR (qPCR)

Total RNA was extracted using the TRIzol reagent (Takara, # 9108), followed by reverse transcription into cDNA using the Hifair® III 1st Strand cDNA Synthesis SuperMix (Yeasen, 11141ES60) according to the manufacturer’s instructions. SYBR (Yeasen, 11201ES08), primers, and cDNA were mixed in the recommended proportions as per the manufacturer’s instructions. The qPCR was conducted using Bio-Rad CFX96. Gene expression levels were normalized with actin used as an internal reference. Specific primer sequences are detailed in Supplementary Table 1.

### Western blot

Tissue and cellular proteins were extracted with pre-cooled RIPA, and protein concentration was confirmed using the BCA method. Denaturing SDS-PAGE was performed to separate the proteins based on their molecular weights, followed by the transfer of proteins to a PVDF membrane. Blocking was performed using 5% milk, followed by an overnight incubation with the primary antibody at 4 °C. The subsequent day, the secondary antibody was added post-washing, and the blot was visualized using enhanced chemiluminescence reagents (Epizyme, SQ101L). The antibodies employed are detailed in Supplementary Table 2.

### Immunofluorescence

Previous studies have used immunofluorescence staining [19]. Briefly, cells were fixed with 4% paraformaldehyde and permeabilized with 0.3% Triton X-100. Goat serum was used for the subsequent containment. Cells were incubated with the primary antibody at 4 °C overnight, and then incubated with a specific secondary antibody for 1 h at room temperature in the dark. For Phalloidin staining, cells were labeled with FITC-phalloidin (Yeasen, 40735ES75) according to the manufacturer’s instructions. Then, a moderate wash was performed after incubation with DAPI for 5 min at room temperature in the dark. For paraffin tissue sections, after antigen repair, permeabilization, and sealing, antibody incubation and DAPI staining were performed, as described previously. Observations were performed using a laser confocal microscope (Leica STELLARIS 5). Fluorescence co-localization was analyzed by LAS X software.

### Purinosomes labeling

The aggregation of purinosomes, a multienzyme complex, is thought to characterize intracellular purine depletion [20, 21]. Purinosomes were labeled as described previously [22]. Briefly, a mixture of PFAS-mCherry, serum-free medium, and DNA transfection reagent was added to the well-grown cells at an appropriate density. Cells were fixed using 4% paraformaldehyde at 48 h post-transfection, permeabilized, and stained with DAPI. Observations were performed using a laser confocal microscope. Aggregated red foci characterize the purinosomes. Finally, the number of foci representing purinosomes was counted for analysis.

### CCK8 assay and drug synergy analysis

The treated cells were incubated with the Cell Counting Kit (Yeasen, 40203ES60) for 30 min at 37 °C, according to the manufacturer’s instructions. The absorbance at 450 nm was determined using an enzyme marker and the cell inhibition rate was calculated. The synergy analysis was performed using SynergyFinder (https://synergyfinder.org). Briefly, the nature of the interaction was determined on the basis of the HSA synergy score. Zero represents additive interaction, negative values (green) represent antagonism, and positive values (red) represent synergy. The gray boxes represent the most synergistic regions.

### EDU uptake and staining

EDU-positive cells reflect proliferating cell populations and represent nucleotide synthesis. EDU uptake and staining were performed using the EDU Cell Proliferation Kit with Alexa Fluor 555 (CellorLab, CX003). Cells were cultured for 2 h at 37 °C in an EDU-containing medium. The cells were fixed and stained, as described in the instructions. Observations and picture acquisition were performed using an inverted microscope (OLYMPUS, IX71).

### DOX uptake

The cells were gently washed with PBS after appropriate interventions. DOX was prepared at a concentration of 2 µM using serum-free medium and co-incubated with the cells at 37 °C for 2 h. In one part, the cells were obtained and analyzed using a flow analyzer PC5.5. Phalloidin and DAPI staining were performed after fixation with 4% paraformaldehyde and observed under a laser confocal microscope to visualize the extent of DOX uptake.

### Comet assay

Comet assays were performed using a Comet Assay Kit (Abcam, ab238544). Briefly, the cells were treated with 100uM DOX for 45 min on ice to induce DNA damage and avoid repair. They were then incubated at 37 °C for 0, 0.5, 1, 2, or 4 h. The cells were obtained, processed, electrophoresed, and stained according to the manufacturer’s instructions. An orthogonal microscope (Olympus BX53F2) was used to capture images. The extent of DNA damage was quantified by the Tail Moment using the CASP software.

### Immunohistochemistry (IHC), HE staining and TUNEL staining

Briefly, samples were fixed, embedded in paraffin, and sectioned. Subsequently, HE staining, IHC or TUNEL staining is performed. HE staining was performed using the Hematoxylin and Eosin Staining Kit (Beyotime, C0105S), and TUNEL staining was performed using the One Step TUNEL Apoptosis Assay Kit (Beyotime, C1086). Perform IHC staining as previously described [23]. The antibodies used for IHC are detailed in Supplementary Table 2.

Use a scoring system for the IHC score. Start by scoring cell staining intensity: no positive staining (0), faint yellow (1), light brown (2), and dark brown (3). Next, score based on the percentage of positive cells: less than 25% (1), 26%-50% (2), 51%-75% (3), and greater than 75% (4). Multiply the two scores to get the final score. All tissue sections and IHC score were confirmed by a certified pathologist.

### Flow cytometry and apoptosis analysis

Apoptosis was detected using an Annexin V-Elab Fluor® Red 780/DAPI Apoptosis Kit (Elabscience, E-CK-A280). Briefly, culture medium and adherent cells were collected and washed with PBS. Cells were stained for 15 min, as indicated. The PB405 and APC-A750 channels of the flow analyzer were used to capture the DAPI and Annexin V signals. The percentage of apoptotic cells was obtained by summing PB405^low^ APC-A750^high^ with PB405^high^ APC-A750^high^.

### Bioinformatics analysis

Gene expression levels and prognostic status of patients with ovarian cancer were obtained from the Cancer Genome Atlas (TCGA) database. Normal ovarian genetic data used as controls were obtained from the Genotype-Tissue Expression (GTEx) database. The normalize function is used for normalization and to avoid batch effects. The limma R package was used to obtain p < 0.05 and |LogFC| greater than 2 for differentially expressed genes. Our previous sequencing data of SKOV3 and SKOV3/DDP were uploaded to the GEO database (GSE195765) and analyzed for differential genes according to the same criteria. Purine metabolism-related genes were obtained from the GeneCards database (accessed November 1, 2021) (Supplementary Table 3). The three groups of genes obtained above were intersected, and candidate key genes were identified by prognosis-based univariate COX regression and K-M analysis. Public IHC data from The Human Protein Atlas (HPA, accessed January 10, 2022). Promoter sequences were retrieved from the National Center for Biotechnology Information (accessed March 6, 2023). Motif sequences were obtained from the JASPAR database (accessed March 6, 2023).

### Co-inmunoprecipitation (Co-IP), silver staining, and IP-MS

Cells were placed in IP Lysis Buffer (Abbkine, BMP2020) and incubated on ice for 15 min. The cells were harvested and centrifuged at 12,000 rpm for 15 min at 4 °C. The supernatant was co-incubated with antibodies and magnetic beads (Biolinkedin, L-1004) at 4 °C overnight. Magnetic beads were harvested, gently washed and mixed with SDS loading buffer and boiled for immunoblotting. For silver staining, SDS-PAGE gels were obtained, fixed, and stained according to the instructions of the Fast Silver Stain Kit (Beyotime, P0017S). The gels were then cut and stored in pure water. IP-MS was performed using the iProteome (China). Briefly, after decolorizing the gel, the peptide extracts were freeze-dried, redissolved, and quantified. Peptides were analyzed using a Q Exactive HF-X Hybrid Quadrupole-Orbitrap Mass Spectrometer (Thermo Fisher Scientific) coupled with a high-performance liquid chromatography system (EASY nLC 1200, Thermo Fisher Scientific).

### Chromatin Immunoprecipitation (ChIP)

Chip experiments were performed using the ChIP Assay Kit (Beyotime, P2078) according to the manufacturer’s instructions. Briefly, the cells are pre-treated with methanol to cross-link the proteins to the DNA. The cells are then lysed and sonicated to shear the DNA, and incubated overnight with the corresponding antibody to capture the target protein. The next day, the product is washed several times according to the manufacturer’s instructions to obtain a product that can be used in subsequent operations.

### DR-GFP reporter assay

Briefly, cells were transfected with pDr-GFP and screened for stably transfected cells with puromycin (4 µg/ml). Cells were then transfected with the I-Sce1 plasmid for 24 h. Cells were harvested and after they were treated with DOX (100 µM) for 45 minutes and repaired for 2 h. Finally, GFP-positive cells were measured by flow cytometry. A total of three independent experiments were conducted.

### Protein purification and GST-pulldown

The target plasmid was transfected into BL21 (DE3), and the monoclonal bacteria were picked and placed in a resistant medium for overnight shaking at 37 °C. Subsequently, the culture was expanded to an OD600 value of 0.6. IPTG was added at a final concentration of 1 mM, and the culture was incubated overnight at 16 °C with shaking. Bacteria were collected by centrifugation and proteins were extracted using a Bacterial Protein Extraction Kit (Sangon, C600596). Target proteins were purified using protein purification kits (Biolinkedin, PK-2003, and PK-2004). SDS-PAGE gels carrying the target proteins were stained with Coomassie Blue to visualize the protein bands. His-FOXO3 protein was crosslinked with GST protein or GST-NEK6 protein and incubated with antibodies and magnetic beads at 4 °C with rotation. After appropriate elution, the proteins were obtained and analyzed by western blotting.

### Proximity ligation analysis (PLA)

PLA experiments and analysis were performed according to the manufacturer’s instructions. Briefly, plasmids were transfected in cells, which were then fixed and permeabilized to bind to the protein of interest using a specific primary antibody. The primary antibody is then labeled using a secondary antibody in NaveniFlex Cell MR (Navinci, 39505-100RXN). When the two proteins are close enough and interact, the DNA single strands in the secondary antibody probe are close together and replicate and amplify, resulting in red fluorescence.

### Protein activity analysis

NEK6 kinase activity was determined using a Human NEK6 ELISA Kit (Camilo, 2H-KMLJh315400). Briefly, after extracting cellular proteins on ice, antibodies and enzyme conjugates were added sequentially and incubated at 37 °C, as indicated. Subsequently, color development was performed. Absorbance was measured at 450 nm at the appropriate time. Finally, kinase activity was determined from the standard curve. ABCB1 activity assays were performed using Pgp-Glo™ Assay Systems (Promega, USA). Briefly, ABCB1 was blocked using 10 µM verapamil for 3 minutes or not, and then ATP was co-incubated with the samples according to the manufacturer’s instructions. The luminescent signal generated by fluorophores was detected and the ratio of the differences was finally calculated to obtain the relative ABCB1 activity. As described previously, we utilized 20 µM KO143 (MCE, HY-10010) for the activity assay of ABCG2 [24].

### Molecular docking

The protein structure of NEK6 was obtained from UniProt database (ID: Q9HC98). The sequence of the FOXO3 peptide was obtained from the PDB database (ID: 6MNL). The molecular structure of PAE was downloaded from PubChem (Compound CID: 11092). Molecular docking was performed using AutoDock, and the optimal conformation was selected. Visualization after docking was performed using PyMOL software.

### Dual luciferase reporter activity assays

Dual-luciferase reporter gene experiments were performed according to the manufacturer’s instructions (Yeason, 11402ES60). Briefly, the promoter sequence was cloned upstream of firefly luciferase. HEK293T cells were co-transfected with FOXO3 plasmid or control vector. Cells were lysed after 48 h, and the luminescence intensity was detected. Renilla luciferase was used as the internal reference.

### In vitro kinase assay

Add 200 µM ATP (Yeasen, 10129ES03) and substrate FOXO3 (3 µg) to purified NEK6 (1 µg). The mixture was incubated at 30 °C for 30 min. All fractions were collected and samples were subsequently analyzed by western blotting.

### Separation of nuclear and cytoplasmic proteins

Separate cytoplasmic proteins from nuclear proteins using a Nuclear Protein Extraction Kit (Abmart, #A10009). Briefly, cells were fully lysed on ice using a lysis solution, and the supernatant was collected after centrifugation as cytoplasmic proteins. After several washes, lysis was continued using a highly permeable lysate with sonication to obtain nuclear proteins.

### Statistical analysis

All studies were performed as at least three independent biological experiments. The student’s t-test was employed to compare results between the two groups. Comparative analyses among multiple groups were conducted using ANOVA. Data normality was assessed using the Shapiro-Wilk test, and homogeneity of variances was tested using Levene’s test. *P < 0.05 was considered statistically significant; **P < 0.01 or ***P < 0.001 was deemed more significant; ns, P ≥ 0.05 indicated that the difference was not statistically significant. Pearson correlation analysis was utilized to assess correlations between the two sets of data. Under the premise of P < 0.05, an R value below 0.1 suggests no correlation; R between 0.1 and 0.3 indicates weak correlation; R between 0.3 and 0.7 signifies moderate correlation, while R exceeding 0.7 implies strong correlation. Statistical plots were visualized using GraphPad Prism 9 or R software (version 4.0.3).

## Results

### Chemoresistant ovarian cancer exhibit elevated purine levels and robust de novo purine synthesis

**Fig. 1:**
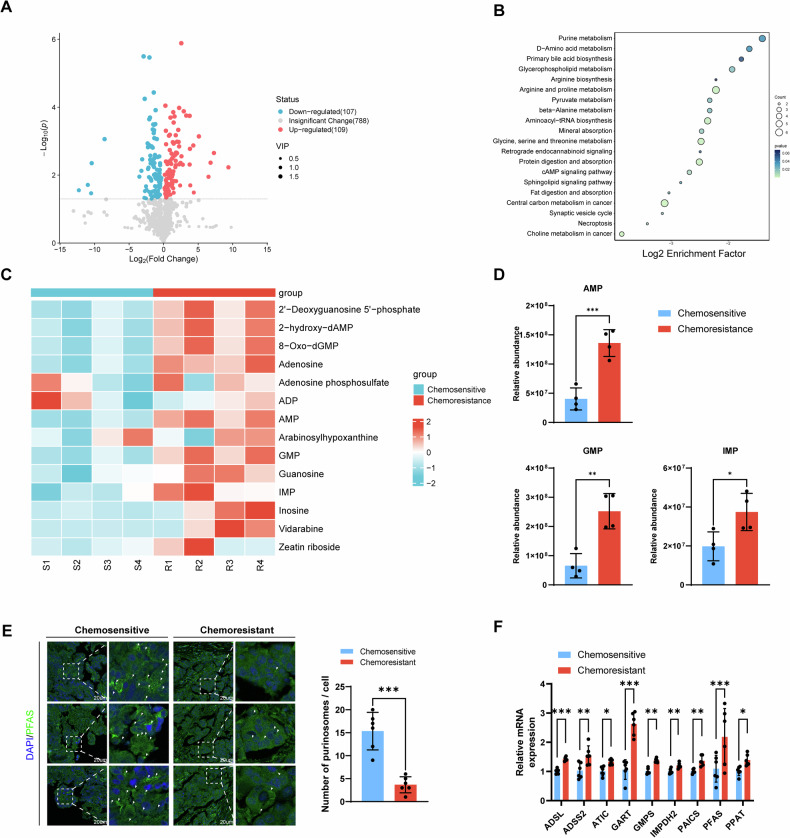
De novo purine synthesis is enhanced in chemoresistant ovarian cancer tissues. **A** Volcano plot showing differential metabolites in chemosensitive and chemoresistant ovarian cancer tissues. **B** Metabolic pathway enrichment map of differential metabolites. **C** UHPLC used to detect purine metabolite abundance in chemosensitive and chemoresistant ovarian cancer tissues, presented as a normalized heatmap. *N* = 4. **D** UHPLC analysis was conducted to assess the relative abundance of purine intermediates (AMP, GMP, and IMP) in chemosensitive and chemoresistant ovarian cancer tissues. *N* = 4. Data presented as mean ± SD. *P < 0.05; **P < 0.01; Student’s t-test. **E** Purinosome visualization in chemosensitive and chemoresistant ovarian cancer tissues. DAPI (blue) represents nuclei, and PFAS (green) indicates purinosomes. *N* = 6. Data presented as mean ± SD. ***P < 0.001; Student’s t-test. **F** mRNA levels of enzymes related to de novo purine synthesis in chemosensitive and chemoresistant ovarian cancer tissues. *N* = 6. Data presented as mean ± SD. *P < 0.05; **P < 0.01; ***P < 0.001; ANOVA.

To clarify the pivotal metabolic pathways influencing chemoresistance in ovarian cancer, we conducted metabolite analyses on ovarian cancer tissues from chemosensitive and chemoresistant cases. Characteristics of ovarian cancer patients are shown in Supplementary Table 4. A total of 216 distinct metabolites were identified and mapped onto various metabolic pathways (Fig. 1A, B). Notably, our attention was drawn to purine metabolism due to the upregulation of several purine metabolites in chemoresistant ovarian cancer tissues, as indicated by heat maps (Fig. 1C, Supplementary Table 5). Supporting this finding, the relative abundance of key purine intermediates (AMP, GMP, and IMP) is higher in chemoresistant ovarian cancer tissues (Fig. 1D). Furthermore, we assessed endogenous PFAS in tissues to characterize purinosomes, finding their deficiency in chemoresistant tissues and abundance in chemosensitive ones, indicating altered intracellular purine levels (Fig. 1E). Our previous review summarized these nine enzymes are the key enzymes that drive de novo purine synthesis [1]. Subsequently, mRNA levels of nine key enzymes involved in de novo purine synthesis were examined, indicating higher expression in chemoresistant tissues (Fig. 1F). These findings suggest elevated purine abundance and heightened de novo purine synthesis in chemoresistant ovarian cancer tissues.

We validated our findings at the cellular level using OVCAR8, SKOV3, and the corresponding paired multi-chemoresistant NCI/ADR-RES and SKOV3/DDP cells. Comprehensive metabolomics revealed that chemosensitive ovarian cancer cells exhibit lower intracellular purine levels (Fig. S1A, B, Supplementary Tables 6 and 7). Supporting this finding, the relative abundance of purine intermediates was higher in chemoresistant ovarian cancer cells compared to chemosensitive cells (Fig. S1C, D). Concurrently, higher levels of de novo purine synthesis enzyme mRNA were consistently present in chemoresistant cells (Fig. S1E, F). Cells were transfected with a plasmid encoding FGAMS-mCherry to visualize purinosome assembly using fluorescent fusion proteins (Fig. S1G). As expected, more purinosomes were observed in chemosensitive ovarian cancer cells. These results suggest that chemoresistant ovarian cancer cells possess a more abundant intracellular purine pool and higher purine metabolic flux than chemosensitive ovarian cancer cells.

### Purines contribute to DNA damage repair and chemoresistance in ovarian cancer cells

**Fig. 2:**
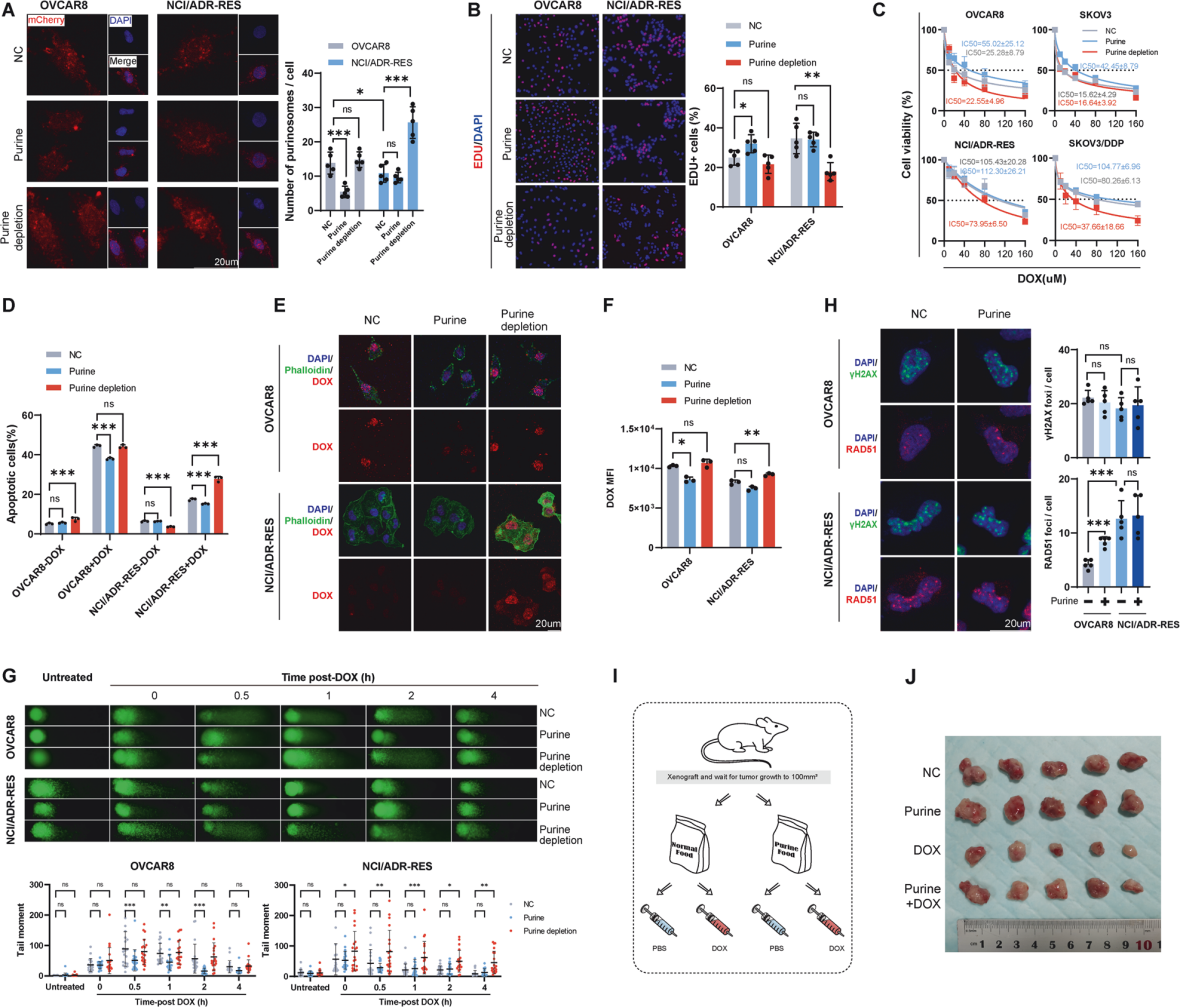
Purines contribute to DNA damage repair and chemoresistance in ovarian cancer cells. **A** Representative purinosome images and quantitative analysis after purine supplementation and depletion in OVCAR8 and NCI/ADR-RES cells. *N* = 5. Data presented as mean ± SD. *P < 0.05; ***P < 0.001; ns, not significant; ANOVA. **B** Representative images of EDU uptake after purine supplementation and depletion in OVCAR8 and NCI/ADR-RES cells. Quantification of EDU+ cell proportions. *N* = 5. Data presented as mean ± SD. *P < 0.05; **P < 0.01; ns not significant; ANOVA. **C** Cell viability curves in OVCAR8, NCI/ADR-RES, SKOV3, and SKOV3/DDP cells after purine supplementation and depletion in response to DOX treatment. IC50 values under different interventions are labeled. *N* = 3. Data presented as mean ± SD. **D** Impact of purine supplementation and depletion on DOX (60uM)-induced apoptosis in OVCAR8 and NCI/ADR-RES cells. *N* = 3. Data presented as mean ± SD. ***P < 0.001; ANOVA. **E** DOX uptake levels in OVCAR8 and NCI/ADR-RES cells treated with 2uM DOX after purine supplementation or depletion, followed by DAPI staining and observation under confocal microscopy. **F** Quantification of DOX uptake levels using flow cytometry in OVCAR8 and NCI/ADR-RES cells. *N* = 3. Data presented as mean ± SD. *P < 0.05; **P < 0.01; ns, not significant; ANOVA. **G** Alkaline comet assay performed on OVCAR8 and NCI/ADR-RES cells under different treatment conditions, with tail moment quantification. *N* = 20. **H** Observation of γH2AX and RAD51 foci levels after purine supplementation in OVCAR8 and NCI/ADR-RES cells following 2 h of DOX-induced DNA damage and repair. *N* = 5. Data presented as mean ± SD. ***, P < 0.001; ns, not significant; ANOVA. **I** Schematic representation of the construction and grouping of the ovarian cancer xenograft model. (**J**) Endpoint images of ovarian cancer xenografts derived from SKOV3 cells in each group. *N* = 5.

To explore the impact of purines on chemoresistance in ovarian cancer cells, interventions involving supplementation with permeable purine nucleosides or the creation of a purine-deficient environment were conducted. DOX was employed to evaluate the drug response. Purine intervention was verified by observing purinosome formation (Fig. 2A). Remarkably, purine supplementation notably enhanced proliferation and EDU incorporation in chemosensitive cells, while purine depletion hindered these processes in chemoresistant cells (Figs. 2B and S2A). Furthermore, purine depletion contributed to the chemosensitivity of NCI/ADR-RES and SKOV3/DDP cells (Figs. 2C, D and S2B, D). Conversely, purine supplementation impaired the chemosensitivity of OVCAR8 and SKOV3 cells. Apoptosis levels were quantified using flow cytometry (Figs. 2C, D and S2B-D). Overall, purine supplementation markedly boosts cell growth and chemoresistance, while purine depletion accelerates apoptosis. The former is particularly evident in chemosensitive cells, whereas the latter is primarily observed in chemoresistant cells. Moreover, no significant changes were found in the mRNA levels of enzymes related to de novo purine synthesis during DOX effects (Fig. S2E). Multiple chemotherapeutic agents bind to DNA, disrupting the DNA damage or repair processes that lead to cell death. We utilized the autofluorescence of DOX to investigate intranuclear drug retention, indicative of DNA binding. As anticipated, purine supplementation led to low DOX retention in chemoresistant cells but markedly increased DOX uptake in chemosensitive cells. Notably, purine degradation resulted in a significant accumulation of DOX in chemoresistant cells (Figs. 2E and S2F, G). Quantification of intracellular DOX mean fluorescence intensity (MFI) using flow cytometry yielded consistent results (Figs. 2F and S2H).

DOX induces DNA damage within half an hour, triggering a damage repair response. To validate the role of purines in this process, we conducted an alkaline comet assay to assess the extent of DNA damage in single cells (Figs. 2G and S3A). In general, purine supplementation significantly expedited DNA repair in chemotherapy-sensitive cells. Purine depletion markedly impeded the DNA damage repair process in chemoresistant cells. However, it remains unclear whether this is due to an intervention in the DNA damage process or post-damage repair. Intranuclear γH2AX and RAD51 foci reflect the extent of DNA damage and repair, respectively. The results indicate that, regardless of purine supplementation, DOX causes almost identical DNA damage in chemosensitive and chemoresistant cells (Figs. 2H and S3B-C). Chemoresistant cells demonstrate a heightened efficiency in DNA repair. Furthermore, purine supplementation enhances DNA repair efficiency in chemosensitive cells. Simultaneously, the DR-GFP experiment reveals that under DOX pressure, chemoresistant ovarian cancer cells demonstrate a heightened capacity for homologous recombination repair compared to their chemosensitive counterparts (Fig. S3D). More precisely, under DOX stress, purine supplementation enhances homologous recombination repair in chemosensitive ovarian cancer cells, whereas purine depletion impedes the homologous recombination repair capability of chemoresistant ovarian cancer cells.

We employed a tumor xenograft model derived from SKOV3 cells to validate these in vivo effects (Fig. 2I). In vivo, purine supplementation promoted tumor growth and mitigated DOX-induced cell death, leading to a reduction in tumor volume (Figs. 2J and S4A, B). Ki67 and TUNEL staining confirmed that purines facilitated tumor growth and conferred resistance to DOX (Fig. S4C). Additionally, these interventions did not induce liver or kidney damage (Fig. S4D). These results suggest that purines play a role in DNA damage repair and contribute to chemoresistance in ovarian cancer cells.

### Silencing NEK6 inhibits de novo purine synthesis and chemoresistance in ovarian cancer cells

In elucidating the association between de novo purine synthesis and chemoresistance in ovarian cancer, we aimed to pinpoint the pivotal genes governing chemoresistance. Through a comprehensive approach encompassing genetic differential analysis, including purine metabolism-related genes from GENECARDS, ovarian tumor tissue, paraneoplastic tissue data from TCGA and GTEx, and previously conducted sequencing data in SKOV3 and SKOV3/DDP cell lines, coupled with prognostic analysis, we identified 11 candidate key genes (Fig. S5A). Analyzing protein IHC data from the HPA database, we observed elevated expression of PKD1 and NEK6 proteins in ovarian cancer compared to normal ovarian tissue (Fig. S5B). Next, in TCGA-OV, we conducted correlation analysis between the mRNA expression profiles of PKD1 and NEK6 and de novo purine synthesis enzymes. The results revealed a positive correlation between both PKD1 or NEK6 with de novo purine synthesis enzymes (Fig. S5C). In this study, our focus shifted to a more in-depth exploration of NEK6, given its relatively lesser-known association with tumors.

We acquired 12 pairs of ovarian tumor tissue specimens from Renmin Hospital of Wuhan University, comprising 6 pairs of chemosensitive ovarian cancers and 6 pairs of chemoresistant ovarian cancers. Our investigation revealed a marked increase in NEK6 expression intensity in cancer tissues compared to paraneoplastic tissues (Fig. S6A). Notably, chemoresistant ovarian cancer tissues exhibited significantly elevated NEK6 protein levels compared to their chemosensitive counterparts (Fig. S6B). Integrating these observations, we posit that NEK6 may serve as a pivotal nexus linking chemoresistance and de novo purine synthesis in ovarian cancer. This speculation gained further support as we validated higher NEK6 expression in chemoresistant ovarian cancer cells within in vitro cell line models (Fig. S6C, D).

**Fig. 3:**
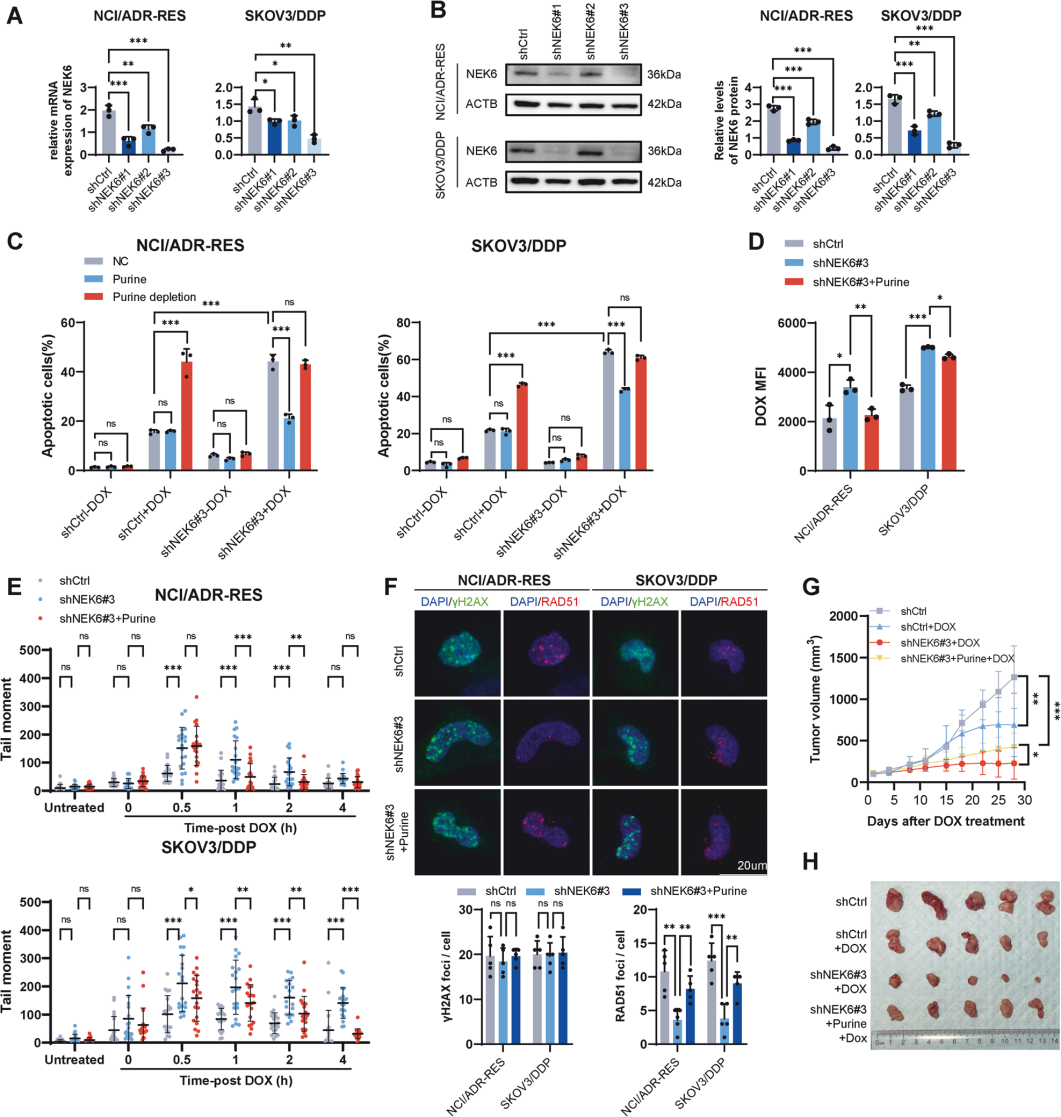
Silencing NEK6 inhibits de novo purine synthesis and chemoresistance in ovarian cancer cells. **A** mRNA levels of NEK6 knockdown. *N* = 3. Data presented as mean ± SD. *P < 0.05; **P < 0.01; ***P < 0.001; ANOVA. **B** Western blot reveals protein levels of NEK6 knockdown. *N* = 3. Data presented as mean ± SD. **P < 0.01; ***P < 0.001; ANOVA. **C** Flow cytometry detection of apoptosis levels in cells after NEK6 knockdown and purine supplementation or depletion, followed by 24 h of DOX treatment (60 µM DOX for NCI/ADR-RES and 30 µM DOX for SKOV3/DDP). *N* = 3. Data presented as mean ± SD. ***P < 0.001; ns not significant; ANOVA. **D** Quantification of DOX uptake levels in each group using flow cytometry in NCI/ADR-RES and SKOV3/DDP cells. *N* = 3. Data presented as mean ± SD. *P < 0.05; **P < 0.01; ***P < 0.001; ANOVA. **E** Alkaline comet assay performed on NCI/ADR-RES and SKOV3/DDP cells under different treatment conditions, with tail moment quantification. *N* = 20. **F** Observation of γH2AX and RAD51 foci levels after purine supplementation in NEK6 knockdown cells following 2 h of DOX-induced DNA damage and repair. *N* = 5. Data presented as mean ± SD. **P < 0.01; ***P < 0.001; ns not significant; ANOVA. **G** Growth curves of ovarian cancer xenografts under NEK6 knockdown or purine intervention. *N* = 5. Data presented as mean ± SD. *P < 0.05; **P < 0.01; ***P < 0.001; ANOVA. **H** Endpoint images of ovarian cancer xenografts obtained at the endpoint.

To validate the involvement of NEK6 in de novo purine synthesis, we conducted NEK6 knockdown in chemoresistant cells (Fig. 3A, B). Cells infected with sequence #3 exhibited optimal mRNA and protein knockdown efficiency for subsequent investigations. The reduced mRNA levels of de novo purine synthesis enzymes and the aggregation of purinosomes indicated decreased intracellular purine levels in NEK6 knockdown cells. (Fig. S7A, B). Despite the strong inhibition of NCI/ADR-RES and SKOV3/DDP cell proliferation upon NEK6 silencing, this effect was reversed by purine supplementation (Fig. S7C). Furthermore, purine supplementation reverses the increased chemosensitivity and DOX uptake caused by NEK6 silencing (Fig. 3C, D and S7D, E). Notably, transporter proteins ABCB1 and ABCG2 remained unchanged at the protein level, even though NEK6 knockdown facilitated DOX uptake (Fig. S7F). The protein activities of ABCB1 and ABCG2 remained unaffected by NEK6 knockdown (Fig. S7G). This implies that NEK6 induces chemoresistance by not interfering with drug influx or efflux modalities.

Alkaline comet assay and intranuclear γH2AX/RAD51 foci revealed that purine supplementation restored the DNA damage repair capacity inhibited by NEK6 silencing (Fig. 3E, F, Fig.S8A). Furthermore, DR-GFP experiments demonstrate that purine supplementation can restore homologous recombination repair capacity in cells compromised by NEK6 inhibition under DOX stress (Fig. S8B, C). In vivo, NEK6 knockdown significantly enhances sensitivity to DOX in ovarian cancer xenografts derived from SKOV3/DDP cells and reduces tumor volume (Figs. 3G, H and S8D). However, purine supplementation rescued tumor volume after DOX treatment and NEK6 knockdown. Ki67 staining indicated that NEK6 knockdown cells’ ability to grow was significantly inhibited under DOX stress, but this inhibition was alleviated by purine supplementation (Fig. S8E). Furthermore, purine supplementation reduced apoptosis induced by DOX in NEK6-knockdown ovarian cancer xenografts. In conclusion, our data suggest that NEK6 may promote chemotherapy resistance and DNA damage repair in ovarian cancer by activating de novo purine synthesis.

### NEK6 modulation of C-MYC protein regulates de novo purine synthesis and chemoresistance

**Fig. 4:**
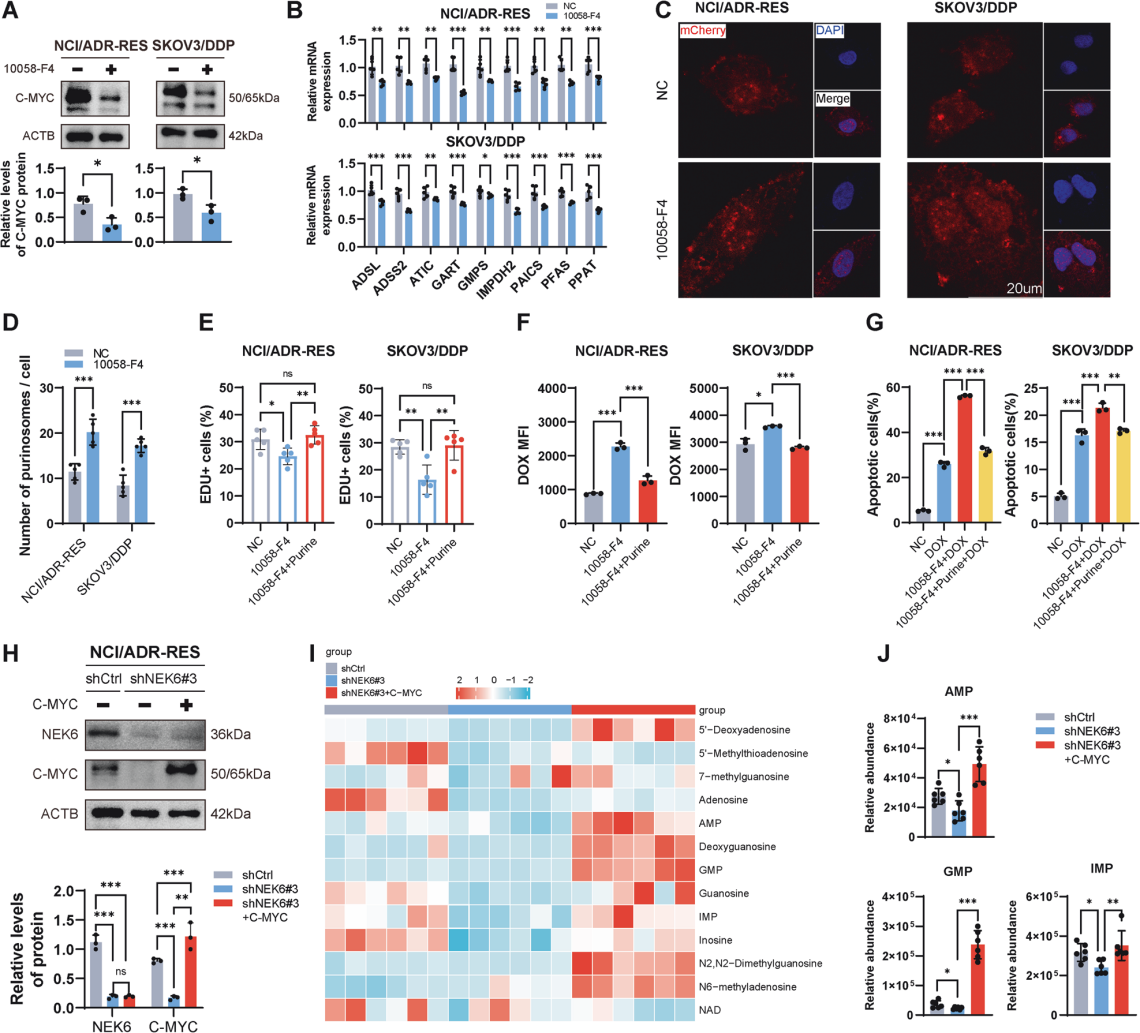
NEK6 modulation of C-MYC protein regulates de novo purine synthesis and chemoresistance. **A** Changes in C-MYC protein levels in NCI/ADR-RES and SKOV3/DDP cells after 10058-F4 (8 µM) treatment. *N* = 3. Data presented as mean ± SD. *P < 0.05; Student’s t-test. **B** Changes in mRNA levels of de novo purine synthesis-related enzymes after 10058-F4 treatment. *N* = 5. Data presented as mean ± SD. *P < 0.05; **P < 0.01; ***P < 0.001; ANOVA. **C** Representative images of purine bodies in cells after 10058-F4 intervention. **D** Quantification of purinosomes. *N* = 5. Data presented as mean ± SD. ***P < 0.001; ANOVA. **E** Impact of 10058-F4 and subsequent purine supplementation on cell proliferation based on EDU detection. *N* = 5. Data presented as mean ± SD. *P < 0.05; **P < 0.01; ns, not significant; ANOVA. **F** Flow cytometry detection of the influence of 10058-F4 and subsequent purine supplementation on intracellular DOX uptake. *N* = 3. Data presented as mean ± SD. *P < 0.05; ***P < 0.001; ANOVA. **G** Flow cytometry detection of the effect of 10058-F4 and subsequent purine supplementation on DOX-induced cell apoptosis. **H** Protein expression levels after NEK6 knockdown and C-MYC introduction. *N* = 3. Data presented as mean ± SD. **P < 0.01; ***P < 0.001 ns, not significant; ANOVA. **I** Heatmap of normalized abundance of purine metabolites after NEK6 knockdown and C-MYC introduction. *N* = 6. **J** Relative abundance of purine intermediates. *N* = 6. Data presented as mean ± SD. *P < 0.05; **P < 0.01; ***P < 0.001; ANOVA.

The crucial role of C-MYC in de novo purine synthesis metabolism is well-documented, yet its significance in ovarian cancer remains unexplored. GeneMANIA revealed a comprehensive association of C-MYC with genes related to enzymes crucial for de novo purine synthesis (Fig. S9A). Using 10058-F4 (8 µM), a C-MYC inhibitor, we targeted C-MYC protein level (Fig. 4A). As anticipated, the administration of 10058-F4 significantly reduced mRNA levels of de novo purine synthesis enzymes in NCI/ADR-RES and SKOV3/DDP (Fig. 4B). Additionally, purinosome formation was inhibited (Fig. 4C, D). The use of another C-MYC inhibitor, IZTZ-1 (5 µm), produced the similar effect (Fig. S9B-D). These results suggest that C-MYC mediates de novo purine synthesis in chemoresistant ovarian cancer cells. In addition, we found that 10058-F4 inhibited the proliferation of chemoresistant cells, and subsequent purine supplementation counteracted this effect (Fig. 4E). Notably, 10058-F4 enhanced DOX uptake and DOX-induced apoptosis in NCI/ADR-RES and SKOV3/DDP cells (Fig. 4F, G and S9E). However, the additional purine supplementation reversed this effect. These results imply that C-MYC serves as a crucial mediator in de novo purine synthesis, correlating with chemoresistance in ovarian cancer cells.

The complex relationship between NEK6 and C-MYC, which potentially underpins de novo purine synthesis, caught our attention. To elucidate this relationship, we overexpressed C-MYC in NEK6-knockdown NCI/ADR-RES cells to clarify uncertainties (Fig. 4H). Remarkably, C-MYC levels decreased with NEK6 knockdown, while the overexpression of c-MYC didn’t significantly elevate NEK6 levels, suggesting NEK6 operates as an upstream regulator of C-MYC. Metabolomics unveiled that NEK6 knockdown suppressed intracellular purine metabolite abundance, and C-MYC supplementation rescued this depletion (Fig. 4I, Supplementary Table 8). Specifically measuring key purine intermediates (AMP, GMP, and IMP), we found their levels decreased with NEK6 knockdown, and introducing C-MYC rescued the reduction caused by NEK6 knockdown (Fig. 4J).

### Regulation of C-MYC by NEK6 involves stability and phosphokinase activity

**Fig. 5:**
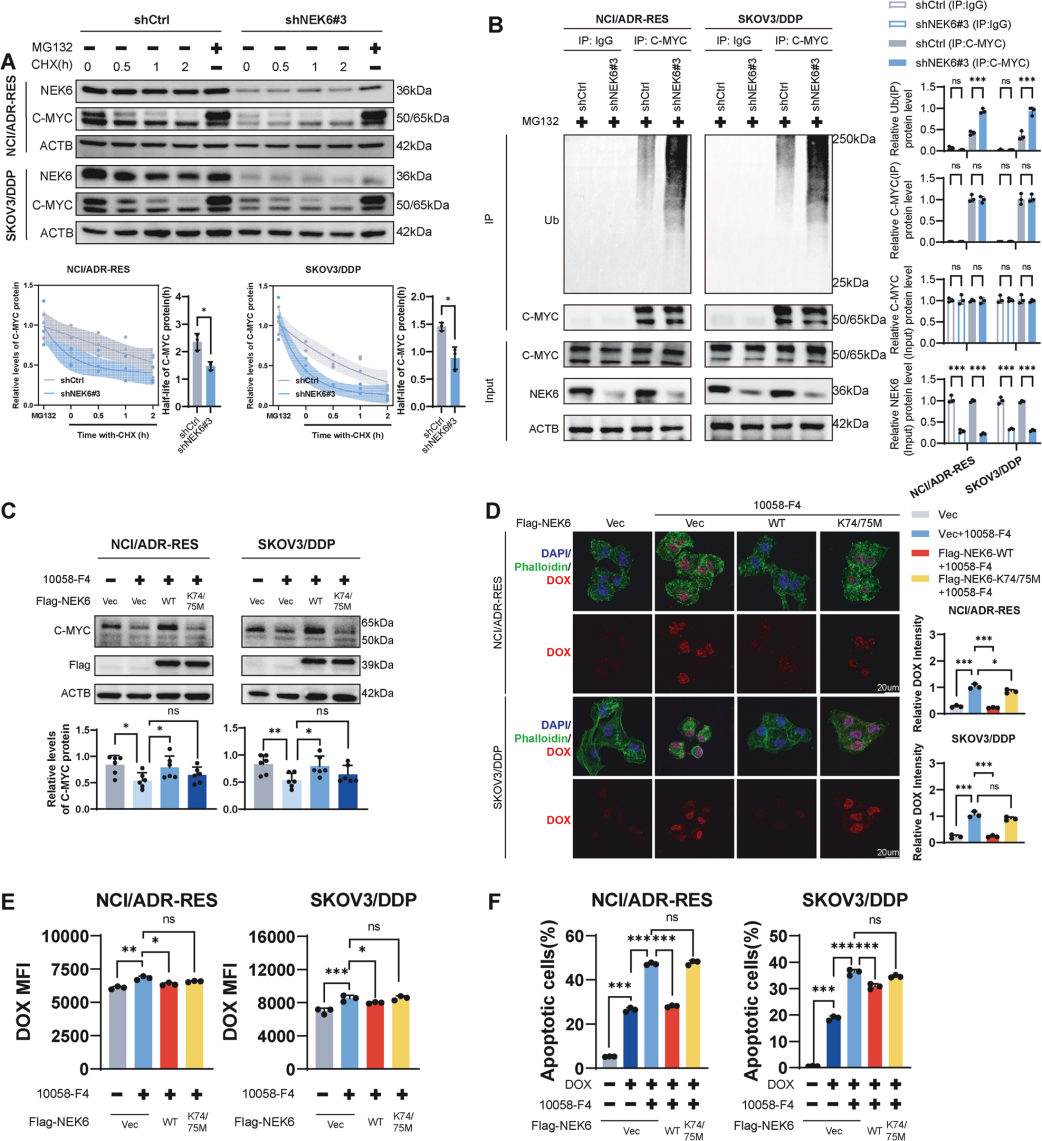
Regulation of C-MYC by NEK6 involves stability and phosphokinase activity. **A** Assessment of the impact of NEK6 knockdown on C-MYC protein stability in NCI/ADR-RES and SKOV3/DDP cells using CHX and MG132. *N* = 3. Data presented as mean ± SD. *P < 0.05; Student’s t-test. **B** Influence of NEK6 knockdown on C-MYC ubiquitination. *N* = 3. Data presented as mean ± SD. **P < 0.01; ***P < 0.001 ns, not significant; ANOVA. (C) Detection of C-MYC protein levels with interventions of wild-type NEK6-WT and kinase-dead NEK6-K74/75 M. *N* = 6. Data presented as mean ± SD. *P < 0.05; **P < 0.01 ns, not significant; ANOVA. **D** Laser confocal-based visualization of DOX uptake levels. Quantifying the relative intensity of DOX in cells. *N* = 3. Data presented as mean ± SD. *P < 0.05; **P < 0.01 ns, not significant; ANOVA. **E** Flow cytometry detection of the combined intervention of NEK6-WT, NEK6-K74/75 M, and 10058-F4 on intracellular DOX uptake levels. *N* = 3. Data presented as mean ± SD. *P < 0.05; **P < 0.01 ns, not significant; ANOVA. **F** Flow cytometry detection of the combined intervention of NEK6-WT, NEK6-K74/75 M, and 10058-F4 on DOX-induced cell apoptosis levels. *N* = 3. Data presented as mean ± SD. ***P < 0.001; ns not significant; ANOVA.

Moreover, we assessed C-MYC protein stability utilizing MG132 and CHX (Fig. 5A). Our findings indicate that NEK6 plays a crucial role in sustaining C-MYC stability, with cells lacking NEK6 exhibiting a faster rate of C-MYC degradation. Subsequently, we explored ubiquitination levels, a predominant post-translational modification influencing C-MYC degradation. Remarkably, NEK6 knockdown cells demonstrated higher endogenous ubiquitin binding to C-MYC (Fig. 5B).

NEK6 belongs to the phosphokinase family. To elucidate the potential link between NEK6 and C-MYC, we introduced a structural mutation in the NEK6 kinase domain, generating a K74/75M mutant devoid of kinase activity [25]. Notably, the overexpression of NEK6-WT, but not NEK6-K74/75M, significantly reversed 10058-F4-induced c-MYC depletion (Fig. 5C). Furthermore, NEK6-WT, but not NEK6-K74/75 M, impeded the 10058-F4-induced enhancement of DOX uptake (Fig. 5D, E). Apoptotic analysis revealed that NEK6-WT rescued apoptosis resulting from 10058-F4-mediated C-MYC downregulation; however, NEK6-K74/75M did not exhibit this capability (Fig. 5F, Fig. S9F). These indicate that the kinase activity of NEK6 plays a crucial regulatory role in maintaining C-MYC-mediated chemoresistance.

### NEK6 phosphorylates FOXO3 at S7 site and prevents FOXO3 nuclear translocation

**Fig. 6:**
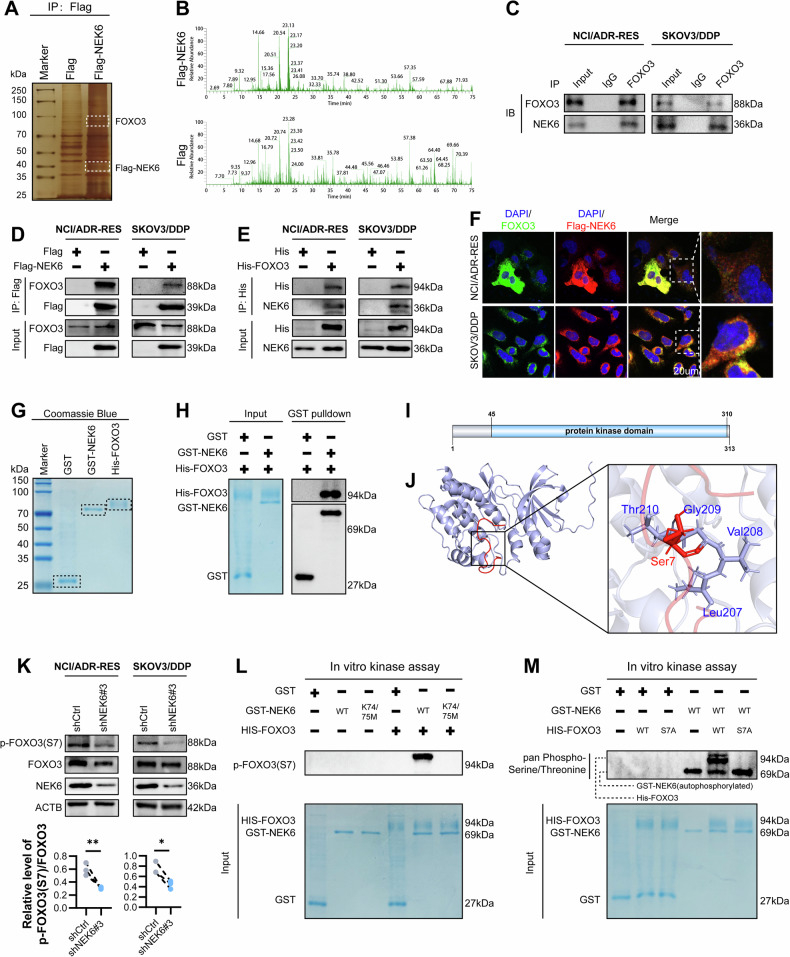
NEK6 phosphorylates FOXO3 at S7 site and prevents nuclear translocation. **A** IP was performed using Flag tags followed by silver staining to visualize the protein blot. **B** Representative images of IP-MS. **C** Co-IP detection of endogenous interactions between NEK6 and FOXO3. **D** Detection of interactions between exogenous NEK6 and FOXO3. **E** Detection of interactions between exogenous FOXO3 and NEK6. **F** Spatial co-localization of NEK6 with FOXO3. **G** Coomassie Blue staining of GST derived from purified proteins of E. coli, GST-NEK6, and His-FOXO3. **H** The GST pull-down assay detects the direct interaction of NEK6 with FOXO3. (**I**) Schematic diagram of the structural domain of NEK6 protein kinase. **J** Molecular docking of NEK6 with FOXO3 peptide by Autodock. **K** Effect of NEK6 knockdown on FOXO3 S7 phosphorylation level. *N* = 3. Data presented as mean ± SD. *P < 0.05; **P < 0.01; Student’s t-test. **L** Direct phosphorylation of FOXO3 S7 by NEK6-WT and NEK6-K74/75M detected by in vitro kinase assays. **M** Direct phosphorylation of FOXO3-WT and FOXO3-S7A by NEK6-WT was detected by in vitro kinase assays.

We attempted to identify the key connecting role of NEK6 and C-MYC. We performed IP on cells loaded with the control vector and Flag-NEK6, silver-stained, and analyzed by mass spectrometry (Fig. 6A). IP-MS analysis of the NEK6 protein complex isolated from NCI/ADR-RES cells (Fig. 6B). We focused on FOXO3 because it is closely related to MYC and has been shown to be a substrate for NEK6. Meanwhile, the metabolic profiles of shCtrl and shNEK6#3 were comprehensively analyzed, and the metabolic interaction network map pointed to the FOXO family (Fig. S10A). Endogenous Co-IP further confirmed the interaction between NEK6 and FOXO3 (Fig. 6C). We exogenously overexpressed NEK6 or FOXO3 to further confirm this interaction (Fig. 6D-E). Immunofluorescence revealed spatial co-localization of NEK6 and FOXO3 in NCI/ADR-RES (Colocalization rate = 89.35 ± 3.17%) and SKOV3/DDP (Colocalization rate= 88.66 ± 1.31%) cells (Fig. 6F). Furthermore, we produced and purified NEK6 and FOXO3 proteins using an Escherichia coli system (Fig. 6G). GST-pulldown assay demonstrated that NEK6 directly binds to FOXO3 (Fig. 6H). Further, to confirm that intrinsic direct protein interactions occur in chemoresistant and chemosensitive ovarian cancer cells, we took PLA experiments. The results showed that NEK6 was able to exert a direct interaction with FOXO3 in both chemosensitive and chemoresistant ovarian cancer cells. However, it cannot be ignored that the extent of this interaction is much lower in chemosensitive cells than in chemoresistant cells (Fig. S10B).

As a protein kinase, the phosphokinase activity of NEK6 is of great interest to us. Previous studies have reported that FOXO3 may be a phosphorylation substrate of NEK6 [18]. We obtained the range of protein kinase domains of NEK6 from UniProt (Fig. 6I). Using computational modeling based on Autodock and PyMOL, we found that FOXO3(S7), a serine site susceptible to phosphorylation, docked with the NEK6 kinase structural domain (Fig. 6J). Subsequently, we found that NEK6 knockdown inhibited FOXO3 S7 phosphorylation in chemoresistant ovarian cancer cells (Fig. 6K). More importantly, nucleoplasmic separation experiments demonstrated that FOXO3 S7 phosphorylation might be primarily located in the cytoplasm rather than in the nucleus (Fig. S10C). In addition, NEK6 knockdown promoted the translocation of FOXO3 to the nucleus. We also used immunofluorescence to visualize the changes in nuclear translocation (Fig. S10D). We then overexpressed NEK6 in chemosensitive cells, both wild-type and K74/75M kinase-dead. Notably, annihilation of NEK6 kinase activity significantly inhibited FOXO3 S7 site phosphorylation (Fig. S10E). Furthermore, FOXO3 nuclear translocation increased with NEK6 kinase death (Fig. S10F).

To understand whether NEK6 directly causes phosphorylation of FOXO3 S7 or whether some other bypass ultimately mediates this result, we performed in vitro kinase assays. As previously expected, the kinase-active form of NEK6 is able to directly phosphorylate FOXO3 S7, and the kinase-dead variant of NEK6 is unable to perform this function (Fig. 6L). We generated an S7 site dephosphorylation mutant, FOXO3-S7A, for further validation. Pan-phosphorylation assays were performed on the samples and showed that when S7A was mutated, phosphorylation of FOXO3 by NEK6 was barely visible (Fig. 6M). These results suggest that NEK6 directly phosphorylates the FOXO3 S7 site.

### FOXO3 contributes to C-MYC ubiquitination

**Fig. 7:**
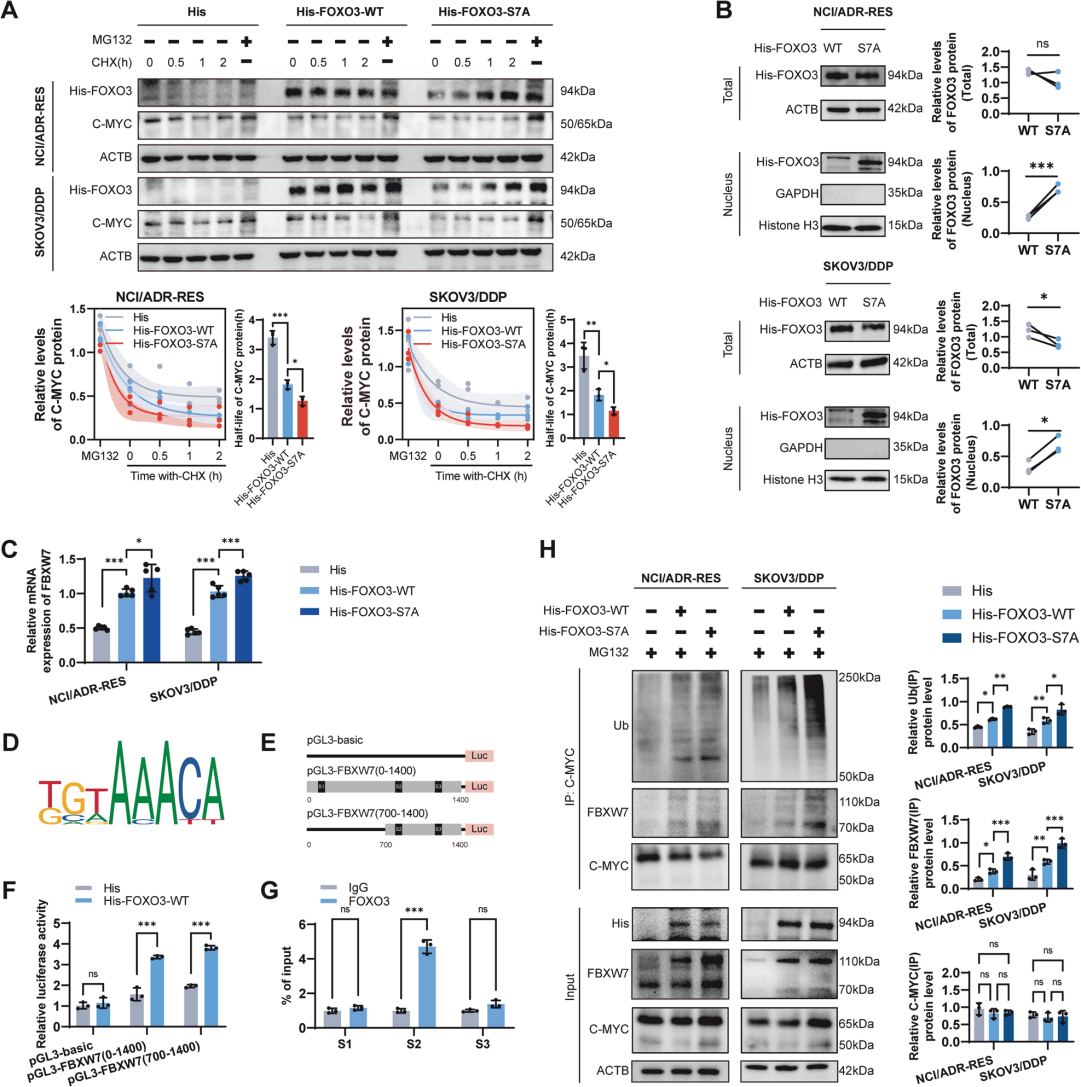
Nuclear FOXO3 contributes to C-MYC ubiquitination. **A** Impact of introducing FOXO3-WT and FOXO3E-S7A on C-MYC protein stability. *N* = 3. Data presented as mean ± SD. *P < 0.05; **P < 0.01; ***P < 0.001; ANOVA. **B** Effect of introducing FOXO3-WT and FOXO3E-S7A on FOXO3 protein nuclear translocation. *N* = 3. Data presented as mean ± SD. *P < 0.05; ***P < 0.001; ns, not significant; Student’s t-test. **C** Detection of mRNA levels of FBXW7 after introducing FOXO3-WT and FOXO3E-S7A. *N* = 3. Data presented as mean ± SD. *P < 0.05; ***P < 0.001; ANOVA. **D** Motif sequence of FOXO3. **E** Schematic of promoter truncation sequences for luciferase gene reporter analysis experiments. **F** Dual-luciferase reporter gene assay detecting FOXO3 activation of FBXW7 transcription in HEK293T cells. The relative luciferase activity reflects the degree of activation. *N* = 3. Data presented as mean ± SD. ***P < 0.001; ns, not significant; ANOVA. **G** ChIP-qPCR detecting the binding between FOXO3 and the FBXW7 promoter. *N* = 3. Data presented as mean ± SD. ***P < 0.001; ns, not significant; ANOVA. **H** Co-IP detecting the level of binding and ubiquitination between C-MYC and FBXW7 after introducing FOXO3-WT and FOXO3E-S7A. *N* = 3. Data presented as mean ± SD. *P < 0.05; **P < 0.01; ***P < 0.001; ns not significant; ANOVA.

We observed that FOXO3 overexpression, particularly FOXO3-S7A, hindered the protein stability of C-MYC, facilitating its rapid degradation (Fig. 7A). FOXO3-S7A exhibited enhanced nuclear aggregation compared to FOXO3-WT (Fig. 7B). In our exploration of the link between FOXO3 and C-MYC, FBXW7 emerged as a noteworthy candidate, given its role in C-MYC ubiquitination according to recent studies [26–28]. Although the relationship between FOXO3 and FBXW7 was previously unclear, we demonstrated that exogenous FOXO3, particularly the S7A mutant, elevated FBXW7 mRNA levels (Fig. 7C). The motif sequence of FOXO3 from JASPAR revealed three binding sites in the 1400 bp promoter region of FBXW7 (Fig. 7D, E). Subsequently, dual-luciferase reporter assays indicated that FOXO3 transcriptionally regulates FBXW7, mainly activating its promoter region between 700–1400 bp (Fig. 7F). ChIP-qPCR experiments confirmed FOXO3 binding to the S2 (803-810) site of the FBXW7 promoter region (Fig. 7G). Moreover, overexpression of FOXO3, particularly the S7A mutant, increased FBXW7 protein levels, fostering the interaction between C-MYC and FBXW7, ultimately leading to ubiquitination (Fig. 7H).

### Correlations between NEK6, C-MYC, FOXO3, and purine intermediates in ovarian cancer tissues

We previously examined the expression of NEK6 in ovarian cancer tissues. We extended our analysis to include the expression of p-FOXO3 (S7) and C-MYC in ovarian cancer tissues. IHC results indicated higher IHC scores for p-FOXO3 (S7) and C-MYC in chemoresistant ovarian cancer tissues compared to chemosensitive ones (Fig. S11A). Pearson correlation analysis based on IHC scores revealed a positive correlation among the levels of these three biomarkers (Fig. S11B).

Furthermore, as we correlated the expression of these protein markers with the abundance of purine metabolic intermediates, we found a positive correlation between the IHC scores of these three markers and the abundance of purine intermediates (Fig. S11C-E).

### Paeonol (PAE) inhibits NEK6 and helps deter chemoresistance in ovarian cancer

**Fig. 8:**
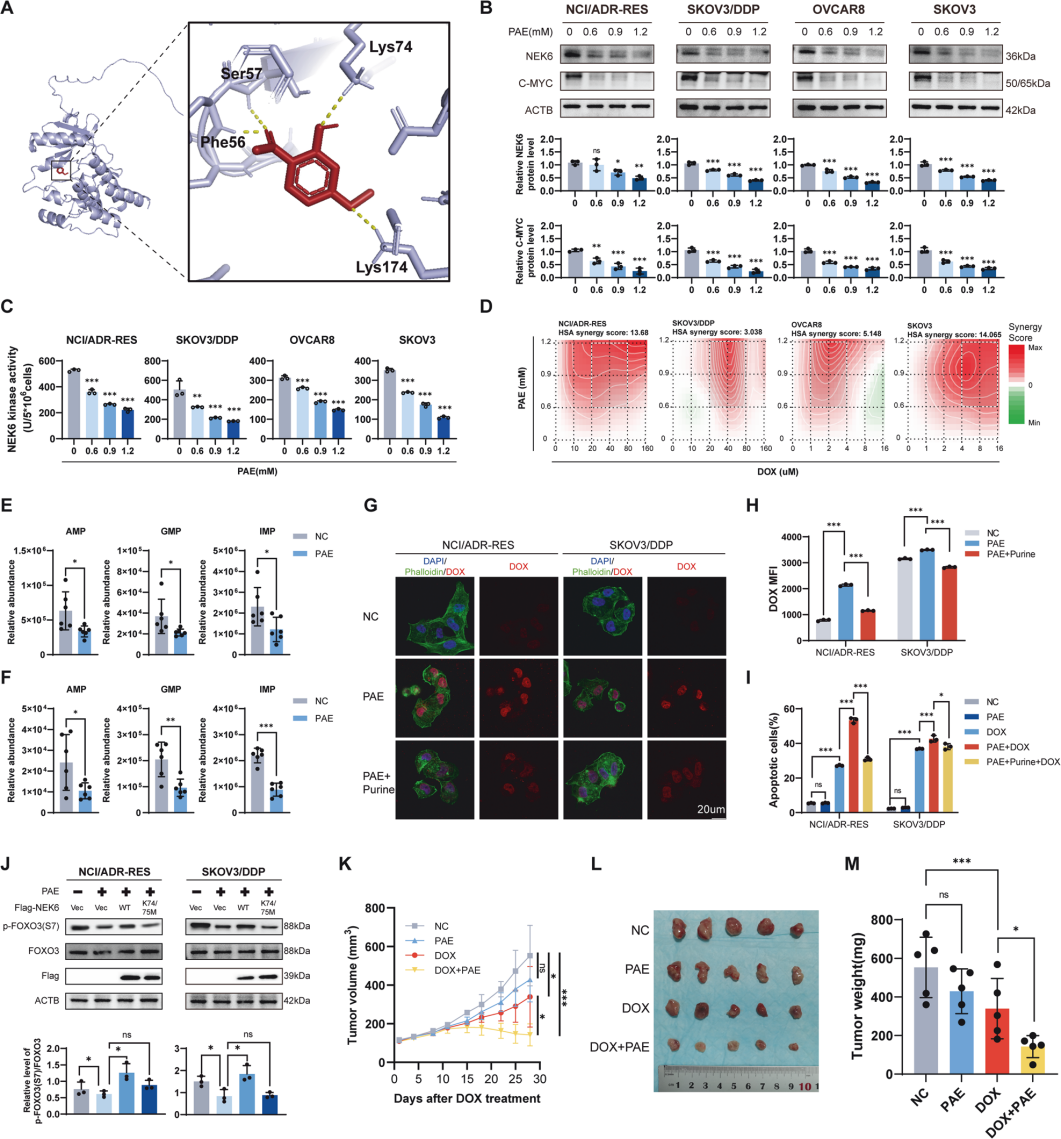
Targeting NEK6 with PAE inhibits de novo purine synthesis and overcomes ovarian cancer chemoresistance. **A** Molecular docking of NEK6 with PAE using Autodock. **B** Dose-dependent inhibition of NEK6 and C-MYC expression levels by PAE. *N* = 3. Data presented as mean ± SD. *P < 0.05; **P < 0.01; ***P < 0.001; ns not significant vs. the control group (0 mM); ANOVA. **C** Impact of PAE on NEK6 kinase activity. *N* = 3. Data presented as mean ± SD. **P < 0.01; ***P < 0.001 vs. the control group (0 mM); ANOVA. **D** Synergy analysis of PAE with DOX. Red represents synergistic effect, and green represents antagonistic effect. Color depth represents the intensity of action. **E** Effect of PAE (0.9 mM) on the relative abundance of purine intermediates in NCI/ADR-RES cells. *N* = 6. Data presented as mean ± SD. *P < 0.05; Student’s t-test. **F** Effect of PAE (0.9 mM) on the relative abundance of purine intermediates in SKOV3/DDP cells. *N* = 6. Data presented as mean ± SD. *P < 0.05; Student’s t-test. **G** Representative images of the impact of PAE and purine supplementation on intracellular DOX uptake under laser confocal microscopy. **H** Flow cytometry quantification of the impact of PAE and purine supplementation on intracellular DOX uptake levels. *N* = 3. Data presented as mean ± SD. ***P < 0.001; ANOVA. **I** Flow cytometry detection of the impact of PAE and purine supplementation on DOX-induced apoptosis levels. *N* = 3. *P < 0.05; ***P < 0.001; ns, not significant; ANOVA. **J** Impact of PAE, NEK6-WT, and NEK6-K74/75 M co-intervention on FOXO3 S7 phosphorylation. *N* = 3. Data presented as mean ± SD. *P < 0.05; ns, not significant; ANOVA. **K** Growth curves of ovarian cancer xenografts derived from SKOV3/DDP cells in various treatment groups. *N* = 5. Data presented as mean ± SD. *P < 0.05; ***P < 0.001; ns not significant; ANOVA. **L** Macroscopic images of ovarian cancer xenografts in each group at the endpoint. **M** Measurement of volumes of ovarian cancer xenografts derived from SKOV3/DDP cells at the endpoint. *N* = 5. Data presented as mean ± SD. *P < 0.05; ***P < 0.001; ns not significant; ANOVA.

Our earlier research has underscored the potential of PAE as a promising monomer in the fight against ovarian cancer [23]. Molecular docking models indicated a potential interaction between PAE and NEK6, with the most likely binding site located within the structural kinase domain of NEK6 (Fig. 8A). In vitro, PAE exhibited a dose-dependent reduction in the protein levels of NEK6 and C-MYC (Fig. 8B), accompanied by the inhibition of NEK6 kinase activity (Fig. 8C). In our exploration of the interaction between PAE and DOX in cancer inhibition, drug-drug interaction analysis revealed a broad synergistic effect in both chemosensitive and chemoresistant ovarian cancer cells (Fig. 8D). Considering the effective NEK6 inhibitory potency and synergistic interaction with DOX observed at 0.9 mM PAE, this concentration was employed for subsequent investigations. UHPLC analysis demonstrated that PAE significantly inhibited the levels of purine intermediates in NCI/ADR-RES and SKOV3/DDP cells (Fig. 8E, F). Concurrently, mRNA levels of de novo purine synthesis-related enzymes were also inhibited by PAE, indicating its role in deterring de novo purine synthesis in ovarian cancer cells (Fig. S12A). Laser confocal fluorescence and flow cytometry analyses unveiled that PAE elevated the degree of DOX binding in chemoresistant cells, a phenomenon rescued by subsequent purine supplementation (Fig. 8G, H and S12B). Furthermore, co-intervention with PAE and DOX significantly promoted apoptosis in NCI/ADR-RES and SKOV3/DDP, an effect that could be countered by purine supplementation (Fig. 8I, Fig. S12C, D). Multiple interventions on OVCAR8 and SKOV3 cells were conducted to fathom the complexity of changes in synergistic killing. Notably, this synergistic killing effect was mitigated by purine supplementation, the addition of C-MYC, or NEK6-WT (Fig. S12E, F). However, supplementation with NEK6-K74/75M, characterized by deficient kinase activity, proved ineffective. Mechanistically, PAE reduced FOXO3 S7 phosphorylation, and the addition of NEK6-WT, but not NEK6-K74/75 M, rescued defective FOXO3 S7 phosphorylation under PAE intervention (Fig. 8J)

In vivo, the combined treatment approach involving PAE and DOX demonstrated a significant reduction in the volume of ovarian cancer xenografts derived from SKOV3/DDP cells (Fig. 8K–M). Ki67 and TUNEL staining indicated that PAE contributed to DOX-induced arrest of proliferation and promotion of apoptosis of ovarian cancer xenografts in vivo (Fig. S12G). Furthermore, the inclusion of PAE exhibited reliable biosafety, with no significant damage observed in the heart, liver, spleen, lungs, kidneys, or intestines of the experimental animals (Fig. S12H). In summary, these findings indicate that PAE holds promise as an adjunct therapy for overcoming chemoresistance in ovarian cancer treatment.

## Discussion

Chemoresistance poses a significant challenge in cancer treatment, increasing the difficulty of therapy and often resulting in a poorer prognosis. Cancer’s aggressive behaviors, including chemoresistance, are frequently accompanied by distinct metabolic reprogramming features. However, the understanding of the underlying regulatory networks and their interactions with metabolism remains incomplete [29, 30]. The roles of glycolysis, lipid metabolism, iron metabolism, and amino acid metabolism in chemoresistance have been widely explored [8, 31–35]. It is noteworthy that highly malignant tumor cells exhibit a pronounced dependency on hyperactive nucleotide synthesis to support DNA synthesis, promote DNA damage repair, and shield tumor cells from the assault of chemotherapeutic agents [36]. In recent years, there has been increasing interest in the relationship between purine metabolism, an important pathway in nucleotide metabolism, and therapy resistance. Wahl DR et al. reported that purine metabolites, particularly guanylates, significantly induce radiation resistance in glioblastoma [37]. The specific mechanism is related to DNA protection from nucleosides. Noriko Gotoh et al. found that MTHFD2, a mitochondrial enzyme involved in one-carbon metabolism, mediates purine synthesis, depletes AICAR, and promotes tumor stemness and gefitinib resistance [38]. PAICS, a key enzyme for de novo purine synthesis, activates the cAmp/PKA signaling pathway, promotes mTOR activity, activates estrogen receptor alpha, and enhances estrogen dependence and tamoxifen resistance in breast cancer [39]. Our earlier investigation delineated aberrant changes in purine metabolism in ovarian cancer, yet its association with chemoresistance remained unclear [1]. In this study, we identified a significantly enriched purine pool in chemoresistant ovarian cancer. Moreover, augmenting purine level facilitated DNA damage repair post chemotherapeutic agent and fostered chemoresistance in chemosensitive cells. Conversely, depleting purines hindered DNA repair in chemoresistant cells, thereby enhancing chemosensitivity. We believe this phenomenon is associated with the intracellular purine abundance. Chemoresistant ovarian cancer cells have elevated purine levels, making purine supplementation relatively ineffective. In chemosensitive cells with low purine levels, adding purines swiftly restores their intracellular pool, fostering chemoresistance.

Oncogenes and tumor suppressor genes are directly or indirectly involved in cellular metabolic reprogramming to interfere with chemotherapy resistance in tumors. C-MYC is an oncogene that is present and overexpressed in various human solid tumors, influencing tumor metabolic reprogramming. Previous studies have indicated that C-MYC extensively participates in de novo purine synthesis in cancer cells [40–42]. Given its non-druggable nature, directly targeting it is impractical. Hence, exploring upstream regulatory factors of C-MYC for intervening in tumor de novo purine synthesis and chemotherapy resistance holds considerable importance. Unlike C-MYC-mediated purine metabolism, metabolic changes and regulation by NEK6, a serine/threonine kinase, remain largely unexplored. Although several studies have suggested that NEK6 is associated with malignant tumor features, the chemoresistance mediated by NEK6 has not been fully elucidated [15–17]. In our study, NEK6 was associated with chemoresistance and poor prognosis in ovarian cancer. NEK6 supports de novo purine synthesis and maintains the intracellular purine pool by preventing C-MYC ubiquitination degradation, thereby promoting DNA damage repair and driving chemoresistance. The function of NEK6 is reminiscent of its kinase activity. Chun-Li Zhang et al. reported that NEK6 directly phosphorylated STAT3, promoted reactive astrogliosis, and exacerbated brain damage [25]. NEK6, with kinase activity, has been reported to block SMAD4 nuclear translocation and prevent growth arrest in hepatocellular carcinoma cells [43]. Our findings suggest that kinase activity of NEK6 contributes to de novo purine synthesis and chemoresistance. Future studies should target NEK6 kinase activity or look for new kinases with its structural similarity. This will not only aid in uncovering overlooked functions of NEK6 but also contribute to the advancement of targeted therapies.

FOXO3 is known as a tumor suppressor transcription factor [44]. William C. Hahn et al. found that FOXO3 is phosphorylated substrate of NEK6 and acts at the S7 site [18]. Our results confirmed the direct interaction of NEK6 with FOXO3 and its ability to phosphorylate FOXO3 at the S7 site. However, little is known about the significance of S7 phosphorylation of FOXO3. Eric W-F Lam et al. reported that DOX or dexamethasone induced- FOXO3 S7 phosphorylation in breast cancer cells MCF7 and in B-ALL was predominantly localized in the nucleus [45, 46]. An observational study conducted by Bowen Wu et al. revealed an augmentation in both the nuclear translocation of FOXO3 and FOXO3 S7 phosphorylation following the induction of autophagy [47]. Notably, in our study, FOXO3 S7 phosphorylation was mainly found in the cytoplasm, but not in the nucleus of ovarian cancer cells. When S7 phosphorylation was disrupted by a single site mutation, FOXO3 accumulation was significantly increased in the nucleus. This may be due to cancer-specific localization in ovarian cancer. How do we interpret this difference? Firstly, the intricate intracellular signaling systems could trigger diverse responses. The phosphorylation and subcellular repositioning induced by drugs may be the ultimate outcome of various signaling regulations and compensatory reactions. Additionally, the functional variances mediated by cancer-specific protein modifications are also taken into consideration. These diverse outcomes offer a research space for the post-translational modifications and functions of FOXO3.

Considering the non-druggability of C-MYC, it is important to identify upstream targets for intervention [48]. Several natural and chemically synthesized compounds have been reported to exhibit NEK6 inhibitory activity [49–51]. Here, we found that PAE, a bioactive phenol, possesses NEK6 inhibitory activity and can inhibit de novo purine synthesis and chemoresistance in ovarian cancer. Our previous study reported the cytotoxic effect of PAE on ovarian cancer cells [23]. Recent studies have reported a deterrent effect of PAE against chemoresistance in other solid human tumors, involving MAPK signaling and regulation of Transgelin 2 [52, 53]. In clinical medicine, PAE has been shown to have a favorable safety profile and high efficiency. Although PAE has no precedent for use in tumor therapy, it may be a candidate for chemoresistance against ovarian cancer.

Another direction that still needs to be explored is whether we can curb tumor malignancy through dietary intervention [54]. A common perception is that obesity and alcohol are major nutritional contributors to cancer [54]. Fasting therapies, including intake restriction and ketogenic diets, are thought to be synergistic with anticancer therapy [55]. Specific amino acid-restricted and fat-restricted diets have gained initial effectiveness in anticancer therapy. The relationship between purine diets and tumor development has rarely been explored, and future in-depth observations are still needed to judge this.

There are currently unavoidable limitations of this study. First, although it is clear that chemoresistance in tumors is heterogeneous, we could not use primary cells in this study because of the limited availability of clinical samples. Besides, Chemotherapy is a common outcome of a combination of multiple drugs. In the current study, we used DOX to simulate chemotherapy for visualization. Third, when purines are mentioned, purinergic signaling pathways and energy metabolism may come to mind. In this study, we focused on purine metabolism and chemoresistance without discussing other signaling pathways. These will be realized in future studies. These limitations provide room for future development and in-depth study.

## Conclusions

In conclusion, we have identified a mechanistic chain initiated by NEK6 that regulates de novo purine synthesis through C-MYC, contributing to the development of chemotherapy resistance. This provides insights into finding suitable drugs to combat chemotherapy resistance. Furthermore, our study indicates that PAE may be a potential treatment for chemoresistant ovarian cancer, a prospect that requires further confirmation.

## Supplementary information

Supplementary Figure

Supplementary Table 1

Supplementary Table 2

Supplementary Table 3

Supplementary Table 4

Supplementary Table 5

Supplementary Table 6

Supplementary Table 7

Supplementary Table 8

Original western blots

## Data Availability

All data supporting the results of this study can be found in the article and its supplementary information file. Detailed information can be obtained by contacting the corresponding author.
