# Supplementary material for: NEK6 dampens FOXO3 nuclear translocation to stabilize C-MYC and promotes subsequent de novo purine synthesis to support ovarian cancer chemoresistance
Source: Cell Death Dis. 2024 Sep 10;15(9):661. doi: 10.1038/s41419-024-07045-2 (PMC11387829; doi:10.1038/s41419-024-07045-2)
Supplement: Supplementary file 1 — Supplementary Figure [file 41419_2024_7045_MOESM1_ESM.docx]

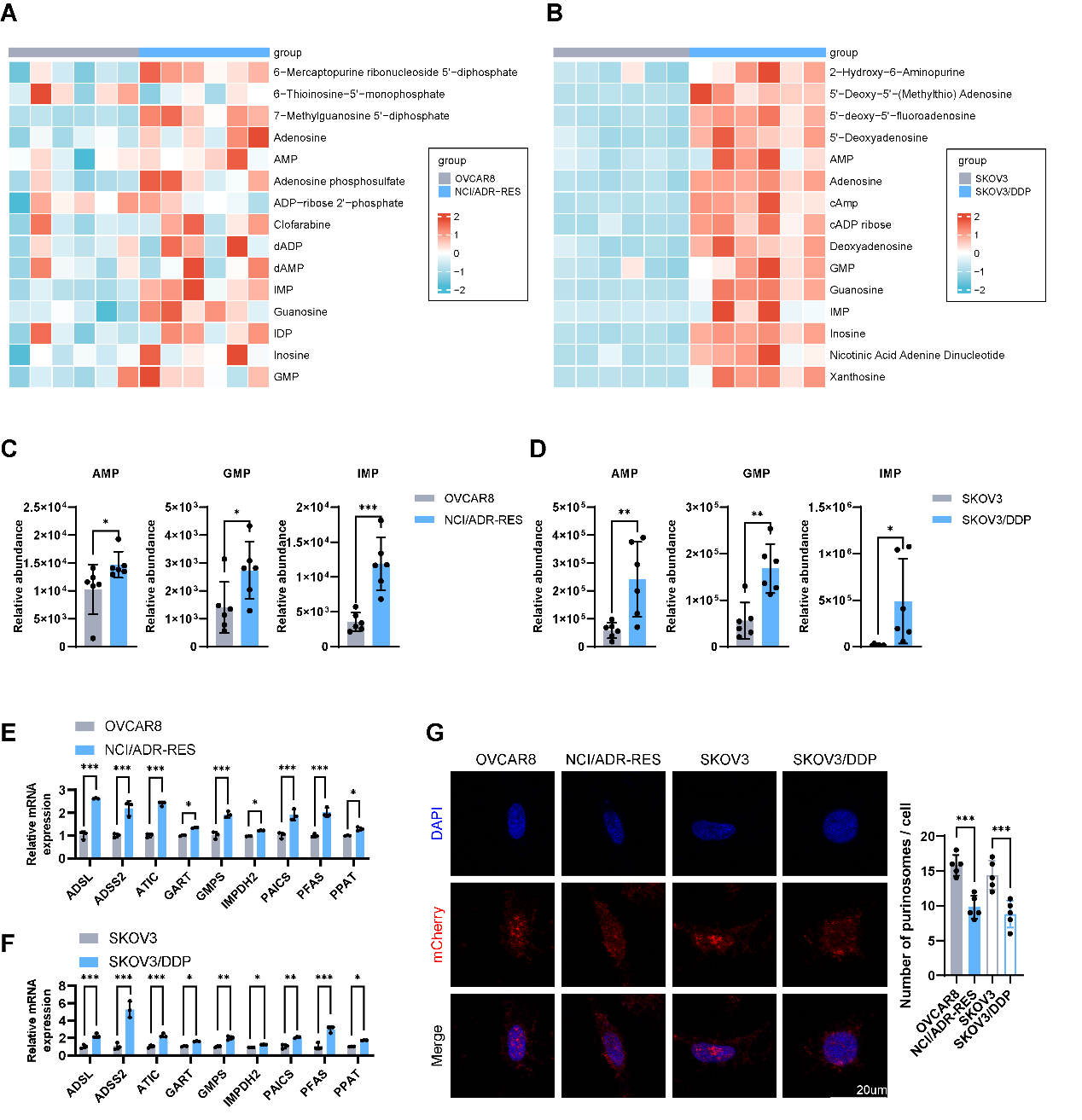


**Fig. S1 De novo purine synthesis is enhanced in chemoresistant ovarian cancer cell lines.**

(A&B) Normalized abundance heatmap of purine metabolites (A) in OVCAR8 and NCI/ADR-RES cells, and (B) in SKOV3 and SKOV3/DDP cells. N=6. (C&D) Relative abundance of AMP, GMP, and IMP (C) in OVCAR8 and NCI/ADR-RES cells, and (D) in SKOV3 and SKOV3/DDP cells. N=5. Data presented as mean ± SD. *, P < 0.05; **, P<0.01; ***, P<0.001; Student’s t-test. (E&F) Relative mRNA levels of de novo synthesis-related enzymes in OVCAR8 and NCI/ADR-RES cells, and in SKOV3 and SKOV3/DDP cells. N=3. Data presented as mean ± SD. *, P < 0.05; **, P<0.01; ***, P<0.001; ANOVA. (G) Characterization and counting of purinosomes in ovarian cancer cell lines. N=5. Data presented as mean ± SD. ***, P<0.001; ANOVA.


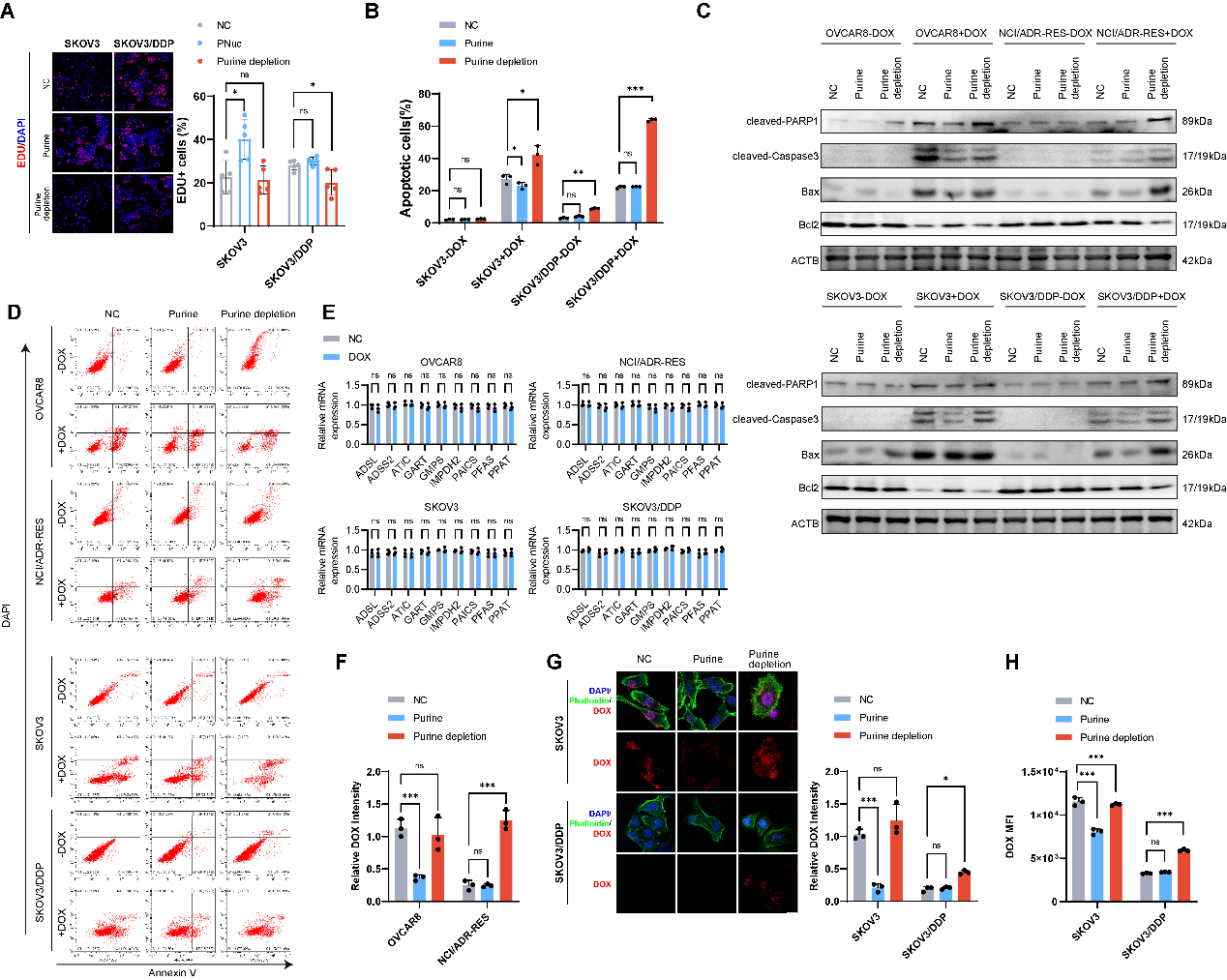


**Fig. S2 Purines facilitate chemoresistance and DNA damage repair in ovarian cancer cells.**

(A) Impact of purine supplementation and depletion on the proliferation of SKOV3 and SKOV3/DDP cells. N=5. Data presented as mean ± SD. *, P < 0.05; ns, not significant; ANOVA. (B) Influence of purine supplementation and depletion on DOX (30uM)-induced apoptosis in SKOV3 and SKOV3/DDP cells. N=3. Data presented as mean ± SD. **, P<0.01; ***, P<0.001; ns, not significant; ANOVA. (C&D) Representative (C) western blots of apoptosis-related proteins and (D) flow cytometry scatter plot in OVCAR8, NCI/ADR-RES, SKOV3, and SKOV3/DDP cells treated under different conditions. (E) Changes in mRNA levels of enzymes related to de novo purine synthesis in four cell lines after DOX treatment. N=3. Data presented as mean ± SD. ns, not significant; ANOVA. (F) Quantification of relative DOX intensity in OVCAR8 and NCI/ADR-RES cells. N=3. Data presented as mean ± SD. ***, P<0.001; ns, not significant; ANOVA. (G) Intracellular DOX uptake levels observed through confocal microscopy in SKOV3 and SKOV3/DDP cells. Red represents DOX, blue represents DAPI, and green represents phalloidin. Quantify the relative DOX intensity. N=3. Data presented as mean ± SD. *, P < 0.05; ***, P<0.001; ns, not significant; ANOVA. (H) Quantification of DOX uptake levels in each group using flow cytometry in SKOV3 and SKOV3/DDP cells. N=3. Data presented as mean ± SD. ***, P<0.001; ns, not significant; ANOVA.


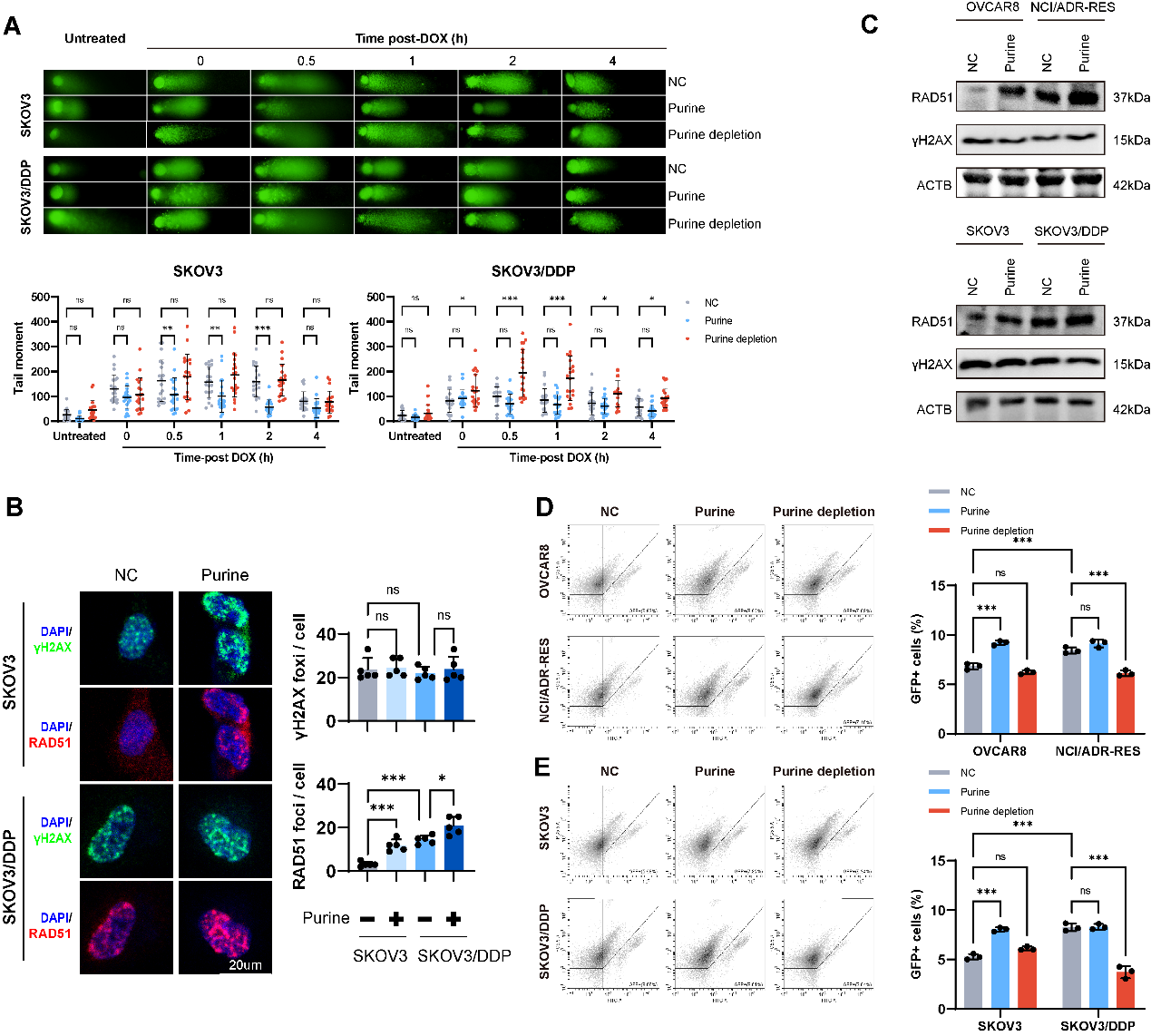


**Fig. S3 Purines affect DNA damage repair in ovarian cancer cells.**

(A) Alkaline comet assay performed on SKOV3 and SKOV3/DDP cells under different treatment conditions, with tail moment quantification. N=20. (B) Purine supplementation in SKOV3 and SKOV3/DDP cells, observing γH2AX and RAD51 foci levels. N=5. Data presented as mean ± SD. *, P < 0.05; ***, P<0.001; ns, not significant; ANOVA. (C) Representative Western blots of γH2AX and RAD51 after DOX treatment and repair for 2h in different purine conditions. (D&E) DR-GFP assay detecting homologous recombination repair levels in (D) OVCAR8, NCI/ADR-RES, (E) SKOV3 and SKOV3/DDP cells under DOX pressure. Proportion of GFP (+) cells quantified. N=3. Data presented as mean ± SD. ***, P<0.001; ns, not significant; ANOVA.


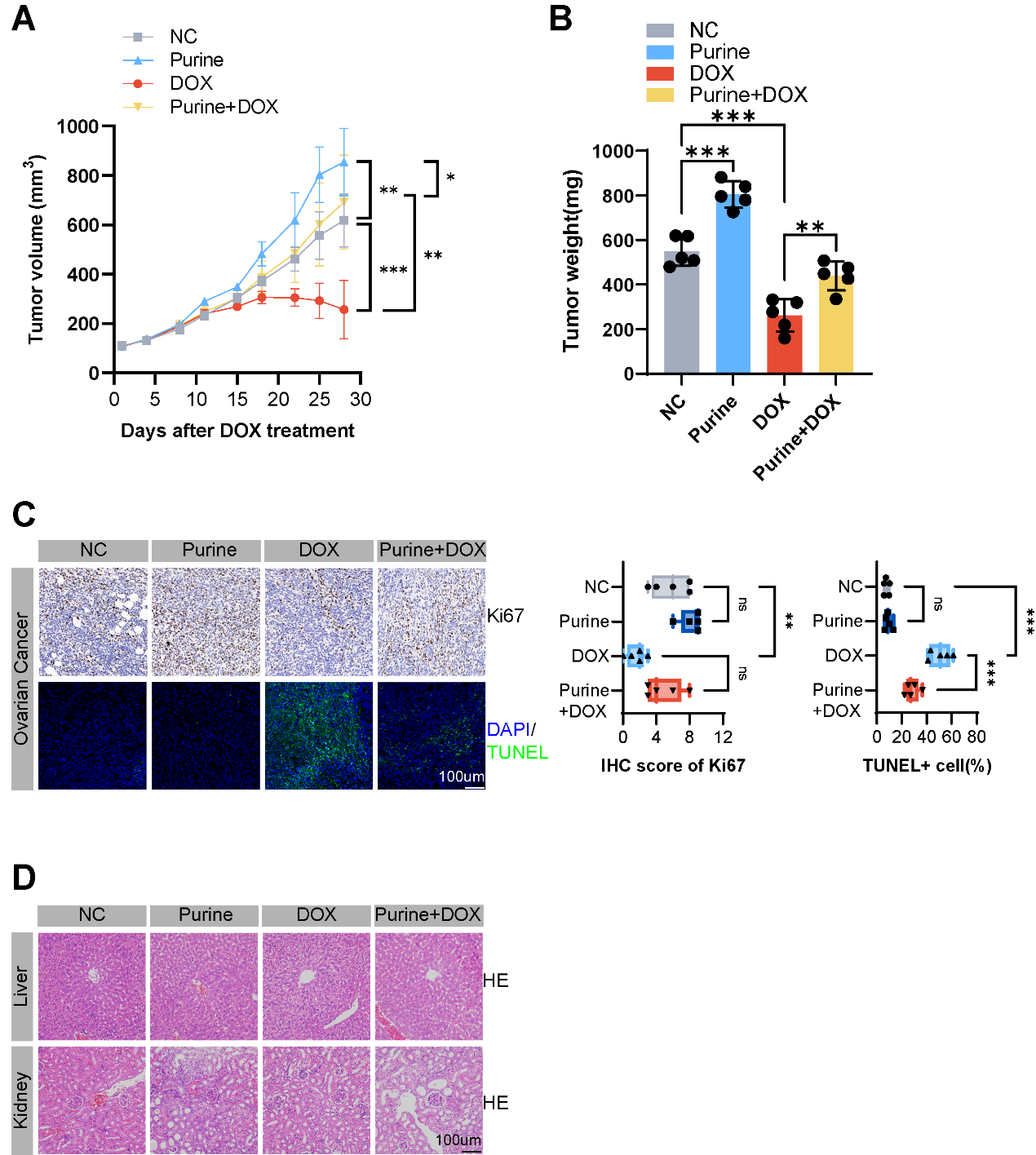


**Fig. S4 Purine supplementation promotes chemoresistance in ovarian cancer xenografts.**

(A) The xenograft model was established using the SKOV3 cell line, and the tumor volume of each group of ovarian cancer xenografts was regularly monitored and growth curves were plotted. N=5. Data presented as mean ± SD. *, P < 0.05; **, P<0.01; ***, P<0.001; ns, not significant; ANOVA. (B) Ovarian cancer xenografts were harvested at the endpoint, and the tumor tissue volumes were measured. *, P<0.01; ***, P<0.001; ns, not significant; ANOVA. (C) Representative images of Ki67 and TUNEL staining in tumor tissues. Ki67 IHC scores were determined, and the proportion of TUNEL+ cells was calculated. N=5. Data presented as mean ± SD. **, P<0.01; ***, P<0.001; ns, not significant; ANOVA. (D) Representative HE staining images of mouse liver and kidney tissues in each group.


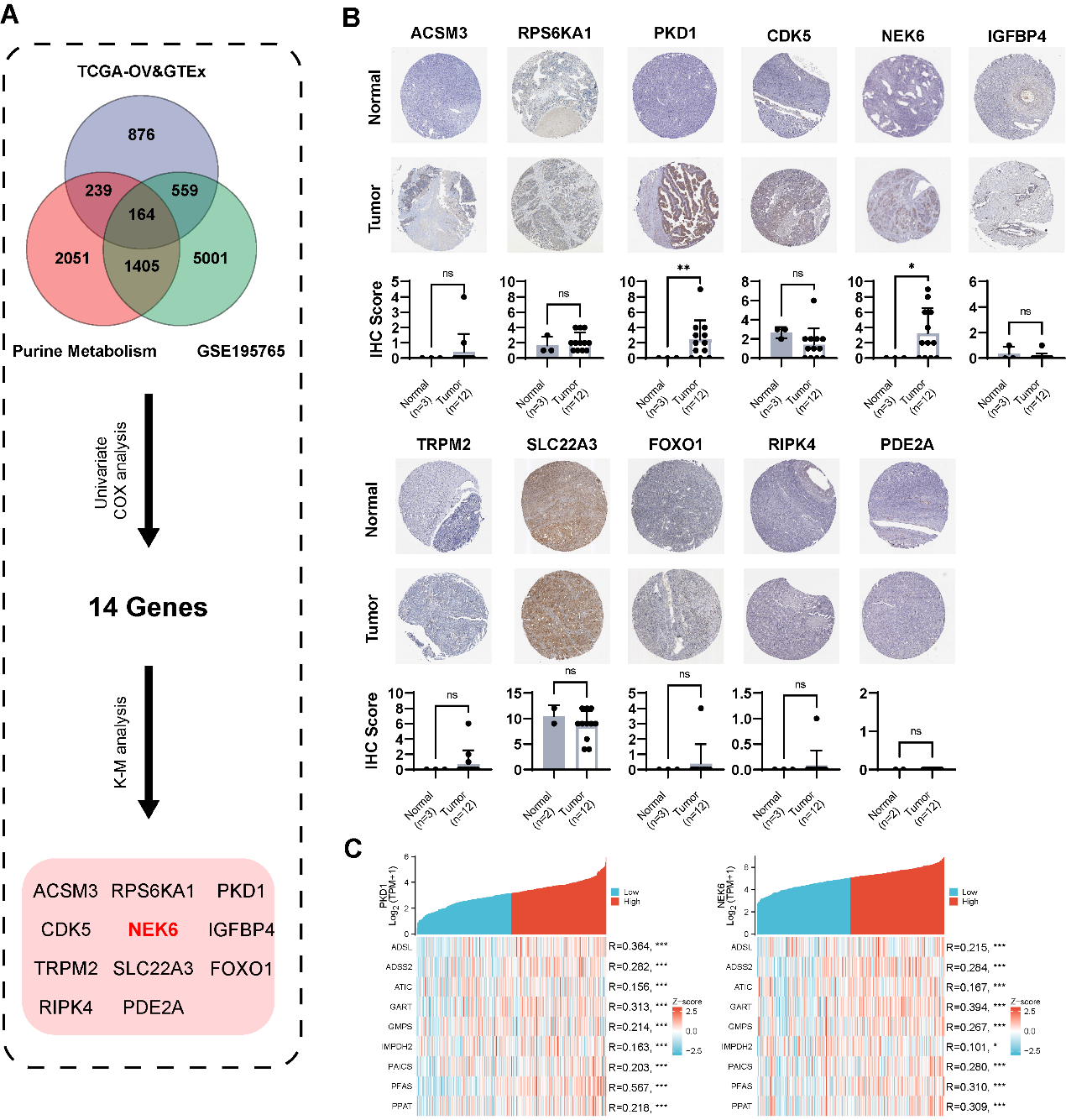


**Fig. S5 Comprehensive bioinformatics analysis identifies NEK6 as a key molecule mediating chemoresistance and de novo purine synthesis pathways in ovarian cancer.**

(A) Process of obtaining candidate genes. Three datasets were acquired: (1) Differential genes in TCGA-OV and GTEx; (2) Purine metabolism-related genes from Genecards; (3) Differential genes in SKOV3 and SKOV3/DDP cells (uploaded to GEO with ID: GSE195765). Venn diagrams depict the intersection of the three datasets. Based on univariate COX analysis and K-M analysis, 11 candidate genes were identified. (B) Representative immunohistochemical images of ovarian tissues and ovarian tumor tissues were obtained from the HPA database and scored. Data presented as mean ± SD. *, P < 0.05; **, P<0.01; ns, not significant; Student’s t-test. (C) Visualization and correlation of mRNA expression of PKD1 and NEK6 with enzymes related to de novo purine synthesis in ovarian cancer samples from TCGA-OV. TPM, transcripts per million.


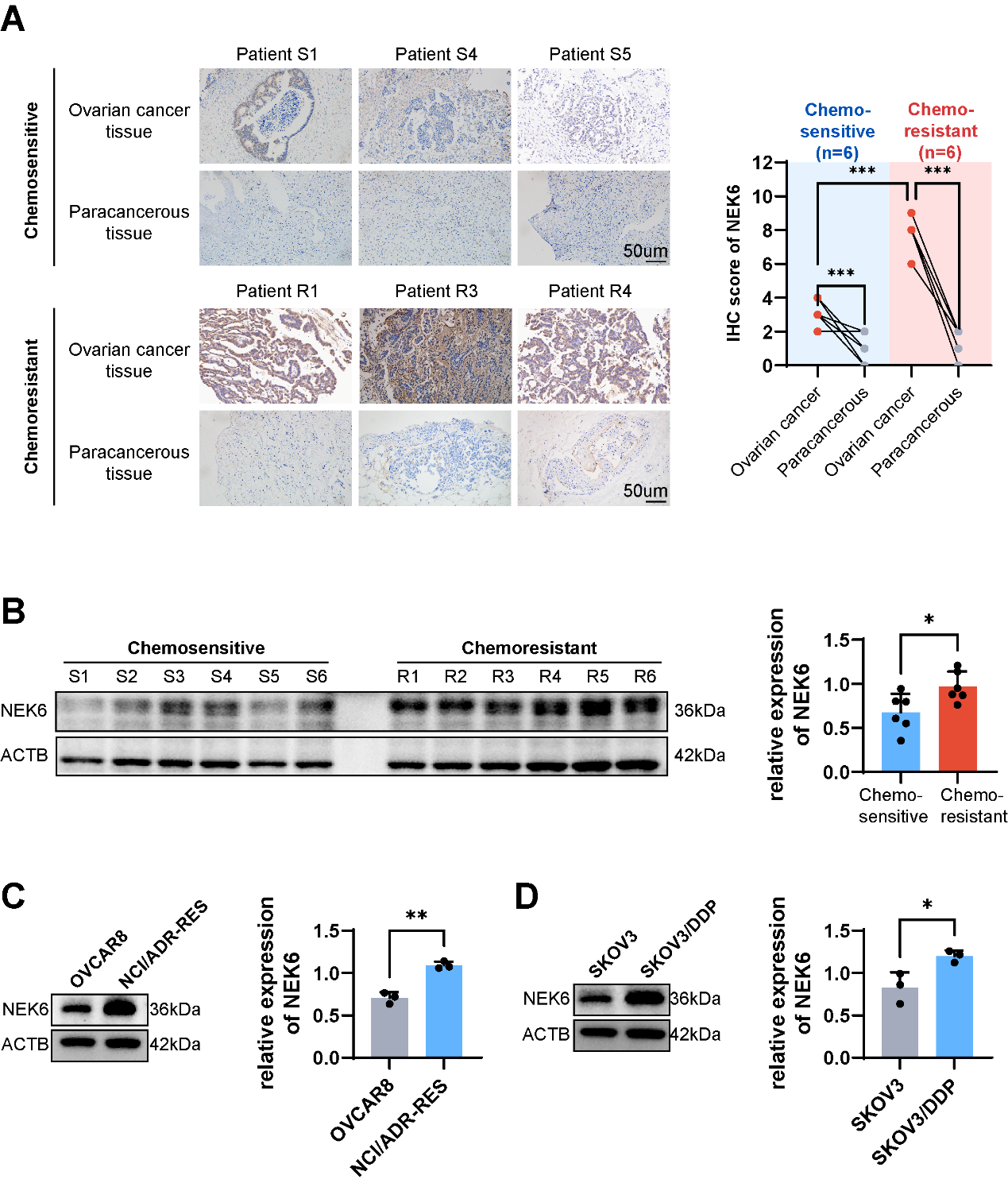


**Fig. S6 NEK6 differs in chemosensitive and chemoresistant ovarian cancer.**

(A) Representative immunohistochemical images and scores of NEK6 in chemosensitive and chemoresistant ovarian cancer tissues and their paired paracancerous tissue. N=6. Data presented as mean ± SD. *, P < 0.05; Student’s t-test. (B) Representative Western blot images and protein level quantification of NEK6 in chemosensitive and chemoresistant ovarian cancer tissues. N=6. Data presented as mean ± SD. *, P < 0.05; Student’s t-test. (C) Representative Western blot images and protein level quantification of NEK6 in OVCAR8 and NCI/ADR-RES cells. N=3. Data presented as mean ± SD. **, P<0.01; Student’s t-test. (D) Representative Western blot images and protein level quantification of NEK6 in SKOV3 and SKOV3/DDP cells. N=3. Data presented as mean ± SD. *, P < 0.05; Student’s t-test.


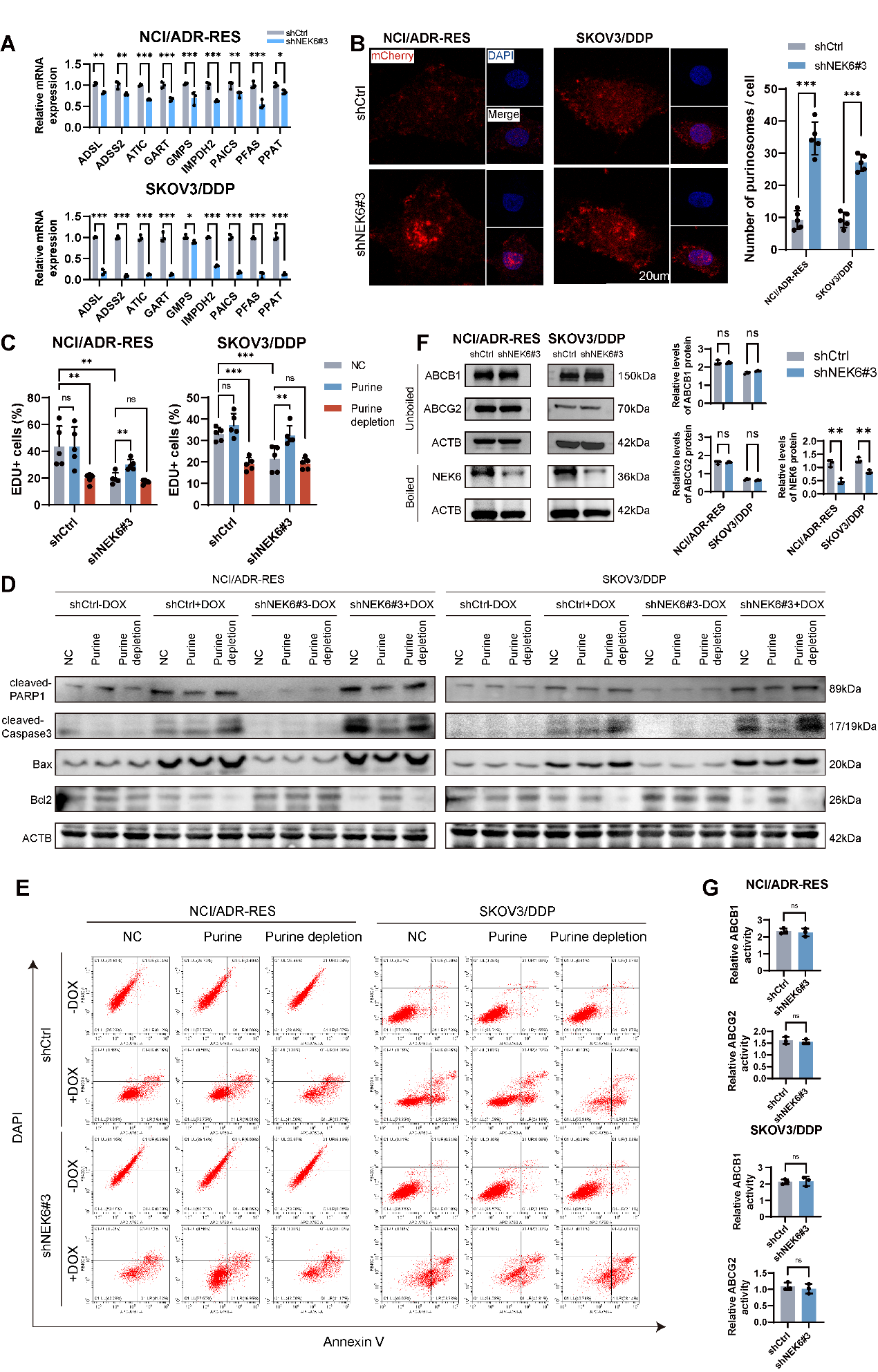


**Fig. S7 NEK6 contributes to chemoresistance in ovarian cancer.**

(A) Impact of NEK6 knockdown on the mRNA levels of enzymes related to de novo purine synthesis in NCI/ADR-RES and SKOV3/DDP cells. N=3. Data presented as mean ± SD. *, P < 0.05; **, P<0.01; ***, P<0.001; ANOVA. (B) Changes in purinosome levels after NEK6 knockdown in NCI/ADR-RES and SKOV3/DDP cells. N=5. Data presented as mean ± SD. ***, P<0.001; ANOVA. (C) Effect of NEK6 knockdown and concomitant purine supplementation or depletion on the proliferation of NCI/ADR-RES and SKOV3/DDP cells. N=5. Data presented as mean ± SD. **, P<0.01; ***, P<0.001; ns, not significant; ANOVA. (D&E) Representative (D) western blots of apoptosis-related proteins and (E) flow cytometry scatter plots under NEK6 knockdown, purine, and DOX interventions. (F) Western blots showing the impact of NEK6 knockdown on the protein levels of drug transporters ABCB1 and ABCG2. N=3. Data presented as mean ± SD. **, P<0.01; ns, not significant; ANOVA. (G) Impact of NEK6 knockdown on the protein activity of ABCB1 and ABCG2. N=3. Data presented as mean ± SD. Ns, not significant; ANOVA.


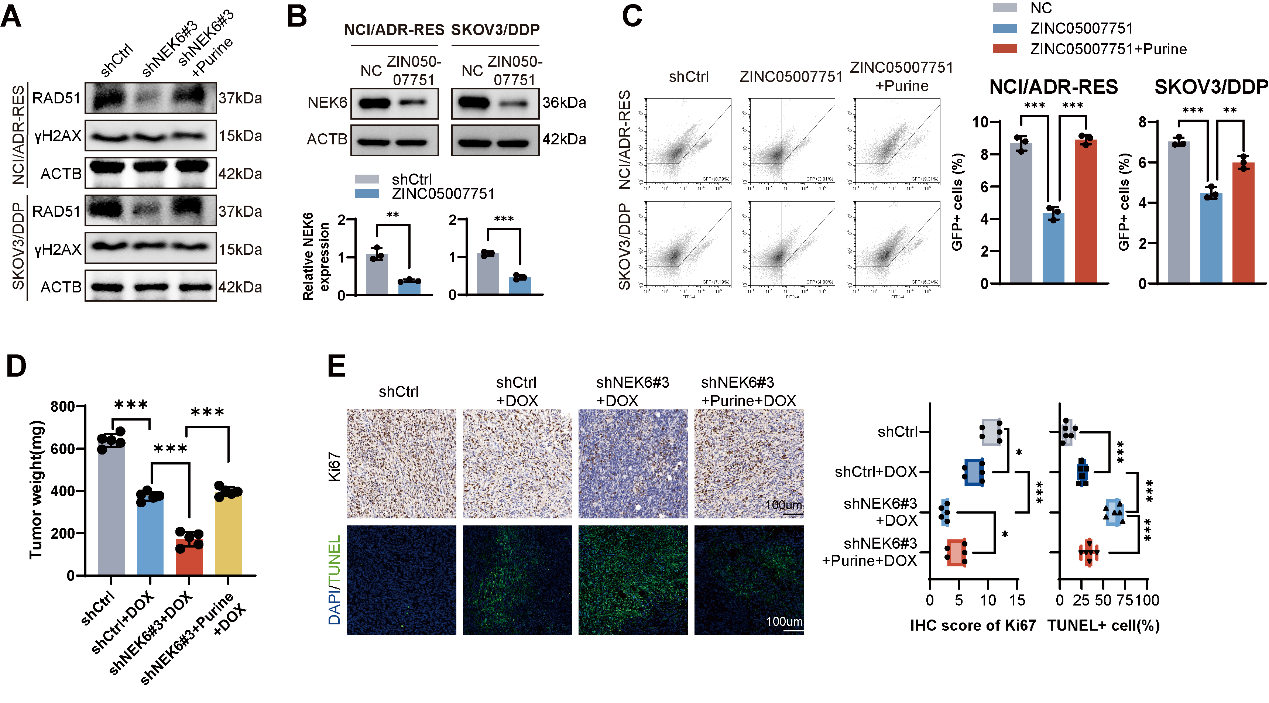


**Fig. S8 NEK6 contributes to chemoresistance and DNA damage repair in ovarian cancer.**

(A) Representative western blot images of RAD51 and γH2AX in NCI/ADR-RES and SKOV3/DDP after undergoing DOX-mediated damage and repairing for 2h under NEK6 and purine intervention. (B) Western blot showing inhibition of NEK6 protein levels using ZIN05007751 (10uM). N=3. Data presented as mean ± SD. **, P<0.01; ***, P<0.001; Student’s t-test. (C) DR-GFP experiment demonstrating the impact of NEK6 knockdown and concomitant purine supplementation on homologous recombination repair under DOX stress. **, P<0.01; ***, P<0.001; ANOVA. (D) Measurement of the volume of ovarian cancer xenografts derived from SKOV3/DDP cells at the endpoint. N=5. Data presented as mean ± SD. ***, P<0.001; ANOVA. (E) Representative Ki67 and TUNEL staining images of ovarian cancer xenografts. Calculation of IHC score for Ki67 and proportion of TUNEL+ cells. N=5. Data presented as mean ± SD. *, P < 0.05; ***, P<0.001; ANOVA.


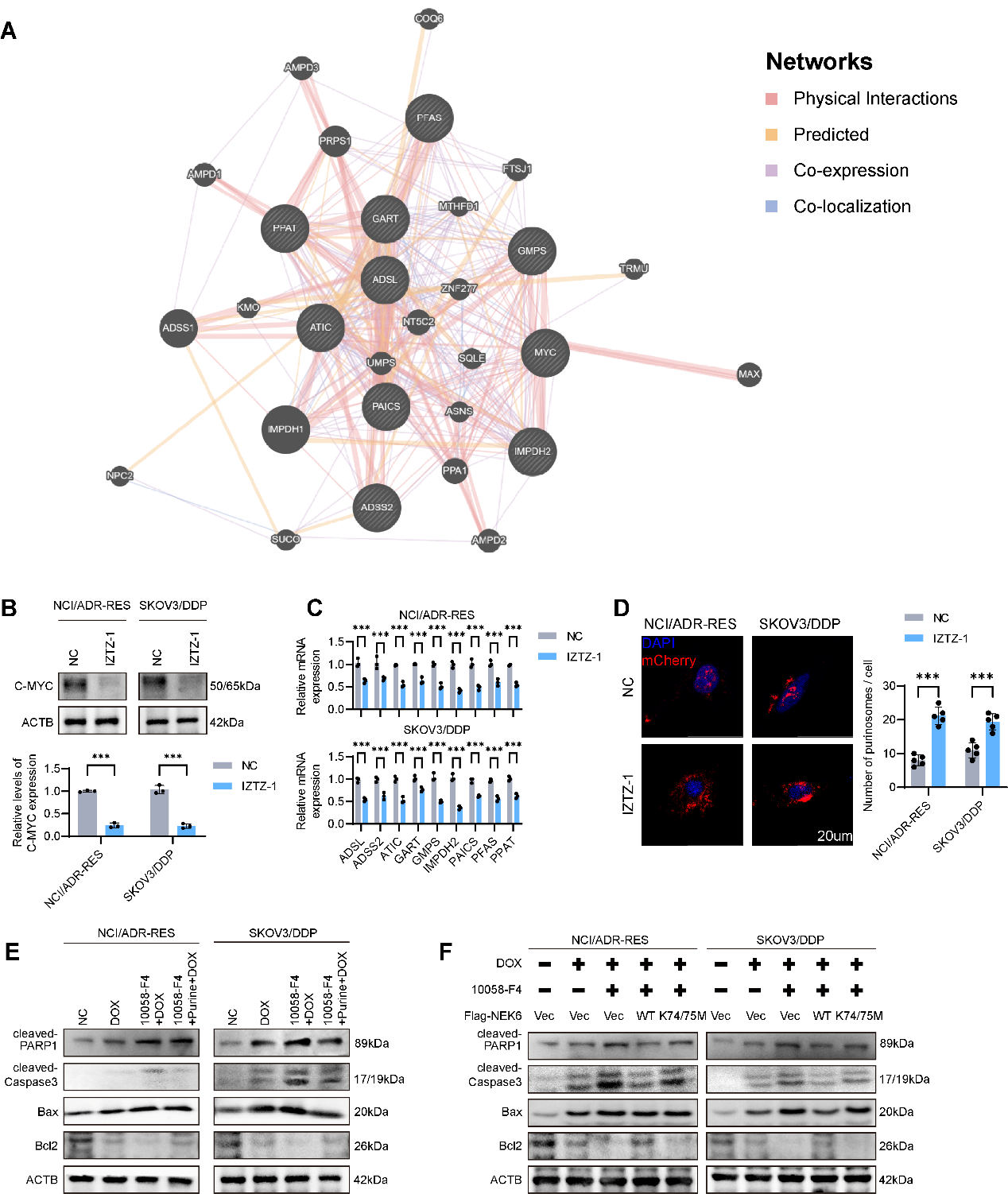


**Fig. S9 C-MYC is essential for de novo purine synthesis.**

(A) The GeneMANIA analysis revealed the association of C-MYC with de novo purine synthesis-related enzymes. The network diagrams illustrate diverse connections, encompassing physical interactions, predicted roles, co-expression, and co-localization, providing a comprehensive overview of their relationships. (B) Changes in C-MYC protein levels in NCI/ADR-RES and SKOV3/DDP cells after IZTZ-1 (5µM) treatment. N=3. Data presented as mean ± SD. ***, P < 0.001; ANOVA. (C) Changes in mRNA levels of de novo purine synthesis-related enzymes after IZTZ-1 treatment. N=3. Data presented as mean ± SD. ***, P<0.001; ANOVA. (D) Changes in the number of purinosomes after IZTZ-1 intervention. N=5. Data presented as mean ± SD. ***, P<0.001; ANOVA. (E) Representative western blot images of apoptosis-related proteins under 10058-F4, DOX, and purine intervention in NCI/ADR-RES and SKOV3/DDP. (F) Representative western blot images of apoptosis-related proteins under 10058-F4, DOX, and NEK6 intervention in NCI/ADR-RES and SKOV3/DDP.


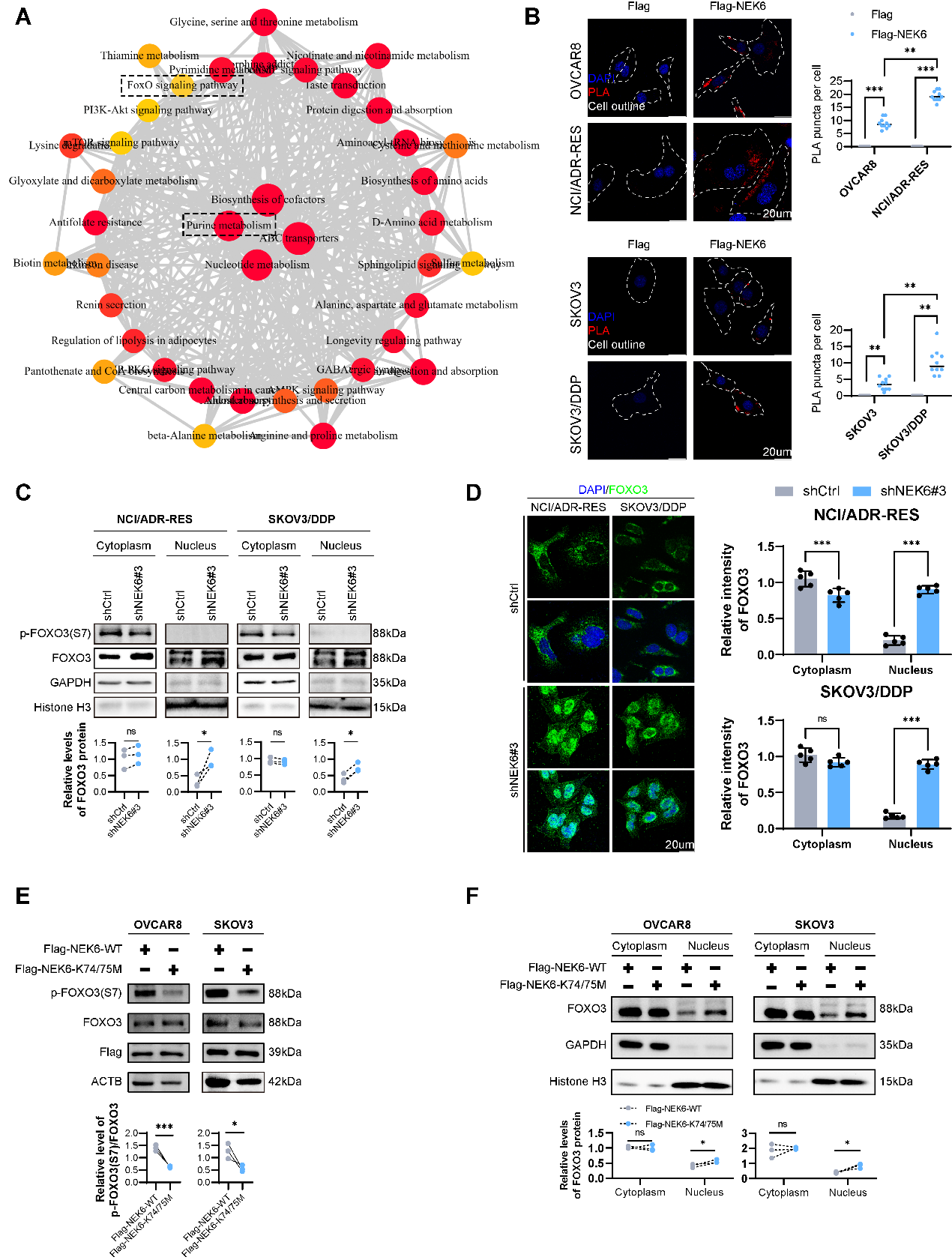


**Fig. S10 NEK6 kinase phosphorylates FOXO3 S7, and inhibiting FOXO3 nuclear translocation.**

(A) Enrichment network maps based on metabolites highlight the differential enrichment of metabolites related to the FOXO family pathway in NCI/ADR-RES cells between shCtrl and shNEK6#3 groups. (B) PLA assay showing direct interaction between NEK6 and FOXO3 in ovarian cancer cells. N=10. Data presented as mean ± SD. **, P<0.01; ***, P<0.001; ANOVA. (C) Western blot detecting the impact of NEK6 knockdown on FOXO3 nuclear translocation. N=3. Data presented as mean ± SD. *, P<0.05; **, P<0.01; ns, not significant; Student’s t-test. (D) Quantification of FOXO3 immunofluorescent subcellular localization and intensity in NCI/ADR-RES and SKOV3/DDP cells after NEK6 knockdown. N=5. Data presented as mean ± SD. ***, P<0.001; ns, not significant; ANOVA. (E) Western blot showing the impact of introducing Flag-NEK6-WT or Flag-NEK6-K74/75M on FOXO3 S7 phosphorylation level. N=3. Data presented as mean ± SD. *, P < 0.05; ***, P<0.001; Student’s t-test. (F) Impact of introducing Flag-NEK6-WT or Flag-NEK6-K74/75M on FOXO3 protein nuclear translocation. *, P < 0.05; ns, not significant; ANOVA.


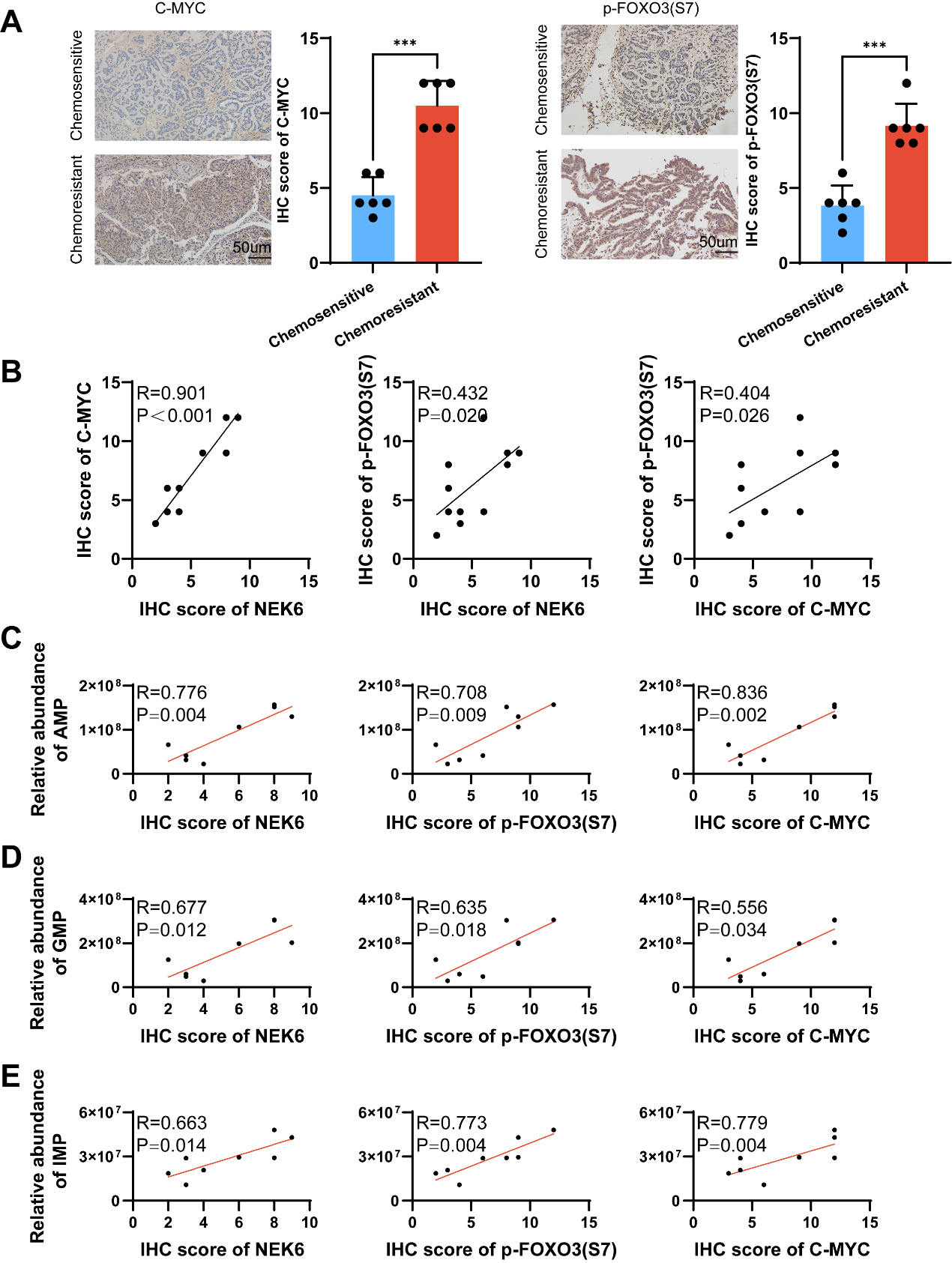


**Fig. S11 Correlations between NEK6, C-MYC, FOXO3, and purine intermediates in ovarian cancer tissues.**

(A) Images show C-MYC and p-FOXO3(S7) IHC staining in chemo-sensitive and chemo-resistant ovarian cancer tissues, and IHC scores were calculated. N=6. Data presented as mean ± SD. ***, P<0.001; Student’s t-test. (B) Pearson correlation analysis of IHC scores of NEK6, p-FOXO3(S7) and C-MYC in ovarian cancer tissues. N=12. (C) Pearson correlation analysis of IHC scores of NEK6, p-FOXO3(S7), C-MYC and relative abundance of AMP in ovarian cancer tissues. N=8. (D) Pearson correlation analysis of IHC scores of NEK6, p-FOXO3(S7), C-MYC and relative abundance of GMP in ovarian cancer tissues. N=8. (E) Pearson correlation analysis of IHC scores of NEK6, p-FOXO3(S7), C-MYC and relative abundance of IMP in ovarian cancer tissues. N=8.


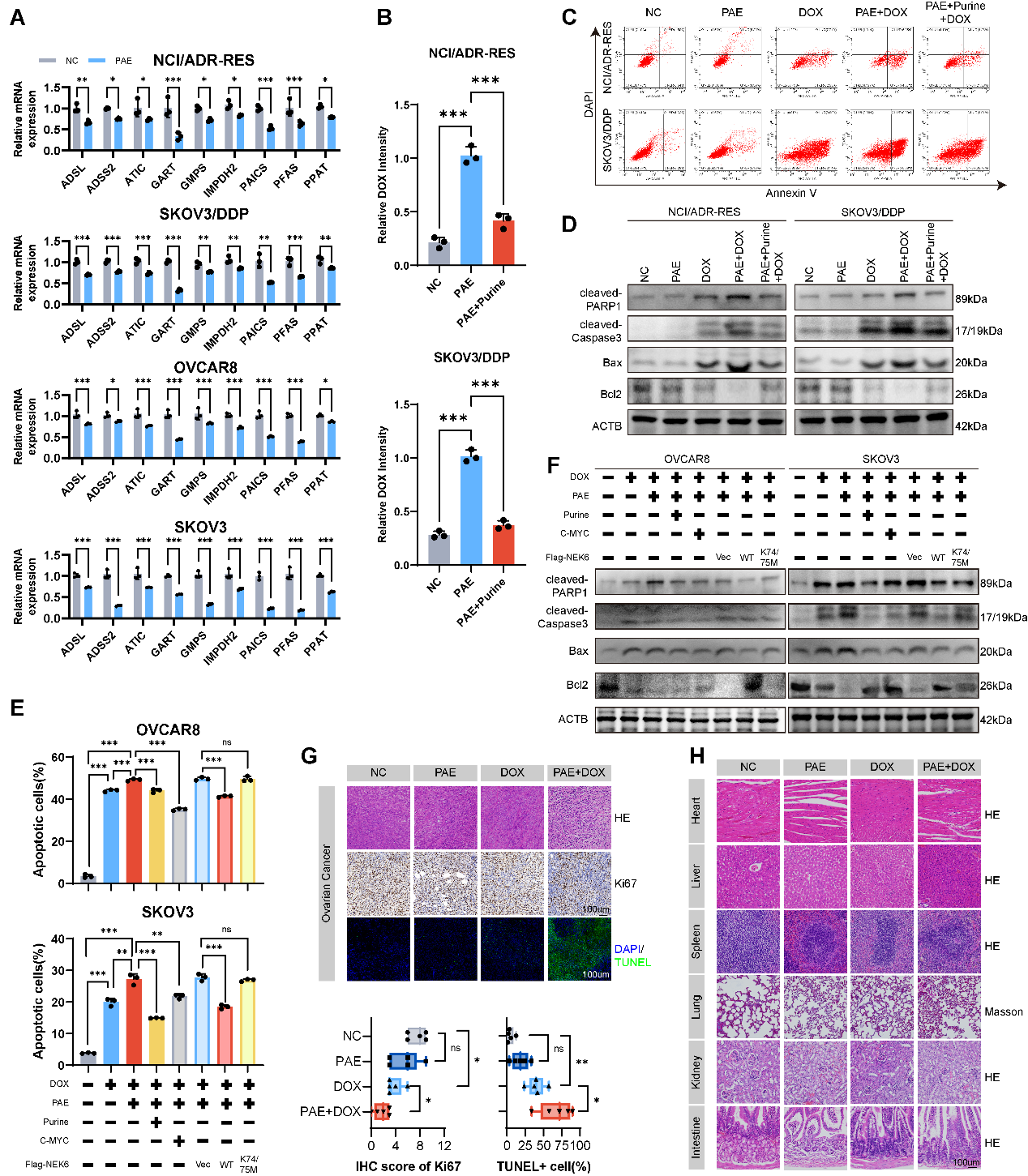


**Fig. S12 PAE suppresses de novo purine synthesis and chemoresistance in ovarian cancer.**

(A) Impact of PAE on the mRNA levels of enzymes related to de novo purine synthesis in NCI/ADR-RES, SKOV3/DDP, OVCAR8 and SKOV3 cells. N=3. Data presented as mean ± SD. *, P < 0.05; **, P<0.01; ***, P<0.001; ANOVA. (B) Quantifying the relative intensity of DOX in NCI/ADR-RES and SKOV3/DDP cells. N=3. Data presented as mean ± SD. ***, P<0.001; ANOVA. (C&D) Representative (C) flow cytometry scatter plots and (D) western blot images of apoptosis-related proteins under PAE, DOX, and purine treatments in NCI/ADR-RES and SKOV3/DDP. (E) Detection of apoptosis levels in OVCAR8 and SKOV3 under PAE, C-MYC and NEK6 intervention. N=3. Data presented as mean ± SD. **, P<0.01; ***, P<0.001; ns, not significant; ANOVA. (F) Representative western blot images of apoptosis-related proteins under each intervention treatment in OVCAR8 and SKOV3 cells. (G) Representative HE images, Ki67 staining images, and TUNEL staining images of ovarian cancer xenografts in each group. Evaluation of IHC score for Ki67 and calculation of TUNEL+ cell percentage. N=5. Data presented as mean ± SD.*, P < 0.05; **, P<0.01; ns, not significant; ANOVA. (H) Representative HE staining images of mouse heart, liver, spleen, kidney, and small intestine, and Masson staining images of the lung in each treatment group.
