# Supplementary material for: NEK6 dampens FOXO3 nuclear translocation to stabilize C-MYC and promotes subsequent de novo purine synthesis to support ovarian cancer chemoresistance
Source: Cell Death Dis. 2024 Sep 10;15(9):661. doi: 10.1038/s41419-024-07045-2 (PMC11387829; doi:10.1038/s41419-024-07045-2)
Supplement: Supplementary file 2 — Supplementary Table 1 [file 41419_2024_7045_MOESM2_ESM.pdf]

Supplementary Table 1. Primer sequences.

Manufacturer	Gene	Species	Direction	Sequences
Sangon Biotech (China)	FBXW7	Human	FORWARD	CTGGAACCCAAAGACCTGCTACAAG
Sangon Biotech (China)	FBXW7	Human	REVERSE	TGCCTGTGACTGCTGACCAAAC
Sangon Biotech (China)	GART	Human	FORWARD	GCCATTCACTGTTACCTGTCCTACG
Sangon Biotech (China)	GART	Human	REVERSE	CTGCTCTGTCTGCTCCTTTGATACC
Sangon Biotech (China)	C-MYC	Human	FORWARD	AGTCTGGATCACCTTCTGCTGGAG
Sangon Biotech (China)	C-MYC	Human	REVERSE	GCTTGGACGGACAGGATGTATGC
Sangon Biotech (China)	ADSS2	Human	FORWARD	CCGCTGCCAGGGAGGAAATAATG
Sangon Biotech (China)	ADSS2	Human	REVERSE	AGTCAGAAACAAGGTCGCACATCC
Sangon Biotech (China)	GMPS	Human	FORWARD	TTTCCAGGCATCCATTTCCAGGTC
Sangon Biotech (China)	GMPS	Human	REVERSE	TGGCAGCAAGAAGGCATTCAGTG
Sangon Biotech (China)	IMPDH2	Human	FORWARD	TGGACCTGACTTCTGCTCTGACC
Sangon Biotech (China)	IMPDH2	Human	REVERSE	GCCTGTGTCTGTGATTGGGATACC
Sangon Biotech (China)	PFAS	Human	FORWARD	GAGAGGAGGGCAGTAATGGAGACC
Sangon Biotech (China)	PFAS	Human	REVERSE	CAGGTTGTGGCGTAGCAGAAGG
Sangon Biotech (China)	PPAT	Human	FORWARD	CAGAAACAGAAGGATGGGTGGTGTC
Sangon Biotech (China)	PPAT	Human	REVERSE	GAGCAGCAGGCGTAGCAGATTC
Sangon Biotech (China)	PAICS	Human	FORWARD	CCATCAGGAGATCGAAGCCAACAG
Sangon Biotech (China)	PAICS	Human	REVERSE	AACCATTACTTCTGCCTGCCACTG
Sangon Biotech (China)	ADSL	Human	FORWARD	GATGATAGTGCCAACCGACGGATC
Sangon Biotech (China)	ADSL	Human	REVERSE	CAACTGGGAGTGAATGGGACTGAAG
Sangon Biotech (China)	ATIC	Human	FORWARD	GGTTATCGGCATTGGAGCAGGAC
Sangon Biotech (China)	ATIC	Human	REVERSE	CGGAAAGGGAAGAAGGCATCAGAG
Sangon Biotech (China)	NEK6	Human	FORWARD	ACATTGTGCTGGAGTTGGCTGAC
Sangon Biotech (China)	NEK6	Human	REVERSE	GGACCAGATGTCGGACTTGAAGTTG
Sangon Biotech (China)	ACTB	Human	FORWARD	GGCACCCAGCACAATGAAG
Sangon Biotech (China)	ACTB	Human	REVERSE	CCGATCCACACGGAGTACTTG
Sangon Biotech (China)	S1	Human	FORWARD	GGGCTGCAGAACTCTGATGT
Sangon Biotech (China)	S1	Human	REVERSE	TTACGTGACCAATCCCCTGC
Sangon Biotech (China)	S2	Human	FORWARD	TGCTCCTACCTTACCCCCTC
Sangon Biotech (China)	S2	Human	REVERSE	GCTTTCGAGAGCTTCTGGTC
Sangon Biotech (China)	S3	Human	FORWARD	GGATGGCTCCCTTCCAATCC
Sangon Biotech (China)	S3	Human	REVERSE	CCTACAGCCTGATCTGCACC
