# Supplementary material for: NEK6 dampens FOXO3 nuclear translocation to stabilize C-MYC and promotes subsequent de novo purine synthesis to support ovarian cancer chemoresistance
Source: Cell Death Dis. 2024 Sep 10;15(9):661. doi: 10.1038/s41419-024-07045-2 (PMC11387829; doi:10.1038/s41419-024-07045-2)
Supplement: Supplementary file 3 — Supplementary Table 2 [file 41419_2024_7045_MOESM3_ESM.pdf]

Supplementary Table 2. Antibodies.

Manufacturer	Target	Cat No.
Proteintech	ACTB	81115-1-RR
Proteintech	C-MYC	10828-1-AP
Proteintech	GST Tag	10000-0-AP
Proteintech	FBXW7	28424-1-AP
Proteintech	Histone H3	68345-1-Ig
Proteintech	GAPDH	10494-1-AP
Proteintech	HRP-conjugated Affinipure Goat Anti-Mouse IgG(H+L)	SA00001-1
Proteintech	HRP-conjugated Affinipure Goat Anti-Rabbit IgG(H+L)	SA00001-2
Proteintech	ABCB1	22336-1-AP
Proteintech	ABCG2	27286-1-AP
Proteintech	BAX	50599-2-Ig
Proteintech	Cleaved-Caspase3	19677-1-AP
Proteintech	Multi-rAb CoraLite® Plus 488-Goat Anti-Rabbit Recombinant Secondary Antibody (H+L)	RGAR002
Proteintech	Multi-rAb CoraLite® Plus 555-Goat Anti-Rabbit Recombinant Secondary Antibody (H+L)	RGAR003
Proteintech	Multi-rAb CoraLite® Plus 488-Goat Anti-Mouse Recombinant Secondary Antibody (H+L)	RGAM002
ABclonal	NEK6	A3536
ABclonal	Ubiquitin	A0162
ABclonal	pan Phospho-Serine/Threonine	AP1067
ABclonal	IgG	AC005
ABclonal	HRP-conjugated Goat Anti-Mouse IgG Light Chain	AS062
ABclonal	HRP-conjugated Goat Anti-Rabbit IgG Heavy Chain	AS063
Cell Signaling Technology	Flag Tag	#14793
Cell Signaling Technology	His Tag	#2366
Cell Signaling Technology	FOXO3	#99199
Cell Signaling Technology	Phospho-FoxO3a (Ser7)	#14724
Cell Signaling Technology	γH2AX	#2577
Cell Signaling Technology	Ki67	#9449
Cell Signaling Technology	Cleaved-PARP	#5625
Abcam	RAD51	ab133534
Abcam	PFAS	ab251740
Abcam	NEK6	ab133494
Abmart	BCL2	T40056
