# Supplementary material for: NEK6 dampens FOXO3 nuclear translocation to stabilize C-MYC and promotes subsequent de novo purine synthesis to support ovarian cancer chemoresistance
Source: Cell Death Dis. 2024 Sep 10;15(9):661. doi: 10.1038/s41419-024-07045-2 (PMC11387829; doi:10.1038/s41419-024-07045-2)
Supplement: Supplementary file 4 — Supplementary Table 3 [file 41419_2024_7045_MOESM4_ESM.pdf]

Supplementary Table 3. Purine metabolism-related genes

Gene Symbol	Description	Category	Gifts	GC Id	Relevance score
PNP	Purine Nucleoside Phosphorylase	Protein Coding	48	GC14P020468	100.8780441
HPRT1	Hypoxanthine Phosphoribosyltransferase 1	Protein Coding	49	GC0XP134460	33.57402802
APRT	Adenine Phosphoribosyltransferase	Protein Coding	48	GC16M088810	33.50376892
ADA	Adenosine Deaminase	Protein Coding	52	GC20M044620	30.68210602
XDH	Xanthine Dehydrogenase	Protein Coding	47	GC02M031334	30.1312809
ADSL	Adenylosuccinate Lyase	Protein Coding	48	GC22P040346	28.6300869
ATIC	5-Aminoimidazole-4-Carboxamide Ribonucleotide Formyltransferase/IMP Cyclohydrolase	Protein Coding	47	GC02P215311	26.7547226
PRPS1	Phosphoribosyl Pyrophosphate Synthetase 1	Protein Coding	47	GC0XP107628	23.95949745
PURA	Purine Rich Element Binding Protein A	Protein Coding	44	GC05P140076	20.08096504
PURB	Purine Rich Element Binding Protein B	Protein Coding	37	GC07M044879	19.53914833
PURG	Purine Rich Element Binding Protein G	Protein Coding	34	GC08M030995	18.13925362
PPAT	Phosphoribosyl Pyrophosphate Amidotransferase	Protein Coding	44	GC04M056393	17.75395393
GART	Phosphoribosylglycinamide Formyltransferase, Phosphoribosylglycinamide Synthetase, Phosphoribosylaminoimidazole Synthetase	Protein Coding	43	GC21M033503	16.54654312

NT5C2	5'-Nucleotidase, Cytosolic II	Protein Coding	47	GC10M103088	15.25541687
ITPA	Inosine Triphosphatase	Protein Coding	47	GC20P003284	14.68950939
SLC28A2	Solute Carrier Family 28 Member 2	Protein Coding	43	GC15P045252	14.47646427
DCK	Deoxycytidine Kinase	Protein Coding	45	GC04P070992	14.40042305
PAICS	Phosphoribosylaminoimidazole Carboxylase And Phosphoribosylaminoimidazolesuccinocarboxamide Synthase	Protein Coding	43	GC04P056410	13.50473118
GDA	Guanine Deaminase	Protein Coding	44	GC09P072114	12.28308487
DPYD	Dihydropyrimidine Dehydrogenase	Protein Coding	53	GC01M097015	12.2287817
MTAP	Methylthioadenosine Phosphorylase	Protein Coding	48	GC09P021792	12.16714954
ADK	Adenosine Kinase	Protein Coding	52	GC10P074152	11.80184555
ADSS2	Adenylosuccinate Synthase 2	Protein Coding	36	GC01M244410	11.70924664
PFAS	Phosphoribosylformylglycinamidine Synthase	Protein Coding	44	GC17P008247	10.7866478
DGUOK	Deoxyguanosine Kinase	Protein Coding	45	GC02P073926	10.60031128
AHCY	Adenosylhomocysteinase	Protein Coding	51	GC20M034341	10.18976974
CDK2	Cyclin Dependent Kinase 2	Protein Coding	52	GC12P055966	10.08246803
SLC28A3	Solute Carrier Family 28 Member 3	Protein Coding	39	GC09M086096	9.748725891
AMPD3	Adenosine Monophosphate Deaminase 3	Protein Coding	46	GC11P010309	9.711313248
TPMT	Thiopurine S-Methyltransferase	Protein Coding	48	GC06M018128	9.674671173
LACC1	Laccase Domain Containing 1	Protein Coding	35	GC13P043879	9.489016533
AK2	Adenylate Kinase 2	Protein Coding	49	GC01M033007	9.408278465
NT5E	5'-Nucleotidase Ecto	Protein Coding	52	GC06P085449	9.293776512

GMPS	Guanine Monophosphate Synthase	Protein Coding	45	GC03P155870	9.112840652
ADSS1	Adenylosuccinate Synthase 1	Protein Coding	36	GC14P106068	8.629751205
AOX1	Aldehyde Oxidase 1	Protein Coding	45	GC02P200585	8.326910973
AMPD1	Adenosine Monophosphate Deaminase 1	Protein Coding	46	GC01M114673	8.29387188
NUDT1	Nudix Hydrolase 1	Protein Coding	44	GC07P002242	8.269208908
PNPP1	PNP Pseudogene 1	Pseudogene	4	GC02M076259	8.247542381
SLC29A2	Solute Carrier Family 29 Member 2	Protein Coding	45	GC11M069464	8.165662766
AMPD2	Adenosine Monophosphate Deaminase 2	Protein Coding	47	GC01P109616	8.058411598
PRPS1L1	Phosphoribosyl Pyrophosphate Synthetase 1 Like 1	Protein Coding	37	GC07M018026	8.052090645
DHFR	Dihydrofolate Reductase	Protein Coding	50	GC05M080626	7.958518505
ZAP70	Zeta Chain Of T Cell Receptor Associated Protein Kinase 70	Protein Coding	52	GC02P097696	7.864109516
SLC28A1	Solute Carrier Family 28 Member 1	Protein Coding	43	GC15P084884	7.660475254
MOCOS	Molybdenum Cofactor Sulfurase	Protein Coding	43	GC18P036187	7.652844429
IMPDH2	Inosine Monophosphate Dehydrogenase 2	Protein Coding	47	GC03M049439	7.355089188
OGG1	8-Oxoguanine DNA Glycosylase	Protein Coding	47	GC03P009751	7.348906994
CDK1	Cyclin Dependent Kinase 1	Protein Coding	47	GC10P060772	7.288618088
MPG	N-Methylpurine DNA Glycosylase	Protein Coding	42	GC16P005486	7.113366604
PDE5A	Phosphodiesterase 5A	Protein Coding	45	GC04M119494	7.022368908
ADORA2A	Adenosine A2a Receptor	Protein Coding	46	GC22P024417	6.957066536

UMPS	Uridine Monophosphate Synthetase	Protein Coding	47	GC03P124730	6.818005085
MTHFD1	Methylenetetrahydrofolate Dehydrogenase, Cyclohydrolase And Formyltetrahydrofolate Synthetase 1	Protein Coding	45	GC14P064388	6.634972095
PRTFDC1	Phosphoribosyl Transferase Domain Containing 1	Protein Coding	34	GC10M024848	6.552353382
TYMS	Thymidylate Synthetase	Protein Coding	48	GC18P000657	6.530340672
SUOX	Sulfite Oxidase	Protein Coding	46	GC12P055997	6.124397278
IMPDH1	Inosine Monophosphate Dehydrogenase 1	Protein Coding	50	GC07M128392	6.123563766
NME2	NME/NM23 Nucleoside Diphosphate Kinase 2	Protein Coding	47	GC17P051165	6.003092289
RAG1	Recombination Activating 1	Protein Coding	46	GC11P036520	5.937989712
HINT1	Histidine Triad Nucleotide Binding Protein 1	Protein Coding	45	GC05M131159	5.908269405
GRHPR	Glyoxylate And Hydroxypyruvate Reductase	Protein Coding	47	GC09P037412	5.877959251
ABCC4	ATP Binding Cassette Subfamily C Member 4	Protein Coding	47	GC13M095019	5.861433983
ADORA3	Adenosine A3 Receptor	Protein Coding	47	GC01M111499	5.81032753
MAZ	MYC Associated Zinc Finger Protein	Protein Coding	41	GC16P029806	5.776957512
ADORA1	Adenosine A1 Receptor	Protein Coding	48	GC01P203090	5.773026943
SLC29A1	Solute Carrier Family 29 Member 1 (Augustine Blood Group)	Protein Coding	48	GC06P044219	5.751510143

HSP90AA1	Heat Shock Protein 90 Alpha Family Class A Member 1	Protein Coding	48	GC14M102080	5.707082748
DARS2	Aspartyl-TRNA Synthetase 2, Mitochondrial	Protein Coding	43	GC01P173824	5.690256119
UCP1	Uncoupling Protein 1	Protein Coding	45	GC04M140559	5.660588264
LOC107032760	Origin Of Replication In Promoter/Intron 1 Of HPRT1	Biological Region	2	GC0XP134461	5.63449192
FPGS	Folylpolyglutamate Synthase	Protein Coding	43	GC09P127794	5.470290184
RRM1	Ribonucleotide Reductase Catalytic Subunit M1	Protein Coding	48	GC11P004115	5.397259235
DCLRE1C	DNA Cross-Link Repair 1C	Protein Coding	45	GC10M014897	5.279363632
NHEJ1	Non-Homologous End Joining Factor 1	Protein Coding	43	GC02M219086	5.279363632
RFXANK	Regulatory Factor X Associated Ankyrin Containing Protein	Protein Coding	43	GC19P019192	5.279363632
PNPT1	Polyribonucleotide Nucleotidyltransferase 1	Protein Coding	44	GC02M055634	5.245066643
SLC22A12	Solute Carrier Family 22 Member 12	Protein Coding	44	GC11P064609	5.215261459
PRPS2	Phosphoribosyl Pyrophosphate Synthetase 2	Protein Coding	44	GC0XP012791	5.096124649
UPB1	Beta-Ureidopropionase 1	Protein Coding	44	GC22P024494	5.054831982
SLC2A9	Solute Carrier Family 2 Member 9	Protein Coding	46	GC04M009772	5.047652721
CDKN2A	Cyclin Dependent Kinase Inhibitor 2A	Protein Coding	52	GC09M021967	4.98399353
P2RY6	Pyrimidinergic Receptor P2Y6	Protein Coding	43	GC11P073264	4.967431545

SHMT1	Serine Hydroxymethyltransferase 1	Protein Coding	46	GC17M021636	4.924109459
GUK1	Guanylate Kinase 1	Protein Coding	44	GC01P228139	4.748103619
DNASE1	Deoxyribonuclease 1	Protein Coding	43	GC16P003611	4.631211281
NME1	NME/NM23 Nucleoside Diphosphate Kinase 1	Protein Coding	47	GC17P051154	4.579955101
RNGTT	RNA Guanylyltransferase And 5'-Phosphatase	Protein Coding	44	GC06M088609	4.500836849
ERCC6	ERCC Excision Repair 6, Chromatin Remodeling Factor	Protein Coding	46	GC10M049454	4.445313454
CDK5	Cyclin Dependent Kinase 5	Protein Coding	54	GC07M151053	4.429901123
TP53	Tumor Protein P53	Protein Coding	54	GC17M007661	4.418533802
P2RY2	Purinergic Receptor P2Y2	Protein Coding	47	GC11P073202	4.393837452
SLC17A1	Solute Carrier Family 17 Member 1	Protein Coding	40	GC06M025723	4.358979702
ADORA2B	Adenosine A2b Receptor	Protein Coding	48	GC17P015927	4.351038456
P2RY1	Purinergic Receptor P2Y1	Protein Coding	47	GC03P152835	4.341411114
CAV1	Caveolin 1	Protein Coding	48	GC07P116524	4.315004349
MUTYH	MutY DNA Glycosylase	Protein Coding	45	GC01M045329	4.283921242
PDE4A	Phosphodiesterase 4A	Protein Coding	45	GC19P010416	4.279602051
ADAL	Adenosine Deaminase Like	Protein Coding	34	GC15P043330	4.264083862
P2RY4	Pyrimidinergic Receptor P2Y4	Protein Coding	44	GC0XM070258	4.237788677
GMPR	Guanosine Monophosphate Reductase	Protein Coding	44	GC06P016238	4.23637867
CCNA2	Cyclin A2	Protein Coding	46	GC04M121816	4.185595989
TYMP	Thymidine Phosphorylase	Protein Coding	47	GC22M050525	4.155554771
MTHFR	Methylenetetrahydrofolate Reductase	Protein Coding	49	GC01M011785	4.126284122

POLI	DNA Polymerase Iota	Protein Coding	43	GC18P054274	4.122253895
NGF	Nerve Growth Factor	Protein Coding	51	GC01M115285	4.112717628
PRKACA	Protein Kinase CAMP-Activated Catalytic Subunit Alpha	Protein Coding	52	GC19M014129	4.030287266
DPYS	Dihydropyrimidinase	Protein Coding	47	GC08M104331	4.005625725
TKT	Transketolase	Protein Coding	48	GC03M053224	3.992311478
SHMT2	Serine Hydroxymethyltransferase 2	Protein Coding	46	GC12P057229	3.942404985
TNF	Tumor Necrosis Factor	Protein Coding	51	GC06P055202	3.900313139
P2RX7	Purinergic Receptor P2X 7	Protein Coding	46	GC12P123575	3.898384333
SLC19A1	Solute Carrier Family 19 Member 1	Protein Coding	47	GC21M045493	3.892272472
DNTT	DNA Nucleotidylexotransferase	Protein Coding	43	GC10P096304	3.885804892
POLA1	DNA Polymerase Alpha 1, Catalytic Subunit	Protein Coding	46	GC0XP024693	3.865114689
NT5C	5', 3'-Nucleotidase, Cytosolic	Protein Coding	41	GC17M075130	3.83154583
AK1	Adenylate Kinase 1	Protein Coding	48	GC09M127866	3.806127787
PRODH	Proline Dehydrogenase 1	Protein Coding	47	GC22M018912	3.793948174
IFNB1	Interferon Beta 1	Protein Coding	42	GC09M021077	3.792104959
DNPH1	2'-Deoxynucleoside 5'-Phosphate N-Hydrolase 1	Protein Coding	35	GC06M047130	3.774025679
PYGL	Glycogen Phosphorylase L	Protein Coding	49	GC14M050857	3.700289726
NT5C1A	5'-Nucleotidase, Cytosolic IA	Protein Coding	40	GC01M039659	3.693295956
EN1	Engrailed Homeobox 1	Protein Coding	39	GC02M118842	3.683432341
THAP1	THAP Domain Containing 1	Protein Coding	40	GC08M042836	3.683432341
P2RY10	P2Y Receptor Family Member 10	Protein Coding	41	GC0XP078945	3.564252138

CAT	Catalase	Protein Coding	50	GC11P034460	3.522228718
DPYD-IT1	DPYD Intronic Transcript 1	RNA Gene	10	GC01M097394	3.501078129
SLC7A9	Solute Carrier Family 7 Member 9	Protein Coding	46	GC19M032830	3.501078129
GMPR2	Guanosine Monophosphate Reductase 2	Protein Coding	39	GC14P024232	3.472105503
PRKAA2	Protein Kinase AMP-Activated Catalytic Subunit Alpha 2	Protein Coding	51	GC01P056645	3.425529003
TK1	Thymidine Kinase 1	Protein Coding	47	GC17M078175	3.364658117
ALB	Albumin	Protein Coding	50	GC04P073397	3.331794024
PARP1	Poly(ADP-Ribose) Polymerase 1	Protein Coding	50	GC01M226360	3.318228722
G6PD	Glucose-6-Phosphate Dehydrogenase	Protein Coding	51	GC0XM154531	3.312633991
FOXK2	Forkhead Box K2	Protein Coding	38	GC17P082519	3.309506655
SPI1	Spi-1 Proto-Oncogene	Protein Coding	44	GC11M068998	3.306237459
DNMT1	DNA Methyltransferase 1	Protein Coding	51	GC19M010133	3.294989824
AK4	Adenylate Kinase 4	Protein Coding	41	GC01P065147	3.29101944
PCK2	Phosphoenolpyruvate Carboxykinase 2, Mitochondrial	Protein Coding	48	GC14P024094	3.282449484
UGT1A1	UDP Glucuronosyltransferase Family 1 Member A1	Protein Coding	49	GC02P233760	3.278171301
POLB	DNA Polymerase Beta	Protein Coding	47	GC08P042338	3.269356251
AQP9	Aquaporin 9	Protein Coding	44	GC15P058138	3.235515118
H2AZ2	H2A.Z Variant Histone 2	Protein Coding	27	GC07M044829	3.223104954
NUDT15	Nudix Hydrolase 15	Protein Coding	38	GC13P048037	3.207851171
NT5C1B	5'-Nucleotidase, Cytosolic IB	Protein Coding	38	GC02M018562	3.206442118
MYD88	MYD88 Innate Immune Signal Transduction Adaptor	Protein Coding	50	GC03P038139	3.186019897
IL2	Interleukin 2	Protein Coding	45	GC04M122451	3.182717562

GAPDH	Glyceraldehyde-3-Phosphate Dehydrogenase	Protein Coding	48	GC12P011841	3.16703558
LOC107303343	Adenosine Deaminase Intronic Regulatory Elements	Biological Region	2	GC20P044629	3.164265394
IFNA2	Interferon Alpha 2	Protein Coding	41	GC09M021384	3.152386189
PKN1	Protein Kinase N1	Protein Coding	47	GC19P014433	3.132461309
PDE3B	Phosphodiesterase 3B	Protein Coding	44	GC11P014643	3.081345558
TGS1	Trimethylguanosine Synthase 1	Protein Coding	40	GC08P055773	2.998898983
INS	Insulin	Protein Coding	48	GC11M002159	2.988300085
PDE7A	Phosphodiesterase 7A	Protein Coding	45	GC08M065720	2.980587482
SLC22A6	Solute Carrier Family 22 Member 6	Protein Coding	45	GC11M069278	2.974694252
GALNS	Galactosamine (N-Acetyl)-6-Sulfatase	Protein Coding	45	GC16M088813	2.944138765
FMR1	FMRP Translational Regulator 1	Protein Coding	45	GC0XP147925	2.933513165
ADCY10	Adenylate Cyclase 10	Protein Coding	45	GC01M167809	2.930760145
DTYMK	Deoxythymidylate Kinase	Protein Coding	43	GC02M241675	2.930192471
CSNK2A1	Casein Kinase 2 Alpha 1	Protein Coding	52	GC20M000472	2.91513896
CXCL8	C-X-C Motif Chemokine Ligand 8	Protein Coding	42	GC04P073740	2.912037134
ABCB1	ATP Binding Cassette Subfamily B Member 1	Protein Coding	52	GC07M087504	2.880408287
MTR	5-Methyltetrahydrofolate-Homocysteine Methyltransferase	Protein Coding	47	GC01P236795	2.874047041
APEX1	Apurinic/Apyrimidinic Endodeoxyribonuclease 1	Protein Coding	45	GC14P020455	2.871146917

CCND1	Cyclin D1	Protein Coding	52	GC11P069641	2.86975193
UCK1	Uridine-Cytidine Kinase 1	Protein Coding	39	GC09M131523	2.844044685
NT5M	5',3'-Nucleotidase, Mitochondrial	Protein Coding	37	GC17P017303	2.835562706
PDE4B	Phosphodiesterase 4B	Protein Coding	45	GC01P065792	2.833988905
KDR	Kinase Insert Domain Receptor	Protein Coding	54	GC04M055078	2.81865406
POLH	DNA Polymerase Eta	Protein Coding	49	GC06P043576	2.818390369
SOD1	Superoxide Dismutase 1	Protein Coding	52	GC21P031659	2.811605215
CYP1A2	Cytochrome P450 Family 1 Subfamily A Member 2	Protein Coding	46	GC15P074748	2.807173491
ERVK-6	Endogenous Retrovirus Group K Member 6, Envelope	Protein Coding	16	GC07U903184	2.790398598
SLC22A8	Solute Carrier Family 22 Member 8	Protein Coding	43	GC11M069279	2.790295601
ABCG2	ATP Binding Cassette Subfamily G Member 2 (Junior Blood Group)	Protein Coding	51	GC04M088090	2.778950214
UCK2	Uridine-Cytidine Kinase 2	Protein Coding	42	GC01P165796	2.777539015
MTHFS	Methenyltetrahydrofolate Synthetase	Protein Coding	44	GC15M079833	2.761023521
DPYD-AS1	DPYD Antisense RNA 1	RNA Gene	16	GC01P097095	2.74158287
RRM2	Ribonucleotide Reductase Regulatory Subunit M2	Protein Coding	48	GC02P010123	2.735484838
ENTPD2	Ectonucleoside Triphosphate Diphosphohydrolase 2	Protein Coding	40	GC09M137048	2.734582901
UCP2	Uncoupling Protein 2	Protein Coding	46	GC11M073974	2.731819153
ABCC5	ATP Binding Cassette Subfamily C Member 5	Protein Coding	44	GC03M183919	2.729152679

PIK3CD	Phosphatidylinositol-4,5-Bisphosphate 3-Kinase Catalytic Subunit Delta	Protein Coding	54	GC01P009629	2.721470833
CD38	CD38 Molecule	Protein Coding	45	GC04P015779	2.716682911
GPT	Glutamic--Pyruvic Transaminase	Protein Coding	43	GC08P144502	2.708509922
RRM2B	Ribonucleotide Reductase Regulatory TP53 Inducible Subunit M2B	Protein Coding	50	GC08M102204	2.693305492
CRAT	Carnitine O-Acetyltransferase	Protein Coding	45	GC09M129094	2.692632675
DPP4	Dipeptidyl Peptidase 4	Protein Coding	50	GC02M161992	2.686451674
AKT1	AKT Serine/Threonine Kinase 1	Protein Coding	54	GC14M104769	2.675852299
CAMK2G	Calcium/Calmodulin Dependent Protein Kinase II Gamma	Protein Coding	48	GC10M073812	2.672637939
CYP3A4	Cytochrome P450 Family 3 Subfamily A Member 4	Protein Coding	51	GC07M099759	2.671376944
MTHFD2	Methylenetetrahydrofolate Dehydrogenase (NADP+ Dependent) 2, Methenyltetrahydrofolate Cyclohydrolase	Protein Coding	44	GC02P074186	2.644932985
GLUD1	Glutamate Dehydrogenase 1	Protein Coding	50	GC10M087050	2.640607119
NT5C3A	5'-Nucleotidase, Cytosolic IIIA	Protein Coding	41	GC07M033014	2.634278059
NUDT16	Nudix Hydrolase 16	Protein Coding	36	GC03P131381	2.630273819
ABCC11	ATP Binding Cassette Subfamily C Member 11	Protein Coding	43	GC16M048166	2.628441095
RYR1	Ryanodine Receptor 1	Protein Coding	47	GC19P040452	2.619526386

P2RY12	Purinergic Receptor P2Y12	Protein Coding	49	GC03M151336	2.608891249
AK5	Adenylate Kinase 5	Protein Coding	43	GC01P077281	2.58697772
MCL1	MCL1 Apoptosis Regulator, BCL2 Family Member	Protein Coding	48	GC01M150561	2.579493761
CASP3	Caspase 3	Protein Coding	51	GC04M184627	2.57234931
CBS	Cystathionine Beta-Synthase	Protein Coding	49	GC21M043053	2.56552124
CDKN2B	Cyclin Dependent Kinase Inhibitor 2B	Protein Coding	48	GC09M022002	2.558047533
P2RY8	P2Y Receptor Family Member 8	Protein Coding	38	GC0XM001462	2.549012661
IFNA1	Interferon Alpha 1	Protein Coding	40	GC09P021522	2.544610262
BCL2	BCL2 Apoptosis Regulator	Protein Coding	51	GC18M063123	2.536677837
FGF2	Fibroblast Growth Factor 2	Protein Coding	48	GC04P122826	2.535669804
CYCS	Cytochrome C, Somatic	Protein Coding	49	GC07M025118	2.503499746
MSMB	Microseminoprotein Beta	Protein Coding	41	GC10M046033	2.496563911
FHIT	Fragile Histidine Triad Diadenosine Triphosphatase	Protein Coding	45	GC03M059747	2.470802307
UPP1	Uridine Phosphorylase 1	Protein Coding	41	GC07P048088	2.466930389
CDK4	Cyclin Dependent Kinase 4	Protein Coding	54	GC12M057743	2.462711334
OARD1	O-Acyl-ADP-Ribose Deacylase 1	Protein Coding	33	GC06M047075	2.462524414
AGXT	Alanine--Glyoxylate And Serine--Pyruvate Aminotransferase	Protein Coding	46	GC02P240868	2.461928844
CDK6	Cyclin Dependent Kinase 6	Protein Coding	54	GC07M092604	2.448228836
AK7	Adenylate Kinase 7	Protein Coding	40	GC14P096392	2.442151785
AK8	Adenylate Kinase 8	Protein Coding	40	GC09M132725	2.442151785
TLR9	Toll Like Receptor 9	Protein Coding	45	GC03M052222	2.441901207
PCNA	Proliferating Cell Nuclear Antigen	Protein Coding	52	GC20M005114	2.438406706

CD9	CD9 Molecule	Protein Coding	44	GC12P011813	2.434531689
RNASET2	Ribonuclease T2	Protein Coding	44	GC06M166924	2.402598381
PDE4D	Phosphodiesterase 4D	Protein Coding	49	GC05M058969	2.39304781
EIF4E	Eukaryotic Translation Initiation Factor 4E	Protein Coding	50	GC04M098879	2.37888813
GSTO1	Glutathione S-Transferase Omega 1	Protein Coding	44	GC10P104235	2.372594595
TDG	Thymine DNA Glycosylase	Protein Coding	45	GC12P103965	2.37021637
CDKN3	Cyclin Dependent Kinase Inhibitor 3	Protein Coding	43	GC14P054398	2.36805892
LBR	Lamin B Receptor	Protein Coding	48	GC01M225401	2.364531279
ADA2	Adenosine Deaminase 2	Protein Coding	34	GC22M017179	2.359402895
MUC1	Mucin 1, Cell Surface Associated	Protein Coding	47	GC01M155185	2.341638803
IL6	Interleukin 6	Protein Coding	50	GC07P022725	2.324497461
MAPK1	Mitogen-Activated Protein Kinase 1	Protein Coding	52	GC22M021754	2.317011833
NOS3	Nitric Oxide Synthase 3	Protein Coding	52	GC07P150990	2.313108206
P2RY11	Purinergic Receptor P2Y11	Protein Coding	45	GC19P010184	2.312505722
CD40LG	CD40 Ligand	Protein Coding	48	GC0XP136649	2.307441473
IL4	Interleukin 4	Protein Coding	46	GC05P132673	2.305728436
AS3MT	Arsenite Methyltransferase	Protein Coding	41	GC10P102869	2.299057245
HELLS	Helicase, Lymphoid Specific	Protein Coding	46	GC10P094501	2.289150238
MTHFD1L	Methylenetetrahydrofolate Dehydrogenase (NADP+ Dependent) 1 Like	Protein Coding	43	GC06P150865	2.287996292
ENO2	Enolase 2	Protein Coding	48	GC12P006913	2.276940107
UPF2	UPF2 Regulator Of Nonsense Mediated MRNA Decay	Protein Coding	41	GC10M011920	2.274604321
PDE9A	Phosphodiesterase 9A	Protein Coding	44	GC21P042653	2.27067256

PDE10A	Phosphodiesterase 10A	Protein Coding	47	GC06M165327	2.270196199
IGKV2D-29	Immunoglobulin Kappa Variable 2D-29	Protein Coding	12	GC02P090703	2.246145725
SLC15A1	Solute Carrier Family 15 Member 1	Protein Coding	45	GC13M098683	2.235894203
PDE4C	Phosphodiesterase 4C	Protein Coding	45	GC19M018218	2.228564978
PKM	Pyruvate Kinase M1/2	Protein Coding	46	GC15M072199	2.225922585
CANT1	Calcium Activated Nucleotidase 1	Protein Coding	45	GC17M078992	2.213940382
POLR2L	RNA Polymerase II, I And III Subunit L	Protein Coding	41	GC11M001144	2.212482929
NT5C3B	5'-Nucleotidase, Cytosolic IIIB	Protein Coding	33	GC17M041825	2.203528166
SRSF2	Serine And Arginine Rich Splicing Factor 2	Protein Coding	41	GC17M076734	2.193651199
CD14	CD14 Molecule	Protein Coding	45	GC05M140631	2.191571951
MAPK3	Mitogen-Activated Protein Kinase 3	Protein Coding	50	GC16M031188	2.191388369
SNRPA	Small Nuclear Ribonucleoprotein Polypeptide A	Protein Coding	41	GC19P040750	2.183983564
APP	Amyloid Beta Precursor Protein	Protein Coding	52	GC21M025880	2.179938078
CDKN1A	Cyclin Dependent Kinase Inhibitor 1A	Protein Coding	50	GC06P055348	2.17872262
TG	Thyroglobulin	Protein Coding	43	GC08P132866	2.153712988
MSH2	MutS Homolog 2	Protein Coding	49	GC02P047402	2.140249729
BRAF	B-Raf Proto-Oncogene, Serine/Threonine Kinase	Protein Coding	54	GC07M140717	2.124379396
VIM	Vimentin	Protein Coding	51	GC10P017227	2.123146296
HOGA1	4-Hydroxy-2-Oxoglutarate Aldolase 1	Protein Coding	38	GC10P097585	2.117578506
MPO	Myeloperoxidase	Protein Coding	51	GC17M058269	2.111641169
ELK1	ETS Transcription Factor ELK1	Protein Coding	44	GC0XM047635	2.106223822

MGMT	O-6-Methylguanine-DNA Methyltransferase	Protein Coding	50	GC10P129467	2.092391014
RAPGEF3	Rap Guanine Nucleotide Exchange Factor 3	Protein Coding	45	GC12M047736	2.091523886
AHCYL1	Adenosylhomocysteinase Like 1	Protein Coding	44	GC01P109984	2.084568262
ODC1	Ornithine Decarboxylase 1	Protein Coding	49	GC02M010432	2.067553282
IL1B	Interleukin 1 Beta	Protein Coding	49	GC02M112829	2.061890602
RNASE1	Ribonuclease A Family Member 1, Pancreatic	Protein Coding	40	GC14M020801	2.05702424
HSP90AB1	Heat Shock Protein 90 Alpha Family Class B Member 1	Protein Coding	46	GC06P044246	2.053460836
MS4A1	Membrane Spanning 4-Domains A1	Protein Coding	47	GC11P060475	2.040959597
CSNK1G3	Casein Kinase 1 Gamma 3	Protein Coding	43	GC05P123512	2.034762383
RPA2	Replication Protein A2	Protein Coding	45	GC01M027902	2.0273633
MAPK14	Mitogen-Activated Protein Kinase 14	Protein Coding	52	GC06P055339	2.026161432
MAPK10	Mitogen-Activated Protein Kinase 10	Protein Coding	52	GC04M085990	2.011178732
CGB5	Chorionic Gonadotropin Subunit Beta 5	Protein Coding	33	GC19P049043	2.007963419
IL18	Interleukin 18	Protein Coding	44	GC11M112143	1.999084711
MYC	MYC Proto-Oncogene, BHLH Transcription Factor	Protein Coding	51	GC08P127735	1.99686563
SPRR2F	Small Proline Rich Protein 2F	Protein Coding	28	GC01M153114	1.995131254

SLCO1B1	Solute Carrier Organic Anion Transporter Family Member 1B1	Protein Coding	48	GC12P021132	1.994520545
PDE7B	Phosphodiesterase 7B	Protein Coding	39	GC06P135795	1.991218209
NONO	Non-POU Domain Containing Octamer Binding	Protein Coding	45	GC0XP071255	1.98643446
SFPQ	Splicing Factor Proline And Glutamine Rich	Protein Coding	44	GC01M035176	1.98643446
UNG	Uracil DNA Glycosylase	Protein Coding	47	GC12P109097	1.980410337
MT-RNR1	Mitochondrially Encoded 12S RRNA	RNA Gene	15	GCMTP000642	1.978997946
PDE2A	Phosphodiesterase 2A	Protein Coding	46	GC11M072576	1.976050258
CCND3	Cyclin D3	Protein Coding	48	GC06M041934	1.967175722
SRSF1	Serine And Arginine Rich Splicing Factor 1	Protein Coding	43	GC17M058000	1.966393352
RB1	RB Transcriptional Corepressor 1	Protein Coding	49	GC13P048303	1.964489698
HBB	Hemoglobin Subunit Beta	Protein Coding	46	GC11M005434	1.957989812
TFAP2A	Transcription Factor AP-2 Alpha	Protein Coding	48	GC06M010393	1.951749325
UMOD	Uromodulin	Protein Coding	43	GC16M020344	1.950322032
NME3	NME/NM23 Nucleoside Diphosphate Kinase 3	Protein Coding	44	GC16M001770	1.947336316
NME6	NME/NM23 Nucleoside Diphosphate Kinase 6	Protein Coding	41	GC03M048292	1.947336316
NME7	NME/NM23 Family Member 7	Protein Coding	43	GC01M169101	1.947336316
DNAH8	Dynein Axonemal Heavy Chain 8	Protein Coding	38	GC06P055369	1.941572309
NPY	Neuropeptide Y	Protein Coding	46	GC07P024290	1.935935736
MYLK	Myosin Light Chain Kinase	Protein Coding	53	GC03M123610	1.925219893
SLC6A3	Solute Carrier Family 6 Member 3	Protein Coding	50	GC05M001392	1.919916987

AKR1A1	Aldo-Keto Reductase Family 1 Member A1	Protein Coding	45	GC01P045550	1.91919136
OTC	Ornithine Transcarbamylase	Protein Coding	48	GC0XP038353	1.9180305
GAP43	Growth Associated Protein 43	Protein Coding	44	GC03P115623	1.915141344
URAD	Ureidoimidazoline (2-Oxo-4-Hydroxy-4-Carboxy-5-) Decarboxylase	Protein Coding	26	GC13M027978	1.913477182
DUSP19	Dual Specificity Phosphatase 19	Protein Coding	39	GC02P183078	1.906725287
FASLG	Fas Ligand	Protein Coding	48	GC01P172628	1.90323019
SLC29A3	Solute Carrier Family 29 Member 3	Protein Coding	44	GC10P071320	1.88259685
ACP1	Acid Phosphatase 1	Protein Coding	44	GC02P000254	1.882022858
PGK1	Phosphoglycerate Kinase 1	Protein Coding	49	GC0XP077944	1.875691891
SLC29A4	Solute Carrier Family 29 Member 4	Protein Coding	43	GC07P005289	1.875584483
SLC43A3	Solute Carrier Family 43 Member 3	Protein Coding	38	GC11M069073	1.866211653
PDE8A	Phosphodiesterase 8A	Protein Coding	42	GC15P085364	1.859576106
ACOX1	Acyl-CoA Oxidase 1	Protein Coding	47	GC17M075941	1.853023052
GLUL	Glutamate-Ammonia Ligase	Protein Coding	50	GC01M182378	1.852416754
ITGAM	Integrin Subunit Alpha M	Protein Coding	47	GC16P032553	1.852092028
NME1-NME2	NME1-NME2 Readthrough	Protein Coding	32	GC17P051153	1.847249627
TTR	Transthyretin	Protein Coding	50	GC18P031557	1.847175479
E2F1	E2F Transcription Factor 1	Protein Coding	44	GC20M033675	1.843178988
CYP3A5	Cytochrome P450 Family 3 Subfamily A Member 5	Protein Coding	48	GC07M099648	1.842272401
NEIL1	Nei Like DNA Glycosylase 1	Protein Coding	40	GC15P075346	1.835533619

GGT1	Gamma-Glutamyltransferase 1	Protein Coding	48	GC22P024583	1.827225804
NRF1	Nuclear Respiratory Factor 1	Protein Coding	44	GC07P129611	1.825157404
VIP	Vasoactive Intestinal Peptide	Protein Coding	45	GC06P152750	1.824047089
FAM114A2	Family With Sequence Similarity 114 Member A2	Protein Coding	34	GC05M153990	1.822883129
CSF3	Colony Stimulating Factor 3	Protein Coding	40	GC17P040015	1.820984244
MYB	MYB Proto-Oncogene, Transcription Factor	Protein Coding	51	GC06P135180	1.817529082
ERBB2	Erb-B2 Receptor Tyrosine Kinase 2	Protein Coding	54	GC17P039687	1.814738274
NME2P1	NME2 Pseudogene 1	Pseudogene	19	GC12P120282	1.8025105
NME5	NME/NM23 Family Member 5	Protein Coding	38	GC05M138115	1.8025105
FGFR2	Fibroblast Growth Factor Receptor 2	Protein Coding	54	GC10M121478	1.796739697
BAX	BCL2 Associated X, Apoptosis Regulator	Protein Coding	49	GC19P048954	1.795338511
VWF	Von Willebrand Factor	Protein Coding	49	GC12M005917	1.794823766
CAD	Carbamoyl-Phosphate Synthetase 2, Aspartate Transcarbamylase, And Dihydroorotase	Protein Coding	51	GC02P027217	1.79464674
RAPGEF4	Rap Guanine Nucleotide Exchange Factor 4	Protein Coding	44	GC02P172735	1.791348577
EGF	Epidermal Growth Factor	Protein Coding	51	GC04P109912	1.786958933
NUDT6	Nudix Hydrolase 6	Protein Coding	38	GC04M122888	1.786130428

QTRT1	Queuine TRNA-Ribosyltransferase Catalytic Subunit 1	Protein Coding	38	GC19P010701	1.78574574
CSN1S1	Casein Alpha S1	Protein Coding	34	GC04P069932	1.785568714
PGD	Phosphogluconate Dehydrogenase	Protein Coding	47	GC01P010398	1.785568714
AR	Androgen Receptor	Protein Coding	53	GC0XP067544	1.784520149
PDE8B	Phosphodiesterase 8B	Protein Coding	47	GC05P077180	1.781101823
PDZK1	PDZ Domain Containing 1	Protein Coding	42	GC01M145670	1.775656581
ADPRH	ADP-Ribosylarginine Hydrolase	Protein Coding	37	GC03P119579	1.769706607
IL1R1	Interleukin 1 Receptor Type 1	Protein Coding	46	GC02P102136	1.7687397
CHEK1	Checkpoint Kinase 1	Protein Coding	50	GC11P125625	1.768449068
PRKDC	Protein Kinase, DNA-Activated, Catalytic Subunit	Protein Coding	50	GC08M047773	1.768006444
BID	BH3 Interacting Domain Death Agonist	Protein Coding	46	GC22M017734	1.766082048
PRKG1	Protein Kinase CGMP-Dependent 1	Protein Coding	51	GC10P050991	1.763935328
PRPSAP1	Phosphoribosyl Pyrophosphate Synthetase Associated Protein 1	Protein Coding	36	GC17M076309	1.763041735
ALDH2	Aldehyde Dehydrogenase 2 Family Member	Protein Coding	51	GC12P111766	1.761737943
MAOA	Monoamine Oxidase A	Protein Coding	51	GC0XP043654	1.760390162
F9	Coagulation Factor IX	Protein Coding	47	GC0XP139530	1.753205299
CREB1	CAMP Responsive Element Binding Protein 1	Protein Coding	50	GC02P207529	1.748905897
CYP2D6	Cytochrome P450 Family 2 Subfamily D Member 6	Protein Coding	49	GC22M042126	1.745214462

PRPSAP2	Phosphoribosyl Pyrophosphate Synthetase Associated Protein 2	Protein Coding	35	GC17P019244	1.744968891
FAS	Fas Cell Surface Death Receptor	Protein Coding	51	GC10P088969	1.739263892
ATP5F1A	ATP Synthase F1 Subunit Alpha	Protein Coding	37	GC18M046081	1.737723947
ATP5F1B	ATP Synthase F1 Subunit Beta	Protein Coding	34	GC12M056639	1.737723947
ATP5F1C	ATP Synthase F1 Subunit Gamma	Protein Coding	33	GC10P007789	1.737723947
PDXK	Pyridoxal Kinase	Protein Coding	49	GC21P043719	1.729341388
TH	Tyrosine Hydroxylase	Protein Coding	52	GC11M002163	1.727683544
PIK3CG	Phosphatidylinositol-4,5-Bisphosphate 3-Kinase Catalytic Subunit Gamma	Protein Coding	49	GC07P106865	1.724251509
TAT	Tyrosine Aminotransferase	Protein Coding	45	GC16M071565	1.719253659
RAF1	Raf-1 Proto-Oncogene, Serine/Threonine Kinase	Protein Coding	54	GC03M012583	1.7170434
NTF3	Neurotrophin 3	Protein Coding	43	GC12P005432	1.716531157
TYR	Tyrosinase	Protein Coding	48	GC11P089177	1.715681314
BAK1	BCL2 Antagonist/Killer 1	Protein Coding	45	GC06M033572	1.710592389
LGALS9	Galectin 9	Protein Coding	40	GC17P027629	1.706539869
GSTO2	Glutathione S-Transferase Omega 2	Protein Coding	44	GC10P104268	1.706349254
CDK7	Cyclin Dependent Kinase 7	Protein Coding	47	GC05P069263	1.703610182
BRCA1	BRCA1 DNA Repair Associated	Protein Coding	51	GC17M043044	1.702344418
SP1	Sp1 Transcription Factor	Protein Coding	45	GC12P053380	1.693738103
ELF1	E74 Like ETS Transcription Factor 1	Protein Coding	41	GC13M040932	1.692730188
HRAS	HRas Proto-Oncogene, GTPase	Protein Coding	52	GC11M001078	1.687399149
XIAP	X-Linked Inhibitor Of Apoptosis	Protein Coding	50	GC0XP123859	1.678935051

SLC17A5	Solute Carrier Family 17 Member 5	Protein Coding	45	GC06M073593	1.67356956
B2M	Beta-2-Microglobulin	Protein Coding	49	GC15P044711	1.669553757
RNASE3	Ribonuclease A Family Member 3	Protein Coding	41	GC14P020891	1.660164952
RET	Ret Proto-Oncogene	Protein Coding	54	GC10P043081	1.64372921
CCNB1	Cyclin B1	Protein Coding	47	GC05P069167	1.640868545
DHODH	Dihydroorotate Dehydrogenase (Quinone)	Protein Coding	46	GC16P072008	1.636780739
ETV6	ETS Variant Transcription Factor 6	Protein Coding	47	GC12P011649	1.636412621
ATP5F1D	ATP Synthase F1 Subunit Delta	Protein Coding	34	GC19P001242	1.630720258
ATP5ME	ATP Synthase Membrane Subunit E	Protein Coding	30	GC04M000688	1.630720258
ATP5MF	ATP Synthase Membrane Subunit F	Protein Coding	30	GC07M099449	1.630720258
ATP5PD	ATP Synthase Peripheral Stalk Subunit D	Protein Coding	32	GC17M075039	1.630720258
MT-ATP6	Mitochondrially Encoded ATP Synthase Membrane Subunit 6	Protein Coding	33	GCMTP008531	1.630720258
MT-ATP8	Mitochondrially Encoded ATP Synthase Membrane Subunit 8	Protein Coding	30	GCMTP008368	1.630720258
ILF3	Interleukin Enhancer Binding Factor 3	Protein Coding	39	GC19P010625	1.62781024
ENTPD1	Ectonucleoside Triphosphate Diphosphohydrolase 1	Protein Coding	47	GC10P095711	1.625547886
RHOA	Ras Homolog Family Member A	Protein Coding	48	GC03M049359	1.623340845
RAD51	RAD51 Recombinase	Protein Coding	53	GC15P040694	1.622834325

CDK5R1	Cyclin Dependent Kinase 5 Regulatory Subunit 1	Protein Coding	46	GC17P032486	1.616480589
RUNX1	RUNX Family Transcription Factor 1	Protein Coding	49	GC21M034787	1.615411878
GPX1	Glutathione Peroxidase 1	Protein Coding	48	GC03M049486	1.611110687
GABPA	GA Binding Protein Transcription Factor Subunit Alpha	Protein Coding	41	GC21P025734	1.60645318
NPPA	Natriuretic Peptide A	Protein Coding	47	GC01M011846	1.594173431
RPL11	Ribosomal Protein L11	Protein Coding	49	GC01P023691	1.592294574
SLC22A11	Solute Carrier Family 22 Member 11	Protein Coding	41	GC11P064573	1.590691805
AFP	Alpha Fetoprotein	Protein Coding	46	GC04P073431	1.589891315
RYR2	Ryanodine Receptor 2	Protein Coding	48	GC01P237042	1.586803436
P2RY13	Purinergic Receptor P2Y13	Protein Coding	43	GC03M151326	1.583533525
AHR	Aryl Hydrocarbon Receptor	Protein Coding	49	GC07P016916	1.573916435
MEFV	MEFV Innate Immuity Regulator, Pyrin	Protein Coding	44	GC16M003757	1.570150971
CASP8	Caspase 8	Protein Coding	52	GC02P201233	1.568708062
NLRP3	NLR Family Pyrin Domain Containing 3	Protein Coding	48	GC01P247415	1.568566203
JUN	Jun Proto-Oncogene, AP-1 Transcription Factor Subunit	Protein Coding	50	GC01M058780	1.564620852
ABCC1	ATP Binding Cassette Subfamily C Member 1	Protein Coding	50	GC16P015949	1.545148373
NGFR	Nerve Growth Factor Receptor	Protein Coding	46	GC17P049495	1.537448049
ISG20	Interferon Stimulated Exonuclease Gene 20	Protein Coding	40	GC15P088635	1.530385733

SOD2	Superoxide Dismutase 2	Protein Coding	51	GC06M159669	1.527435064
GATA1	GATA Binding Protein 1	Protein Coding	47	GC0XP048786	1.525143623
LEP	Leptin	Protein Coding	48	GC07P128241	1.52500844
AK3	Adenylate Kinase 3	Protein Coding	43	GC09M004703	1.524489164
CD52	CD52 Molecule	Protein Coding	37	GC01P026317	1.521157146
BIRC5	Baculoviral IAP Repeat Containing 5	Protein Coding	47	GC17P078214	1.518647075
IL2RA	Interleukin 2 Receptor Subunit Alpha	Protein Coding	51	GC10M006010	1.513982773
HSPA8	Heat Shock Protein Family A (Hsp70) Member 8	Protein Coding	48	GC11M123057	1.509647369
GSTM1	Glutathione S-Transferase Mu 1	Protein Coding	43	GC01P109687	1.506895781
FOXN2	Forkhead Box N2	Protein Coding	36	GC02P048314	1.506366491
SPIB	Spi-B Transcription Factor	Protein Coding	41	GC19P050418	1.506366491
CD4	CD4 Molecule	Protein Coding	50	GC12P006786	1.499870777
EEF1A1	Eukaryotic Translation Elongation Factor 1 Alpha 1	Protein Coding	44	GC06M073515	1.498755813
GRK1	G Protein-Coupled Receptor Kinase 1	Protein Coding	43	GC13P113645	1.493915558
GJA1	Gap Junction Protein Alpha 1	Protein Coding	51	GC06P121436	1.493425369
NAT2	N-Acetyltransferase 2	Protein Coding	44	GC08P018391	1.478921294
BMP6	Bone Morphogenetic Protein 6	Protein Coding	44	GC06P007726	1.478631258
THBD	Thrombomodulin	Protein Coding	44	GC20M023026	1.478114605
GABPB1	GA Binding Protein Transcription Factor Subunit Beta 1	Protein Coding	39	GC15M050275	1.477694869
TRIR	Telomerase RNA Component Interacting RNase	Protein Coding	25	GC19M013061	1.477694869
IL18R1	Interleukin 18 Receptor 1	Protein Coding	43	GC02P102311	1.468655348

SGCB	Sarcoglycan Beta	Protein Coding	41	GC04M052019	1.467107654
NDUFS4	NADH:Ubiquinone Oxidoreductase Subunit S4	Protein Coding	45	GC05P053560	1.466682553
PANX1	Pannexin 1	Protein Coding	44	GC11P094128	1.461562514
IFI27	Interferon Alpha Inducible Protein 27	Protein Coding	38	GC14P094104	1.46030724
CNTF	Ciliary Neurotrophic Factor	Protein Coding	42	GC11P058622	1.459991455
BTK	Bruton Tyrosine Kinase	Protein Coding	54	GC0XM101349	1.453420043
P2RX3	Purinergic Receptor P2X 3	Protein Coding	43	GC11P057356	1.451300025
HSD17B10	Hydroxysteroid 17-Beta Dehydrogenase 10	Protein Coding	46	GC0XM053431	1.449696541
NTHL1	Nth Like DNA Glycosylase 1	Protein Coding	45	GC16M003055	1.449663043
KHDRBS3	KH RNA Binding Domain Containing, Signal Transduction Associated 3	Protein Coding	39	GC08P135457	1.443685174
LACTB2	Lactamase Beta 2	Protein Coding	37	GC08M070635	1.443685174
LDHD	Lactate Dehydrogenase D	Protein Coding	41	GC16M075111	1.440338492
CNR1	Cannabinoid Receptor 1	Protein Coding	47	GC06M088139	1.439155936
IL4I1	Interleukin 4 Induced 1	Protein Coding	38	GC19M049890	1.438767433
KNG1	Kininogen 1	Protein Coding	45	GC03P186717	1.438767433
PIK3C2A	Phosphatidylinositol-4-Phosphate 3-Kinase Catalytic Subunit Type 2 Alpha	Protein Coding	48	GC11M017273	1.431478381
PSMC6	Proteasome 26S Subunit, ATPase 6	Protein Coding	41	GC14P052707	1.430076003
NQO2	N-Ribosyldihydronicotinamide:Quinone Reductase 2	Protein Coding	46	GC06P003014	1.426428795
ATP5F1EP2	ATP Synthase F1 Subunit Epsilon Pseudogene 2	Pseudogene	13	GC13P027946	1.423629761

ATP5MG	ATP Synthase Membrane Subunit G	Protein Coding	31	GC11P118403	1.423629761
DMAC2L	Distal Membrane Arm Assembly Component 2 Like	Protein Coding	28	GC14P050312	1.423629761
CDC25C	Cell Division Cycle 25C	Protein Coding	49	GC05M138285	1.420786381
PTPA	Protein Phosphatase 2 Phosphatase Activator	Protein Coding	36	GC09P129111	1.420649529
CSF2	Colony Stimulating Factor 2	Protein Coding	44	GC05P132073	1.420423627
TBP	TATA-Box Binding Protein	Protein Coding	49	GC06P170554	1.416758418
PTPN11	Protein Tyrosine Phosphatase Non-Receptor Type 11	Protein Coding	54	GC12P112418	1.413121462
ELK3	ETS Transcription Factor ELK3	Protein Coding	40	GC12P096194	1.412098885
MLH1	MutL Homolog 1	Protein Coding	49	GC03P036993	1.411007881
PCK1	Phosphoenolpyruvate Carboxykinase 1	Protein Coding	49	GC20P057561	1.410299301
CDK9	Cyclin Dependent Kinase 9	Protein Coding	47	GC09P127852	1.408461094
NCAM1	Neural Cell Adhesion Molecule 1	Protein Coding	47	GC11P112961	1.40597403
ALDH16A1	Aldehyde Dehydrogenase 16 Family Member A1	Protein Coding	36	GC19P049453	1.40535605
SLC17A3	Solute Carrier Family 17 Member 3	Protein Coding	41	GC06M025833	1.40535605
LHPP	Phospholysine Phosphohistidine Inorganic Pyrophosphate Phosphatase	Protein Coding	39	GC10P124461	1.404020309
CD59	CD59 Molecule (CD59 Blood Group)	Protein Coding	47	GC11M033704	1.402095795

MTHFD2L	Methylenetetrahydrofolate Dehydrogenase (NADP+ Dependent) 2 Like	Protein Coding	38	GC04P074114	1.401212335
ACOX3	Acyl-CoA Oxidase 3, Pristanoyl	Protein Coding	41	GC04M008380	1.400308371
MAOB	Monoamine Oxidase B	Protein Coding	44	GC0XM043766	1.400308371
KHDRBS2	KH RNA Binding Domain Containing, Signal Transduction Associated 2	Protein Coding	38	GC06M061542	1.399362803
NR2E3	Nuclear Receptor Subfamily 2 Group E Member 3	Protein Coding	43	GC15P071792	1.395786166
MTOR	Mechanistic Target Of Rapamycin Kinase	Protein Coding	54	GC01M011106	1.395744562
NPPB	Natriuretic Peptide B	Protein Coding	44	GC01M011858	1.389841318
NUDT7	Nudix Hydrolase 7	Protein Coding	38	GC16P077722	1.382842302
GRM1	Glutamate Metabotropic Receptor 1	Protein Coding	51	GC06P145973	1.379802346
ALLC	Allantoicase	Protein Coding	35	GC02P003655	1.377360821
SLC22A1	Solute Carrier Family 22 Member 1	Protein Coding	44	GC06P160121	1.376489401
LPAR6	Lysophosphatidic Acid Receptor 6	Protein Coding	46	GC13M048389	1.373655796
NQO1	NAD(P)H Quinone Dehydrogenase 1	Protein Coding	50	GC16M069706	1.371690035
TK2	Thymidine Kinase 2	Protein Coding	42	GC16M066508	1.3683604
PHGDH	Phosphoglycerate Dehydrogenase	Protein Coding	50	GC01P119660	1.361776471
GSS	Glutathione Synthetase	Protein Coding	47	GC20M034928	1.361651659
CRP	C-Reactive Protein	Protein Coding	47	GC01M159719	1.35888207
S100A9	S100 Calcium Binding Protein A9	Protein Coding	43	GC01P153357	1.354558825
TLR4	Toll Like Receptor 4	Protein Coding	51	GC09P117704	1.354558825

CTRL	Chymotrypsin Like	Protein Coding	40	GC16M067927	1.352987766
HDAC9	Histone Deacetylase 9	Protein Coding	47	GC07P018086	1.350190639
MB	Myoglobin	Protein Coding	44	GC22M035606	1.348813415
MDM2	MDM2 Proto-Oncogene	Protein Coding	53	GC12P068808	1.3435781
POLG	DNA Polymerase Gamma, Catalytic Subunit	Protein Coding	46	GC15M089365	1.339063168
CXCL10	C-X-C Motif Chemokine Ligand 10	Protein Coding	45	GC04M076021	1.337521076
PLAU	Plasminogen Activator, Urokinase	Protein Coding	52	GC10P073909	1.332167029
PIK3CA	Phosphatidylinositol-4,5-Bisphosphate 3-Kinase Catalytic Subunit Alpha	Protein Coding	53	GC03P179148	1.326195121
AGTR1	Angiotensin II Receptor Type 1	Protein Coding	51	GC03P148697	1.326025605
TP73	Tumor Protein P73	Protein Coding	46	GC01P003652	1.323052406
LTF	Lactotransferrin	Protein Coding	44	GC03M046435	1.322806358
ADCY3	Adenylate Cyclase 3	Protein Coding	48	GC02M024819	1.320352554
ENTPD7	Ectonucleoside Triphosphate Diphosphohydrolase 7	Protein Coding	38	GC10P099659	1.319334984
HACD3	3-Hydroxyacyl-CoA Dehydratase 3	Protein Coding	35	GC15P065530	1.317891479
PNPLA1	Patatin Like Phospholipase Domain Containing 1	Protein Coding	37	GC06P055343	1.317891479
URAHP	Urate (Hydroxyiso-) Hydrolase, Pseudogene	Pseudogene	11	GC16M090039	1.317891479
ALK	ALK Receptor Tyrosine Kinase	Protein Coding	52	GC02M029190	1.315690517
PDE3A	Phosphodiesterase 3A	Protein Coding	50	GC12P020294	1.315358877
NFATC2	Nuclear Factor Of Activated T Cells 2	Protein Coding	45	GC20M051386	1.315081835

RAC1	Rac Family Small GTPase 1	Protein Coding	50	GC07P006377	1.314537883
AGT	Angiotensinogen	Protein Coding	50	GC01M230702	1.313419938
NR1I2	Nuclear Receptor Subfamily 1 Group I Member 2	Protein Coding	47	GC03P119780	1.312730193
PDE6G	Phosphodiesterase 6G	Protein Coding	45	GC17M081650	1.310285091
APC	APC Regulator Of WNT Signaling Pathway	Protein Coding	49	GC05P112707	1.309754729
SLPI	Secretory Leukocyte Peptidase Inhibitor	Protein Coding	40	GC20M045252	1.309754729
IL10	Interleukin 10	Protein Coding	48	GC01M206767	1.300551772
SLC16A9	Solute Carrier Family 16 Member 9	Protein Coding	40	GC10M059650	1.298794508
ALDH9A1	Aldehyde Dehydrogenase 9 Family Member A1	Protein Coding	42	GC01M165670	1.2975564
RHO	Rhodopsin	Protein Coding	48	GC03P131457	1.296518207
POR	Cytochrome P450 Oxidoreductase	Protein Coding	50	GC07P075899	1.293357849
TTF2	Transcription Termination Factor 2	Protein Coding	40	GC01P117060	1.293304682
GABPB2	GA Binding Protein Transcription Factor Subunit Beta 2	Protein Coding	36	GC01P151070	1.292359114
HINT3	Histidine Triad Nucleotide Binding Protein 3	Protein Coding	32	GC06P125956	1.292359114
PPP1R8	Protein Phosphatase 1 Regulatory Subunit 8	Protein Coding	40	GC01P027830	1.292359114
SPIC	Spi-C Transcription Factor	Protein Coding	36	GC12P101474	1.292359114
LPAR4	Lysophosphatidic Acid Receptor 4	Protein Coding	41	GC0XP078747	1.290663362
ADRA2B	Adrenoceptor Alpha 2B	Protein Coding	47	GC02M096112	1.287954569
HTR1A	5-Hydroxytryptamine Receptor 1A	Protein Coding	48	GC05M063960	1.287954569

ADCY1	Adenylate Cyclase 1	Protein Coding	49	GC07P045580	1.285110116
PDE11A	Phosphodiesterase 11A	Protein Coding	47	GC02M177624	1.285110116
CHUK	Component Of Inhibitor Of Nuclear Factor Kappa B Kinase Complex	Protein Coding	52	GC10M100188	1.281615019
NPHS1	NPHS1 Adhesion Molecule, Nephrin	Protein Coding	45	GC19M035825	1.27975297
UCP3	Uncoupling Protein 3	Protein Coding	44	GC11M074000	1.271244287
BCL2L1	BCL2 Like 1	Protein Coding	47	GC20M031664	1.270834327
DBH	Dopamine Beta-Hydroxylase	Protein Coding	51	GC09P133636	1.270486832
POLG2	DNA Polymerase Gamma 2, Accessory Subunit	Protein Coding	42	GC17M064477	1.267669797
HNRNPA1	Heterogeneous Nuclear Ribonucleoprotein A1	Protein Coding	47	GC12P054280	1.260740519
PACSIN2	Protein Kinase C And Casein Kinase Substrate In Neurons 2	Protein Coding	41	GC22M042835	1.260575414
CYP2E1	Cytochrome P450 Family 2 Subfamily E Member 1	Protein Coding	46	GC10P133520	1.260034323
ACADM	Acyl-CoA Dehydrogenase Medium Chain	Protein Coding	47	GC01P075724	1.259681821
CDH1	Cadherin 1	Protein Coding	51	GC16P068737	1.259681821
CP	Ceruloplasmin	Protein Coding	48	GC03M149162	1.259681821
GJB2	Gap Junction Protein Beta 2	Protein Coding	47	GC13M020187	1.259681821
CARMIL1	Capping Protein Regulator And Myosin 1 Linker 1	Protein Coding	28	GC06P025322	1.254472136
EPO	Erythropoietin	Protein Coding	41	GC07P100720	1.251878381
ABL1	ABL Proto-Oncogene 1, Non-Receptor Tyrosine Kinase	Protein Coding	53	GC09P130713	1.251044154

MAP2K1	Mitogen-Activated Protein Kinase Kinase 1	Protein Coding	54	GC15P066386	1.249554753
PTP4A2	Protein Tyrosine Phosphatase 4A2	Protein Coding	42	GC01M031907	1.247036576
THBS1	Thrombospondin 1	Protein Coding	45	GC15P039581	1.247036576
PRKAA1	Protein Kinase AMP-Activated Catalytic Subunit Alpha 1	Protein Coding	48	GC05M040759	1.245984077
TGFA	Transforming Growth Factor Alpha	Protein Coding	46	GC02M070447	1.24472034
CTLA4	Cytotoxic T-Lymphocyte Associated Protein 4	Protein Coding	46	GC02P203867	1.242542505
EGFR	Epidermal Growth Factor Receptor	Protein Coding	54	GC07P055019	1.241660595
MT3	Metallothionein 3	Protein Coding	40	GC16P056589	1.238345027
DNMT3A	DNA Methyltransferase 3 Alpha	Protein Coding	52	GC02M025228	1.227399349
GSR	Glutathione-Disulfide Reductase	Protein Coding	50	GC08M030678	1.227399349
XRCC6	X-Ray Repair Cross Complementing 6	Protein Coding	47	GC22P041622	1.225850582
RPGR	Retinitis Pigmentosa GTPase Regulator	Protein Coding	41	GC0XM038269	1.225257397
LOC106029240	S232-VCX3A Recombination Region	Biological Region	1	GC0XP006531	1.212331295
LOC106029241	S232-VCX2 Recombination Region	Biological Region	1	GC0XP008167	1.212331295
MYOD1	Myogenic Differentiation 1	Protein Coding	47	GC11P017741	1.208686829
CYBB	Cytochrome B-245 Beta Chain	Protein Coding	47	GC0XP037780	1.207534194
HMBS	Hydroxymethylbilane Synthase	Protein Coding	45	GC11P119084	1.207534194
TIMP1	TIMP Metallopeptidase Inhibitor 1	Protein Coding	45	GC0XP047583	1.207534194

TIMP2	TIMP Metallopeptidase Inhibitor 2	Protein Coding	44	GC17M078852	1.207534194
APOE	Apolipoprotein E	Protein Coding	51	GC19P044906	1.207284093
EGR1	Early Growth Response 1	Protein Coding	44	GC05P138465	1.207284093
PTK2B	Protein Tyrosine Kinase 2 Beta	Protein Coding	50	GC08P027311	1.207284093
PIK3R1	Phosphoinositide-3-Kinase Regulatory Subunit 1	Protein Coding	51	GC05P068215	1.204941392
SLC22A7	Solute Carrier Family 22 Member 7	Protein Coding	43	GC06P055408	1.202823162
MYH7	Myosin Heavy Chain 7	Protein Coding	48	GC14M023412	1.201809168
GCH1	GTP Cyclohydrolase 1	Protein Coding	48	GC14M054842	1.198206067
ADCY5	Adenylate Cyclase 5	Protein Coding	49	GC03M123282	1.195410967
IRF1	Interferon Regulatory Factor 1	Protein Coding	47	GC05M132440	1.18964386
TSC2	TSC Complex Subunit 2	Protein Coding	50	GC16P005579	1.189337611
MYH6	Myosin Heavy Chain 6	Protein Coding	47	GC14M023380	1.189323068
SRC	SRC Proto-Oncogene, Non-Receptor Tyrosine Kinase	Protein Coding	52	GC20P037344	1.1893152
SELL	Selectin L	Protein Coding	43	GC01M169690	1.18635416
EIF2AK2	Eukaryotic Translation Initiation Factor 2 Alpha Kinase 2	Protein Coding	46	GC02M037099	1.185347438
NFATC1	Nuclear Factor Of Activated T Cells 1	Protein Coding	48	GC18P079395	1.185302973
PAWR	Pro-Apoptotic WT1 Regulator	Protein Coding	41	GC12M079574	1.185302973
XRCC5	X-Ray Repair Cross Complementing 5	Protein Coding	45	GC02P216107	1.185302973
HSD11B2	Hydroxysteroid 11-Beta Dehydrogenase 2	Protein Coding	47	GC16P067433	1.18330276
INSR	Insulin Receptor	Protein Coding	54	GC19M007112	1.18330276

TSHR	Thyroid Stimulating Hormone Receptor	Protein Coding	48	GC14P080954	1.183076978
ATM	ATM Serine/Threonine Kinase	Protein Coding	54	GC11P108222	1.180466771
AK9	Adenylate Kinase 9	Protein Coding	34	GC06M109492	1.180138826
ENPP4	Ectonucleotide Pyrophosphatase/Phosphodiesterase 4	Protein Coding	34	GC06P046129	1.180138826
ENPP3	Ectonucleotide Pyrophosphatase/Phosphodiesterase 3	Protein Coding	44	GC06P131617	1.177717924
CFTR	CF Transmembrane Conductance Regulator	Protein Coding	52	GC07P117287	1.173929095
KCNMA1	Potassium Calcium-Activated Channel Subfamily M Alpha 1	Protein Coding	50	GC10M076869	1.173826098
CYP2C9	Cytochrome P450 Family 2 Subfamily C Member 9	Protein Coding	50	GC10P094938	1.171480298
MECP2	Methyl-CpG Binding Protein 2	Protein Coding	46	GC0XM154021	1.171480298
SETD2	SET Domain Containing 2, Histone Lysine Methyltransferase	Protein Coding	47	GC03M047033	1.171480298
MSH6	MutS Homolog 6	Protein Coding	51	GC02P047695	1.16890955
GRM2	Glutamate Metabotropic Receptor 2	Protein Coding	45	GC03P051707	1.163483143
CS	Citrate Synthase	Protein Coding	45	GC12M056271	1.162961721
LDLR	Low Density Lipoprotein Receptor	Protein Coding	50	GC19P011091	1.162961721
SRY	Sex Determining Region Y	Protein Coding	36	GC0YM002698	1.162961721
PKLR	Pyruvate Kinase L/R	Protein Coding	48	GC01M155289	1.161768794
GSTP1	Glutathione S-Transferase Pi 1	Protein Coding	51	GC11P067583	1.156948805

RNPC3	RNA Binding Region (RNP1, RRM) Containing 3	Protein Coding	36	GC01P103525	1.156675935
PDE1B	Phosphodiesterase 1B	Protein Coding	44	GC12P054549	1.154767036
NAT1	N-Acetyltransferase 1	Protein Coding	45	GC08P018179	1.153030634
TSPO	Translocator Protein	Protein Coding	44	GC22P043151	1.149144769
ALPK1	Alpha Kinase 1	Protein Coding	40	GC04P112285	1.147468448
PYCARD	PYD And CARD Domain Containing	Protein Coding	43	GC16M031201	1.147468448
SLC22A13	Solute Carrier Family 22 Member 13	Protein Coding	38	GC03P038265	1.147468448
LPO	Lactoperoxidase	Protein Coding	39	GC17P058218	1.144496799
CD40	CD40 Molecule	Protein Coding	48	GC20P046118	1.139241099
CNR2	Cannabinoid Receptor 2	Protein Coding	46	GC01M023870	1.138980389
IKBKB	Inhibitor Of Nuclear Factor Kappa B Kinase Subunit Beta	Protein Coding	53	GC08P042271	1.138980389
SLC2A2	Solute Carrier Family 2 Member 2	Protein Coding	50	GC03M170996	1.138980389
NUDT2	Nudix Hydrolase 2	Protein Coding	41	GC09P034329	1.136535406
ENTPD3	Ectonucleoside Triphosphate Diphosphohydrolase 3	Protein Coding	42	GC03P040403	1.136260629
INTS2	Integrator Complex Subunit 2	Protein Coding	35	GC17M061865	1.134343266
ACAA1	Acetyl-CoA Acyltransferase 1	Protein Coding	44	GC03M038103	1.13428092
APOB	Apolipoprotein B	Protein Coding	46	GC02M020956	1.13428092
F5	Coagulation Factor V	Protein Coding	45	GC01M169511	1.13428092
FOLH1	Folate Hydrolase 1	Protein Coding	47	GC11M069030	1.13428092
PECR	Peroxisomal Trans-2-Enoyl-CoA Reductase	Protein Coding	43	GC02M215996	1.13428092

SLC47A1	Solute Carrier Family 47 Member 1	Protein Coding	42	GC17P019495	1.13428092
SLC47A2	Solute Carrier Family 47 Member 2	Protein Coding	39	GC17M019678	1.13428092
ANXA5	Annexin A5	Protein Coding	47	GC04M121667	1.130239844
CXCR4	C-X-C Motif Chemokine Receptor 4	Protein Coding	52	GC02M136114	1.125174999
JAK3	Janus Kinase 3	Protein Coding	52	GC19M017824	1.125174999
RNASEL	Ribonuclease L	Protein Coding	45	GC01M182542	1.122666121
PKIA	CAMP-Dependent Protein Kinase Inhibitor Alpha	Protein Coding	42	GC08P078472	1.122442842
TNFRSF1A	TNF Receptor Superfamily Member 1A	Protein Coding	50	GC12M006328	1.12153542
RHOD	Ras Homolog Family Member D	Protein Coding	40	GC11P067057	1.115956903
SLC15A2	Solute Carrier Family 15 Member 2	Protein Coding	40	GC03P121894	1.115216017
ITGAL	Integrin Subunit Alpha L	Protein Coding	47	GC16P030472	1.111472249
NFYA	Nuclear Transcription Factor Y Subunit Alpha	Protein Coding	40	GC06P055382	1.108534336
ADH5	Alcohol Dehydrogenase 5 (Class III), Chi Polypeptide	Protein Coding	47	GC04M099070	1.107991457
ILF2	Interleukin Enhancer Binding Factor 2	Protein Coding	40	GC01M153661	1.107991457
FEV	FEV Transcription Factor, ETS Family Member	Protein Coding	36	GC02M218981	1.105327606
P2RY14	Purinergic Receptor P2Y14	Protein Coding	43	GC03M151212	1.105327606
AACS	Acetoacetyl-CoA Synthetase	Protein Coding	40	GC12P125065	1.102709532
CDKN1B	Cyclin Dependent Kinase Inhibitor 1B	Protein Coding	48	GC12P012722	1.10079515
GP1BA	Glycoprotein Ib Platelet Subunit Alpha	Protein Coding	46	GC17P004932	1.100333571

SULT1A1	Sulfotransferase Family 1A Member 1	Protein Coding	43	GC16M028606	1.100333571
TF	Transferrin	Protein Coding	50	GC03P133666	1.100333571
EEF2	Eukaryotic Translation Elongation Factor 2	Protein Coding	49	GC19M003976	1.100174427
CCR4	C-C Motif Chemokine Receptor 4	Protein Coding	45	GC03P032951	1.097556829
TXNRD2	Thioredoxin Reductase 2	Protein Coding	48	GC22M019863	1.095819473
ALDH1A1	Aldehyde Dehydrogenase 1 Family Member A1	Protein Coding	48	GC09M072900	1.094417095
CCL5	C-C Motif Chemokine Ligand 5	Protein Coding	44	GC17M035871	1.091202378
PTGS2	Prostaglandin-Endoperoxide Synthase 2	Protein Coding	49	GC01M186640	1.088962913
EIF4G1	Eukaryotic Translation Initiation Factor 4 Gamma 1	Protein Coding	47	GC03P184314	1.082333922
PDGFRB	Platelet Derived Growth Factor Receptor Beta	Protein Coding	55	GC05M150113	1.082333922
DDIT3	DNA Damage Inducible Transcript 3	Protein Coding	46	GC12M057516	1.078343749
EIF2S1	Eukaryotic Translation Initiation Factor 2 Subunit Alpha	Protein Coding	45	GC14P067359	1.078343749
GTF3A	General Transcription Factor IIIA	Protein Coding	37	GC13P027427	1.078343749
SNAPC4	Small Nuclear RNA Activating Complex Polypeptide 4	Protein Coding	37	GC09M136375	1.077024102
MMP3	Matrix Metallopeptidase 3	Protein Coding	51	GC11M102835	1.076073289
CYP2C19	Cytochrome P450 Family 2 Subfamily C Member 19	Protein Coding	48	GC10P094762	1.076073289

HLA-DRB1	Major Histocompatibility Complex, Class II, DR Beta 1	Protein Coding	48	GC06M032578	1.076073289
ACADVL	Acyl-CoA Dehydrogenase Very Long Chain	Protein Coding	46	GC17P007219	1.075513959
TNFSF10	TNF Superfamily Member 10	Protein Coding	47	GC03M172505	1.074209929
KRAS	KRAS Proto-Oncogene, GTPase	Protein Coding	52	GC12M025204	1.072651982
ADCY8	Adenylate Cyclase 8	Protein Coding	45	GC08M130780	1.070714235
PLK1	Polo Like Kinase 1	Protein Coding	50	GC16P023958	1.069688439
MAP2	Microtubule Associated Protein 2	Protein Coding	43	GC02P209424	1.067862988
DES	Desmin	Protein Coding	48	GC02P219418	1.06570828
MBP	Myelin Basic Protein	Protein Coding	45	GC18M076978	1.06415391
RHEB	Ras Homolog, MTORC1 Binding	Protein Coding	49	GC07M151466	1.06415391
ENPP1	Ectonucleotide Pyrophosphatase/Phosphodiesterase 1	Protein Coding	48	GC06P131808	1.061681986
ACP3	Acid Phosphatase 3	Protein Coding	36	GC03P132319	1.06073904
IL2RB	Interleukin 2 Receptor Subunit Beta	Protein Coding	49	GC22M037125	1.0560112
SRD5A2	Steroid 5 Alpha-Reductase 2	Protein Coding	43	GC02M031522	1.0560112
ZFP36	ZFP36 Ring Finger Protein	Protein Coding	40	GC19P039406	1.0560112
FTO	FTO Alpha-Ketoglutarate Dependent Dioxygenase	Protein Coding	45	GC16P053737	1.055958033
GH1	Growth Hormone 1	Protein Coding	44	GC17M063917	1.055958033
HLA-DQA1	Major Histocompatibility Complex, Class II, DQ Alpha 1	Protein Coding	43	GC06P055231	1.055958033
MBD4	Methyl-CpG Binding Domain 4, DNA Glycosylase	Protein Coding	43	GC03M129430	1.055958033

TGFBI	Transforming Growth Factor Beta Induced	Protein Coding	46	GC05P136027	1.055958033
THRB	Thyroid Hormone Receptor Beta	Protein Coding	51	GC03M024117	1.055958033
ADAR	Adenosine Deaminase RNA Specific	Protein Coding	45	GC01M154582	1.054012179
HMGCS1	3-Hydroxy-3-Methylglutaryl-CoA Synthase 1	Protein Coding	44	GC05M043288	1.053822041
PDE1A	Phosphodiesterase 1A	Protein Coding	44	GC02M182140	1.047763348
PDE1C	Phosphodiesterase 1C	Protein Coding	46	GC07M031616	1.047763348
BMP4	Bone Morphogenetic Protein 4	Protein Coding	50	GC14M053949	1.039404392
PPIG	Peptidylprolyl Isomerase G	Protein Coding	43	GC02P169584	1.039404392
TOP1	DNA Topoisomerase I	Protein Coding	49	GC20P041028	1.036965847
CD19	CD19 Molecule	Protein Coding	50	GC16P032270	1.03680253
RAG2	Recombination Activating 2	Protein Coding	43	GC11M036575	1.03680253
AHCYL2	Adenosylhomocysteinase Like 2	Protein Coding	39	GC07P129225	1.035452008
COMT	Catechol-O-Methyltransferase	Protein Coding	52	GC22P019941	1.035452008
GFAP	Glial Fibrillary Acidic Protein	Protein Coding	48	GC17M044905	1.035452008
HADHB	Hydroxyacyl-CoA Dehydrogenase Trifunctional Multienzyme Complex Subunit Beta	Protein Coding	48	GC02P026243	1.035452008
BGLAP	Bone Gamma-Carboxyglutamate Protein	Protein Coding	41	GC01P156242	1.033140182
BMP2	Bone Morphogenetic Protein 2	Protein Coding	47	GC20P006696	1.033140182
GATA3	GATA Binding Protein 3	Protein Coding	50	GC10P008045	1.033140182
GRM4	Glutamate Metabotropic Receptor 4	Protein Coding	45	GC06M047013	1.033140182

CMPK1	Cytidine/Uridine Monophosphate Kinase 1	Protein Coding	44	GC01P047333	1.032067537
RECK	Reversion Inducing Cysteine Rich Protein With Kazal Motifs	Protein Coding	41	GC09P036036	1.0319767
PNKP	Polynucleotide Kinase 3'-Phosphatase	Protein Coding	46	GC19M049861	1.03164959
GRP	Gastrin Releasing Peptide	Protein Coding	41	GC18P059220	1.031191587
CFLAR	CASP8 And FADD Like Apoptosis Regulator	Protein Coding	47	GC02P201117	1.03020227
MKNK1	MAPK Interacting Serine/Threonine Kinase 1	Protein Coding	47	GC01M046557	1.029283404
CTNNB1	Catenin Beta 1	Protein Coding	54	GC03P041236	1.025366068
CYP2B6	Cytochrome P450 Family 2 Subfamily B Member 6	Protein Coding	48	GC19P040991	1.022500038
FGA	Fibrinogen Alpha Chain	Protein Coding	48	GC04M154583	1.018473029
POMC	Proopiomelanocortin	Protein Coding	48	GC02M025160	1.014531732
SLC6A2	Solute Carrier Family 6 Member 2	Protein Coding	49	GC16P055656	1.014531732
ICAM1	Intercellular Adhesion Molecule 1	Protein Coding	51	GC19P010270	1.012208104
FOLR1	Folate Receptor Alpha	Protein Coding	47	GC11P072190	1.010222197
LCAT	Lecithin-Cholesterol Acyltransferase	Protein Coding	47	GC16M067939	1.010222197
PARN	Poly(A)-Specific Ribonuclease	Protein Coding	47	GC16M014435	1.010222197
PPP2CA	Protein Phosphatase 2 Catalytic Subunit Alpha	Protein Coding	50	GC05M134194	1.010222197
TCF7L2	Transcription Factor 7 Like 2	Protein Coding	46	GC10P112950	1.010222197
TRC-GCA24-1	TRNA-Cys (GCA) 24-1	RNA Gene	7	GC17M038993	1.010222197

CKM	Creatine Kinase, M-Type	Protein Coding	45	GC19M045306	1.009943962
CD79A	CD79a Molecule	Protein Coding	47	GC19P041877	1.009689808
CEACAM3	CEA Cell Adhesion Molecule 3	Protein Coding	41	GC19P041796	1.009689808
PLIN2	Perilipin 2	Protein Coding	44	GC09M019127	1.009689808
HMGB1	High Mobility Group Box 1	Protein Coding	45	GC13M030456	1.008637428
NOS2	Nitric Oxide Synthase 2	Protein Coding	50	GC17M027756	1.004910111
PDE6A	Phosphodiesterase 6A	Protein Coding	48	GC05M149857	1.003565788
PDE6B	Phosphodiesterase 6B	Protein Coding	47	GC04P000587	1.003565788
PDE6C	Phosphodiesterase 6C	Protein Coding	44	GC10P093612	1.003565788
PIK3CB	Phosphatidylinositol-4,5-Bisphosphate 3-Kinase Catalytic Subunit Beta	Protein Coding	50	GC03M138652	0.999227703
TGFB1	Transforming Growth Factor Beta 1	Protein Coding	52	GC19M041301	0.995877862
EIF4EBP1	Eukaryotic Translation Initiation Factor 4E Binding Protein 1	Protein Coding	47	GC08P038032	0.993791521
S100B	S100 Calcium Binding Protein B	Protein Coding	46	GC21M048326	0.993170738
SOD3	Superoxide Dismutase 3	Protein Coding	41	GC04P024798	0.993170738
GAMT	Guanidinoacetate N-Methyltransferase	Protein Coding	47	GC19M001397	0.987915039
HBG1	Hemoglobin Subunit Gamma 1	Protein Coding	43	GC11M005431	0.987393558
ARHGEF2	Rho/Rac Guanine Nucleotide Exchange Factor 2	Protein Coding	47	GC01M155946	0.985613883
CCNT1	Cyclin T1	Protein Coding	41	GC12M048688	0.985411823
PDE6H	Phosphodiesterase 6H	Protein Coding	41	GC12P014982	0.984934628
SST	Somatostatin	Protein Coding	43	GC03M187668	0.98406291

ALDH6A1	Aldehyde Dehydrogenase 6 Family Member A1	Protein Coding	47	GC14M074059	0.984021783
CALB1	Calbindin 1	Protein Coding	43	GC08M090058	0.979965866
EIF4A2	Eukaryotic Translation Initiation Factor 4A2	Protein Coding	45	GC03P186783	0.979965866
BCL2L2	BCL2 Like 2	Protein Coding	45	GC14P026355	0.976212382
GLUD1P5	Glutamate Dehydrogenase 1 Pseudogene 5	Pseudogene	6	GC10P031617	0.976212382
PAX6	Paired Box 6	Protein Coding	48	GC11M031784	0.976212382
SIX3	SIX Homeobox 3	Protein Coding	43	GC02P044941	0.976212382
SLC46A1	Solute Carrier Family 46 Member 1	Protein Coding	44	GC17M031080	0.976212382
USP7	Ubiquitin Specific Peptidase 7	Protein Coding	49	GC16M008892	0.976212382
HDAC2	Histone Deacetylase 2	Protein Coding	52	GC06M113933	0.974412858
ACACB	Acetyl-CoA Carboxylase Beta	Protein Coding	47	GC12P109116	0.97134012
ATP5F1E	ATP Synthase F1 Subunit Epsilon	Protein Coding	32	GC20M059026	0.97134012
BCS1L	BCS1 Homolog, Ubiquinol-Cytochrome C Reductase Complex Chaperone	Protein Coding	45	GC02P218658	0.97134012
ELAVL1	ELAV Like RNA Binding Protein 1	Protein Coding	43	GC19M007958	0.97134012
KCNJ11	Potassium Inwardly Rectifying Channel Subfamily J Member 11	Protein Coding	48	GC11M017385	0.97134012
SRPK2	SRSF Protein Kinase 2	Protein Coding	45	GC07M105110	0.97134012
TGFBR2	Transforming Growth Factor Beta Receptor 2	Protein Coding	51	GC03P030623	0.97134012
TIA1	TIA1 Cytotoxic Granule Associated RNA Binding Protein	Protein Coding	44	GC02M070209	0.97134012

TRPM4	Transient Receptor Potential Cation Channel Subfamily M Member 4	Protein Coding	46	GC19P049157	0.97134012
PSEN1	Presenilin 1	Protein Coding	52	GC14P073136	0.965621591
GRB2	Growth Factor Receptor Bound Protein 2	Protein Coding	49	GC17M075318	0.960859358
LIF	LIF Interleukin 6 Family Cytokine	Protein Coding	43	GC22M030240	0.960859358
RASA1	RAS P21 Protein Activator 1	Protein Coding	47	GC05P087267	0.960859358
TNFSF11	TNF Superfamily Member 11	Protein Coding	48	GC13P042562	0.960859358
EP300	E1A Binding Protein P300	Protein Coding	51	GC22P041091	0.95900619
CTSK	Cathepsin K	Protein Coding	50	GC01M150837	0.954472542
KMT2A	Lysine Methyltransferase 2A	Protein Coding	45	GC11P118436	0.950053155
SRM	Spermidine Synthase	Protein Coding	42	GC01M011054	0.949928403
CYB5R3	Cytochrome B5 Reductase 3	Protein Coding	45	GC22M042617	0.949007511
FANCC	FA Complementation Group C	Protein Coding	48	GC09M095099	0.949007511
IDH1	Isocitrate Dehydrogenase (NADP(+)) 1	Protein Coding	53	GC02M208236	0.949007511
IDO1	Indoleamine 2,3-Dioxygenase 1	Protein Coding	45	GC08P039891	0.949007511
IRF9	Interferon Regulatory Factor 9	Protein Coding	45	GC14P024161	0.949007511
MBD2	Methyl-CpG Binding Domain Protein 2	Protein Coding	41	GC18M054151	0.949007511
MTTP	Microsomal Triglyceride Transfer Protein	Protein Coding	45	GC04P099563	0.949007511
PI4K2A	Phosphatidylinositol 4-Kinase Type 2 Alpha	Protein Coding	41	GC10P097640	0.949007511
PI4K2B	Phosphatidylinositol 4-Kinase Type 2 Beta	Protein Coding	40	GC04P025167	0.949007511

SCGB1A1	Secretoglobin Family 1A Member 1	Protein Coding	41	GC11P062405	0.949007511
SLC35B2	Solute Carrier Family 35 Member B2	Protein Coding	40	GC06M044254	0.949007511
SLC35B3	Solute Carrier Family 35 Member B3	Protein Coding	36	GC06M008413	0.949007511
TFB1M	Transcription Factor B1, Mitochondrial	Protein Coding	43	GC06M155247	0.949007511
UGT1A	UDP Glucuronosyltransferase Family 1 Member A Complex Locus	Genetic Locus	10	GC02P233592	0.949007511
VAV1	Vav Guanine Nucleotide Exchange Factor 1	Protein Coding	47	GC19P006772	0.949007511
WT1	WT1 Transcription Factor	Protein Coding	50	GC11M032365	0.949007511
ANXA1	Annexin A1	Protein Coding	50	GC09P073151	0.945956111
MDH2	Malate Dehydrogenase 2	Protein Coding	49	GC07P076048	0.945956111
NTRK3	Neurotrophic Receptor Tyrosine Kinase 3	Protein Coding	52	GC15M087859	0.945956111
PRMT5	Protein Arginine Methyltransferase 5	Protein Coding	44	GC14M022920	0.944904089
BAG1	BAG Cochaperone 1	Protein Coding	43	GC09M033245	0.942659795
KHDRBS1	KH RNA Binding Domain Containing, Signal Transduction Associated 1	Protein Coding	43	GC01P032013	0.940388143
TRPA1	Transient Receptor Potential Cation Channel Subfamily A Member 1	Protein Coding	46	GC08M072019	0.938515902
GLA	Galactosidase Alpha	Protein Coding	49	GC0XM101393	0.937910259

GNB3	G Protein Subunit Beta 3	Protein Coding	48	GC12P006839	0.937761605
GATA4	GATA Binding Protein 4	Protein Coding	49	GC08P011676	0.935365319
GZMB	Granzyme B	Protein Coding	46	GC14M024630	0.935365319
SCT	Secretin	Protein Coding	35	GC11M000626	0.935365319
SLC10A2	Solute Carrier Family 10 Member 2	Protein Coding	43	GC13M103043	0.935365319
TNFRSF11B	TNF Receptor Superfamily Member 11b	Protein Coding	48	GC08M118923	0.935365319
ABAT	4-Aminobutyrate Aminotransferase	Protein Coding	46	GC16P008674	0.93242538
BIRC3	Baculoviral IAP Repeat Containing 3	Protein Coding	47	GC11P102317	0.93242538
CYP1A1	Cytochrome P450 Family 1 Subfamily A Member 1	Protein Coding	48	GC15M074719	0.932010889
IFNG	Interferon Gamma	Protein Coding	48	GC12M068154	0.932010889
SLC22A2	Solute Carrier Family 22 Member 2	Protein Coding	45	GC06M160173	0.932010889
ASCL1	Achaete-Scute Family BHLH Transcription Factor 1	Protein Coding	45	GC12P102957	0.931890011
KIF5A	Kinesin Family Member 5A	Protein Coding	45	GC12P057549	0.931890011
PTCH1	Patched 1	Protein Coding	51	GC09M095442	0.931890011
RNASE2	Ribonuclease A Family Member 2	Protein Coding	38	GC14P021633	0.931890011
ARF1	ADP Ribosylation Factor 1	Protein Coding	48	GC01P228082	0.92994833
MAPT	Microtubule Associated Protein Tau	Protein Coding	51	GC17P045894	0.929196656
MT-CO2	Mitochondrially Encoded Cytochrome C Oxidase II	Protein Coding	34	GCMTP007587	0.926187456
APOH	Apolipoprotein H	Protein Coding	44	GC17M066212	0.926136494
CTSG	Cathepsin G	Protein Coding	45	GC14M024573	0.926136494
EDNRB	Endothelin Receptor Type B	Protein Coding	50	GC13M077895	0.926136494
GPHN	Gephyrin	Protein Coding	48	GC14P066507	0.926136494
GRM3	Glutamate Metabotropic Receptor 3	Protein Coding	47	GC07P086643	0.926136494

GRM5	Glutamate Metabotropic Receptor 5	Protein Coding	48	GC11M088504	0.926136494
GSTA1	Glutathione S-Transferase Alpha 1	Protein Coding	43	GC06M052791	0.926136494
IGF2	Insulin Like Growth Factor 2	Protein Coding	48	GC11M002130	0.926136494
MMP2	Matrix Metallopeptidase 2	Protein Coding	54	GC16P055390	0.926136494
MUC5AC	Mucin 5AC, Oligomeric Mucus/Gel-Forming	Protein Coding	39	GC11P001151	0.926136494
PTPN3	Protein Tyrosine Phosphatase Non-Receptor Type 3	Protein Coding	45	GC09M109375	0.926136494
TAS2R14	Taste 2 Receptor Member 14	Protein Coding	36	GC12M010937	0.926136494
RECQL4	RecQ Like Helicase 4	Protein Coding	43	GC08M144512	0.924645901
RENBP	Renin Binding Protein	Protein Coding	41	GC0XM153935	0.924645901
CYP3A7	Cytochrome P450 Family 3 Subfamily A Member 7	Protein Coding	44	GC07M099705	0.924187899
CABIN1	Calcineurin Binding Protein 1	Protein Coding	42	GC22P024011	0.909061432
OSM	Oncostatin M	Protein Coding	43	GC22M030262	0.909061432
PDGFB	Platelet Derived Growth Factor Subunit B	Protein Coding	50	GC22M049027	0.909061432
TRPM2	Transient Receptor Potential Cation Channel Subfamily M Member 2	Protein Coding	43	GC21P044350	0.904589415
CYP20A1	Cytochrome P450 Family 20 Subfamily A Member 1	Protein Coding	37	GC02P203238	0.902686179
ACAA2	Acetyl-CoA Acyltransferase 2	Protein Coding	44	GC18M049782	0.902686179
AGTR2	Angiotensin II Receptor Type 2	Protein Coding	44	GC0XP116170	0.902686179
CD58	CD58 Molecule	Protein Coding	40	GC01M116514	0.902686179

CDC42	Cell Division Cycle 42	Protein Coding	51	GC01P022112	0.902686179
CEL	Carboxyl Ester Lipase	Protein Coding	47	GC09P133061	0.902686179
COX5A	Cytochrome C Oxidase Subunit 5A	Protein Coding	45	GC15M074919	0.902686179
CXADR	CXADR Ig-Like Cell Adhesion Molecule	Protein Coding	44	GC21P017513	0.902686179
FTH1	Ferritin Heavy Chain 1	Protein Coding	51	GC11M061959	0.902686179
HADHA	Hydroxyacyl-CoA Dehydrogenase Trifunctional Multienzyme Complex Subunit Alpha	Protein Coding	47	GC02M026190	0.902686179
KITLG	KIT Ligand	Protein Coding	45	GC12M088492	0.902686179
MDK	Midkine	Protein Coding	44	GC11P046380	0.902686179
MMP8	Matrix Metallopeptidase 8	Protein Coding	48	GC11M102617	0.902686179
NF1	Neurofibromin 1	Protein Coding	50	GC17P031094	0.902686179
PDX1	Pancreatic And Duodenal Homeobox 1	Protein Coding	48	GC13P027921	0.902686179
PLA2G7	Phospholipase A2 Group VII	Protein Coding	50	GC06M047382	0.902686179
PRDX5	Peroxiredoxin 5	Protein Coding	47	GC11P064317	0.902686179
PSG2	Pregnancy Specific Beta-1-Glycoprotein 2	Protein Coding	34	GC19M043064	0.902686179
SLC1A2	Solute Carrier Family 1 Member 2	Protein Coding	50	GC11M035252	0.902686179
TERT	Telomerase Reverse Transcriptase	Protein Coding	52	GC05M001253	0.902686179
THBS2	Thrombospondin 2	Protein Coding	46	GC06M169215	0.902686179
TRDMT1	TRNA Aspartic Acid Methyltransferase 1	Protein Coding	40	GC10M017138	0.902686179
GUCY2D	Guanylate Cyclase 2D, Retinal	Protein Coding	44	GC17P008002	0.89891398
ITGA4	Integrin Subunit Alpha 4	Protein Coding	49	GC02P181456	0.895938575

PYGM	Glycogen Phosphorylase, Muscle Associated	Protein Coding	47	GC11M064746	0.895938575
CASP9	Caspase 9	Protein Coding	48	GC01M015491	0.895791113
HSPA4	Heat Shock Protein Family A (Hsp70) Member 4	Protein Coding	43	GC05P133051	0.895791113
P2RX4	Purinergic Receptor P2X 4	Protein Coding	44	GC12P123577	0.895791113
ADARB1	Adenosine Deaminase RNA Specific B1	Protein Coding	45	GC21P045073	0.892260075
DRD1	Dopamine Receptor D1	Protein Coding	45	GC05M175440	0.892260075
EDN3	Endothelin 3	Protein Coding	47	GC20P059300	0.892260075
STK11	Serine/Threonine Kinase 11	Protein Coding	50	GC19P001177	0.892260075
POLR1C	RNA Polymerase I And III Subunit C	Protein Coding	44	GC06P055413	0.892224073
SELP	Selectin P	Protein Coding	46	GC01M169558	0.892224073
POLL	DNA Polymerase Lambda	Protein Coding	44	GC10M101578	0.889302373
MAG	Myelin Associated Glycoprotein	Protein Coding	46	GC19P035292	0.88782829
EPX	Eosinophil Peroxidase	Protein Coding	44	GC17P058192	0.885750711
MAPK8	Mitogen-Activated Protein Kinase 8	Protein Coding	51	GC10P048306	0.885750711
HSPA1A	Heat Shock Protein Family A (Hsp70) Member 1A	Protein Coding	44	GC06P055219	0.883568645
AGFG1	ArfGAP With FG Repeats 1	Protein Coding	40	GC02P227473	0.878610194
GATA2	GATA Binding Protein 2	Protein Coding	48	GC03M128479	0.878610194
GNAS	GNAS Complex Locus	Protein Coding	51	GC20P058839	0.878610194
OPRD1	Opioid Receptor Delta 1	Protein Coding	45	GC01P028812	0.878610194
RAP1A	RAP1A, Member Of RAS Oncogene Family	Protein Coding	46	GC01P111542	0.878610194
NOS1	Nitric Oxide Synthase 1	Protein Coding	50	GC12M117208	0.874567091
H2AX	H2A.X Variant Histone	Protein Coding	36	GC11M119097	0.874164701

DNMT3B	DNA Methyltransferase 3 Beta	Protein Coding	51	GC20P032762	0.868708313
TRAF3	TNF Receptor Associated Factor 3	Protein Coding	48	GC14P106036	0.866604328
PPARG	Peroxisome Proliferator Activated Receptor Gamma	Protein Coding	53	GC03P012287	0.858617902
NME4	NME/NM23 Nucleoside Diphosphate Kinase 4	Protein Coding	43	GC16P000396	0.854591608
NPR1	Natriuretic Peptide Receptor 1	Protein Coding	47	GC01P153725	0.854591608
NPR2	Natriuretic Peptide Receptor 2	Protein Coding	48	GC09P035782	0.854591608
PTHLH	Parathyroid Hormone Like Hormone	Protein Coding	46	GC12M027959	0.853855669
RAB5A	RAB5A, Member RAS Oncogene Family	Protein Coding	47	GC03P019963	0.853855669
GLYAT	Glycine-N-Acyltransferase	Protein Coding	42	GC11M069098	0.853678763
PRKN	Parkin RBR E3 Ubiquitin Protein Ligase	Protein Coding	40	GC06M161348	0.853678763
CCL3	C-C Motif Chemokine Ligand 3	Protein Coding	40	GC17M036088	0.851740956
FABP12	Fatty Acid Binding Protein 12	Protein Coding	32	GC08M081524	0.851740956
G6PC1	Glucose-6-Phosphatase Catalytic Subunit 1	Protein Coding	36	GC17P044746	0.851740956
CREBBP	CREB Binding Protein	Protein Coding	52	GC16M003745	0.851616204
HLCS	Holocarboxylase Synthetase	Protein Coding	44	GC21M036750	0.851616204
SYP	Synaptophysin	Protein Coding	44	GC0XM049187	0.851616204
TRMT10C	TRNA Methyltransferase 10C, Mitochondrial RNase P Subunit	Protein Coding	36	GC03P101561	0.849644065

FASN	Fatty Acid Synthase	Protein Coding	50	GC17M082078	0.847844601
GJB1	Gap Junction Protein Beta 1	Protein Coding	48	GC0XP071212	0.846731663
OAS1	2'-5'-Oligoadenylate Synthetase 1	Protein Coding	46	GC12P112911	0.846731663
EDNRA	Endothelin Receptor Type A	Protein Coding	50	GC04P147480	0.842924714
SMS	Spermine Synthase	Protein Coding	45	GC0XP021958	0.842924714
NCOA3	Nuclear Receptor Coactivator 3	Protein Coding	47	GC20P047501	0.836863279
ADCY2	Adenylate Cyclase 2	Protein Coding	44	GC05P007396	0.833367586
ADCY4	Adenylate Cyclase 4	Protein Coding	43	GC14M024318	0.833367586
ADCY6	Adenylate Cyclase 6	Protein Coding	48	GC12M048766	0.833367586
ADCY7	Adenylate Cyclase 7	Protein Coding	46	GC16P050412	0.833367586
ADCY9	Adenylate Cyclase 9	Protein Coding	45	GC16M003953	0.833367586
XRCC1	X-Ray Repair Cross Complementing 1	Protein Coding	44	GC19M043543	0.833109796
HSPG2	Heparan Sulfate Proteoglycan 2	Protein Coding	46	GC01M021822	0.831014872
APTX	Aprataxin	Protein Coding	45	GC09M032886	0.829916954
IHH	Indian Hedgehog Signaling Molecule	Protein Coding	48	GC02M219054	0.829916954
UPP2	Uridine Phosphorylase 2	Protein Coding	37	GC02P157876	0.829916954
BAAT	Bile Acid-CoA:Amino Acid N-Acyltransferase	Protein Coding	45	GC09M101354	0.82836163
CD44	CD44 Molecule (Indian Blood Group)	Protein Coding	48	GC11P035139	0.82836163
CHGA	Chromogranin A	Protein Coding	43	GC14P092925	0.82836163
CYP2A6	Cytochrome P450 Family 2 Subfamily A Member 6	Protein Coding	48	GC19M040843	0.82836163
GSN	Gelsolin	Protein Coding	49	GC09P121201	0.82836163
IL11	Interleukin 11	Protein Coding	41	GC19M055364	0.82836163
LPL	Lipoprotein Lipase	Protein Coding	50	GC08P019901	0.82836163

RARB	Retinoic Acid Receptor Beta	Protein Coding	51	GC03P024830	0.82836163
S100A6	S100 Calcium Binding Protein A6	Protein Coding	43	GC01M153535	0.82836163
TFRC	Transferrin Receptor	Protein Coding	49	GC03M196027	0.82836163
VDR	Vitamin D Receptor	Protein Coding	52	GC12M047841	0.82836163
HTR2A	5-Hydroxytryptamine Receptor 2A	Protein Coding	48	GC13M046831	0.824886322
SULT1E1	Sulfotransferase Family 1E Member 1	Protein Coding	45	GC04M069841	0.824886322
TUG1	Taurine Up-Regulated 1	RNA Gene	23	GC22P030969	0.824886322
ACHE	Acetylcholinesterase (Cartwright Blood Group)	Protein Coding	47	GC07M100889	0.824385941
PTEN	Phosphatase And Tensin Homolog	Protein Coding	53	GC10P087863	0.823593378
RPS6KA1	Ribosomal Protein S6 Kinase A1	Protein Coding	51	GC01P026540	0.823593378
RPS27	Ribosomal Protein S27	Protein Coding	43	GC01P153991	0.823172569
UGT1A6	UDP Glucuronosyltransferase Family 1 Member A6	Protein Coding	42	GC02P233691	0.811382651
UGT1A8	UDP Glucuronosyltransferase Family 1 Member A8	Protein Coding	36	GC02P233618	0.811382651
MOCS1	Molybdenum Cofactor Synthesis 1	Protein Coding	41	GC06M039899	0.811080933
GSK3B	Glycogen Synthase Kinase 3 Beta	Protein Coding	51	GC03M119821	0.810970247
CARM1	Coactivator Associated Arginine Methyltransferase 1	Protein Coding	47	GC19P010871	0.807418585
MCF2	MCF.2 Cell Line Derived Transforming Sequence	Protein Coding	41	GC0XM139581	0.807418585

NFKBIA	NFKB Inhibitor Alpha	Protein Coding	51	GC14M035401	0.807418585
SELE	Selectin E	Protein Coding	44	GC01M169722	0.807418585
SLCO1B3	Solute Carrier Organic Anion Transporter Family Member 1B3	Protein Coding	44	GC12P020810	0.807418585
STAT3	Signal Transducer And Activator Of Transcription 3	Protein Coding	53	GC17M042313	0.803522706
YBX1	Y-Box Binding Protein 1	Protein Coding	40	GC01P042682	0.803522706
GNMT	Glycine N-Methyltransferase	Protein Coding	46	GC06P042960	0.802057743
SP3	Sp3 Transcription Factor	Protein Coding	43	GC02M173882	0.802057743
ACP5	Acid Phosphatase 5, Tartrate Resistant	Protein Coding	47	GC19M011574	0.802057743
CYP2C8	Cytochrome P450 Family 2 Subfamily C Member 8	Protein Coding	49	GC10M095038	0.802057743
DDRGK1	DDRGK Domain Containing 1	Protein Coding	38	GC20M003191	0.802057743
GSTA2	Glutathione S-Transferase Alpha 2	Protein Coding	42	GC06M052750	0.802057743
H19	H19 Imprinted Maternally Expressed Transcript	RNA Gene	28	GC11M001995	0.802057743
HLTF	Helicase Like Transcription Factor	Protein Coding	40	GC03M149030	0.802057743
IGFBP3	Insulin Like Growth Factor Binding Protein 3	Protein Coding	45	GC07M045912	0.802057743
MUC2	Mucin 2, Oligomeric Mucus/Gel-Forming	Protein Coding	40	GC11P001074	0.802057743
NOG	Noggin	Protein Coding	47	GC17P056593	0.802057743
PRTN3	Proteinase 3	Protein Coding	45	GC19P000840	0.802057743

RASSF1	Ras Association Domain Family Member 1	Protein Coding	45	GC03M050329	0.802057743
ACTA2	Actin Alpha 2, Smooth Muscle	Protein Coding	49	GC10M088935	0.80154705
PRORP	Protein Only RNase P Catalytic Subunit	Protein Coding	29	GC14P035132	0.80154705
SLC6A4	Solute Carrier Family 6 Member 4	Protein Coding	48	GC17M030194	0.798727155
ACE	Angiotensin I Converting Enzyme	Protein Coding	50	GC17P063477	0.795385599
EDN1	Endothelin 1	Protein Coding	48	GC06P012256	0.795385599
ACADL	Acyl-CoA Dehydrogenase Long Chain	Protein Coding	44	GC02M210187	0.793844879
SRF	Serum Response Factor	Protein Coding	42	GC06P043171	0.793844879
MTRR	5-Methyltetrahydrofolate-Homocysteine Methyltransferase Reductase	Protein Coding	43	GC05P007851	0.790370941
PKD1	Polycystin 1, Transient Receptor Potential Channel Interacting	Protein Coding	46	GC16M003057	0.785256386
GSTT1	Glutathione S-Transferase Theta 1	Protein Coding	33	GC22Mi00270	0.782812417
H1-5	H1.5 Linker Histone, Cluster Member	Protein Coding	33	GC06M047206	0.782016754
AKAP13	A-Kinase Anchoring Protein 13	Protein Coding	44	GC15P085388	0.779855013
PDPK1	3-Phosphoinositide Dependent Protein Kinase 1	Protein Coding	50	GC16P002537	0.779855013
VEGFA	Vascular Endothelial Growth Factor A	Protein Coding	49	GC06P043770	0.779286325

ACSBG1	Acyl-CoA Synthetase Bubblegum Family Member 1	Protein Coding	40	GC15M078167	0.774861395
ACSBG2	Acyl-CoA Synthetase Bubblegum Family Member 2	Protein Coding	37	GC19P006135	0.774861395
ACSL1	Acyl-CoA Synthetase Long Chain Family Member 1	Protein Coding	45	GC04M184755	0.774861395
ACSL3	Acyl-CoA Synthetase Long Chain Family Member 3	Protein Coding	41	GC02P222860	0.774861395
ACSL4	Acyl-CoA Synthetase Long Chain Family Member 4	Protein Coding	45	GC0XM109624	0.774861395
ACSL5	Acyl-CoA Synthetase Long Chain Family Member 5	Protein Coding	43	GC10P112374	0.774861395
ACSL6	Acyl-CoA Synthetase Long Chain Family Member 6	Protein Coding	40	GC05M131949	0.774861395
ACSM1	Acyl-CoA Synthetase Medium Chain Family Member 1	Protein Coding	40	GC16M020634	0.774861395
ACSM2A	Acyl-CoA Synthetase Medium Chain Family Member 2A	Protein Coding	37	GC16P020463	0.774861395
ACSM2B	Acyl-CoA Synthetase Medium Chain Family Member 2B	Protein Coding	36	GC16M020547	0.774861395
ACSM3	Acyl-CoA Synthetase Medium Chain Family Member 3	Protein Coding	38	GC16P020610	0.774861395
ACSM4	Acyl-CoA Synthetase Medium Chain Family Member 4	Protein Coding	33	GC12P007304	0.774861395

ACSM5	Acyl-CoA Synthetase Medium Chain Family Member 5	Protein Coding	39	GC16P020410	0.774861395
ACSM6	Acyl-CoA Synthetase Medium Chain Family Member 6	Protein Coding	31	GC10P095194	0.774861395
NCF2	Neutrophil Cytosolic Factor 2	Protein Coding	48	GC01M183555	0.774861395
SLC27A2	Solute Carrier Family 27 Member 2	Protein Coding	46	GC15P050182	0.774861395
SLC27A5	Solute Carrier Family 27 Member 5	Protein Coding	43	GC19M058479	0.774861395
SOAT1	Sterol O-Acyltransferase 1	Protein Coding	45	GC01P179262	0.774861395
BCL6	BCL6 Transcription Repressor	Protein Coding	45	GC03M187721	0.772875488
CNOT7	CCR4-NOT Transcription Complex Subunit 7	Protein Coding	41	GC08M017224	0.772875488
NAGLU	N-Acetyl-Alpha-Glucosaminidase	Protein Coding	43	GC17P042537	0.772597313
PPARA	Peroxisome Proliferator Activated Receptor Alpha	Protein Coding	47	GC22P046150	0.772597313
NRAS	NRAS Proto-Oncogene, GTPase	Protein Coding	50	GC01M114704	0.76978761
PGM2	Phosphoglucomutase 2	Protein Coding	42	GC04P037865	0.764378965
ADRB2	Adrenoceptor Beta 2	Protein Coding	50	GC05P148825	0.761881053
RPA1	Replication Protein A1	Protein Coding	47	GC17P001829	0.761881053
MYBL1	MYB Proto-Oncogene Like 1	Protein Coding	41	GC08M066562	0.76157105
MYBL2	MYB Proto-Oncogene Like 2	Protein Coding	43	GC20P043667	0.76157105
PON1	Paraoxonase 1	Protein Coding	46	GC07M095297	0.758531153
LRRK2	Leucine Rich Repeat Kinase 2	Protein Coding	50	GC12P040196	0.756756663
NTPCR	Nucleoside-Triphosphatase, Cancer-Related	Protein Coding	38	GC01P232950	0.747587919

ENTPD8	Ectonucleoside Triphosphate Diphosphohydrolase 8	Protein Coding	37	GC09M137434	0.747587919
GUCY2C	Guanylate Cyclase 2C	Protein Coding	45	GC12M014612	0.747587919
NUDT5	Nudix Hydrolase 5	Protein Coding	40	GC10M012165	0.747587919
PAPSS1	3’-Phosphoadenosine 5’-Phosphosulfate Synthase 1	Protein Coding	44	GC04M107590	0.747587919
PAPSS2	3’-Phosphoadenosine 5’-Phosphosulfate Synthase 2	Protein Coding	45	GC10P087659	0.747587919
PDE6D	Phosphodiesterase 6D	Protein Coding	46	GC02M231732	0.747587919
PRUNE1	Prune Exopolyphosphatase 1	Protein Coding	33	GC01P151008	0.747587919
U2AF2	U2 Small Nuclear RNA Auxiliary Factor 2	Protein Coding	38	GC19P055654	0.747089148
KLK3	Kallikrein Related Peptidase 3	Protein Coding	47	GC19P050854	0.746675074
ACOT1	Acyl-CoA Thioesterase 1	Protein Coding	37	GC14P073493	0.746675074
ACOT2	Acyl-CoA Thioesterase 2	Protein Coding	38	GC14P073567	0.746675074
ACOT4	Acyl-CoA Thioesterase 4	Protein Coding	37	GC14P073592	0.746675074
ACOT7	Acyl-CoA Thioesterase 7	Protein Coding	41	GC01M006265	0.746675074
ACOT8	Acyl-CoA Thioesterase 8	Protein Coding	39	GC20M045841	0.746675074
ADAT1	Adenosine Deaminase TRNA Specific 1	Protein Coding	38	GC16M075596	0.746675074
AGPAT1	1-Acylglycerol-3-Phosphate O-Acyltransferase 1	Protein Coding	43	GC06M032168	0.746675074
AGPAT2	1-Acylglycerol-3-Phosphate O-Acyltransferase 2	Protein Coding	47	GC09M136673	0.746675074

AGPAT3	1-Acylglycerol-3-Phosphate O-Acyltransferase 3	Protein Coding	41	GC21P043865	0.746675074
AGPAT4	1-Acylglycerol-3-Phosphate O-Acyltransferase 4	Protein Coding	41	GC06M161129	0.746675074
AGPAT5	1-Acylglycerol-3-Phosphate O-Acyltransferase 5	Protein Coding	42	GC08P006708	0.746675074
AWAT1	Acyl-CoA Wax Alcohol Acyltransferase 1	Protein Coding	29	GC0XP070234	0.746675074
AWAT2	Acyl-CoA Wax Alcohol Acyltransferase 2	Protein Coding	33	GC0XM070040	0.746675074
BLZF1	Basic Leucine Zipper Nuclear Factor 1	Protein Coding	40	GC01P169367	0.746675074
CASP1	Caspase 1	Protein Coding	51	GC11M105025	0.746675074
DBI	Diazepam Binding Inhibitor, Acyl-CoA Binding Protein	Protein Coding	47	GC02P119366	0.746675074
DBT	Dihydrolipoamide Branched Chain Transacylase E2	Protein Coding	43	GC01M100186	0.746675074
DGAT1	Diacylglycerol O-Acyltransferase 1	Protein Coding	47	GC08M144316	0.746675074
DGAT2	Diacylglycerol O-Acyltransferase 2	Protein Coding	44	GC11P075759	0.746675074
DNM3	Dynamin 3	Protein Coding	44	GC01P171841	0.746675074
EPHA3	EPH Receptor A3	Protein Coding	47	GC03P089077	0.746675074
FAR1	Fatty Acyl-CoA Reductase 1	Protein Coding	43	GC11P013668	0.746675074
FAR2	Fatty Acyl-CoA Reductase 2	Protein Coding	40	GC12P029145	0.746675074
GLYATL1	Glycine-N-Acyltransferase Like 1	Protein Coding	36	GC11P058906	0.746675074
GLYATL2	Glycine-N-Acyltransferase Like 2	Protein Coding	34	GC11M069100	0.746675074

GNPAT	Glyceronephosphate O-Acyltransferase	Protein Coding	46	GC01P231241	0.746675074
GPAM	Glycerol-3-Phosphate Acyltransferase, Mitochondrial	Protein Coding	43	GC10M112148	0.746675074
GPAT2	Glycerol-3-Phosphate Acyltransferase 2, Mitochondrial	Protein Coding	33	GC02M096216	0.746675074
GPAT3	Glycerol-3-Phosphate Acyltransferase 3	Protein Coding	34	GC04P083536	0.746675074
GPAT4	Glycerol-3-Phosphate Acyltransferase 4	Protein Coding	33	GC08P041577	0.746675074
LCLAT1	Lysocardiolipin Acyltransferase 1	Protein Coding	38	GC02P030447	0.746675074
LPCAT1	Lysophosphatidylcholine Acyltransferase 1	Protein Coding	37	GC05M001456	0.746675074
LPCAT3	Lysophosphatidylcholine Acyltransferase 3	Protein Coding	36	GC12M006976	0.746675074
LTA	Lymphotoxin Alpha	Protein Coding	43	GC06P055200	0.746675074
MOGAT1	Monoacylglycerol O-Acyltransferase 1	Protein Coding	33	GC02P222671	0.746675074
MOGAT2	Monoacylglycerol O-Acyltransferase 2	Protein Coding	38	GC11P075717	0.746675074
MOGAT3	Monoacylglycerol O-Acyltransferase 3	Protein Coding	33	GC07M101192	0.746675074
MX1	MX Dynamin Like GTPase 1	Protein Coding	43	GC21P041420	0.746675074
PAK1	P21 (RAC1) Activated Kinase 1	Protein Coding	49	GC11M077321	0.746675074
PITX2	Paired Like Homeodomain 2	Protein Coding	48	GC04M110617	0.746675074

RAN	RAN, Member RAS Oncogene Family	Protein Coding	45	GC12P130871	0.746675074
SCD	Stearoyl-CoA Desaturase	Protein Coding	49	GC10P100347	0.746675074
SIGMAR1	Sigma Non-Opioid Intracellular Receptor 1	Protein Coding	48	GC09M034634	0.746675074
SOAT2	Sterol O-Acyltransferase 2	Protein Coding	44	GC12P053103	0.746675074
SUCLA2	Succinate-CoA Ligase ADP-Forming Subunit Beta	Protein Coding	47	GC13M047745	0.746675074
SUCLG1	Succinate-CoA Ligase GDP/ADP-Forming Subunit Alpha	Protein Coding	46	GC02M084423	0.746675074
SLC6A1	Solute Carrier Family 6 Member 1	Protein Coding	48	GC03P011049	0.746644974
SAFB	Scaffold Attachment Factor B	Protein Coding	40	GC19P005623	0.746533394
CHEK2	Checkpoint Kinase 2	Protein Coding	54	GC22M028687	0.744612575
CNTN2	Contactin 2	Protein Coding	46	GC01P205043	0.744612575
LIG1	DNA Ligase 1	Protein Coding	47	GC19M048115	0.744612575
RPS27A	Ribosomal Protein S27a	Protein Coding	44	GC02P055231	0.744612575
TPI1	Triosephosphate Isomerase 1	Protein Coding	48	GC12P011857	0.744612575
TRIM22	Tripartite Motif Containing 22	Protein Coding	40	GC11P005689	0.744224072
CNOT1	CCR4-NOT Transcription Complex Subunit 1	Protein Coding	41	GC16M058519	0.738865733
MANF	Mesencephalic Astrocyte Derived Neurotrophic Factor	Protein Coding	40	GC03P051385	0.738865733
AURKA	Aurora Kinase A	Protein Coding	52	GC20M056370	0.735569358
MERTK	MER Proto-Oncogene, Tyrosine Kinase	Protein Coding	51	GC02P111898	0.735569358
RPS6KB1	Ribosomal Protein S6 Kinase B1	Protein Coding	50	GC17P059893	0.735532641

ENSG00000284762		Protein Coding	5	GC05P077092	0.733338416
HADH	Hydroxyacyl-CoA Dehydrogenase	Protein Coding	47	GC04P107989	0.728274941
CPS1	Carbamoyl-Phosphate Synthase 1	Protein Coding	46	GC02P210477	0.728085101
H4-16	H4 Histone 16	Protein Coding	34	GC12M015752	0.728085101
IGF1R	Insulin Like Growth Factor 1 Receptor	Protein Coding	55	GC15P098648	0.728085101
RNPS1	RNA Binding Protein With Serine Rich Domain 1	Protein Coding	38	GC16M002253	0.727542162
CCNH	Cyclin H	Protein Coding	47	GC05M087311	0.72519058
RPS17	Ribosomal Protein S17	Protein Coding	43	GC15M082536	0.72308588
CSF1R	Colony Stimulating Factor 1 Receptor	Protein Coding	52	GC05M150053	0.721273184
DAO	D-Amino Acid Oxidase	Protein Coding	45	GC12P108859	0.721273184
PTGIR	Prostaglandin I2 Receptor	Protein Coding	48	GC19M047072	0.721273184
MSH3	MutS Homolog 3	Protein Coding	44	GC05P080654	0.717555523
PSMA6	Proteasome 20S Subunit Alpha 6	Protein Coding	47	GC14P035278	0.717555523
RPS3	Ribosomal Protein S3	Protein Coding	45	GC11P076260	0.717555523
ACTG1	Actin Gamma 1	Protein Coding	50	GC17M081509	0.717382252
CCN6	Cellular Communication Network Factor 6	Protein Coding	33	GC06P112053	0.717382252
DNMT3L	DNA Methyltransferase 3 Like	Protein Coding	43	GC21M044246	0.717382252
ECI2	Enoyl-CoA Delta Isomerase 2	Protein Coding	41	GC06M004115	0.717382252
EFS	Embryonal Fyn-Associated Substrate	Protein Coding	36	GC14M023356	0.717382252
EHHADH	Enoyl-CoA Hydratase And 3-Hydroxyacyl CoA Dehydrogenase	Protein Coding	45	GC03M185190	0.717382252
GP6	Glycoprotein VI Platelet	Protein Coding	45	GC19M055013	0.717382252

GYPA	Glycophorin A (MNS Blood Group)	Protein Coding	45	GC04M144109	0.717382252
PLEK	Pleckstrin	Protein Coding	40	GC02P068365	0.717382252
RAC2	Rac Family Small GTPase 2	Protein Coding	51	GC22M037227	0.717382252
SUCLG2	Succinate-CoA Ligase GDP-Forming Subunit Beta	Protein Coding	43	GC03M067358	0.717382252
TOLLIP	Toll Interacting Protein	Protein Coding	44	GC11M001274	0.717382252
TSC1	TSC Complex Subunit 1	Protein Coding	48	GC09M132891	0.717382252
CNP	2',3'-Cyclic Nucleotide 3' Phosphodiesterase	Protein Coding	44	GC17P041966	0.715552568
GAL	Galanin And GMAP Prepropeptide	Protein Coding	45	GC11P068684	0.715552568
STAT5A	Signal Transducer And Activator Of Transcription 5A	Protein Coding	47	GC17P042287	0.715552568
CCND2	Cyclin D2	Protein Coding	50	GC12P011795	0.703041494
ARF6	ADP Ribosylation Factor 6	Protein Coding	45	GC14P049895	0.700414896
FLNA	Filamin A	Protein Coding	50	GC0XM154348	0.700414896
NCOA1	Nuclear Receptor Coactivator 1	Protein Coding	45	GC02P024492	0.700414896
SHBG	Sex Hormone Binding Globulin	Protein Coding	41	GC17P007613	0.700414896
NTF4	Neurotrophin 4	Protein Coding	44	GC19M049098	0.694543362
SLC7A1	Solute Carrier Family 7 Member 1	Protein Coding	44	GC13M029509	0.694543362
SPARC	Secreted Protein Acidic And Cysteine Rich	Protein Coding	51	GC05M151661	0.694543362
CEBPB	CCAAT Enhancer Binding Protein Beta	Protein Coding	44	GC20P050190	0.694075346
FOS	Fos Proto-Oncogene, AP-1 Transcription Factor Subunit	Protein Coding	51	GC14P075278	0.694075346
HSPA5	Heat Shock Protein Family A (Hsp70) Member 5	Protein Coding	48	GC09M125234	0.694075346

HMGCR	3-Hydroxy-3-Methylglutaryl-CoA Reductase	Protein Coding	47	GC05P075336	0.692743838
DMD	Dystrophin	Protein Coding	47	GC0XM031097	0.692601681
CACNA1C	Calcium Voltage-Gated Channel Subunit Alpha1 C	Protein Coding	50	GC12P001970	0.688700676
AASDH	Aminoadipate-Semialdehyde Dehydrogenase	Protein Coding	36	GC04M056340	0.68684119
ACAD8	Acyl-CoA Dehydrogenase Family Member 8	Protein Coding	45	GC11P134253	0.68684119
ACAD9	Acyl-CoA Dehydrogenase Family Member 9	Protein Coding	44	GC03P131450	0.68684119
ACADS	Acyl-CoA Dehydrogenase Short Chain	Protein Coding	47	GC12P120922	0.68684119
ACADSB	Acyl-CoA Dehydrogenase Short/Branched Chain	Protein Coding	47	GC10P123008	0.68684119
ACOX2	Acyl-CoA Oxidase 2	Protein Coding	44	GC03M058490	0.68684119
ACOXL	Acyl-CoA Oxidase Like	Protein Coding	33	GC02P110732	0.68684119
AIDA	Axin Interactor, Dorsalization Associated	Protein Coding	34	GC01M223071	0.68684119
CYP3A43	Cytochrome P450 Family 3 Subfamily A Member 43	Protein Coding	43	GC07P099829	0.68684119
DROSHA	Drosha Ribonuclease III	Protein Coding	43	GC05M031401	0.68684119
GCDH	Glutaryl-CoA Dehydrogenase	Protein Coding	47	GC19P012891	0.68684119
IVD	Isovaleryl-CoA Dehydrogenase	Protein Coding	46	GC15P040405	0.68684119
PPARD	Peroxisome Proliferator Activated Receptor Delta	Protein Coding	48	GC06P055324	0.68684119
ASS1	Argininosuccinate Synthase 1	Protein Coding	49	GC09P130444	0.683704615
PRKAB1	Protein Kinase AMP-Activated Non-Catalytic Subunit Beta 1	Protein Coding	48	GC12P119632	0.683704615
PXN	Paxillin	Protein Coding	46	GC12M120210	0.681783795

HMOX1	Heme Oxygenase 1	Protein Coding	53	GC22P035380	0.681542814
LCK	LCK Proto-Oncogene, Src Family Tyrosine Kinase	Protein Coding	53	GC01P032251	0.681542814
NTRK1	Neurotrophic Receptor Tyrosine Kinase 1	Protein Coding	50	GC01P156815	0.681542814
NFS1	NFS1 Cysteine Desulfurase	Protein Coding	46	GC20M035668	0.680737913
PTPRC	Protein Tyrosine Phosphatase Receptor Type C	Protein Coding	51	GC01P198607	0.680737913
ADRB3	Adrenoceptor Beta 3	Protein Coding	46	GC08M037962	0.658591926
NMUR1	Neuromedin U Receptor 1	Protein Coding	41	GC02M231666	0.658591926
GLS	Glutaminase	Protein Coding	48	GC02P190880	0.658444405
GRIA2	Glutamate Ionotropic Receptor AMPA Type Subunit 2	Protein Coding	51	GC04P157204	0.654913425
IL1RAPL2	Interleukin 1 Receptor Accessory Protein Like 2	Protein Coding	38	GC0XP104566	0.654913425
NTS	Neurotensin	Protein Coding	41	GC12P085876	0.654913425
SCP2	Sterol Carrier Protein 2	Protein Coding	48	GC01P052927	0.654913425
SKP1	S-Phase Kinase Associated Protein 1	Protein Coding	44	GC05M134148	0.654913425
ADIPOQ	Adiponectin, C1Q And Collagen Domain Containing	Protein Coding	45	GC03P186842	0.654877365
CCR5	C-C Motif Chemokine Receptor 5	Protein Coding	47	GC03P046383	0.654877365
CKB	Creatine Kinase B	Protein Coding	46	GC14M103519	0.654877365
CYP1B1	Cytochrome P450 Family 1 Subfamily B Member 1	Protein Coding	49	GC02M038066	0.654877365
ELANE	Elastase, Neutrophil Expressed	Protein Coding	47	GC19P000855	0.654877365

FMO3	Flavin Containing Dimethylaniline Monoxygenase 3	Protein Coding	47	GC01P171090	0.654877365
HP	Haptoglobin	Protein Coding	45	GC16P072089	0.654877365
HRH2	Histamine Receptor H2	Protein Coding	45	GC05P175659	0.654877365
LIPC	Lipase C, Hepatic Type	Protein Coding	46	GC15P058410	0.654877365
MMP1	Matrix Metallopeptidase 1	Protein Coding	52	GC11M102810	0.654877365
PRDM2	PR/SET Domain 2	Protein Coding	41	GC01P013755	0.654877365
SLC27A1	Solute Carrier Family 27 Member 1	Protein Coding	41	GC19P026639	0.654877365
SLC6A14	Solute Carrier Family 6 Member 14	Protein Coding	41	GC0XP116436	0.654877365
TAC1	Tachykinin Precursor 1	Protein Coding	44	GC07P097731	0.654877365
GCKR	Glucokinase Regulator	Protein Coding	42	GC02P027496	0.653448343
HBG2	Hemoglobin Subunit Gamma 2	Protein Coding	43	GC11M005442	0.653448343
APAF1	Apoptotic Peptidase Activating Factor 1	Protein Coding	47	GC12P098645	0.651527524
ASH1L	ASH1 Like Histone Lysine Methyltransferase	Protein Coding	41	GC01M155335	0.651527524
KAT2B	Lysine Acetyltransferase 2B	Protein Coding	48	GC03P020043	0.651527524
KAT5	Lysine Acetyltransferase 5	Protein Coding	47	GC11P065711	0.651527524
PRMT6	Protein Arginine Methyltransferase 6	Protein Coding	40	GC01P107056	0.651527524
RETN	Resistin	Protein Coding	44	GC19P007669	0.651527524
ACO1	Aconitase 1	Protein Coding	45	GC09P032374	0.649752975
CDC6	Cell Division Cycle 6	Protein Coding	46	GC17P040287	0.649752975
JAK2	Janus Kinase 2	Protein Coding	54	GC09P004985	0.649752975
KRT8	Keratin 8	Protein Coding	48	GC12M052897	0.649752975
PTK2	Protein Tyrosine Kinase 2	Protein Coding	48	GC08M140657	0.649752975

ACLY	ATP Citrate Lyase	Protein Coding	47	GC17M041866	0.648404062
IL1A	Interleukin 1 Alpha	Protein Coding	44	GC02M112773	0.648404062
PLA2G4A	Phospholipase A2 Group IVA	Protein Coding	50	GC01P186798	0.648404062
TERC	Telomerase RNA Component	RNA Gene	29	GC03M169765	0.648404062
ENTPD4	Ectonucleoside Triphosphate Diphosphohydrolase 4	Protein Coding	37	GC08M023385	0.64750123
ENTPD5	Ectonucleoside Triphosphate Diphosphohydrolase 5 (Inactive)	Protein Coding	44	GC14M073958	0.64750123
ENSG00000264545		Protein Coding	10	GC09P021802	0.639641285
MACROD1	Mono-ADP Ribosylhydrolase 1	Protein Coding	34	GC11M063998	0.639641285
MACROD2	Mono-ADP Ribosylhydrolase 2	Protein Coding	36	GC20P013925	0.639641285
TRMT10A	TRNA Methyltransferase 10A	Protein Coding	37	GC04M099546	0.638310969
TRMT10B	TRNA Methyltransferase 10B	Protein Coding	30	GC09P037828	0.638310969
BAIAP2L1	BAR/IMD Domain Containing Adaptor Protein 2 Like 1	Protein Coding	40	GC07M098294	0.637220442
DDO	D-Aspartate Oxidase	Protein Coding	40	GC06M110391	0.637220442
DDX41	DEAD-Box Helicase 41	Protein Coding	44	GC05M177511	0.629772902
HSP90B1	Heat Shock Protein 90 Beta Family Member 1	Protein Coding	47	GC12P103930	0.629772902
IP6K1	Inositol Hexakisphosphate Kinase 1	Protein Coding	41	GC03M050062	0.629772902
PFKFB4	6-Phosphofructo-2-Kinase/Fructose-2,6-Biphosphatase 4	Protein Coding	42	GC03M048517	0.629772902
RBKS	Ribokinase	Protein Coding	41	GC02M027781	0.629772902

SLC25A3	Solute Carrier Family 25 Member 3	Protein Coding	45	GC12P098593	0.629772902
WARS1	Tryptophanyl-TRNA Synthetase 1	Protein Coding	38	GC14M100334	0.629772902
BDNF	Brain Derived Neurotrophic Factor	Protein Coding	48	GC11M027654	0.628528953
CACNA1B	Calcium Voltage-Gated Channel Subunit Alpha1 B	Protein Coding	50	GC09P137877	0.628528953
CSNK2A2	Casein Kinase 2 Alpha 2	Protein Coding	48	GC16M058157	0.628528953
EEF2K	Eukaryotic Elongation Factor 2 Kinase	Protein Coding	47	GC16P022217	0.628528953
BCL2L11	BCL2 Like 11	Protein Coding	45	GC02P111119	0.626019418
AANAT	Aralkylamine N-Acetyltransferase	Protein Coding	40	GC17P076453	0.621271253
ACAD10	Acyl-CoA Dehydrogenase Family Member 10	Protein Coding	39	GC12P111686	0.621271253
ACAD11	Acyl-CoA Dehydrogenase Family Member 11	Protein Coding	38	GC03M132559	0.621271253
ACBD3	Acyl-CoA Binding Domain Containing 3	Protein Coding	39	GC01M226144	0.621271253
ACBD4	Acyl-CoA Binding Domain Containing 4	Protein Coding	38	GC17P045132	0.621271253
ACBD5	Acyl-CoA Binding Domain Containing 5	Protein Coding	40	GC10M027182	0.621271253
ACBD6	Acyl-CoA Binding Domain Containing 6	Protein Coding	37	GC01M181947	0.621271253
ACBD7	Acyl-CoA Binding Domain Containing 7	Protein Coding	34	GC10M015114	0.621271253
ACOT11	Acyl-CoA Thioesterase 11	Protein Coding	40	GC01P054542	0.621271253
ACOT9	Acyl-CoA Thioesterase 9	Protein Coding	38	GC0XM023701	0.621271253
ACSF2	Acyl-CoA Synthetase Family Member 2	Protein Coding	38	GC17P050426	0.621271253

ACSF3	Acyl-CoA Synthetase Family Member 3	Protein Coding	43	GC16P089088	0.621271253
AMBP	Alpha-1-Microglobulin/Bikunin Precursor	Protein Coding	43	GC09M114060	0.621271253
APOA1	Apolipoprotein A1	Protein Coding	49	GC11M116835	0.621271253
AVP	Arginine Vasopressin	Protein Coding	47	GC20M003082	0.621271253
BCKDHA	Branched Chain Keto Acid Dehydrogenase E1 Subunit Alpha	Protein Coding	45	GC19P041400	0.621271253
BCKDHB	Branched Chain Keto Acid Dehydrogenase E1 Subunit Beta	Protein Coding	44	GC06P080106	0.621271253
BRD1	Bromodomain Containing 1	Protein Coding	41	GC22M049773	0.621271253
CKMT1A	Creatine Kinase, Mitochondrial 1A	Protein Coding	38	GC15P043693	0.621271253
CKMT2	Creatine Kinase, Mitochondrial 2	Protein Coding	44	GC05P081232	0.621271253
CYP2C18	Cytochrome P450 Family 2 Subfamily C Member 18	Protein Coding	45	GC10P094684	0.621271253
DGAT2L6	Diacylglycerol O-Acyltransferase 2 Like 6	Protein Coding	30	GC0XP070177	0.621271253
ECHS1	Enoyl-CoA Hydratase, Short Chain 1	Protein Coding	48	GC10M133362	0.621271253
FABP2	Fatty Acid Binding Protein 2	Protein Coding	43	GC04M119317	0.621271253
FABP3	Fatty Acid Binding Protein 3	Protein Coding	44	GC01M031365	0.621271253
FPR1	Formyl Peptide Receptor 1	Protein Coding	48	GC19M051745	0.621271253
HSD17B4	Hydroxysteroid 17-Beta Dehydrogenase 4	Protein Coding	47	GC05P119452	0.621271253
IL13	Interleukin 13	Protein Coding	45	GC05P132656	0.621271253
LPCAT4	Lysophosphatidylcholine Acyltransferase 4	Protein Coding	38	GC15M034358	0.621271253

MECR	Mitochondrial Trans-2-Enoyl-CoA Reductase	Protein Coding	44	GC01M029192	0.621271253
PGK2	Phosphoglycerate Kinase 2	Protein Coding	42	GC06M049785	0.621271253
SLC27A3	Solute Carrier Family 27 Member 3	Protein Coding	40	GC01P153786	0.621271253
SLC27A4	Solute Carrier Family 27 Member 4	Protein Coding	47	GC09P128340	0.621271253
TRMO	TRNA Methyltransferase 0	Protein Coding	29	GC09M097896	0.621271253
TLR8	Toll Like Receptor 8	Protein Coding	48	GC0XP012924	0.618763983
NR5A1	Nuclear Receptor Subfamily 5 Group A Member 1	Protein Coding	51	GC09M124481	0.618186891
RPL15	Ribosomal Protein L15	Protein Coding	45	GC03P023916	0.616082191
RPL18	Ribosomal Protein L18	Protein Coding	45	GC19M048615	0.616082191
RPL26	Ribosomal Protein L26	Protein Coding	43	GC17M008377	0.616082191
RPL27	Ribosomal Protein L27	Protein Coding	43	GC17P042998	0.616082191
RPL31	Ribosomal Protein L31	Protein Coding	43	GC02P100985	0.616082191
RPL35	Ribosomal Protein L35	Protein Coding	45	GC09M124857	0.616082191
RPL35A	Ribosomal Protein L35a	Protein Coding	45	GC03P197949	0.616082191
RPL5	Ribosomal Protein L5	Protein Coding	48	GC01P092832	0.616082191
RPS10	Ribosomal Protein S10	Protein Coding	45	GC06M047017	0.616082191
RPS15A	Ribosomal Protein S15a	Protein Coding	43	GC16M018781	0.616082191
RPS19	Ribosomal Protein S19	Protein Coding	49	GC19P041859	0.616082191
RPS24	Ribosomal Protein S24	Protein Coding	44	GC10P078033	0.616082191
RPS26	Ribosomal Protein S26	Protein Coding	43	GC12P056043	0.616082191
RPS28	Ribosomal Protein S28	Protein Coding	40	GC19P008332	0.616082191
RPS29	Ribosomal Protein S29	Protein Coding	42	GC14M049570	0.616082191
RPS7	Ribosomal Protein S7	Protein Coding	43	GC02P003575	0.616082191

TSR2	TSR2 Ribosome Maturation Factor	Protein Coding	37	GC0XP054441	0.616082191
CCK	Cholecystokinin	Protein Coding	43	GC03M042274	0.614394307
CPT2	Carnitine Palmitoyltransferase 2	Protein Coding	50	GC01P053196	0.614394307
FZD4	Frizzled Class Receptor 4	Protein Coding	51	GC11M086945	0.614394307
TRA	T Cell Receptor Alpha Locus	Protein Coding	18	GC14P021621	0.614394307
ATR	ATR Serine/Threonine Kinase	Protein Coding	52	GC03M142449	0.614269555
CALCA	Calcitonin Related Polypeptide Alpha	Protein Coding	43	GC11M014945	0.614269555
CYP19A1	Cytochrome P450 Family 19 Subfamily A Member 1	Protein Coding	50	GC15M051208	0.614269555
FGFR3	Fibroblast Growth Factor Receptor 3	Protein Coding	55	GC04P001795	0.614269555
FLT3	Fms Related Receptor Tyrosine Kinase 3	Protein Coding	53	GC13M028003	0.614269555
GCG	Glucagon	Protein Coding	41	GC02M162142	0.614269555
KYAT1	Kynurenine Aminotransferase 1	Protein Coding	34	GC09M128833	0.614269555
QDPR	Quinoid Dihydropteridine Reductase	Protein Coding	49	GC04M017460	0.614269555
SLC22A3	Solute Carrier Family 22 Member 3	Protein Coding	45	GC06P160348	0.614269555
TFAM	Transcription Factor A, Mitochondrial	Protein Coding	45	GC10P058385	0.614269555
WRN	WRN RecQ Like Helicase	Protein Coding	45	GC08P031033	0.614269555
IMMT	Inner Membrane Mitochondrial Protein	Protein Coding	40	GC02M086144	0.597958624
SNU13	Small Nuclear Ribonucleoprotein 13	Protein Coding	34	GC22M048542	0.5977633

AIFM1	Apoptosis Inducing Factor Mitochondria Associated 1	Protein Coding	50	GC0XM130129	0.596037805
MAPK9	Mitogen-Activated Protein Kinase 9	Protein Coding	49	GC05M180234	0.596037805
ATP1A1	ATPase Na+/K+ Transporting Subunit Alpha 1	Protein Coding	51	GC01P116372	0.595763147
ATP6V0A2	ATPase H+ Transporting V0 Subunit A2	Protein Coding	45	GC12P123712	0.595763147
CSNK1A1	Casein Kinase 1 Alpha 1	Protein Coding	50	GC05M149492	0.595763147
FBP2	Fructose-Bisphosphatase 2	Protein Coding	44	GC09M094558	0.595763147
FGF7	Fibroblast Growth Factor 7	Protein Coding	43	GC15P049423	0.595763147
FH	Fumarate Hydratase	Protein Coding	46	GC01M241499	0.595763147
GATM	Glycine Amidinotransferase	Protein Coding	46	GC15M045361	0.595763147
HIF1A	Hypoxia Inducible Factor 1 Subunit Alpha	Protein Coding	48	GC14P061695	0.595763147
NCL	Nucleolin	Protein Coding	43	GC02M231453	0.595763147
NPM1	Nucleophosmin 1	Protein Coding	50	GC05P171387	0.595763147
PTGES3	Prostaglandin E Synthase 3	Protein Coding	44	GC12M056667	0.595763147
QARS1	Glutaminyl-TRNA Synthetase 1	Protein Coding	26	GC03M049445	0.595763147
STUB1	STIP1 Homology And U-Box Containing Protein 1	Protein Coding	46	GC16P005516	0.595763147
TCIRG1	T Cell Immune Regulator 1, ATPase H+ Transporting V0 Subunit A3	Protein Coding	45	GC11P068038	0.595763147
TIE1	Tyrosine Kinase With Immunoglobulin Like And EGF Like Domains 1	Protein Coding	43	GC01P043300	0.595763147

TRPV1	Transient Receptor Potential Cation Channel Subfamily V Member 1	Protein Coding	47	GC17M003565	0.595763147
ALDH1L1	Aldehyde Dehydrogenase 1 Family Member L1	Protein Coding	41	GC03M126103	0.592237711
ANKRD1	Ankyrin Repeat Domain 1	Protein Coding	43	GC10M090912	0.587539673
ALDH7A1	Aldehyde Dehydrogenase 7 Family Member A1	Protein Coding	48	GC05M126541	0.585740149
AMACR	Alpha-Methylacyl-CoA Racemase	Protein Coding	46	GC05M033986	0.585740149
BMP7	Bone Morphogenetic Protein 7	Protein Coding	46	GC20M057168	0.585740149
BPHL	Biphenyl Hydrolase Like	Protein Coding	40	GC06P003118	0.585740149
CLNS1A	Chloride Nucleotide-Sensitive Channel 1A	Protein Coding	40	GC11M077515	0.585740149
DOK5	Docking Protein 5	Protein Coding	40	GC20P054476	0.585740149
HLA-B	Major Histocompatibility Complex, Class I, B	Protein Coding	46	GC06M046832	0.585740149
N6AMT1	N-6 Adenine-Specific DNA Methyltransferase 1	Protein Coding	41	GC21M028441	0.585740149
NEU1	Neuraminidase 1	Protein Coding	45	GC06M031857	0.585740149
POLE3	DNA Polymerase Epsilon 3, Accessory Subunit	Protein Coding	42	GC09M113407	0.585740149
HDAC1	Histone Deacetylase 1	Protein Coding	50	GC01P032292	0.581697047
TTK	TTK Protein Kinase	Protein Coding	48	GC06P080003	0.581697047
CDKN2B-AS1	CDKN2B Antisense RNA 1	RNA Gene	22	GC09P021994	0.581378222
CD34	CD34 Molecule	Protein Coding	44	GC01M207880	0.573734224
CD3D	CD3d Molecule	Protein Coding	48	GC11M118338	0.573734224
CD5	CD5 Molecule	Protein Coding	41	GC11P061114	0.573734224

COL11A1	Collagen Type XI Alpha 1 Chain	Protein Coding	43	GC01M102876	0.573734224
DPYD-AS2	DPYD Antisense RNA 2	RNA Gene	12	GC01P097796	0.573734224
GBA	Glucosylceramidase Beta	Protein Coding	48	GC01M155234	0.573734224
HAS1	Hyaluronan Synthase 1	Protein Coding	40	GC19M051714	0.573734224
HNRNPAB	Heterogeneous Nuclear Ribonucleoprotein A/B	Protein Coding	39	GC05P178204	0.573734224
IGHV4-38-2	Immunoglobulin Heavy Variable 4-38-2	Protein Coding	7	GC14U901616	0.573734224
IL2RG	Interleukin 2 Receptor Subunit Gamma	Protein Coding	48	GC0XM071108	0.573734224
IRF4	Interferon Regulatory Factor 4	Protein Coding	44	GC06P000391	0.573734224
LMO2	LIM Domain Only 2	Protein Coding	43	GC11M033858	0.573734224
MAGOH	Mago Homolog, Exon Junction Complex Subunit	Protein Coding	38	GC01M053226	0.573734224
MAGOHB	Mago Homolog B, Exon Junction Complex Subunit	Protein Coding	36	GC12M015694	0.573734224
MIR155	MicroRNA 155	RNA Gene	19	GC21P025560	0.573734224
MIR184	MicroRNA 184	RNA Gene	23	GC15P079209	0.573734224
MIR206	MicroRNA 206	RNA Gene	20	GC06P052144	0.573734224
MIR3681	MicroRNA 3681	RNA Gene	10	GC02P012199	0.573734224
MIR494	MicroRNA 494	RNA Gene	16	GC14P106333	0.573734224
MIR542	MicroRNA 542	RNA Gene	17	GC0XM134638	0.573734224
MIR9-1	MicroRNA 9-1	RNA Gene	20	GC01M156420	0.573734224
MYOM2	Myomesin 2	Protein Coding	38	GC08P002045	0.573734224
PAX5	Paired Box 5	Protein Coding	47	GC09M036828	0.573734224
PLPPR4	Phospholipid Phosphatase Related 4	Protein Coding	31	GC01P099262	0.573734224
POP4	POP4 Homolog, Ribonuclease P/MRP Subunit	Protein Coding	40	GC19P029604	0.573734224
SAMD12	Sterile Alpha Motif Domain Containing 12	Protein Coding	37	GC08M118131	0.573734224
SDC1	Syndecan 1	Protein Coding	44	GC02M020200	0.573734224
TREX1	Three Prime Repair Exonuclease 1	Protein Coding	43	GC03P048466	0.573734224

UBTD1	Ubiquitin Domain Containing 1	Protein Coding	34	GC10P097498	0.573734224
VKORC1	Vitamin K Epoxide Reductase Complex Subunit 1	Protein Coding	47	GC16M031116	0.573734224
ABCC2	ATP Binding Cassette Subfamily C Member 2	Protein Coding	48	GC10P099782	0.570071936
ACSS2	Acyl-CoA Synthetase Short Chain Family Member 2	Protein Coding	44	GC20P034873	0.570071936
COASY	Coenzyme A Synthase	Protein Coding	46	GC17P042561	0.570071936
CRKL	CRK Like Proto-Oncogene, Adaptor Protein	Protein Coding	47	GC22P020917	0.570071936
CTSD	Cathepsin D	Protein Coding	52	GC11M001752	0.570071936
IL12B	Interleukin 12B	Protein Coding	44	GC05M159314	0.570071936
PLAUR	Plasminogen Activator, Urokinase Receptor	Protein Coding	45	GC19M043646	0.570071936
TIAM1	TIAM Rac1 Associated GEF 1	Protein Coding	45	GC21M031118	0.570071936
VCAM1	Vascular Cell Adhesion Molecule 1	Protein Coding	46	GC01P100719	0.570071936
NNMT	Nicotinamide N-Methyltransferase	Protein Coding	46	GC11P114257	0.565506876
PRDM9	PR/SET Domain 9	Protein Coding	39	GC05P023443	0.565506876
STAT1	Signal Transducer And Activator Of Transcription 1	Protein Coding	54	GC02M190908	0.565506876
ABCC6	ATP Binding Cassette Subfamily C Member 6	Protein Coding	47	GC16M016148	0.551440775
ATF4	Activating Transcription Factor 4	Protein Coding	47	GC22P039547	0.551440775
ATP8A1	ATPase Phospholipid Transporting 8A1	Protein Coding	43	GC04M042410	0.551440775
CASP2	Caspase 2	Protein Coding	50	GC07P145813	0.551440775

CNGA3	Cyclic Nucleotide Gated Channel Subunit Alpha 3	Protein Coding	44	GC02P098329	0.551440775
CTTN	Cortactin	Protein Coding	44	GC11P070398	0.551440775
DARS1	Aspartyl-TRNA Synthetase 1	Protein Coding	37	GC02M135905	0.551440775
DRD4	Dopamine Receptor D4	Protein Coding	47	GC11P000979	0.551440775
EIF4A1	Eukaryotic Translation Initiation Factor 4A1	Protein Coding	45	GC17P007572	0.551440775
ENPP2	Ectonucleotide Pyrophosphatase/Phosphodiesterase 2	Protein Coding	44	GC08M119556	0.551440775
HDAC6	Histone Deacetylase 6	Protein Coding	52	GC0XP048801	0.551440775
KRT18	Keratin 18	Protein Coding	49	GC12P052948	0.551440775
LDHA	Lactate Dehydrogenase A	Protein Coding	52	GC11P018394	0.551440775
MAT2A	Methionine Adenosyltransferase 2A	Protein Coding	48	GC02P085538	0.551440775
NMNAT1	Nicotinamide Nucleotide Adenylyltransferase 1	Protein Coding	49	GC01P009944	0.551440775
PAK4	P21 (RAC1) Activated Kinase 4	Protein Coding	49	GC19P039125	0.551440775
PCCA	Propionyl-CoA Carboxylase Subunit Alpha	Protein Coding	48	GC13P100089	0.551440775
PPIA	Peptidylprolyl Isomerase A	Protein Coding	48	GC07P044807	0.551440775
SNRNP200	Small Nuclear Ribonucleoprotein U5 Subunit 200	Protein Coding	42	GC02M096363	0.551440775
TRNT1	TRNA Nucleotidyl Transferase 1	Protein Coding	42	GC03P003126	0.551440775
ADH6	Alcohol Dehydrogenase 6 (Class V)	Protein Coding	41	GC04M099202	0.547909737
BDKRB2	Bradykinin Receptor B2	Protein Coding	45	GC14P096205	0.547909737
CHRM1	Cholinergic Receptor Muscarinic 1	Protein Coding	47	GC11M069277	0.547909737

CSNK1E	Casein Kinase 1 Epsilon	Protein Coding	48	GC22M048647	0.547909737
GALT	Galactose-1-Phosphate Uridylyltransferase	Protein Coding	48	GC09P035875	0.547909737
GNAI1	G Protein Subunit Alpha I1	Protein Coding	48	GC07P079769	0.547909737
HTR2C	5-Hydroxytryptamine Receptor 2C	Protein Coding	48	GC0XP114584	0.547909737
ITPR1	Inositol 1,4,5-Trisphosphate Receptor Type 1	Protein Coding	49	GC03P004486	0.547909737
KRIT1	KRIT1 Ankyrin Repeat Containing	Protein Coding	43	GC07M092198	0.547909737
LST1	Leukocyte Specific Transcript 1	Protein Coding	36	GC06P055203	0.547909737
LTB	Lymphotoxin Beta	Protein Coding	40	GC06M046882	0.547909737
METTL3	Methyltransferase Like 3	Protein Coding	40	GC14M021498	0.547909737
OXA1L	OXA1L Mitochondrial Inner Membrane Protein	Protein Coding	40	GC14P022766	0.547909737
PLCB1	Phospholipase C Beta 1	Protein Coding	49	GC20P008061	0.547909737
PPARGC1A	PPARG Coactivator 1 Alpha	Protein Coding	47	GC04M023755	0.547909737
SAG	S-Antigen Visual Arrestin	Protein Coding	45	GC02P233328	0.547909737
TMPO	Thymopoietin	Protein Coding	45	GC12P098515	0.547909737
ABCA4	ATP Binding Cassette Subfamily A Member 4	Protein Coding	45	GC01M093992	0.542749286
BMPR2	Bone Morphogenetic Protein Receptor Type 2	Protein Coding	50	GC02P202376	0.542749286
BUB1B	BUB1 Mitotic Checkpoint Serine/Threonine Kinase B	Protein Coding	50	GC15P040161	0.542749286
CDC37	Cell Division Cycle 37, HSP90 Cochaperone	Protein Coding	42	GC19M010391	0.542749286
CDH2	Cadherin 2	Protein Coding	53	GC18M027950	0.542749286

DDX3X	DEAD-Box Helicase 3 X-Linked	Protein Coding	48	GC0XP041333	0.542749286
EPHB4	EPH Receptor B4	Protein Coding	52	GC07M100803	0.542749286
FGFR1	Fibroblast Growth Factor Receptor 1	Protein Coding	55	GC08M038400	0.542749286
KCNH2	Potassium Voltage-Gated Channel Subfamily H Member 2	Protein Coding	50	GC07M150944	0.542749286
KCNJ5	Potassium Inwardly Rectifying Channel Subfamily J Member 5	Protein Coding	48	GC11P128891	0.542749286
MITF	Melanocyte Inducing Transcription Factor	Protein Coding	48	GC03P069788	0.542749286
PPA1	Inorganic Pyrophosphatase 1	Protein Coding	41	GC10M070202	0.542749286
ROCK2	Rho Associated Coiled-Coil Containing Protein Kinase 2	Protein Coding	48	GC02M011227	0.542749286
RPS6	Ribosomal Protein S6	Protein Coding	44	GC09M019375	0.542749286
RUNX2	RUNX Family Transcription Factor 2	Protein Coding	48	GC06P055427	0.542749286
STIP1	Stress Induced Phosphoprotein 1	Protein Coding	45	GC11P064196	0.542749286
SYVN1	Synoviolin 1	Protein Coding	41	GC11M069375	0.542749286
UBE2C	Ubiquitin Conjugating Enzyme E2 C	Protein Coding	48	GC20P045812	0.542749286
WEE1	WEE1 G2 Checkpoint Kinase	Protein Coding	47	GC11P009573	0.542749286
ADPRM	ADP-Ribose/CDP-Alcohol Diphosphatase, Manganese Dependent	Protein Coding	31	GC17P010697	0.540497541
AK6	Adenylate Kinase 6	Protein Coding	27	GC05M069350	0.540497541

ENTPD6	Ectonucleoside Triphosphate Diphosphohydrolase 6	Protein Coding	43	GC20P025196	0.540497541
NUDT9	Nudix Hydrolase 9	Protein Coding	40	GC04P087422	0.540497541
RDH14	Retinol Dehydrogenase 14	Protein Coding	38	GC02M018555	0.540497541
CCNA1	Cyclin A1	Protein Coding	43	GC13P036431	0.530723274
TP53AIP1	Tumor Protein P53 Regulated Apoptosis Inducing Protein 1	Protein Coding	37	GC11M128934	0.530723274
ARG1	Arginase 1	Protein Coding	50	GC06P131473	0.530216753
ATF2	Activating Transcription Factor 2	Protein Coding	47	GC02M175072	0.530216753
FYN	FYN Proto-Oncogene, Src Family Tyrosine Kinase	Protein Coding	48	GC06M111660	0.530216753
HTR3A	5-Hydroxytryptamine Receptor 3A	Protein Coding	47	GC11P113974	0.530216753
PRKCZ	Protein Kinase C Zeta	Protein Coding	49	GC01P002050	0.530216753
SLC9A1	Solute Carrier Family 9 Member A1	Protein Coding	51	GC01M027109	0.530216753
ACTG2	Actin Gamma 2, Smooth Muscle	Protein Coding	47	GC02P073892	0.52847898
CSNK1G1	Casein Kinase 1 Gamma 1	Protein Coding	44	GC15M064165	0.52847898
CROT	Carnitine O-Octanoyltransferase	Protein Coding	42	GC07P087361	0.521184504
CXCL12	C-X-C Motif Chemokine Ligand 12	Protein Coding	46	GC10M044294	0.521184504
CYSLTR1	Cysteinyl Leukotriene Receptor 1	Protein Coding	44	GC0XM078271	0.521184504
ERCC1	ERCC Excision Repair 1, Endonuclease Non-Catalytic Subunit	Protein Coding	47	GC19M046972	0.521184504
GPX3	Glutathione Peroxidase 3	Protein Coding	44	GC05P150997	0.521184504

KAT6A	Lysine Acetyltransferase 6A	Protein Coding	44	GC08M041929	0.521184504
METTL6	Methyltransferase Like 6	Protein Coding	34	GC03M016942	0.521184504
RBP4	Retinol Binding Protein 4	Protein Coding	45	GC10M093591	0.521184504
RNMT	RNA Guanine-7 Methyltransferase	Protein Coding	43	GC18P014026	0.521184504
SLC23A1	Solute Carrier Family 23 Member 1	Protein Coding	43	GC05M139377	0.520451784
SLC23A2	Solute Carrier Family 23 Member 2	Protein Coding	43	GC20M004852	0.520451784
AASDHPPT	Aminoadipate-Semialdehyde Dehydrogenase-Phosphopantetheinyl Transferase	Protein Coding	40	GC11P106075	0.507265866
ADARB2	Adenosine Deaminase RNA Specific B2 (Inactive)	Protein Coding	40	GC10M001187	0.507265866
ADRA2A	Adrenoceptor Alpha 2A	Protein Coding	47	GC10P111077	0.507265866
ADRB1	Adrenoceptor Beta 1	Protein Coding	49	GC10P114044	0.507265866
ALDH1A3	Aldehyde Dehydrogenase 1 Family Member A3	Protein Coding	46	GC15P100877	0.507265866
ALDH1B1	Aldehyde Dehydrogenase 1 Family Member B1	Protein Coding	45	GC09P038392	0.507265866
ALDH3A2	Aldehyde Dehydrogenase 3 Family Member A2	Protein Coding	45	GC17P019648	0.507265866
ATP2A3	ATPase Sarcoplasmic/Endoplasmic Reticulum Ca2+ Transporting 3	Protein Coding	46	GC17M003923	0.507265866
CAMP	Cathelicidin Antimicrobial Peptide	Protein Coding	42	GC03P048342	0.507265866
CBL	Cbl Proto-Oncogene	Protein Coding	51	GC11P119206	0.507265866
CCKAR	Cholecystokinin A Receptor	Protein Coding	47	GC04M026483	0.507265866

CDC25A	Cell Division Cycle 25A	Protein Coding	48	GC03M048173	0.507265866
CDC7	Cell Division Cycle 7	Protein Coding	45	GC01P091500	0.507265866
CHAT	Choline O-Acetyltransferase	Protein Coding	48	GC10P049609	0.507265866
CHKA	Choline Kinase Alpha	Protein Coding	42	GC11M068052	0.507265866
CHRM2	Cholinergic Receptor Muscarinic 2	Protein Coding	49	GC07P136868	0.507265866
CRK	CRK Proto-Oncogene, Adaptor Protein	Protein Coding	46	GC17M001420	0.507265866
CYP2A13	Cytochrome P450 Family 2 Subfamily A Member 13	Protein Coding	42	GC19P041088	0.507265866
CYP2A7	Cytochrome P450 Family 2 Subfamily A Member 7	Protein Coding	39	GC19M040875	0.507265866
CYP2F1	Cytochrome P450 Family 2 Subfamily F Member 1	Protein Coding	43	GC19P041114	0.507265866
CYP2J2	Cytochrome P450 Family 2 Subfamily J Member 2	Protein Coding	45	GC01M059893	0.507265866
CYP2S1	Cytochrome P450 Family 2 Subfamily S Member 1	Protein Coding	44	GC19P041193	0.507265866
CYP4B1	Cytochrome P450 Family 4 Subfamily B Member 1	Protein Coding	44	GC01P046757	0.507265866
CYP4X1	Cytochrome P450 Family 4 Subfamily X Member 1	Protein Coding	38	GC01P046961	0.507265866
CYP4Z1	Cytochrome P450 Family 4 Subfamily Z Member 1	Protein Coding	36	GC01P047067	0.507265866
DLD	Dihydrolipoamide Dehydrogenase	Protein Coding	51	GC07P107890	0.507265866
DMPK	DM1 Protein Kinase	Protein Coding	49	GC19M045769	0.507265866

ECI1	Enoyl-CoA Delta Isomerase 1	Protein Coding	39	GC16M002239	0.507265866
FGF1	Fibroblast Growth Factor 1	Protein Coding	48	GC05M142555	0.507265866
FKBP1A	FKBP Prolyl Isomerase 1A	Protein Coding	47	GC20M001369	0.507265866
FSHR	Follicle Stimulating Hormone Receptor	Protein Coding	50	GC02M048953	0.507265866
GHR	Growth Hormone Receptor	Protein Coding	47	GC05P042429	0.507265866
GNAQ	G Protein Subunit Alpha Q	Protein Coding	50	GC09M077716	0.507265866
IL1RN	Interleukin 1 Receptor Antagonist	Protein Coding	48	GC02P116694	0.507265866
ITGB2	Integrin Subunit Beta 2	Protein Coding	51	GC21M044885	0.507265866
ITPR3	Inositol 1,4,5-Trisphosphate Receptor Type 3	Protein Coding	47	GC06P033620	0.507265866
LHCGR	Luteinizing Hormone/Choriogonadotropin Receptor	Protein Coding	48	GC02M048686	0.507265866
PLA2G2E	Phospholipase A2 Group IIE	Protein Coding	39	GC01M019920	0.507265866
PLD2	Phospholipase D2	Protein Coding	48	GC17P004808	0.507265866
POLE	DNA Polymerase Epsilon, Catalytic Subunit	Protein Coding	50	GC12M132637	0.507265866
POLE2	DNA Polymerase Epsilon 2, Accessory Subunit	Protein Coding	44	GC14M049643	0.507265866
POLE4	DNA Polymerase Epsilon 4, Accessory Subunit	Protein Coding	37	GC02P074958	0.507265866
RYR3	Ryanodine Receptor 3	Protein Coding	42	GC15P033310	0.507265866
SHC1	SHC Adaptor Protein 1	Protein Coding	46	GC01M154962	0.507265866
SLC24A3	Solute Carrier Family 24 Member 3	Protein Coding	40	GC20P019212	0.507265866
SLC8A1	Solute Carrier Family 8 Member A1	Protein Coding	45	GC02M040078	0.507265866

P2RX1	Purinergic Receptor P2X 1	Protein Coding	44	GC17M003896	0.490195513
APOBEC3G	Apolipoprotein B MRNA Editing Enzyme Catalytic Subunit 3G	Protein Coding	42	GC22P039078	0.486984909
ESR1	Estrogen Receptor 1	Protein Coding	53	GC06P151656	0.486984909
FPGT	Fucose-1-Phosphate Guanylyltransferase	Protein Coding	32	GC01P074199	0.486984909
MYCN	MYCN Proto-Oncogene, BHLH Transcription Factor	Protein Coding	47	GC02P015949	0.486984909
AQP2	Aquaporin 2	Protein Coding	47	GC12P049950	0.47820586
ERVW-1	Endogenous Retrovirus Group W Member 1, Envelope	Protein Coding	33	GC07M092468	0.474693358
ABCB9	ATP Binding Cassette Subfamily B Member 9	Protein Coding	41	GC12M122920	0.469194829
ETS1	ETS Proto-Oncogene 1, Transcription Factor	Protein Coding	49	GC11M128458	0.469194829
P2RX2	Purinergic Receptor P2X 2	Protein Coding	44	GC12P132618	0.469194829
FLAD1	Flavin Adenine Dinucleotide Synthetase 1	Protein Coding	41	GC01P154983	0.465694785
IL15	Interleukin 15	Protein Coding	41	GC04P141636	0.465694785
MT-ND1	Mitochondrially Encoded NADH:Ubiquinone Oxidoreductase Core Subunit 1	Protein Coding	33	GCMTP003309	0.465694785
ABCB7	ATP Binding Cassette Subfamily B Member 7	Protein Coding	44	GC0XM075053	0.463068247
ACSS1	Acyl-CoA Synthetase Short Chain Family Member 1	Protein Coding	40	GC20M024986	0.463068247

ACSS3	Acyl-CoA Synthetase Short Chain Family Member 3	Protein Coding	40	GC12P080936	0.463068247
AKR1B1	Aldo-Keto Reductase Family 1 Member B	Protein Coding	48	GC07M134442	0.463068247
ALAD	Aminolevulinate Dehydratase	Protein Coding	47	GC09M113386	0.463068247
ALAS2	5'-Aminolevulinate Synthase 2	Protein Coding	46	GC0XM055009	0.463068247
ALS2	Alsin Rho Guanine Nucleotide Exchange Factor ALS2	Protein Coding	45	GC02M201701	0.463068247
ARF4	ADP Ribosylation Factor 4	Protein Coding	44	GC03M057559	0.463068247
ARFGEF2	ADP Ribosylation Factor Guanine Nucleotide Exchange Factor 2	Protein Coding	42	GC20P048921	0.463068247
ASMT	Acetylserotonin O-Methyltransferase	Protein Coding	39	GC0XP001595	0.463068247
ATP6V1D	ATPase H+ Transporting V1 Subunit D	Protein Coding	41	GC14M067294	0.463068247
CCKBR	Cholecystokinin B Receptor	Protein Coding	45	GC11P006259	0.463068247
CGA	Glycoprotein Hormones, Alpha Polypeptide	Protein Coding	43	GC06M087085	0.463068247
CIT	Citron Rho-Interacting Serine/Threonine Kinase	Protein Coding	47	GC12M119650	0.463068247
COPB1	COPI Coat Complex Subunit Beta 1	Protein Coding	39	GC11M014436	0.463068247
CPT1A	Carnitine Palmitoyltransferase 1A	Protein Coding	50	GC11M068754	0.463068247
DLAT	Dihydrolipoamide S-Acetyltransferase	Protein Coding	47	GC11P112024	0.463068247

F3	Coagulation Factor III, Tissue Factor	Protein Coding	45	GC01M094530	0.463068247
FKBP1B	FKBP Prolyl Isomerase 1B	Protein Coding	41	GC02P024033	0.463068247
GADD45A	Growth Arrest And DNA Damage Inducible Alpha	Protein Coding	45	GC01P067685	0.463068247
GBP1	Guanylate Binding Protein 1	Protein Coding	42	GC01M089052	0.463068247
GHRH	Growth Hormone Releasing Hormone	Protein Coding	40	GC20M037251	0.463068247
GNAL	G Protein Subunit Alpha L	Protein Coding	46	GC18P011689	0.463068247
GNRH1	Gonadotropin Releasing Hormone 1	Protein Coding	43	GC08M025419	0.463068247
GPSM1	G Protein Signaling Modulator 1	Protein Coding	38	GC09P136327	0.463068247
HIBCH	3-Hydroxyisobutyryl-CoA Hydrolase	Protein Coding	44	GC02M190189	0.463068247
HTR1E	5-Hydroxytryptamine Receptor 1E	Protein Coding	41	GC06P086937	0.463068247
IGF2R	Insulin Like Growth Factor 2 Receptor	Protein Coding	46	GC06P159969	0.463068247
IL4R	Interleukin 4 Receptor	Protein Coding	48	GC16P027325	0.463068247
ITPR2	Inositol 1,4,5-Trisphosphate Receptor Type 2	Protein Coding	47	GC12M026336	0.463068247
L1CAM	L1 Cell Adhesion Molecule	Protein Coding	47	GC0XM153864	0.463068247
LINC01194	Long Intergenic Non-Protein Coding RNA 1194	RNA Gene	16	GC05P012578	0.463068247
MAT1A	Methionine Adenosyltransferase 1A	Protein Coding	48	GC10M080271	0.463068247
MCF2L	MCF.2 Cell Line Derived Transforming Sequence Like	Protein Coding	43	GC13P112894	0.463068247
MMP9	Matrix Metallopeptidase 9	Protein Coding	54	GC20P046008	0.463068247

NPR3	Natriuretic Peptide Receptor 3	Protein Coding	45	GC05P032689	0.463068247
OGDH	Oxoglutarate Dehydrogenase	Protein Coding	46	GC07P044606	0.463068247
PDHA1	Pyruvate Dehydrogenase E1 Subunit Alpha 1	Protein Coding	50	GC0XP019343	0.463068247
PIK3C2B	Phosphatidylinositol-4-Phosphate 3-Kinase Catalytic Subunit Type 2 Beta	Protein Coding	48	GC01M204422	0.463068247
PIK3C2G	Phosphatidylinositol-4-Phosphate 3-Kinase Catalytic Subunit Type 2 Gamma	Protein Coding	45	GC12P018242	0.463068247
PIK3C3	Phosphatidylinositol 3-Kinase Catalytic Subunit Type 3	Protein Coding	50	GC18P041955	0.463068247
PIK3R4	Phosphoinositide-3-Kinase Regulatory Subunit 4	Protein Coding	47	GC03M130678	0.463068247
PLA2G2A	Phospholipase A2 Group IIA	Protein Coding	48	GC01M019975	0.463068247
PMP22	Peripheral Myelin Protein 22	Protein Coding	41	GC17M015229	0.463068247
PNKD	PNKD Metallo-Beta-Lactamase Domain Containing	Protein Coding	43	GC02P218270	0.463068247
PPT1	Palmitoyl-Protein Thioesterase 1	Protein Coding	46	GC01M040072	0.463068247
PRMT1	Protein Arginine Methyltransferase 1	Protein Coding	48	GC19P049675	0.463068247
PTMA	Prothymosin Alpha	Protein Coding	40	GC02P231707	0.463068247
QTRT2	Queuine TRNA-Ribosyltransferase Accessory Subunit 2	Protein Coding	28	GC03P114006	0.463068247
RALA	RAS Like Proto-Oncogene A	Protein Coding	46	GC07P039622	0.463068247

RALGDS	Ral Guanine Nucleotide Dissociation Stimulator	Protein Coding	43	GC09M133097	0.463068247
RASA2	RAS P21 Protein Activator 2	Protein Coding	41	GC03P141487	0.463068247
RASGRF1	Ras Protein Specific Guanine Nucleotide Releasing Factor 1	Protein Coding	43	GC15M078959	0.463068247
SLC12A3	Solute Carrier Family 12 Member 3	Protein Coding	47	GC16P056865	0.463068247
SOS2	SOS Ras/Rho Guanine Nucleotide Exchange Factor 2	Protein Coding	46	GC14M050117	0.463068247
SPP1	Secreted Phosphoprotein 1	Protein Coding	47	GC04P087975	0.463068247
SSTR2	Somatostatin Receptor 2	Protein Coding	48	GC17P073165	0.463068247
ST3GAL4	ST3 Beta-Galactoside Alpha-2,3-Sialyltransferase 4	Protein Coding	43	GC11P126355	0.463068247
SULT2A1	Sulfotransferase Family 2A Member 1	Protein Coding	45	GC19M047870	0.463068247
TCEAL1	Transcription Elongation Factor A Like 1	Protein Coding	38	GC0XP103628	0.463068247
TGFB2	Transforming Growth Factor Beta 2	Protein Coding	50	GC01P218345	0.463068247
TP53BP1	Tumor Protein P53 Binding Protein 1	Protein Coding	45	GC15M043403	0.463068247
TPO	Thyroid Peroxidase	Protein Coding	49	GC02P001374	0.463068247
TPT1	Tumor Protein, Translationally-Controlled 1	Protein Coding	47	GC13M045333	0.463068247
TRDN	Triadin	Protein Coding	41	GC06M123198	0.463068247
TRHR	Thyrotropin Releasing Hormone Receptor	Protein Coding	44	GC08P109084	0.463068247

USF1	Upstream Transcription Factor 1	Protein Coding	44	GC01M161039	0.463068247
XRCC2	X-Ray Repair Cross Complementing 2	Protein Coding	43	GC07M152644	0.463068247
XRCC3	X-Ray Repair Cross Complementing 3	Protein Coding	43	GC14M103697	0.463068247
H3C14	H3 Clustered Histone 14	Protein Coding	29	GC01M150132	0.446357906
P2RX5	Purinergic Receptor P2X 5	Protein Coding	40	GC17M003672	0.446357906
PFKM	Phosphofructokinase, Muscle	Protein Coding	51	GC12P048105	0.446357906
ABCB11	ATP Binding Cassette Subfamily B Member 11	Protein Coding	47	GC02M168922	0.444437087
AOC1	Amine Oxidase Copper Containing 1	Protein Coding	40	GC07P150824	0.444437087
AURKB	Aurora Kinase B	Protein Coding	50	GC17M009194	0.444437087
AURKC	Aurora Kinase C	Protein Coding	48	GC19P057230	0.444437087
BAD	BCL2 Associated Agonist Of Cell Death	Protein Coding	47	GC11M069329	0.444437087
BLM	BLM RecQ Like Helicase	Protein Coding	50	GC15P090717	0.444437087
CCL4	C-C Motif Chemokine Ligand 4	Protein Coding	41	GC17P036103	0.444437087
CDK20	Cyclin Dependent Kinase 20	Protein Coding	41	GC09M087966	0.444437087
CPOX	Coproporphyrinogen Oxidase	Protein Coding	44	GC03M098576	0.444437087
CSNK1A1L	Casein Kinase 1 Alpha 1 Like	Protein Coding	37	GC13M037103	0.444437087
CSNK1D	Casein Kinase 1 Delta	Protein Coding	51	GC17M082239	0.444437087
CXCL9	C-X-C Motif Chemokine Ligand 9	Protein Coding	39	GC04M076001	0.444437087
DYRK1A	Dual Specificity Tyrosine Phosphorylation Regulated Kinase 1A	Protein Coding	51	GC21P037365	0.444437087

EIF4A3	Eukaryotic Translation Initiation Factor 4A3	Protein Coding	44	GC17M080135	0.444437087
ENO1	Enolase 1	Protein Coding	48	GC01M008861	0.444437087
GCK	Glucokinase	Protein Coding	51	GC07M044154	0.444437087
GTF2B	General Transcription Factor IIB	Protein Coding	43	GC01M088853	0.444437087
HDAC3	Histone Deacetylase 3	Protein Coding	50	GC05M141583	0.444437087
HGF	Hepatocyte Growth Factor	Protein Coding	52	GC07M081699	0.444437087
HIPK2	Homeodomain Interacting Protein Kinase 2	Protein Coding	45	GC07M139561	0.444437087
HSPB1	Heat Shock Protein Family B (Small) Member 1	Protein Coding	52	GC07P076302	0.444437087
HTT	Huntingtin	Protein Coding	44	GC04P003041	0.444437087
IKZF1	IKAROS Family Zinc Finger 1	Protein Coding	48	GC07P050303	0.444437087
KIF11	Kinesin Family Member 11	Protein Coding	48	GC10P092574	0.444437087
LIMK1	LIM Domain Kinase 1	Protein Coding	50	GC07P074082	0.444437087
LIMK2	LIM Domain Kinase 2	Protein Coding	48	GC22P031212	0.444437087
MLST8	MTOR Associated Protein, LST8 Homolog	Protein Coding	43	GC16P002204	0.444437087
NR3C2	Nuclear Receptor Subfamily 3 Group C Member 2	Protein Coding	48	GC04M148078	0.444437087
NUAK2	NUAK Family Kinase 2	Protein Coding	40	GC01M205302	0.444437087
PBX1	PBX Homeobox 1	Protein Coding	50	GC01P164524	0.444437087
PECAM1	Platelet And Endothelial Cell Adhesion Molecule 1	Protein Coding	41	GC17M064319	0.444437087
POLR2A	RNA Polymerase II Subunit A	Protein Coding	45	GC17P009102	0.444437087
POLRMT	RNA Polymerase Mitochondrial	Protein Coding	42	GC19M000617	0.444437087
PPP1R1B	Protein Phosphatase 1 Regulatory Inhibitor Subunit 1B	Protein Coding	44	GC17P039626	0.444437087

PRKAR2B	Protein Kinase CAMP-Dependent Type II Regulatory Subunit Beta	Protein Coding	45	GC07P107044	0.444437087
PRKCA	Protein Kinase C Alpha	Protein Coding	52	GC17P066302	0.444437087
PRKCB	Protein Kinase C Beta	Protein Coding	48	GC16P023967	0.444437087
PRKCI	Protein Kinase C Iota	Protein Coding	49	GC03P170222	0.444437087
RGS2	Regulator Of G Protein Signaling 2	Protein Coding	43	GC01P192809	0.444437087
RIOK1	RIO Kinase 1	Protein Coding	41	GC06P007389	0.444437087
ROCK1	Rho Associated Coiled-Coil Containing Protein Kinase 1	Protein Coding	51	GC18M020946	0.444437087
S1PR1	Sphingosine-1-Phosphate Receptor 1	Protein Coding	45	GC01P101236	0.444437087
SLC25A4	Solute Carrier Family 25 Member 4	Protein Coding	50	GC04P185143	0.444437087
SMURF1	SMAD Specific E3 Ubiquitin Protein Ligase 1	Protein Coding	45	GC07M099027	0.444437087
SRPK1	SRSF Protein Kinase 1	Protein Coding	46	GC06M047030	0.444437087
STMN1	Stathmin 1	Protein Coding	44	GC01M025884	0.444437087
TEK	TEK Receptor Tyrosine Kinase	Protein Coding	51	GC09P027109	0.444437087
TLR5	Toll Like Receptor 5	Protein Coding	48	GC01M223132	0.444437087
TRPC1	Transient Receptor Potential Cation Channel Subfamily C Member 1	Protein Coding	43	GC03P142724	0.444437087
TTN	Titin	Protein Coding	48	GC02M178525	0.444437087
TYRP1	Tyrosinase Related Protein 1	Protein Coding	47	GC09P012683	0.444437087
UBE2D1	Ubiquitin Conjugating Enzyme E2 D1	Protein Coding	46	GC10P058334	0.444437087
UBE2S	Ubiquitin Conjugating Enzyme E2 S	Protein Coding	42	GC19M055399	0.444437087

VGF	VGF Nerve Growth Factor Inducible	Protein Coding	38	GC07M101162	0.444437087
ADCYAP1	Adenylate Cyclase Activating Polypeptide 1	Protein Coding	43	GC18P000895	0.444196105
C5AR1	Complement C5a Receptor 1	Protein Coding	44	GC19P047290	0.444196105
CYSLTR2	Cysteinyl Leukotriene Receptor 2	Protein Coding	48	GC13P048653	0.444196105
MT-ND4L	Mitochondrially Encoded NADH:Ubiquinone Oxidoreductase Core Subunit 4L	Protein Coding	29	GCMTP010472	0.444196105
PTAFR	Platelet Activating Factor Receptor	Protein Coding	44	GC01M028147	0.444196105
PTH	Parathyroid Hormone	Protein Coding	47	GC11M013492	0.444196105
YY1	YY1 Transcription Factor	Protein Coding	48	GC14P100238	0.444196105
CDC20	Cell Division Cycle 20	Protein Coding	45	GC01P043358	0.442662537
PGR	Progesterone Receptor	Protein Coding	51	GC11M101030	0.442662537
H1-4	H1.4 Linker Histone, Cluster Member	Protein Coding	36	GC06P055576	0.422682464
H4C1	H4 Clustered Histone 1	Protein Coding	33	GC06P054981	0.422682464
H4C11	H4 Clustered Histone 11	Protein Coding	30	GC06P055048	0.422682464
H4C12	H4 Clustered Histone 12	Protein Coding	29	GC06M047220	0.422682464
H4C13	H4 Clustered Histone 13	Protein Coding	29	GC06M047221	0.422682464
H4C2	H4 Clustered Histone 2	Protein Coding	31	GC06M026026	0.422682464
H4C3	H4 Clustered Histone 3	Protein Coding	30	GC06P054986	0.422682464
H4C4	H4 Clustered Histone 4	Protein Coding	27	GC06M047223	0.422682464
H4C5	H4 Clustered Histone 5	Protein Coding	28	GC06P055590	0.422682464
H4C6	H4 Clustered Histone 6	Protein Coding	29	GC06P055591	0.422682464
H4C8	H4 Clustered Histone 8	Protein Coding	30	GC06M047225	0.422682464

H4C9	H4 Clustered Histone 9	Protein Coding	30	GC06P055592	0.422682464
NEIL2	Nei Like DNA Glycosylase 2	Protein Coding	40	GC08P011769	0.422682464
SRSF7	Serine And Arginine Rich Splicing Factor 7	Protein Coding	40	GC02M038743	0.422682464
H2BC21	H2B Clustered Histone 21	Protein Coding	33	GC01M150135	0.421097755
HIPK3	Homeodomain Interacting Protein Kinase 3	Protein Coding	43	GC11P033278	0.421097755
PF4	Platelet Factor 4	Protein Coding	42	GC04M073980	0.421097755
ABCC10	ATP Binding Cassette Subfamily C Member 10	Protein Coding	41	GC06P043427	0.414180815
ABHD5	Abhydrolase Domain Containing 5, Lysophosphatidic Acid Acyltransferase	Protein Coding	45	GC03P043707	0.414180815
ACAT1	Acetyl-CoA Acetyltransferase 1	Protein Coding	50	GC11P108121	0.414180815
ACAT2	Acetyl-CoA Acetyltransferase 2	Protein Coding	47	GC06P159760	0.414180815
ACOT12	Acyl-CoA Thioesterase 12	Protein Coding	39	GC05M081309	0.414180815
AGER	Advanced Glycosylation End-Product Specific Receptor	Protein Coding	45	GC06M032180	0.414180815
ALAS1	5'-Aminolevulinate Synthase 1	Protein Coding	45	GC03P052198	0.414180815
ALKBH8	AlkB Homolog 8, TRNA Methyltransferase	Protein Coding	38	GC11M107502	0.414180815
ALOX5	Arachidonate 5-Lipoxygenase	Protein Coding	50	GC10P045374	0.414180815
ATG5	Autophagy Related 5	Protein Coding	44	GC06M106045	0.414180815
AXL	AXL Receptor Tyrosine Kinase	Protein Coding	52	GC19P041219	0.414180815

BECN1	Beclin 1	Protein Coding	47	GC17M042810	0.414180815
BUD23	BUD23 RRNA Methyltransferase And Ribosome Maturation Factor	Protein Coding	31	GC07P073686	0.414180815
CAMKMT	Calmodulin-Lysine N-Methyltransferase	Protein Coding	36	GC02P044361	0.414180815
CD28	CD28 Molecule	Protein Coding	47	GC02P203706	0.414180815
CD55	CD55 Molecule (Cromer Blood Group)	Protein Coding	48	GC01P207321	0.414180815
CDY1	Chromodomain Y-Linked 1	Protein Coding	33	GC0YP025709	0.414180815
CDY2A	Chromodomain Y-Linked 2A	Protein Coding	28	GC0YP018025	0.414180815
CDYL	Chromodomain Y Like	Protein Coding	41	GC06P004706	0.414180815
CERS1	Ceramide Synthase 1	Protein Coding	41	GC19M018868	0.414180815
CERS2	Ceramide Synthase 2	Protein Coding	40	GC01M150934	0.414180815
CERS3	Ceramide Synthase 3	Protein Coding	40	GC15M107485	0.414180815
CERS4	Ceramide Synthase 4	Protein Coding	38	GC19P008206	0.414180815
CERS5	Ceramide Synthase 5	Protein Coding	37	GC12M050129	0.414180815
CERS6	Ceramide Synthase 6	Protein Coding	38	GC02P168455	0.414180815
CES1	Carboxylesterase 1	Protein Coding	47	GC16M055836	0.414180815
CLOCK	Clock Circadian Regulator	Protein Coding	44	GC04M055427	0.414180815
CMTR1	Cap Methyltransferase 1	Protein Coding	35	GC06P055361	0.414180815
COMTD1	Catechol-O-Methyltransferase Domain Containing 1	Protein Coding	38	GC10M075233	0.414180815
COQ3	Coenzyme Q3, Methyltransferase	Protein Coding	41	GC06M099369	0.414180815
COQ5	Coenzyme Q5, Methyltransferase	Protein Coding	41	GC12M120503	0.414180815
CPNE1	Copine 1	Protein Coding	40	GC20M035626	0.414180815

CPT1B	Carnitine Palmitoyltransferase 1B	Protein Coding	45	GC22M050569	0.414180815
CPT1C	Carnitine Palmitoyltransferase 1C	Protein Coding	43	GC19P049690	0.414180815
CST3	Cystatin C	Protein Coding	45	GC20M023627	0.414180815
CTSB	Cathepsin B	Protein Coding	51	GC08M011842	0.414180815
DIMT1	DIMT1 RRNA Methyltransferase And Ribosome Maturation Factor	Protein Coding	37	GC05M062387	0.414180815
DLST	Dihydrolipoamide S-Succinyltransferase	Protein Coding	47	GC14P074881	0.414180815
DOT1L	DOT1 Like Histone Lysine Methyltransferase	Protein Coding	45	GC19P002164	0.414180815
DPH5	Diphthamide Biosynthesis 5	Protein Coding	37	GC01M100989	0.414180815
EHMT1	Euchromatic Histone Lysine Methyltransferase 1	Protein Coding	46	GC09P137618	0.414180815
EHMT2	Euchromatic Histone Lysine Methyltransferase 2	Protein Coding	46	GC06M031879	0.414180815
ELN	Elastin	Protein Coding	45	GC07P074027	0.414180815
ELOVL1	ELOVL Fatty Acid Elongase 1	Protein Coding	41	GC01M043363	0.414180815
ELOVL2	ELOVL Fatty Acid Elongase 2	Protein Coding	41	GC06M010980	0.414180815
ELOVL3	ELOVL Fatty Acid Elongase 3	Protein Coding	34	GC10P102226	0.414180815
ELOVL4	ELOVL Fatty Acid Elongase 4	Protein Coding	46	GC06M079914	0.414180815
ELOVL5	ELOVL Fatty Acid Elongase 5	Protein Coding	44	GC06M053240	0.414180815
ELOVL6	ELOVL Fatty Acid Elongase 6	Protein Coding	41	GC04M110045	0.414180815
ELOVL7	ELOVL Fatty Acid Elongase 7	Protein Coding	38	GC05M060751	0.414180815
ELP3	Elongator Acetyltransferase Complex Subunit 3	Protein Coding	40	GC08P028089	0.414180815

EMG1	EMG1 N1-Specific Pseudouridine Methyltransferase	Protein Coding	41	GC12P006970	0.414180815
EZH1	Enhancer Of Zeste 1 Polycomb Repressive Complex 2 Subunit	Protein Coding	45	GC17M042700	0.414180815
EZH2	Enhancer Of Zeste 2 Polycomb Repressive Complex 2 Subunit	Protein Coding	54	GC07M148807	0.414180815
FABP4	Fatty Acid Binding Protein 4	Protein Coding	45	GC08M081478	0.414180815
FGF23	Fibroblast Growth Factor 23	Protein Coding	46	GC12M004368	0.414180815
FTSJ1	FtsJ RNA 2'-O-Methyltransferase 1	Protein Coding	42	GC0XP048476	0.414180815
FTSJ3	FtsJ RNA 2'-O-Methyltransferase 3	Protein Coding	37	GC17M063819	0.414180815
GCAT	Glycine C-Acetyltransferase	Protein Coding	43	GC22P037807	0.414180815
GNPNAT1	Glucosamine-Phosphate N-Acetyltransferase 1	Protein Coding	40	GC14M052775	0.414180815
GTF3C4	General Transcription Factor IIIC Subunit 4	Protein Coding	36	GC09P132671	0.414180815
HAT1	Histone Acetyltransferase 1	Protein Coding	44	GC02P171922	0.414180815
HEMK1	HemK Methyltransferase Family Member 1	Protein Coding	36	GC03P050569	0.414180815
HENMT1	HEN Methyltransferase 1	Protein Coding	33	GC01M108648	0.414180815
HFE	Homeostatic Iron Regulator	Protein Coding	44	GC06P026087	0.414180815

HGSNAT	Heparan-Alpha-Glucosaminide N-Acetyltransferase	Protein Coding	38	GC08P043140	0.414180815
HM13	Histocompatibility Minor 13	Protein Coding	39	GC20P031514	0.414180815
HMGCS2	3-Hydroxy-3-Methylglutaryl-CoA Synthase 2	Protein Coding	45	GC01M119747	0.414180815
HNF1B	HNF1 Homeobox B	Protein Coding	44	GC17M037686	0.414180815
HNMT	Histamine N-Methyltransferase	Protein Coding	46	GC02P137964	0.414180815
HRH1	Histamine Receptor H1	Protein Coding	46	GC03P011113	0.414180815
ICMT	Isoprenylcysteine Carboxyl Methyltransferase	Protein Coding	43	GC01M006222	0.414180815
IGFBP1	Insulin Like Growth Factor Binding Protein 1	Protein Coding	44	GC07P046838	0.414180815
IL16	Interleukin 16	Protein Coding	43	GC15P081159	0.414180815
IL5	Interleukin 5	Protein Coding	45	GC05M132541	0.414180815
INMT	Indolethylamine N-Methyltransferase	Protein Coding	41	GC07P030737	0.414180815
KAT2A	Lysine Acetyltransferase 2A	Protein Coding	48	GC17M042113	0.414180815
KAT6B	Lysine Acetyltransferase 6B	Protein Coding	43	GC10P074840	0.414180815
KAT7	Lysine Acetyltransferase 7	Protein Coding	43	GC17P049788	0.414180815
KAT8	Lysine Acetyltransferase 8	Protein Coding	42	GC16P032545	0.414180815
KIT	KIT Proto-Oncogene, Receptor Tyrosine Kinase	Protein Coding	53	GC04P054657	0.414180815
KMT2B	Lysine Methyltransferase 2B	Protein Coding	39	GC19P040361	0.414180815
KMT2C	Lysine Methyltransferase 2C	Protein Coding	42	GC07M152134	0.414180815

KMT2D	Lysine Methyltransferase 2D	Protein Coding	42	GC12M049018	0.414180815
KMT2E	Lysine Methyltransferase 2E (Inactive)	Protein Coding	40	GC07P104950	0.414180815
KMT5A	Lysine Methyltransferase 5A	Protein Coding	38	GC12P123621	0.414180815
KMT5B	Lysine Methyltransferase 5B	Protein Coding	33	GC11M069558	0.414180815
KMT5C	Lysine Methyltransferase 5C	Protein Coding	30	GC19P056474	0.414180815
LCMT1	Leucine Carboxyl Methyltransferase 1	Protein Coding	36	GC16P026740	0.414180815
LCN1	Lipocalin 1	Protein Coding	40	GC09P135521	0.414180815
LEPQTL1	Leptin, Serum Levels Of	Genetic Locus	2	GC02U903086	0.414180815
LGALS1	Galectin 1	Protein Coding	44	GC22P037675	0.414180815
LPA	Lipoprotein(A)	Protein Coding	42	GC06M160531	0.414180815
LPCAT2	Lysophosphatidylcholine Acyltransferase 2	Protein Coding	43	GC16P055510	0.414180815
LRTOMT	Leucine Rich Transmembrane And O-Methyltransferase Domain Containing	Protein Coding	38	GC11P072080	0.414180815
MBOAT1	Membrane Bound O-Acyltransferase Domain Containing 1	Protein Coding	37	GC06M020102	0.414180815
MBOAT2	Membrane Bound O-Acyltransferase Domain Containing 2	Protein Coding	36	GC02M008853	0.414180815
MBOAT7	Membrane Bound O-Acyltransferase Domain Containing 7	Protein Coding	41	GC19M054173	0.414180815
MCAT	Malonyl-CoA-Acyl Carrier Protein Transacylase	Protein Coding	43	GC22M043132	0.414180815

METTL1	Methyltransferase Like 1	Protein Coding	43	GC12M057768	0.414180815
METTL2B	Methyltransferase Like 2B	Protein Coding	31	GC07P130039	0.414180815
MRM2	Mitochondrial RRNA Methyltransferase 2	Protein Coding	31	GC07M002234	0.414180815
NAA10	N-Alpha-Acetyltransferase 10, NatA Catalytic Subunit	Protein Coding	43	GC0XM153929	0.414180815
NAA20	N-Alpha-Acetyltransferase 20, NatB Catalytic Subunit	Protein Coding	37	GC20P020018	0.414180815
NAA30	N-Alpha-Acetyltransferase 30, NatC Catalytic Subunit	Protein Coding	37	GC14P057390	0.414180815
NAA60	N-Alpha-Acetyltransferase 60, NatF Catalytic Subunit	Protein Coding	35	GC16P003443	0.414180815
NAGS	N-Acetylglutamate Synthase	Protein Coding	43	GC17P044004	0.414180815
NAT8L	N-Acetyltransferase 8 Like	Protein Coding	37	GC04P002061	0.414180815
NMT1	N-Myristoyltransferase 1	Protein Coding	44	GC17P044958	0.414180815
NMT2	N-Myristoyltransferase 2	Protein Coding	41	GC10M015115	0.414180815
NOMO1	NODAL Modulator 1	Protein Coding	33	GC16P015473	0.414180815
NSD1	Nuclear Receptor Binding SET Domain Protein 1	Protein Coding	44	GC05P177134	0.414180815
NSD2	Nuclear Receptor Binding SET Domain Protein 2	Protein Coding	36	GC04P001872	0.414180815
NSD3	Nuclear Receptor Binding SET Domain Protein 3	Protein Coding	35	GC08M038269	0.414180815

NSUN2	NOP2/Sun RNA Methyltransferase 2	Protein Coding	43	GC05M006599	0.414180815
NTMT1	N-Terminal Xaa-Pro-Lys N-Methyltransferase 1	Protein Coding	39	GC09P129608	0.414180815
OCA2	OCA2 Melanosomal Transmembrane Protein	Protein Coding	43	GC15M027754	0.414180815
OCRL	OCRL Inositol Polyphosphate-5-Phosphatase	Protein Coding	45	GC0XP129539	0.414180815
OGA	O-GlcNAcase	Protein Coding	33	GC10M101785	0.414180815
OGDHL	Oxoglutarate Dehydrogenase L	Protein Coding	39	GC10M049734	0.414180815
OXSM	3-Oxoacyl-ACP Synthase, Mitochondrial	Protein Coding	39	GC03P025782	0.414180815
PCMT1	Protein-L-Isoaspartate (D-Aspartate) O-Methyltransferase	Protein Coding	41	GC06P149749	0.414180815
PDHA2	Pyruvate Dehydrogenase E1 Subunit Alpha 2	Protein Coding	43	GC04P095840	0.414180815
PDHB	Pyruvate Dehydrogenase E1 Subunit Beta	Protein Coding	47	GC03M058428	0.414180815
PEMT	Phosphatidylethanolamine N-Methyltransferase	Protein Coding	41	GC17M017506	0.414180815
PIGW	Phosphatidylinositol Glycan Anchor Biosynthesis Class W	Protein Coding	38	GC17P036534	0.414180815
PIK3R2	Phosphoinositide-3-Kinase Regulatory Subunit 2	Protein Coding	50	GC19P018153	0.414180815
PIK3R3	Phosphoinositide-3-Kinase Regulatory Subunit 3	Protein Coding	44	GC01M046041	0.414180815
PIK3R5	Phosphoinositide-3-Kinase Regulatory Subunit 5	Protein Coding	47	GC17M008878	0.414180815

PNMT	Phenylethanolamine N-Methyltransferase	Protein Coding	45	GC17P039667	0.414180815
POMT1	Protein O-Mannosyltransferase 1	Protein Coding	47	GC09P131502	0.414180815
PPT2	Palmitoyl-Protein Thioesterase 2	Protein Coding	38	GC06P032153	0.414180815
PRDM6	PR/SET Domain 6	Protein Coding	40	GC05P123089	0.414180815
PRMT2	Protein Arginine Methyltransferase 2	Protein Coding	42	GC21P046635	0.414180815
PRMT3	Protein Arginine Methyltransferase 3	Protein Coding	43	GC11P020409	0.414180815
PRMT7	Protein Arginine Methyltransferase 7	Protein Coding	45	GC16P068363	0.414180815
RBM14	RNA Binding Motif Protein 14	Protein Coding	37	GC11P066785	0.414180815
RIC3	RIC3 Acetylcholine Receptor Chaperone	Protein Coding	36	GC11M008092	0.414180815
ROR2	Receptor Tyrosine Kinase Like Orphan Receptor 2	Protein Coding	49	GC09M091564	0.414180815
SAA4	Serum Amyloid A4, Constitutive	Protein Coding	40	GC11M018234	0.414180815
SAT1	Spermidine/Spermine N1-Acetyltransferase 1	Protein Coding	46	GC0XP023784	0.414180815
SAT2	Spermidine/Spermine N1-Acetyltransferase Family Member 2	Protein Coding	40	GC17M007626	0.414180815
SERPINE1	Serpin Family E Member 1	Protein Coding	51	GC07P101127	0.414180815
SETD1A	SET Domain Containing 1A, Histone Lysine Methyltransferase	Protein Coding	41	GC16P032486	0.414180815

SETD1B	SET Domain Containing 1B, Histone Lysine Methyltransferase	Protein Coding	37	GC12P123586	0.414180815
SETD3	SET Domain Containing 3, Actin Histidine Methyltransferase	Protein Coding	38	GC14M099397	0.414180815
SETD7	SET Domain Containing 7, Histone Lysine Methyltransferase	Protein Coding	44	GC04M139495	0.414180815
SETDB1	SET Domain Bifurcated Histone Lysine Methyltransferase 1	Protein Coding	43	GC01P150926	0.414180815
SETMAR	SET Domain And Mariner Transposase Fusion Gene	Protein Coding	41	GC03P004303	0.414180815
SLC37A4	Solute Carrier Family 37 Member 4	Protein Coding	44	GC11M119024	0.414180815
SMYD2	SET And MYND Domain Containing 2	Protein Coding	44	GC01P214281	0.414180815
SMYD3	SET And MYND Domain Containing 3	Protein Coding	43	GC01M245749	0.414180815
SPTLC1	Serine Palmitoyltransferase Long Chain Base Subunit 1	Protein Coding	47	GC09M092002	0.414180815
SPTLC2	Serine Palmitoyltransferase Long Chain Base Subunit 2	Protein Coding	48	GC14M077505	0.414180815
SPTLC3	Serine Palmitoyltransferase Long Chain Base Subunit 3	Protein Coding	41	GC20P013008	0.414180815
SUV39H1	Suppressor Of Variegation 3-9 Homolog 1	Protein Coding	44	GC0XP048699	0.414180815
SUV39H2	Suppressor Of Variegation 3-9 Homolog 2	Protein Coding	44	GC10P014878	0.414180815

THEM4	Thioesterase Superfamily Member 4	Protein Coding	40	GC01M151870	0.414180815
THEM5	Thioesterase Superfamily Member 5	Protein Coding	36	GC01M151850	0.414180815
TRMT1	TRNA Methyltransferase 1	Protein Coding	43	GC19M013104	0.414180815
TRMT13	TRNA Methyltransferase 13 Homolog	Protein Coding	30	GC01P100133	0.414180815
TRMT2B	TRNA Methyltransferase 2 Homolog B	Protein Coding	34	GC0XM101024	0.414180815
TRMT44	TRNA Methyltransferase 44 Homolog	Protein Coding	32	GC04P008439	0.414180815
TRMT5	TRNA Methyltransferase 5	Protein Coding	39	GC14M060971	0.414180815
TRMT61A	TRNA Methyltransferase 61A	Protein Coding	34	GC14P103529	0.414180815
TRMT61B	TRNA Methyltransferase 61B	Protein Coding	34	GC02M028849	0.414180815
TRMU	TRNA Mitochondrial 2-Thiouridylase	Protein Coding	40	GC22P046330	0.414180815
TTLL3	Tubulin Tyrosine Ligase Like 3	Protein Coding	36	GC03P009808	0.414180815
ZDHHC1	Zinc Finger DHHC-Type Containing 1	Protein Coding	36	GC16M067394	0.414180815
ZDHHC12	Zinc Finger DHHC-Type Palmitoyltransferase 12	Protein Coding	31	GC09M128720	0.414180815
ZDHHC13	Zinc Finger DHHC-Type Palmitoyltransferase 13	Protein Coding	38	GC11P019095	0.414180815
ZDHHC14	Zinc Finger DHHC-Type Palmitoyltransferase 14	Protein Coding	36	GC06P157381	0.414180815
ZDHHC17	Zinc Finger DHHC-Type Palmitoyltransferase 17	Protein Coding	38	GC12P076763	0.414180815

ZDHHC18	Zinc Finger DHHC-Type Palmitoyltransferase 18	Protein Coding	34	GC01P026911	0.414180815
ZDHHC2	Zinc Finger DHHC-Type Palmitoyltransferase 2	Protein Coding	39	GC08P017156	0.414180815
ZDHHC20	Zinc Finger DHHC-Type Palmitoyltransferase 20	Protein Coding	37	GC13M021372	0.414180815
ZDHHC21	Zinc Finger DHHC-Type Palmitoyltransferase 21	Protein Coding	36	GC09M014590	0.414180815
ZDHHC24	Zinc Finger DHHC-Type Containing 24	Protein Coding	34	GC11M069469	0.414180815
ZDHHC3	Zinc Finger DHHC-Type Palmitoyltransferase 3	Protein Coding	37	GC03M044915	0.414180815
ZDHHC4	Zinc Finger DHHC-Type Palmitoyltransferase 4	Protein Coding	36	GC07P006577	0.414180815
ZDHHC5	Zinc Finger DHHC-Type Palmitoyltransferase 5	Protein Coding	37	GC11P057670	0.414180815
ZDHHC6	Zinc Finger DHHC-Type Palmitoyltransferase 6	Protein Coding	37	GC10M112430	0.414180815
ZDHHC7	Zinc Finger DHHC-Type Palmitoyltransferase 7	Protein Coding	38	GC16M084975	0.414180815
ZDHHC8	Zinc Finger DHHC-Type Palmitoyltransferase 8	Protein Coding	40	GC22P020129	0.414180815
ANGPT1	Angiopoietin 1	Protein Coding	46	GC08M107246	0.411096483
CCNB2	Cyclin B2	Protein Coding	45	GC15P059105	0.411096483
CCNE1	Cyclin E1	Protein Coding	49	GC19P029811	0.411096483
CCP110	Centriolar Coiled-Coil Protein 110	Protein Coding	37	GC16P019536	0.411096483
CDC5L	Cell Division Cycle 5 Like	Protein Coding	40	GC06P044387	0.411096483

NF2	Neurofibromin 2	Protein Coding	49	GC22P029603	0.411096483
SGSH	N-Sulfoglucosamine Sulfohydrolase	Protein Coding	45	GC17M080206	0.411096483
SHH	Sonic Hedgehog Signaling Molecule	Protein Coding	50	GC07M155799	0.411096483
TRPC6	Transient Receptor Potential Cation Channel Subfamily C Member 6	Protein Coding	49	GC11M101451	0.411096483
HCRT	Hypocretin Neuropeptide Precursor	Protein Coding	41	GC17M042185	0.399873734
HSPD1	Heat Shock Protein Family D (Hsp60) Member 1	Protein Coding	48	GC02M197486	0.399873734
ITGB1	Integrin Subunit Beta 1	Protein Coding	51	GC10M032890	0.399873734
JUNB	JunB Proto-Oncogene, AP-1 Transcription Factor Subunit	Protein Coding	43	GC19P012791	0.399873734
KDM1A	Lysine Demethylase 1A	Protein Coding	48	GC01P023019	0.399873734
MIP	Major Intrinsic Protein Of Lens Fiber	Protein Coding	42	GC12M056449	0.399873734
MT-ND6	Mitochondrially Encoded NADH:Ubiquinone Oxidoreductase Core Subunit 6	Protein Coding	33	GCMTM014151	0.399873734
SLC9A3	Solute Carrier Family 9 Member A3	Protein Coding	47	GC05M000472	0.399873734
TXNRD1	Thioredoxin Reductase 1	Protein Coding	48	GC12P104215	0.399873734
CRH	Corticotropin Releasing Hormone	Protein Coding	44	GC08M066176	0.392426193
CTPS1	CTP Synthase 1	Protein Coding	45	GC01P040979	0.392426193
GLO1	Glyoxalase I	Protein Coding	47	GC06M047047	0.392426193
OLA1	Obg Like ATPase 1	Protein Coding	43	GC02M174072	0.392426193
PLP1	Proteolipid Protein 1	Protein Coding	44	GC0XP103773	0.392426193
CLK3	CDC Like Kinase 3	Protein Coding	43	GC15P074598	0.388672709

DRD2	Dopamine Receptor D2	Protein Coding	51	GC11M113409	0.388672709
FLI1	Fli-1 Proto-Oncogene, ETS Transcription Factor	Protein Coding	49	GC11P128686	0.388672709
GLDC	Glycine Decarboxylase	Protein Coding	48	GC09M006522	0.388672709
GLS2	Glutaminase 2	Protein Coding	43	GC12M056470	0.388672709
GPRC6A	G Protein-Coupled Receptor Class C Group 6 Member A	Protein Coding	40	GC06M116793	0.388672709
H4C14	H4 Clustered Histone 14	Protein Coding	29	GC01P149832	0.388672709
H4C15	H4 Clustered Histone 15	Protein Coding	27	GC01M150133	0.388672709
MTERF2	Mitochondrial Transcription Termination Factor 2	Protein Coding	29	GC12M106977	0.388672709
NFKB1	Nuclear Factor Kappa B Subunit 1	Protein Coding	52	GC04P102501	0.388672709
UGT2B17	UDP Glucuronosyltransferase Family 2 Member B17	Protein Coding	41	GC04M068537	0.388672709
BLVRB	Biliverdin Reductase B	Protein Coding	41	GC19M040447	0.358691126
ACR	Acrosin	Protein Coding	40	GC22P050738	0.358691126
ALKBH1	AlkB Homolog 1, Histone H2A Dioxygenase	Protein Coding	36	GC14M077672	0.358691126
BIRC2	Baculoviral IAP Repeat Containing 2	Protein Coding	46	GC11P102347	0.358691126
CD22	CD22 Molecule	Protein Coding	46	GC19P035319	0.358691126
DOCK8	Dedicator Of Cytokinesis 8	Protein Coding	43	GC09P000214	0.358691126
ECH1	Enoyl-CoA Hydratase 1	Protein Coding	41	GC19M038815	0.358691126
ESCO1	Establishment Of Sister Chromatid Cohesion N-Acetyltransferase 1	Protein Coding	38	GC18M021529	0.358691126
GPC3	Glypican 3	Protein Coding	46	GC0XM133535	0.358691126
IDUA	Alpha-L-Iduronidase	Protein Coding	43	GC04P000986	0.358691126

MCIDAS	Multiciliate Differentiation And DNA Synthesis Associated Cell Cycle Protein	Protein Coding	31	GC05M055219	0.358691126
MT-ND4	Mitochondrially Encoded NADH:Ubiquinone Oxidoreductase Core Subunit 4	Protein Coding	33	GCMTP010762	0.358691126
NDUFA5	NADH:Ubiquinone Oxidoreductase Subunit A5	Protein Coding	43	GC07M123536	0.358691126
ORM1	Orosomucoid 1	Protein Coding	40	GC09P114323	0.358691126
ORM2	Orosomucoid 2	Protein Coding	36	GC09P114329	0.358691126
POU2F2	POU Class 2 Homeobox 2	Protein Coding	44	GC19M042086	0.358691126
PTGDR2	Prostaglandin D2 Receptor 2	Protein Coding	42	GC11M060850	0.358691126
SDHB	Succinate Dehydrogenase Complex Iron Sulfur Subunit B	Protein Coding	48	GC01M017238	0.358691126
SULT2B1	Sulfotransferase Family 2B Member 1	Protein Coding	46	GC19P048552	0.358691126
TKFC	Triokinase And FMN Cyclase	Protein Coding	40	GC11P061334	0.358691126
ALDOB	Aldolase, Fructose-Bisphosphate B	Protein Coding	46	GC09M101420	0.358416438
ANGPT2	Angiopoietin 2	Protein Coding	46	GC08M006499	0.358416438
ATP6V0D1	ATPase H+ Transporting V0 Subunit D1	Protein Coding	44	GC16M067438	0.358416438
CFL1	Cofilin 1	Protein Coding	47	GC11M065823	0.358416438
CNBP	CCHC-Type Zinc Finger Nucleic Acid Binding Protein	Protein Coding	43	GC03M129167	0.358416438
ERN1	Endoplasmic Reticulum To Nucleus Signaling 1	Protein Coding	46	GC17M064039	0.358416438
GAD1	Glutamate Decarboxylase 1	Protein Coding	52	GC02P170813	0.358416438
GK	Glycerol Kinase	Protein Coding	48	GC0XP030747	0.358416438

LSM2	LSM2 Homolog, U6 Small Nuclear RNA And MRNA Degradation Associated	Protein Coding	41	GC06M046897	0.358416438
MID1	Midline 1	Protein Coding	43	GC0XM010445	0.358416438
MT-CO1	Mitochondrially Encoded Cytochrome C Oxidase I	Protein Coding	34	GCMTP005906	0.358416438
MT-CYB	Mitochondrially Encoded Cytochrome B	Protein Coding	32	GCMTP014749	0.358416438
NFE2L2	Nuclear Factor, Erythroid 2 Like 2	Protein Coding	50	GC02M177227	0.358416438
PFKFB3	6-Phosphofructo-2-Kinase/Fructose-2,6-Biphosphatase 3	Protein Coding	45	GC10P006144	0.358416438
RAD18	RAD18 E3 Ubiquitin Protein Ligase	Protein Coding	43	GC03M008775	0.358416438
TNFRSF10B	TNF Receptor Superfamily Member 10b	Protein Coding	51	GC08M023020	0.358416438
VDAC1	Voltage Dependent Anion Channel 1	Protein Coding	47	GC05M133975	0.358416438
AICDA	Activation Induced Cytidine Deaminase	Protein Coding	46	GC12M008602	0.354891032
EFTUD2	Elongation Factor Tu GTP Binding Domain Containing 2	Protein Coding	43	GC17M044852	0.344350338
A1CF	APOBEC1 Complementation Factor	Protein Coding	37	GC10M050799	0.344350338
ALPP	Alkaline Phosphatase, Placental	Protein Coding	47	GC02P232378	0.344350338
ANKRD44	Ankyrin Repeat Domain 44	Protein Coding	36	GC02M196967	0.344350338
ARFGAP3	ADP Ribosylation Factor GTPase Activating Protein 3	Protein Coding	41	GC22M042796	0.344350338

BCAR1	BCAR1 Scaffold Protein, Cas Family Member	Protein Coding	45	GC16M075228	0.344350338
CHIA	Chitinase Acidic	Protein Coding	43	GC01P111291	0.344350338
COIL	Coilin	Protein Coding	41	GC17M056938	0.344350338
CSTF2T	Cleavage Stimulation Factor Subunit 2 Tau Variant	Protein Coding	41	GC10M051695	0.344350338
DLL1	Delta Like Canonical Notch Ligand 1	Protein Coding	46	GC06M170282	0.344350338
F10	Coagulation Factor X	Protein Coding	50	GC13P113122	0.344350338
FOXO1	Forkhead Box O1	Protein Coding	48	GC13M040555	0.344350338
FOXO3	Forkhead Box O3	Protein Coding	44	GC06P108559	0.344350338
GRIN1	Glutamate Ionotropic Receptor NMDA Type Subunit 1	Protein Coding	51	GC09P137138	0.344350338
GRXCR1	Glutaredoxin And Cysteine Rich Domain Containing 1	Protein Coding	35	GC04P042897	0.344350338
HIVEP1	HIVEP Zinc Finger 1	Protein Coding	37	GC06P012009	0.344350338
LOC154449	Uncharacterized LOC154449	RNA Gene	13	GC06M170179	0.344350338
MFGE8	Milk Fat Globule EGF And Factor V/VIII Domain Containing	Protein Coding	45	GC15M088898	0.344350338
MYF5	Myogenic Factor 5	Protein Coding	41	GC12P080716	0.344350338
NFYB	Nuclear Transcription Factor Y Subunit Beta	Protein Coding	41	GC12M104117	0.344350338
NLRP12	NLR Family Pyrin Domain Containing 12	Protein Coding	44	GC19M053793	0.344350338
PAK5	P21 (RAC1) Activated Kinase 5	Protein Coding	37	GC20M009538	0.344350338
PAK6	P21 (RAC1) Activated Kinase 6	Protein Coding	45	GC15P040217	0.344350338

PGAP1	Post-GPI Attachment To Proteins Inositol Deacylase 1	Protein Coding	41	GC02M196833	0.344350338
PIP4P1	Phosphatidylinositol-4,5-Bisphosphate 4-Phosphatase 1	Protein Coding	29	GC14M020458	0.344350338
PYCR1	Pyrroline-5-Carboxylate Reductase 1	Protein Coding	48	GC17M081932	0.344350338
REN	Renin	Protein Coding	48	GC01M204154	0.344350338
REST	RE1 Silencing Transcription Factor	Protein Coding	45	GC04P056907	0.344350338
SNRPB	Small Nuclear Ribonucleoprotein Polypeptides B And B1	Protein Coding	45	GC20M002461	0.344350338
SREK1	Splicing Regulatory Glutamic Acid And Lysine Rich Protein 1	Protein Coding	38	GC05P066139	0.344350338
SRSF11	Serine And Arginine Rich Splicing Factor 11	Protein Coding	38	GC01P070206	0.344350338
UBTF	Upstream Binding Transcription Factor	Protein Coding	44	GC17M044205	0.344350338
SLBP	Stem-Loop Binding Protein	Protein Coding	36	GC04M001692	0.33837533
AFF1	AF4/FMR2 Family Member 1	Protein Coding	39	GC04P086934	0.335658878
AMPH	Amphiphysin	Protein Coding	45	GC07M038782	0.335658878
ANAPC4	Anaphase Promoting Complex Subunit 4	Protein Coding	36	GC04P025379	0.335658878
APIP	APAF1 Interacting Protein	Protein Coding	41	GC11M034854	0.335658878
BAP1	BRCA1 Associated Protein 1	Protein Coding	47	GC03M052401	0.335658878
CBX4	Chromobox 4	Protein Coding	43	GC17M079833	0.335658878
CDC16	Cell Division Cycle 16	Protein Coding	40	GC13P114234	0.335658878
CEP63	Centrosomal Protein 63	Protein Coding	41	GC03P134485	0.335658878

CHAF1B	Chromatin Assembly Factor 1 Subunit B	Protein Coding	41	GC21P036385	0.335658878
DCX	Doublecortin	Protein Coding	46	GC0XM111293	0.335658878
ECT2	Epithelial Cell Transforming 2	Protein Coding	41	GC03P172750	0.335658878
EIF4ENIF1	Eukaryotic Translation Initiation Factor 4E Nuclear Import Factor 1	Protein Coding	37	GC22M031436	0.335658878
GBF1	Golgi Brefeldin A Resistant Guanine Nucleotide Exchange Factor 1	Protein Coding	44	GC10P102245	0.335658878
IFNE	Interferon Epsilon	Protein Coding	35	GC09M021480	0.335658878
KLHL9	Kelch Like Family Member 9	Protein Coding	41	GC09M021329	0.335658878
MAD2L1	Mitotic Arrest Deficient 2 Like 1	Protein Coding	46	GC04M120055	0.335658878
MEF2A	Myocyte Enhancer Factor 2A	Protein Coding	48	GC15P099565	0.335658878
MEF2D	Myocyte Enhancer Factor 2D	Protein Coding	45	GC01M156463	0.335658878
PIAS1	Protein Inhibitor Of Activated STAT 1	Protein Coding	45	GC15P068054	0.335658878
PIAS2	Protein Inhibitor Of Activated STAT 2	Protein Coding	44	GC18M046808	0.335658878
PIAS3	Protein Inhibitor Of Activated STAT 3	Protein Coding	41	GC01M145848	0.335658878
PIAS4	Protein Inhibitor Of Activated STAT 4	Protein Coding	43	GC19P004007	0.335658878
PLAA	Phospholipase A2 Activating Protein	Protein Coding	45	GC09M026903	0.335658878
PML	PML Nuclear Body Scaffold	Protein Coding	46	GC15P073994	0.335658878
PPP1R10	Protein Phosphatase 1 Regulatory Subunit 10	Protein Coding	39	GC06M030600	0.335658878
PRDM1	PR/SET Domain 1	Protein Coding	45	GC06P105993	0.335658878

RACGAP1	Rac GTPase Activating Protein 1	Protein Coding	43	GC12M049978	0.335658878
RNF4	Ring Finger Protein 4	Protein Coding	41	GC04P002462	0.335658878
SLC23A4P	Solute Carrier Family 23 Member 4, Pseudogene 4	Pseudogene	7	GC07M135247	0.335658878
SMO	Smoothened, Frizzled Class Receptor	Protein Coding	50	GC07P130058	0.335658878
TRPM8	Transient Receptor Potential Cation Channel Subfamily M Member 8	Protein Coding	44	GC02P233917	0.335658878
TTF1	Transcription Termination Factor 1	Protein Coding	38	GC09M132375	0.335658878
TUSC1	Tumor Suppressor Candidate 1	Protein Coding	30	GC09M025668	0.335658878
WWC1	WW And C2 Domain Containing 1	Protein Coding	43	GC05P168291	0.335658878
XPO1	Exportin 1	Protein Coding	47	GC02M061445	0.335658878
ETF1	Eukaryotic Translation Termination Factor 1	Protein Coding	44	GC05M138506	0.321011037
AARS1	Alanyl-TRNA Synthetase 1	Protein Coding	37	GC16M070343	0.314094067
ACACA	Acetyl-CoA Carboxylase Alpha	Protein Coding	50	GC17M037084	0.314094067
ALDOA	Aldolase, Fructose-Bisphosphate A	Protein Coding	49	GC16P030064	0.314094067
ALPG	Alkaline Phosphatase, Germ Cell	Protein Coding	33	GC02P232407	0.314094067
ANXA2	Annexin A2	Protein Coding	49	GC15M060347	0.314094067
ANXA4	Annexin A4	Protein Coding	45	GC02P069644	0.314094067
ASNS	Asparagine Synthetase (Glutamine-Hydrolyzing)	Protein Coding	47	GC07M097854	0.314094067
BRCA2	BRCA2 DNA Repair Associated	Protein Coding	50	GC13P032315	0.314094067

CAMK2A	Calcium/Calmodulin Dependent Protein Kinase II Alpha	Protein Coding	50	GC05M150219	0.314094067
CARS1	Cysteinyl-TRNA Synthetase 1	Protein Coding	34	GC11M003000	0.314094067
CCL26	C-C Motif Chemokine Ligand 26	Protein Coding	38	GC07M075769	0.314094067
CDK11A	Cyclin Dependent Kinase 11A	Protein Coding	38	GC01M001702	0.314094067
CDK11B	Cyclin Dependent Kinase 11B	Protein Coding	38	GC01M002721	0.314094067
CHD4	Chromodomain Helicase DNA Binding Protein 4	Protein Coding	45	GC12M006570	0.314094067
COX4I1	Cytochrome C Oxidase Subunit 4I1	Protein Coding	47	GC16P085798	0.314094067
CSNK2B	Casein Kinase 2 Beta	Protein Coding	50	GC06P055205	0.314094067
DDR1	Discoidin Domain Receptor Tyrosine Kinase 1	Protein Coding	47	GC06P055184	0.314094067
DDX1	DEAD-Box Helicase 1	Protein Coding	44	GC02P015591	0.314094067
DHX15	DEAH-Box Helicase 15	Protein Coding	38	GC04M024519	0.314094067
DIABLO	Diablo IAP-Binding Mitochondrial Protein	Protein Coding	48	GC12M122208	0.314094067
DIO2	Iodothyronine Deiodinase 2	Protein Coding	41	GC14M080197	0.314094067
EIF4B	Eukaryotic Translation Initiation Factor 4B	Protein Coding	43	GC12P053006	0.314094067
EPRS1	Glutamyl-Prolyl-TRNA Synthetase 1	Protein Coding	37	GC01M219969	0.314094067
ERCC2	ERCC Excision Repair 2, TFIIH Core Complex Helicase Subunit	Protein Coding	48	GC19M045349	0.314094067
ERCC3	ERCC Excision Repair 3, TFIIH Core Complex Helicase Subunit	Protein Coding	48	GC02M127257	0.314094067

EWSR1	EWS RNA Binding Protein 1	Protein Coding	44	GC22P029269	0.314094067
FAM20B	FAM20B Glycosaminoglycan Xylosylkinase	Protein Coding	37	GC01P179025	0.314094067
FCER2	Fc Fragment Of IgE Receptor II	Protein Coding	45	GC19M007689	0.314094067
FST	Follistatin	Protein Coding	47	GC05P053480	0.314094067
FXN	Frataxin	Protein Coding	47	GC09P069035	0.314094067
GARS1	Glycyl-TRNA Synthetase 1	Protein Coding	37	GC07P030580	0.314094067
GET3	Guided Entry Of Tail-Anchored Proteins Factor 3, ATPase	Protein Coding	34	GC19P012737	0.314094067
GTF2F2	General Transcription Factor IIF Subunit 2	Protein Coding	43	GC13P045120	0.314094067
GYPC	Glycophorin C (Gerbich Blood Group)	Protein Coding	44	GC02P126655	0.314094067
H2BS1	H2B.S Histone 1	Protein Coding	23	GC21P043567	0.314094067
HSPA14	Heat Shock Protein Family A (Hsp70) Member 14	Protein Coding	40	GC10P014847	0.314094067
IDE	Insulin Degrading Enzyme	Protein Coding	49	GC10M092451	0.314094067
INO80	INO80 Complex ATPase Subunit	Protein Coding	37	GC15M040979	0.314094067
INTS3	Integrator Complex Subunit 3	Protein Coding	37	GC01P153728	0.314094067
JAK1	Janus Kinase 1	Protein Coding	54	GC01M064833	0.314094067
KARS1	Lysyl-TRNA Synthetase 1	Protein Coding	38	GC16M075628	0.314094067
KCNN4	Potassium Calcium-Activated Channel Subfamily N Member 4	Protein Coding	49	GC19M046911	0.314094067
KIF5B	Kinesin Family Member 5B	Protein Coding	45	GC10M032035	0.314094067
KRT17	Keratin 17	Protein Coding	47	GC17M041619	0.314094067
LATS1	Large Tumor Suppressor Kinase 1	Protein Coding	46	GC06M149658	0.314094067

LATS2	Large Tumor Suppressor Kinase 2	Protein Coding	46	GC13M020973	0.314094067
MAP2K7	Mitogen-Activated Protein Kinase Kinase 7	Protein Coding	47	GC19P007903	0.314094067
MAT2B	Methionine Adenosyltransferase 2B	Protein Coding	41	GC05P163504	0.314094067
MCM3	Minichromosome Maintenance Complex Component 3	Protein Coding	46	GC06M052264	0.314094067
MT-CO3	Mitochondrially Encoded Cytochrome C Oxidase III	Protein Coding	31	GCMTP009209	0.314094067
MTREX	Mtr4 Exosome RNA Helicase	Protein Coding	30	GC05P055308	0.314094067
MVP	Major Vault Protein	Protein Coding	41	GC16P032377	0.314094067
MYO6	Myosin VI	Protein Coding	47	GC06P075749	0.314094067
NEDD4L	NEDD4 Like E3 Ubiquitin Protein Ligase	Protein Coding	47	GC18P058044	0.314094067
NEK3	NIMA Related Kinase 3	Protein Coding	43	GC13M052132	0.314094067
NPPC	Natriuretic Peptide C	Protein Coding	41	GC02M231921	0.314094067
NR4A1	Nuclear Receptor Subfamily 4 Group A Member 1	Protein Coding	47	GC12P052022	0.314094067
NUMA1	Nuclear Mitotic Apparatus Protein 1	Protein Coding	44	GC11M072002	0.314094067
OPLAH	5-Oxoprolinase, ATP-Hydrolysing	Protein Coding	42	GC08M144051	0.314094067
P2RX6	Purinergic Receptor P2X 6	Protein Coding	43	GC22P021183	0.314094067
PDGFA	Platelet Derived Growth Factor Subunit A	Protein Coding	44	GC07M000497	0.314094067
PDIA3	Protein Disulfide Isomerase Family A Member 3	Protein Coding	46	GC15P043746	0.314094067
PDYN	Prodynorphin	Protein Coding	44	GC20M001978	0.314094067

PFKFB2	6-Phosphofructo-2-Kinase/Fructose-2,6-Biphosphatase 2	Protein Coding	41	GC01P207034	0.314094067
PLK2	Polo Like Kinase 2	Protein Coding	44	GC05M058453	0.314094067
POLR2H	RNA Polymerase II, I And III Subunit H	Protein Coding	41	GC03P184361	0.314094067
POU2F1	POU Class 2 Homeobox 1	Protein Coding	45	GC01P167190	0.314094067
PPP1R12A	Protein Phosphatase 1 Regulatory Subunit 12A	Protein Coding	44	GC12M079773	0.314094067
PRDX4	Peroxiredoxin 4	Protein Coding	44	GC0XP023665	0.314094067
PRDX6	Peroxiredoxin 6	Protein Coding	47	GC01P173477	0.314094067
PRKAR1A	Protein Kinase CAMP-Dependent Type I Regulatory Subunit Alpha	Protein Coding	52	GC17P068545	0.314094067
PRKG2	Protein Kinase CGMP-Dependent 2	Protein Coding	47	GC04M081087	0.314094067
PSAP	Prosaposin	Protein Coding	47	GC10M071816	0.314094067
PSMC3	Proteasome 26S Subunit, ATPase 3	Protein Coding	43	GC11M069001	0.314094067
PSMC4	Proteasome 26S Subunit, ATPase 4	Protein Coding	41	GC19P040529	0.314094067
RARA	Retinoic Acid Receptor Alpha	Protein Coding	51	GC17P040309	0.314094067
REL	REL Proto-Oncogene, NF-KB Subunit	Protein Coding	47	GC02P060881	0.314094067
RHD	Rh Blood Group D Antigen	Protein Coding	41	GC01P025272	0.314094067
RIPK2	Receptor Interacting Serine/Threonine Kinase 2	Protein Coding	47	GC08P089758	0.314094067
RPLP0	Ribosomal Protein Lateral Stalk Subunit P0	Protein Coding	42	GC12M120196	0.314094067
RUVBL1	RuvB Like AAA ATPase 1	Protein Coding	45	GC03M128064	0.314094067
SARS1	Seryl-TRNA Synthetase 1	Protein Coding	36	GC01P109214	0.314094067

SLC2A1	Solute Carrier Family 2 Member 1	Protein Coding	53	GC01M042925	0.314094067
SLC2A5	Solute Carrier Family 2 Member 5	Protein Coding	44	GC01M009036	0.314094067
SLC4A1	Solute Carrier Family 4 Member 1 (Diego Blood Group)	Protein Coding	48	GC17M044355	0.314094067
STK16	Serine/Threonine Kinase 16	Protein Coding	41	GC02P219248	0.314094067
TCP1	T-Complex 1	Protein Coding	41	GC06M159778	0.314094067
TGFBR1	Transforming Growth Factor Beta Receptor 1	Protein Coding	54	GC09P099104	0.314094067
TLR2	Toll Like Receptor 2	Protein Coding	52	GC04P153684	0.314094067
TOP2A	DNA Topoisomerase II Alpha	Protein Coding	51	GC17M040388	0.314094067
TRPM7	Transient Receptor Potential Cation Channel Subfamily M Member 7	Protein Coding	46	GC15M050552	0.314094067
TUBB	Tubulin Beta Class I	Protein Coding	50	GC06P055181	0.314094067
UBA1	Ubiquitin Like Modifier Activating Enzyme 1	Protein Coding	47	GC0XP047190	0.314094067
VASP	Vasodilator Stimulated Phosphoprotein	Protein Coding	44	GC19P045507	0.314094067
XPA	XPA, DNA Damage Recognition And Repair Factor	Protein Coding	47	GC09M097654	0.314094067
XPC	XPC Complex Subunit, DNA Damage Recognition And Repair Factor	Protein Coding	46	GC03M016919	0.314094067
TRA2B	Transformer 2 Beta Homolog	Protein Coding	40	GC03M185914	0.302652091
ABCA1	ATP Binding Cassette Subfamily A Member 1	Protein Coding	48	GC09M104781	0.292870075

RPS6KA5	Ribosomal Protein S6 Kinase A5	Protein Coding	46	GC14M090847	0.292870075
ADM	Adrenomedullin	Protein Coding	45	GC11P010304	0.292870075
AGPS	Alkylglycerone Phosphate Synthase	Protein Coding	44	GC02P177392	0.292870075
AHSP	Alpha Hemoglobin Stabilizing Protein	Protein Coding	36	GC16P031527	0.292870075
AIFM2	Apoptosis Inducing Factor Mitochondria Associated 2	Protein Coding	41	GC10M070098	0.292870075
AIFM3	Apoptosis Inducing Factor Mitochondria Associated 3	Protein Coding	38	GC22P020965	0.292870075
AKAP1	A-Kinase Anchoring Protein 1	Protein Coding	40	GC17P057085	0.292870075
AKAP5	A-Kinase Anchoring Protein 5	Protein Coding	40	GC14P064465	0.292870075
ALDH1A2	Aldehyde Dehydrogenase 1 Family Member A2	Protein Coding	48	GC15M067289	0.292870075
ALOX12	Arachidonate 12-Lipoxygenase, 12S Type	Protein Coding	44	GC17P006995	0.292870075
CA2	Carbonic Anhydrase 2	Protein Coding	52	GC08P085463	0.292870075
CAMK1	Calcium/Calmodulin Dependent Protein Kinase I	Protein Coding	45	GC03M009774	0.292870075
CAMK4	Calcium/Calmodulin Dependent Protein Kinase IV	Protein Coding	47	GC05P111223	0.292870075
CAMKK2	Calcium/Calmodulin Dependent Protein Kinase Kinase 2	Protein Coding	47	GC12M121496	0.292870075
CAST	Calpastatin	Protein Coding	46	GC05P096525	0.292870075
CHDH	Choline Dehydrogenase	Protein Coding	41	GC03M053812	0.292870075
CHRM3	Cholinergic Receptor Muscarinic 3	Protein Coding	50	GC01P239386	0.292870075
COQ6	Coenzyme Q6, Monooxygenase	Protein Coding	43	GC14P073949	0.292870075

CRY1	Cryptochrome Circadian Regulator 1	Protein Coding	44	GC12M106991	0.292870075
CRY2	Cryptochrome Circadian Regulator 2	Protein Coding	42	GC11P046088	0.292870075
CRYL1	Crystallin Lambda 1	Protein Coding	38	GC13M020403	0.292870075
CYB5R1	Cytochrome B5 Reductase 1	Protein Coding	42	GC01M202964	0.292870075
CYB5R2	Cytochrome B5 Reductase 2	Protein Coding	42	GC11M007665	0.292870075
CYB5R4	Cytochrome B5 Reductase 4	Protein Coding	41	GC06P083859	0.292870075
CYB5RL	Cytochrome B5 Reductase Like	Protein Coding	30	GC01M054172	0.292870075
CYP24A1	Cytochrome P450 Family 24 Subfamily A Member 1	Protein Coding	48	GC20M054153	0.292870075
CYP27B1	Cytochrome P450 Family 27 Subfamily B Member 1	Protein Coding	48	GC12M057757	0.292870075
D2HGDH	D-2-Hydroxyglutarate Dehydrogenase	Protein Coding	41	GC02P241734	0.292870075
DHCR24	24-Dehydrocholesterol Reductase	Protein Coding	46	GC01M054849	0.292870075
DMGDH	Dimethylglycine Dehydrogenase	Protein Coding	44	GC05M078997	0.292870075
DUOX1	Dual Oxidase 1	Protein Coding	42	GC15P045129	0.292870075
DUOX2	Dual Oxidase 2	Protein Coding	44	GC15M045092	0.292870075
DUS1L	Dihydrouridine Synthase 1 Like	Protein Coding	37	GC17M082057	0.292870075
DUS2	Dihydrouridine Synthase 2	Protein Coding	37	GC16P067987	0.292870075
DUS3L	Dihydrouridine Synthase 3 Like	Protein Coding	34	GC19M005786	0.292870075
DUS4L	Dihydrouridine Synthase 4 Like	Protein Coding	36	GC07P107563	0.292870075
EDN2	Endothelin 2	Protein Coding	41	GC01M041478	0.292870075
ERO1A	Endoplasmic Reticulum Oxidoreductase 1 Alpha	Protein Coding	35	GC14M052640	0.292870075
ERO1B	Endoplasmic Reticulum Oxidoreductase 1 Beta	Protein Coding	33	GC01M236216	0.292870075

ETFA	Electron Transfer Flavoprotein Subunit Alpha	Protein Coding	46	GC15M076215	0.292870075
ETFB	Electron Transfer Flavoprotein Subunit Beta	Protein Coding	45	GC19M051345	0.292870075
ETFDH	Electron Transfer Flavoprotein Dehydrogenase	Protein Coding	45	GC04P158672	0.292870075
EZR	Ezrin	Protein Coding	46	GC06M158765	0.292870075
FDXR	Ferredoxin Reductase	Protein Coding	45	GC17M074862	0.292870075
FGR	FGR Proto-Oncogene, Src Family Tyrosine Kinase	Protein Coding	48	GC01M027622	0.292870075
FMO1	Flavin Containing Dimethylaniline Monoxygenase 1	Protein Coding	44	GC01P171248	0.292870075
FMO2	Flavin Containing Dimethylaniline Monoxygenase 2	Protein Coding	41	GC01P171185	0.292870075
FMO4	Flavin Containing Dimethylaniline Monoxygenase 4	Protein Coding	42	GC01P171315	0.292870075
FMO5	Flavin Containing Dimethylaniline Monoxygenase 5	Protein Coding	43	GC01M147175	0.292870075
FMO6P	Flavin Containing Dimethylaniline Monoxygenase 6, Pseudogene	Pseudogene	22	GC01P171106	0.292870075
FOXRED1	FAD Dependent Oxidoreductase Domain Containing 1	Protein Coding	40	GC11P126269	0.292870075
FOXRED2	FAD Dependent Oxidoreductase Domain Containing 2	Protein Coding	34	GC22M036487	0.292870075
GCGR	Glucagon Receptor	Protein Coding	48	GC17P081804	0.292870075

GDNF	Glial Cell Derived Neurotrophic Factor	Protein Coding	48	GC05M037812	0.292870075
GFER	Growth Factor, Augmenter Of Liver Regeneration	Protein Coding	45	GC16P001984	0.292870075
GPD2	Glycerol-3-Phosphate Dehydrogenase 2	Protein Coding	47	GC02P156435	0.292870075
GRK2	G Protein-Coupled Receptor Kinase 2	Protein Coding	38	GC11P067266	0.292870075
HBEGF	Heparin Binding EGF Like Growth Factor	Protein Coding	44	GC05M140332	0.292870075
HK2	Hexokinase 2	Protein Coding	48	GC02P074833	0.292870075
HMOX2	Heme Oxygenase 2	Protein Coding	48	GC16P004474	0.292870075
HNF4A	Hepatocyte Nuclear Factor 4 Alpha	Protein Coding	51	GC20P044355	0.292870075
HTR1B	5-Hydroxytryptamine Receptor 1B	Protein Coding	45	GC06M077478	0.292870075
IAPP	Islet Amyloid Polypeptide	Protein Coding	42	GC12P021354	0.292870075
KDM1B	Lysine Demethylase 1B	Protein Coding	37	GC06P018156	0.292870075
KMO	Kynurenine 3-Monooxygenase	Protein Coding	45	GC01P241532	0.292870075
L2HGDH	L-2-Hydroxyglutarate Dehydrogenase	Protein Coding	41	GC14M050237	0.292870075
LPAR3	Lysophosphatidic Acid Receptor 3	Protein Coding	44	GC01M084811	0.292870075
MAP3K1	Mitogen-Activated Protein Kinase Kinase Kinase 1	Protein Coding	50	GC05P056815	0.292870075
MAP3K12	Mitogen-Activated Protein Kinase Kinase Kinase 12	Protein Coding	46	GC12M053479	0.292870075
MICAL1	Microtubule Associated Monooxygenase, Calponin And LIM Domain Containing 1	Protein Coding	41	GC06M109444	0.292870075

MICAL2	Microtubule Associated Monooxygenase, Calponin And LIM Domain Containing 2	Protein Coding	38	GC11P012115	0.292870075
MICAL3	Microtubule Associated Monooxygenase, Calponin And LIM Domain Containing 3	Protein Coding	34	GC22M017794	0.292870075
MSBP1	Minisatellite Binding Protein 1	Protein Coding	5	GC00U990213	0.292870075
MT-ND2	Mitochondrially Encoded NADH:Ubiquinone Oxidoreductase Core Subunit 2	Protein Coding	34	GCMTP004472	0.292870075
MTO1	Mitochondrial TRNA Translation Optimization 1	Protein Coding	43	GC06P073461	0.292870075
NDOR1	NADPH Dependent Diflavin Oxidoreductase 1	Protein Coding	40	GC09P137205	0.292870075
NDUFA1	NADH:Ubiquinone Oxidoreductase Subunit A1	Protein Coding	45	GC0XP119871	0.292870075
NDUFA10	NADH:Ubiquinone Oxidoreductase Subunit A10	Protein Coding	45	GC02M239893	0.292870075
NDUFA11	NADH:Ubiquinone Oxidoreductase Subunit A11	Protein Coding	38	GC19M005891	0.292870075
NDUFA12	NADH:Ubiquinone Oxidoreductase Subunit A12	Protein Coding	45	GC12M094898	0.292870075
NDUFA13	NADH:Ubiquinone Oxidoreductase Subunit A13	Protein Coding	45	GC19P019515	0.292870075
NDUFA2	NADH:Ubiquinone Oxidoreductase Subunit A2	Protein Coding	43	GC05M140653	0.292870075
NDUFA3	NADH:Ubiquinone Oxidoreductase Subunit A3	Protein Coding	39	GC19P054102	0.292870075
NDUFA4	NDUFA4 Mitochondrial Complex Associated	Protein Coding	44	GC07M010938	0.292870075

NDUFA4L2	NDUFA4 Mitochondrial Complex Associated Like 2	Protein Coding	37	GC12M057234	0.292870075
NDUFA6	NADH:Ubiquinone Oxidoreductase Subunit A6	Protein Coding	45	GC22M042085	0.292870075
NDUFA7	NADH:Ubiquinone Oxidoreductase Subunit A7	Protein Coding	38	GC19M008308	0.292870075
NDUFA8	NADH:Ubiquinone Oxidoreductase Subunit A8	Protein Coding	43	GC09M122132	0.292870075
NDUFA9	NADH:Ubiquinone Oxidoreductase Subunit A9	Protein Coding	44	GC12P004649	0.292870075
NDUFAB1	NADH:Ubiquinone Oxidoreductase Subunit AB1	Protein Coding	41	GC16M023582	0.292870075
NDUFB1	NADH:Ubiquinone Oxidoreductase Subunit B1	Protein Coding	37	GC14M092116	0.292870075
NDUFB11	NADH:Ubiquinone Oxidoreductase Subunit B11	Protein Coding	40	GC0XM047142	0.292870075
NDUFB2	NADH:Ubiquinone Oxidoreductase Subunit B2	Protein Coding	40	GC07P140690	0.292870075
NDUFB3	NADH:Ubiquinone Oxidoreductase Subunit B3	Protein Coding	43	GC02P201071	0.292870075
NDUFB4	NADH:Ubiquinone Oxidoreductase Subunit B4	Protein Coding	41	GC03P120596	0.292870075
NDUFB5	NADH:Ubiquinone Oxidoreductase Subunit B5	Protein Coding	37	GC03P179604	0.292870075
NDUFB7	NADH:Ubiquinone Oxidoreductase Subunit B7	Protein Coding	40	GC19M014566	0.292870075
NDUFB9	NADH:Ubiquinone Oxidoreductase Subunit B9	Protein Coding	45	GC08P124539	0.292870075
NDUFC2	NADH:Ubiquinone Oxidoreductase Subunit C2	Protein Coding	41	GC11M078068	0.292870075
NDUFS1	NADH:Ubiquinone Oxidoreductase Core Subunit S1	Protein Coding	47	GC02M206114	0.292870075

NDUFS2	NADH:Ubiquinone Oxidoreductase Core Subunit S2	Protein Coding	45	GC01P161197	0.292870075
NDUFS3	NADH:Ubiquinone Oxidoreductase Core Subunit S3	Protein Coding	48	GC11P047567	0.292870075
NDUFS5	NADH:Ubiquinone Oxidoreductase Subunit S5	Protein Coding	41	GC01P039026	0.292870075
NDUFS6	NADH:Ubiquinone Oxidoreductase Subunit S6	Protein Coding	45	GC05P001801	0.292870075
NDUFS7	NADH:Ubiquinone Oxidoreductase Core Subunit S7	Protein Coding	47	GC19P001635	0.292870075
NDUFS8	NADH:Ubiquinone Oxidoreductase Core Subunit S8	Protein Coding	46	GC11P068030	0.292870075
NDUFV1	NADH:Ubiquinone Oxidoreductase Core Subunit V1	Protein Coding	47	GC11P067670	0.292870075
NDUFV2	NADH:Ubiquinone Oxidoreductase Core Subunit V2	Protein Coding	45	GC18P009092	0.292870075
NOX1	NADPH Oxidase 1	Protein Coding	42	GC0XM100843	0.292870075
NOX3	NADPH Oxidase 3	Protein Coding	40	GC06M155395	0.292870075
NOX4	NADPH Oxidase 4	Protein Coding	43	GC11M089324	0.292870075
NOX5	NADPH Oxidase 5	Protein Coding	38	GC15P077106	0.292870075
NPTXR	Neuronal Pentraxin Receptor	Protein Coding	40	GC22M038818	0.292870075
NTRK2	Neurotrophic Receptor Tyrosine Kinase 2	Protein Coding	53	GC09P084668	0.292870075
OXNAD1	Oxidoreductase NAD Binding Domain Containing 1	Protein Coding	36	GC03P016266	0.292870075
PAOX	Polyamine Oxidase	Protein Coding	40	GC10P133379	0.292870075
PCYOX1	Prenylcysteine Oxidase 1	Protein Coding	37	GC02P070257	0.292870075
PCYOX1L	Prenylcysteine Oxidase 1 Like	Protein Coding	35	GC05P149358	0.292870075
PDP2	Pyruvate Dehyrogenase Phosphatase Catalytic Subunit 2	Protein Coding	39	GC16P066881	0.292870075

PIPOX	Pipecolic Acid And Sarcosine Oxidase	Protein Coding	40	GC17P029371	0.292870075
PPBP	Pro-Platelet Basic Protein	Protein Coding	44	GC04M073986	0.292870075
PPOX	Protoporphyrinogen Oxidase	Protein Coding	44	GC01P161184	0.292870075
PRKACB	Protein Kinase CAMP-Activated Catalytic Subunit Beta	Protein Coding	50	GC01P084078	0.292870075
PRKACG	Protein Kinase CAMP-Activated Catalytic Subunit Gamma	Protein Coding	48	GC09M069013	0.292870075
PRKX	Protein Kinase X-Linked	Protein Coding	42	GC0XM003604	0.292870075
PRKY	Protein Kinase Y-Linked (Pseudogene)	Pseudogene	25	GC0YP007273	0.292870075
PRODH2	Proline Dehydrogenase 2	Protein Coding	36	GC19M035799	0.292870075
PYROXD1	Pyridine Nucleotide-Disulphide Oxidoreductase Domain 1	Protein Coding	37	GC12P021437	0.292870075
PYY	Peptide YY	Protein Coding	43	GC17M043952	0.292870075
QSOX1	Quiescin Sulfhydryl Oxidase 1	Protein Coding	38	GC01P180154	0.292870075
QSOX2	Quiescin Sulfhydryl Oxidase 2	Protein Coding	36	GC09M136206	0.292870075
RARS1	Arginyl-TRNA Synthetase 1	Protein Coding	37	GC05P168487	0.292870075
RETSAT	Retinol Saturase	Protein Coding	38	GC02M085344	0.292870075
RNLS	Renalase, FAD Dependent Amine Oxidase	Protein Coding	39	GC10M088180	0.292870075
SARDH	Sarcosine Dehydrogenase	Protein Coding	43	GC09M133663	0.292870075
SC5D	Sterol-C5-Desaturase	Protein Coding	40	GC11P121292	0.292870075
SCARB1	Scavenger Receptor Class B Member 1	Protein Coding	46	GC12M124776	0.292870075
SCG2	Secretogranin II	Protein Coding	40	GC02M223596	0.292870075

SDHA	Succinate Dehydrogenase Complex Flavoprotein Subunit A	Protein Coding	48	GC05P000208	0.292870075
SDHC	Succinate Dehydrogenase Complex Subunit C	Protein Coding	45	GC01P161314	0.292870075
SDHD	Succinate Dehydrogenase Complex Subunit D	Protein Coding	45	GC11P112087	0.292870075
SFTPB	Surfactant Protein B	Protein Coding	44	GC02M085657	0.292870075
SGK1	Serum/Glucocorticoid Regulated Kinase 1	Protein Coding	50	GC06M134169	0.292870075
SLC5A1	Solute Carrier Family 5 Member 1	Protein Coding	50	GC22P032043	0.292870075
SMARCA4	SWI/SNF Related, Matrix Associated, Actin Dependent Regulator Of Chromatin, Subfamily A, Member 4	Protein Coding	50	GC19P010932	0.292870075
SMOX	Spermine Oxidase	Protein Coding	39	GC20P004120	0.292870075
SQLE	Squalene Epoxidase	Protein Coding	44	GC08P124998	0.292870075
SQOR	Sulfide Quinone Oxidoreductase	Protein Coding	30	GC15P045632	0.292870075
STAR	Steroidogenic Acute Regulatory Protein	Protein Coding	47	GC08M038145	0.292870075
STEAP1	STEAP Family Member 1	Protein Coding	40	GC07P090154	0.292870075
STEAP2	STEAP2 Metalloreductase	Protein Coding	41	GC07P090167	0.292870075
STEAP3	STEAP3 Metalloreductase	Protein Coding	45	GC02P119222	0.292870075
STEAP4	STEAP4 Metalloreductase	Protein Coding	41	GC07M088315	0.292870075
TAP2	Transporter 2, ATP Binding Cassette Subfamily B Member	Protein Coding	45	GC06M032821	0.292870075

TBXA2R	Thromboxane A2 Receptor	Protein Coding	49	GC19M003594	0.292870075
TPR	Translocated Promoter Region, Nuclear Basket Protein	Protein Coding	43	GC01M186319	0.292870075
TRH	Thyrotropin Releasing Hormone	Protein Coding	44	GC03P129974	0.292870075
UGT1A4	UDP Glucuronosyltransferase Family 1 Member A4	Protein Coding	42	GC02P233718	0.292870075
VCL	Vinculin	Protein Coding	48	GC10P073995	0.292870075
ANG	Angiogenin	Protein Coding	46	GC14P021583	0.283105105
CSDE1	Cold Shock Domain Containing E1	Protein Coding	39	GC01M114716	0.283105105
NR4A2	Nuclear Receptor Subfamily 4 Group A Member 2	Protein Coding	48	GC02M156324	0.283105105
HBB-LCR	Beta-Globin Locus Control Region	Biological Region	5	GC11P005270	0.262104422
OMP	Olfactory Marker Protein	Protein Coding	37	GC11P077102	0.262104422
PABPC1	Poly(A) Binding Protein Cytoplasmic 1	Protein Coding	43	GC08M100685	0.262104422
SRRM1	Serine And Arginine Repetitive Matrix 1	Protein Coding	38	GC01P024631	0.262104422
ETS2	ETS Proto-Oncogene 2, Transcription Factor	Protein Coding	43	GC21P038805	0.239267498
SRSF5	Serine And Arginine Rich Splicing Factor 5	Protein Coding	40	GC14P069727	0.239267498
SRSF6	Serine And Arginine Rich Splicing Factor 6	Protein Coding	40	GC20P043457	0.239267498
ANAPC11	Anaphase Promoting Complex Subunit 11	Protein Coding	40	GC17P081890	0.237346679

APBB1	Amyloid Beta Precursor Protein Binding Family B Member 1	Protein Coding	44	GC11M006396	0.237346679
APOBEC3F	Apolipoprotein B MRNA Editing Enzyme Catalytic Subunit 3F	Protein Coding	38	GC22P039030	0.237346679
ARHGDIB	Rho GDP Dissociation Inhibitor Beta	Protein Coding	44	GC12M014942	0.237346679
ARNTL	Aryl Hydrocarbon Receptor Nuclear Translocator Like	Protein Coding	43	GC11P013276	0.237346679
BACE1	Beta-Secretase 1	Protein Coding	47	GC11M117285	0.237346679
BUB3	BUB3 Mitotic Checkpoint Protein	Protein Coding	48	GC10P123154	0.237346679
CACNA1A	Calcium Voltage-Gated Channel Subunit Alpha1 A	Protein Coding	47	GC19M013206	0.237346679
CASP10	Caspase 10	Protein Coding	48	GC02P201182	0.237346679
CCL2	C-C Motif Chemokine Ligand 2	Protein Coding	49	GC17P034255	0.237346679
CCNL1	Cyclin L1	Protein Coding	39	GC03M157146	0.237346679
CD69	CD69 Molecule	Protein Coding	41	GC12M015678	0.237346679
CDC14A	Cell Division Cycle 14A	Protein Coding	44	GC01P100351	0.237346679
CDC14B	Cell Division Cycle 14B	Protein Coding	41	GC09M096490	0.237346679
CDC27	Cell Division Cycle 27	Protein Coding	42	GC17M047117	0.237346679
CDCA5	Cell Division Cycle Associated 5	Protein Coding	38	GC11M069368	0.237346679
CDKN1C	Cyclin Dependent Kinase Inhibitor 1C	Protein Coding	47	GC11M002894	0.237346679
CDKN2C	Cyclin Dependent Kinase Inhibitor 2C	Protein Coding	47	GC01P050960	0.237346679
CEBPZ	CCAAT Enhancer Binding Protein Zeta	Protein Coding	37	GC02M037201	0.237346679
CEP290	Centrosomal Protein 290	Protein Coding	41	GC12M088049	0.237346679

CHTF8	Chromosome Transmission Fidelity Factor 8	Protein Coding	33	GC16M069119	0.237346679
CKAP4	Cytoskeleton Associated Protein 4	Protein Coding	38	GC12M106237	0.237346679
CKS1B	CDC28 Protein Kinase Regulatory Subunit 1B	Protein Coding	43	GC01P154974	0.237346679
CLDN2	Claudin 2	Protein Coding	42	GC0XP106900	0.237346679
CXCL5	C-X-C Motif Chemokine Ligand 5	Protein Coding	41	GC04M073995	0.237346679
CYTH2	Cytohesin 2	Protein Coding	42	GC19P048470	0.237346679
DCN	Decorin	Protein Coding	47	GC12M091140	0.237346679
DCT	Dopachrome Tautomerase	Protein Coding	43	GC13M094436	0.237346679
DENR	Density Regulated Re-Initiation And Release Factor	Protein Coding	38	GC12P122752	0.237346679
DNM1	Dynamin 1	Protein Coding	51	GC09P128191	0.237346679
DPYSL2	Dihydropyrimidinase Like 2	Protein Coding	46	GC08P026514	0.237346679
DUSP6	Dual Specificity Phosphatase 6	Protein Coding	50	GC12M089347	0.237346679
E2F4	E2F Transcription Factor 4	Protein Coding	46	GC16P067192	0.237346679
EIF5A	Eukaryotic Translation Initiation Factor 5A	Protein Coding	44	GC17P007306	0.237346679
FBL	Fibrillarin	Protein Coding	44	GC19M039834	0.237346679
FOXP2	Forkhead Box P2	Protein Coding	44	GC07P114086	0.237346679
FTCD	Formimidoyltransferase Cyclodeaminase	Protein Coding	44	GC21M048321	0.237346679
FZR1	Fizzy And Cell Division Cycle 20 Related 1	Protein Coding	43	GC19P003506	0.237346679
GGA3	Golgi Associated, Gamma Adaptin Ear Containing, ARF Binding Protein 3	Protein Coding	41	GC17M075225	0.237346679
GLI1	GLI Family Zinc Finger 1	Protein Coding	48	GC12P057460	0.237346679

GTF2H1	General Transcription Factor IIH Subunit 1	Protein Coding	44	GC11P018323	0.237346679
HEXIM1	HEXIM P-TEFb Complex Subunit 1	Protein Coding	39	GC17P045147	0.237346679
HNRNPC	Heterogeneous Nuclear Ribonucleoprotein C	Protein Coding	42	GC14M021210	0.237346679
IGF1	Insulin Like Growth Factor 1	Protein Coding	50	GC12M102395	0.237346679
IMPA1	Inositol Monophosphatase 1	Protein Coding	49	GC08M081656	0.237346679
KRT9	Keratin 9	Protein Coding	40	GC17M041565	0.237346679
LAPTM4A	Lysosomal Protein Transmembrane 4 Alpha	Protein Coding	38	GC02M020032	0.237346679
LHX8	LIM Homeobox 8	Protein Coding	39	GC01P075128	0.237346679
LMAN1	Lectin, Mannose Binding 1	Protein Coding	46	GC18M059327	0.237346679
LOC111365141	NOS2 5’ Regulatory Region	Biological Region	2	GC17P027800	0.237346679
MAPK8IP3	Mitogen-Activated Protein Kinase 8 Interacting Protein 3	Protein Coding	41	GC16P001706	0.237346679
MAX	MYC Associated Factor X	Protein Coding	48	GC14M065009	0.237346679
MEF2C	Myocyte Enhancer Factor 2C	Protein Coding	50	GC05M088718	0.237346679
METAP2	Methionyl Aminopeptidase 2	Protein Coding	45	GC12P095473	0.237346679
MME	Membrane Metalloendopeptidase	Protein Coding	51	GC03P155024	0.237346679
MNAT1	MNAT1 Component Of CDK Activating Kinase	Protein Coding	43	GC14P060734	0.237346679
MSI1	Musashi RNA Binding Protein 1	Protein Coding	40	GC12M120341	0.237346679
MYOCD	Myocardin	Protein Coding	42	GC17P012665	0.237346679
MYOG	Myogenin	Protein Coding	40	GC01M203083	0.237346679

NDC80	NDC80 Kinetochore Complex Component	Protein Coding	42	GC18P002571	0.237346679
NEDD1	NEDD1 Gamma-Tubulin Ring Complex Targeting Factor	Protein Coding	37	GC12P096907	0.237346679
NFYC	Nuclear Transcription Factor Y Subunit Gamma	Protein Coding	43	GC01P040691	0.237346679
OPN4	Opsin 4	Protein Coding	41	GC10P086654	0.237346679
PA2G4	Proliferation-Associated 2G4	Protein Coding	41	GC12P056372	0.237346679
PDCD4	Programmed Cell Death 4	Protein Coding	43	GC10P110871	0.237346679
PER2	Period Circadian Regulator 2	Protein Coding	44	GC02M238244	0.237346679
PIN1	Peptidylprolyl Cis/Trans Isomerase, NIMA-Interacting 1	Protein Coding	48	GC19P009835	0.237346679
PPM1D	Protein Phosphatase, Mg2+/Mn2+ Dependent 1D	Protein Coding	48	GC17P060600	0.237346679
PSEN2	Presenilin 2	Protein Coding	50	GC01P226870	0.237346679
PTPN6	Protein Tyrosine Phosphatase Non-Receptor Type 6	Protein Coding	50	GC12P011869	0.237346679
RELA	RELA Proto-Oncogene, NF-KB Subunit	Protein Coding	51	GC11M065653	0.237346679
RGS4	Regulator Of G Protein Signaling 4	Protein Coding	44	GC01P163038	0.237346679
RPLP1	Ribosomal Protein Lateral Stalk Subunit P1	Protein Coding	41	GC15P077111	0.237346679
RPLP2	Ribosomal Protein Lateral Stalk Subunit P2	Protein Coding	42	GC11P000984	0.237346679
RPTOR	Regulatory Associated Protein Of MTOR Complex 1	Protein Coding	44	GC17P080544	0.237346679
SASH1	SAM And SH3 Domain Containing 1	Protein Coding	40	GC06P148193	0.237346679

SIRPA	Signal Regulatory Protein Alpha	Protein Coding	44	GC20P001894	0.237346679
SLC5A6	Solute Carrier Family 5 Member 6	Protein Coding	47	GC02M027201	0.237346679
SMAD2	SMAD Family Member 2	Protein Coding	48	GC18M047809	0.237346679
SMC1A	Structural Maintenance Of Chromosomes 1A	Protein Coding	47	GC0XM053374	0.237346679
SNAP91	Synaptosome Associated Protein 91	Protein Coding	40	GC06M083553	0.237346679
SOS1	SOS Ras/Rac Guanine Nucleotide Exchange Factor 1	Protein Coding	49	GC02M038981	0.237346679
STIL	STIL Centriolar Assembly Protein	Protein Coding	42	GC01M047250	0.237346679
STIM1	Stromal Interaction Molecule 1	Protein Coding	49	GC11P003855	0.237346679
STXBP1	Syntaxin Binding Protein 1	Protein Coding	49	GC09P127582	0.237346679
SYNPO	Synaptopodin	Protein Coding	38	GC05P150601	0.237346679
TAF1	TATA-Box Binding Protein Associated Factor 1	Protein Coding	46	GC0XP071366	0.237346679
TAGLN	Transgelin	Protein Coding	44	GC11P117199	0.237346679
TFDP1	Transcription Factor Dp-1	Protein Coding	45	GC13P113584	0.237346679
TFE3	Transcription Factor Binding To IGHM Enhancer 3	Protein Coding	44	GC0XM049028	0.237346679
TFF1	Trefoil Factor 1	Protein Coding	45	GC21M042362	0.237346679
TLE5	TLE Family Member 5, Transcriptional Modulator	Protein Coding	31	GC19M003053	0.237346679
TLN1	Talin 1	Protein Coding	42	GC09M035696	0.237346679
TMEM70	Transmembrane Protein 70	Protein Coding	38	GC08P073972	0.237346679
TNFAIP3	TNF Alpha Induced Protein 3	Protein Coding	48	GC06P137866	0.237346679

TRIP13	Thyroid Hormone Receptor Interactor 13	Protein Coding	41	GC05P000892	0.237346679
TXN	Thioredoxin	Protein Coding	46	GC09M110243	0.237346679
USP33	Ubiquitin Specific Peptidase 33	Protein Coding	39	GC01M077695	0.237346679
VSNL1	Visinin Like 1	Protein Coding	41	GC02P017539	0.237346679
WDR77	WD Repeat Domain 77	Protein Coding	40	GC01M111439	0.237346679
HNRNPH1	Heterogeneous Nuclear Ribonucleoprotein H1	Protein Coding	40	GC05M179614	0.214007348
ALPL	Alkaline Phosphatase, Biomineralization Associated	Protein Coding	51	GC01P021508	0.214007348
APOA2	Apolipoprotein A2	Protein Coding	44	GC01M161222	0.214007348
DPAGT1	Dolichyl-Phosphate N-Acetylglucosaminephosphotransferase 1	Protein Coding	45	GC11M119096	0.214007348
ELF3	E74 Like ETS Transcription Factor 3	Protein Coding	40	GC01P202007	0.214007348
ETV7	ETS Variant Transcription Factor 7	Protein Coding	37	GC06M047035	0.214007348
GUSB	Glucuronidase Beta	Protein Coding	48	GC07M065960	0.214007348
H2AC4	H2A Clustered Histone 4	Protein Coding	30	GC06M026034	0.214007348
HNRNPD	Heterogeneous Nuclear Ribonucleoprotein D	Protein Coding	43	GC04M082352	0.214007348
MAP2K6	Mitogen-Activated Protein Kinase Kinase 6	Protein Coding	48	GC17P069414	0.214007348
POLK	DNA Polymerase Kappa	Protein Coding	43	GC05P075511	0.214007348
SLC25A19	Solute Carrier Family 25 Member 19	Protein Coding	44	GC17M075273	0.214007348
SNRNP70	Small Nuclear Ribonucleoprotein U1 Subunit 70	Protein Coding	40	GC19P049085	0.214007348

SNRPA1	Small Nuclear Ribonucleoprotein Polypeptide A’	Protein Coding	39	GC15M101281	0.214007348
SRSF9	Serine And Arginine Rich Splicing Factor 9	Protein Coding	41	GC12M120461	0.214007348
TAS1R1	Taste 1 Receptor Member 1	Protein Coding	40	GC01P006555	0.214007348
TNNC2	Troponin C2, Fast Skeletal Type	Protein Coding	39	GC20M045823	0.214007348
UHMK1	U2AF Homology Motif Kinase 1	Protein Coding	40	GC01P162467	0.214007348
BMPR1A	Bone Morphogenetic Protein Receptor Type 1A	Protein Coding	51	GC10P086756	0.207090408
CDK17	Cyclin Dependent Kinase 17	Protein Coding	40	GC12M096278	0.207090408
CDK19	Cyclin Dependent Kinase 19	Protein Coding	43	GC06M110609	0.207090408
DDX46	DEAD-Box Helicase 46	Protein Coding	40	GC05P134758	0.207090408
DDX47	DEAD-Box Helicase 47	Protein Coding	38	GC12P012826	0.207090408
DDX5	DEAD-Box Helicase 5	Protein Coding	47	GC17M064498	0.207090408
LRSAM1	Leucine Rich Repeat And Sterile Alpha Motif Containing 1	Protein Coding	39	GC09P127451	0.207090408
RFK	Riboflavin Kinase	Protein Coding	40	GC09M076385	0.207090408
AAK1	AP2 Associated Kinase 1	Protein Coding	41	GC02M069459	0.207090408
AARS2	Alanyl-TRNA Synthetase 2, Mitochondrial	Protein Coding	43	GC06M044297	0.207090408
AASS	Aminoadipate-Semialdehyde Synthase	Protein Coding	45	GC07M122073	0.207090408
ABCA2	ATP Binding Cassette Subfamily A Member 2	Protein Coding	42	GC09M137007	0.207090408
ABCA3	ATP Binding Cassette Subfamily A Member 3	Protein Coding	49	GC16M002275	0.207090408

ABCB4	ATP Binding Cassette Subfamily B Member 4	Protein Coding	47	GC07M087401	0.207090408
ABCB6	ATP Binding Cassette Subfamily B Member 6 (Langereis Blood Group)	Protein Coding	47	GC02M219209	0.207090408
ABCC3	ATP Binding Cassette Subfamily C Member 3	Protein Coding	47	GC17P050634	0.207090408
ABCC8	ATP Binding Cassette Subfamily C Member 8	Protein Coding	47	GC11M017392	0.207090408
ABCC9	ATP Binding Cassette Subfamily C Member 9	Protein Coding	45	GC12M021797	0.207090408
ABCD1	ATP Binding Cassette Subfamily D Member 1	Protein Coding	47	GC0XP153724	0.207090408
ABCD2	ATP Binding Cassette Subfamily D Member 2	Protein Coding	41	GC12M039530	0.207090408
ABCD3	ATP Binding Cassette Subfamily D Member 3	Protein Coding	45	GC01P094418	0.207090408
ABCF1	ATP Binding Cassette Subfamily F Member 1	Protein Coding	40	GC06P030571	0.207090408
ABCG1	ATP Binding Cassette Subfamily G Member 1	Protein Coding	44	GC21P042199	0.207090408
ABCG5	ATP Binding Cassette Subfamily G Member 5	Protein Coding	45	GC02M043806	0.207090408
ABCG8	ATP Binding Cassette Subfamily G Member 8	Protein Coding	43	GC02P043828	0.207090408

ABL2	ABL Proto-Oncogene 2, Non-Receptor Tyrosine Kinase	Protein Coding	46	GC01M179114	0.207090408
ACTR1A	Actin Related Protein 1A	Protein Coding	41	GC10M102479	0.207090408
ACTR2	Actin Related Protein 2	Protein Coding	44	GC02P065227	0.207090408
ACVR1	Activin A Receptor Type 1	Protein Coding	51	GC02M157736	0.207090408
ACVR1B	Activin A Receptor Type 1B	Protein Coding	47	GC12P051951	0.207090408
ACVR1C	Activin A Receptor Type 1C	Protein Coding	43	GC02M157526	0.207090408
ACVR2A	Activin A Receptor Type 2A	Protein Coding	47	GC02P147844	0.207090408
ACVR2B	Activin A Receptor Type 2B	Protein Coding	51	GC03P038453	0.207090408
ACVRL1	Activin A Receptor Like Type 1	Protein Coding	50	GC12P051906	0.207090408
ADAM17	ADAM Metallopeptidase Domain 17	Protein Coding	51	GC02M009488	0.207090408
ADAMTSL1	ADAMTS Like 1	Protein Coding	42	GC09P017906	0.207090408
ADM2	Adrenomedullin 2	Protein Coding	34	GC22P050481	0.207090408
ADPGK	ADP Dependent Glucokinase	Protein Coding	43	GC15M072751	0.207090408
AFG3L2	AFG3 Like Matrix AAA Peptidase Subunit 2	Protein Coding	45	GC18M012328	0.207090408
AGK	Acylglycerol Kinase	Protein Coding	43	GC07P141551	0.207090408
AGRP	Agouti Related Neuropeptide	Protein Coding	44	GC16M067482	0.207090408
AIMP1	Aminoacyl TRNA Synthetase Complex Interacting Multifunctional Protein 1	Protein Coding	43	GC04P106315	0.207090408
AKAP2	A-Kinase Anchoring Protein 2	Protein Coding	20	GC00U936898	0.207090408
AKAP8	A-Kinase Anchoring Protein 8	Protein Coding	42	GC19M015354	0.207090408
AKAP9	A-Kinase Anchoring Protein 9	Protein Coding	44	GC07P091940	0.207090408

AKT2	AKT Serine/Threonine Kinase 2	Protein Coding	54	GC19M040230	0.207090408
AKT3	AKT Serine/Threonine Kinase 3	Protein Coding	54	GC01M243488	0.207090408
ALDH18A1	Aldehyde Dehydrogenase 18 Family Member A1	Protein Coding	46	GC10M095605	0.207090408
ALPI	Alkaline Phosphatase, Intestinal	Protein Coding	44	GC02P232456	0.207090408
AMFR	Autocrine Motility Factor Receptor	Protein Coding	44	GC16M056361	0.207090408
AMHR2	Anti-Mullerian Hormone Receptor Type 2	Protein Coding	47	GC12P053423	0.207090408
ANPEP	Alanyl Aminopeptidase, Membrane	Protein Coding	50	GC15M089784	0.207090408
ANXA6	Annexin A6	Protein Coding	43	GC05M151100	0.207090408
APOC3	Apolipoprotein C3	Protein Coding	44	GC11P116829	0.207090408
ARAF	A-Raf Proto-Oncogene, Serine/Threonine Kinase	Protein Coding	47	GC0XP047562	0.207090408
AREG	Amphiregulin	Protein Coding	43	GC04P074445	0.207090408
ARRB2	Arrestin Beta 2	Protein Coding	45	GC17P004711	0.207090408
ASCC3	Activating Signal Cointegrator 1 Complex Subunit 3	Protein Coding	38	GC06M100508	0.207090408
ASIC5	Acid Sensing Ion Channel Subunit Family Member 5	Protein Coding	34	GC04M155829	0.207090408
ASIP	Agouti Signaling Protein	Protein Coding	38	GC20P034194	0.207090408
ATAD1	ATPase Family AAA Domain Containing 1	Protein Coding	41	GC10M087751	0.207090408
ATAD2	ATPase Family AAA Domain Containing 2	Protein Coding	40	GC08M123319	0.207090408
ATF1	Activating Transcription Factor 1	Protein Coding	47	GC12P050763	0.207090408

ATF3	Activating Transcription Factor 3	Protein Coding	45	GC01P212565	0.207090408
ATF5	Activating Transcription Factor 5	Protein Coding	37	GC19P049931	0.207090408
ATF7	Activating Transcription Factor 7	Protein Coding	41	GC12M053527	0.207090408
ATP10A	ATPase Phospholipid Transporting 10A (Putative)	Protein Coding	40	GC15M025666	0.207090408
ATP10D	ATPase Phospholipid Transporting 10D (Putative)	Protein Coding	39	GC04P047490	0.207090408
ATP11A	ATPase Phospholipid Transporting 11A	Protein Coding	41	GC13P112690	0.207090408
ATP11B	ATPase Phospholipid Transporting 11B (Putative)	Protein Coding	41	GC03P182793	0.207090408
ATP11C	ATPase Phospholipid Transporting 11C	Protein Coding	38	GC0XM139726	0.207090408
ATP12A	ATPase H+/K+ Transporting Non-Gastric Alpha2 Subunit	Protein Coding	44	GC13P024680	0.207090408
ATP13A1	ATPase 13A1	Protein Coding	40	GC19M019645	0.207090408
ATP13A2	ATPase Cation Transporting 13A2	Protein Coding	43	GC01M016985	0.207090408
ATP13A3	ATPase 13A3	Protein Coding	37	GC03M194402	0.207090408
ATP13A5	ATPase 13A5	Protein Coding	34	GC03M193274	0.207090408
ATP1A2	ATPase Na+/K+ Transporting Subunit Alpha 2	Protein Coding	48	GC01P160115	0.207090408
ATP1A3	ATPase Na+/K+ Transporting Subunit Alpha 3	Protein Coding	48	GC19M041966	0.207090408
ATP1A4	ATPase Na+/K+ Transporting Subunit Alpha 4	Protein Coding	41	GC01P160151	0.207090408
ATP1B1	ATPase Na+/K+ Transporting Subunit Beta 1	Protein Coding	48	GC01P169105	0.207090408

ATP1B2	ATPase Na+/K+ Transporting Subunit Beta 2	Protein Coding	42	GC17P009113	0.207090408
ATP1B3	ATPase Na+/K+ Transporting Subunit Beta 3	Protein Coding	43	GC03P141876	0.207090408
ATP1B4	ATPase Na+/K+ Transporting Family Member Beta 4	Protein Coding	33	GC0XP120362	0.207090408
ATP2A1	ATPase Sarcoplasmic/Endoplasmic Reticulum Ca2+ Transporting 1	Protein Coding	49	GC16P032262	0.207090408
ATP2B1	ATPase Plasma Membrane Ca2+ Transporting 1	Protein Coding	45	GC12M089588	0.207090408
ATP2B2	ATPase Plasma Membrane Ca2+ Transporting 2	Protein Coding	48	GC03M010324	0.207090408
ATP2B3	ATPase Plasma Membrane Ca2+ Transporting 3	Protein Coding	48	GC0XP153517	0.207090408
ATP2B4	ATPase Plasma Membrane Ca2+ Transporting 4	Protein Coding	45	GC01P203626	0.207090408
ATP2C1	ATPase Secretory Pathway Ca2+ Transporting 1	Protein Coding	45	GC03P130850	0.207090408
ATP4A	ATPase H+/K+ Transporting Subunit Alpha	Protein Coding	42	GC19M047080	0.207090408
ATP4B	ATPase H+/K+ Transporting Subunit Beta	Protein Coding	41	GC13M113648	0.207090408
ATP5IF1	ATP Synthase Inhibitory Factor Subunit 1	Protein Coding	31	GC01P028237	0.207090408
ATP5MC1	ATP Synthase Membrane Subunit C Locus 1	Protein Coding	30	GC17P048893	0.207090408
ATP5MC3	ATP Synthase Membrane Subunit C Locus 3	Protein Coding	30	GC02M175180	0.207090408
ATP5PB	ATP Synthase Peripheral Stalk-Membrane Subunit B	Protein Coding	30	GC01P111449	0.207090408

ATP5PF	ATP Synthase Peripheral Stalk Subunit F6	Protein Coding	32	GC21M025761	0.207090408
ATP5PO	ATP Synthase Peripheral Stalk Subunit OSCP	Protein Coding	34	GC21M033904	0.207090408
ATP6AP1	ATPase H+ Transporting Accessory Protein 1	Protein Coding	43	GC0XP154428	0.207090408
ATP6V0A4	ATPase H+ Transporting V0 Subunit A4	Protein Coding	44	GC07M138707	0.207090408
ATP6V0C	ATPase H+ Transporting V0 Subunit C	Protein Coding	42	GC16P002513	0.207090408
ATP6V0D2	ATPase H+ Transporting V0 Subunit D2	Protein Coding	39	GC08P085987	0.207090408
ATP6V0E1	ATPase H+ Transporting V0 Subunit E1	Protein Coding	37	GC05P172983	0.207090408
ATP6V1A	ATPase H+ Transporting V1 Subunit A	Protein Coding	45	GC03P113747	0.207090408
ATP6V1B1	ATPase H+ Transporting V1 Subunit B1	Protein Coding	45	GC02P070935	0.207090408
ATP6V1B2	ATPase H+ Transporting V1 Subunit B2	Protein Coding	47	GC08P020197	0.207090408
ATP6V1C1	ATPase H+ Transporting V1 Subunit C1	Protein Coding	41	GC08P103038	0.207090408
ATP6V1C2	ATPase H+ Transporting V1 Subunit C2	Protein Coding	42	GC02P010713	0.207090408
ATP6V1E1	ATPase H+ Transporting V1 Subunit E1	Protein Coding	44	GC22M017592	0.207090408
ATP6V1F	ATPase H+ Transporting V1 Subunit F	Protein Coding	41	GC07P128862	0.207090408
ATP6V1G1	ATPase H+ Transporting V1 Subunit G1	Protein Coding	39	GC09P115756	0.207090408
ATP6V1G2	ATPase H+ Transporting V1 Subunit G2	Protein Coding	39	GC06M046877	0.207090408

ATP6V1G3	ATPase H+ Transporting V1 Subunit G3	Protein Coding	43	GC01M198492	0.207090408
ATP6V1H	ATPase H+ Transporting V1 Subunit H	Protein Coding	42	GC08M053715	0.207090408
ATP7A	ATPase Copper Transporting Alpha	Protein Coding	47	GC0XP077943	0.207090408
ATP7B	ATPase Copper Transporting Beta	Protein Coding	48	GC13M051930	0.207090408
ATP8B1	ATPase Phospholipid Transporting 8B1	Protein Coding	40	GC18M057646	0.207090408
ATP8B2	ATPase Phospholipid Transporting 8B2	Protein Coding	40	GC01P154325	0.207090408
ATP8B3	ATPase Phospholipid Transporting 8B3	Protein Coding	40	GC19M002182	0.207090408
ATP8B4	ATPase Phospholipid Transporting 8B4 (Putative)	Protein Coding	37	GC15M049858	0.207090408
ATP9A	ATPase Phospholipid Transporting 9A (Putative)	Protein Coding	37	GC20M051596	0.207090408
ATP9B	ATPase Phospholipid Transporting 9B (Putative)	Protein Coding	38	GC18P079069	0.207090408
ATRX	ATRX Chromatin Remodeler	Protein Coding	47	GC0XM077504	0.207090408
AVPR2	Arginine Vasopressin Receptor 2	Protein Coding	48	GC0XP153902	0.207090408
BATF	Basic Leucine Zipper ATF-Like Transcription Factor	Protein Coding	42	GC14P075523	0.207090408
BCKDK	Branched Chain Keto Acid Dehydrogenase Kinase	Protein Coding	47	GC16P032547	0.207090408
BCR	BCR Activator Of RhoGEF And GTPase	Protein Coding	52	GC22P023179	0.207090408

BHLHE40	Basic Helix-Loop-Helix Family Member E40	Protein Coding	42	GC03P004980	0.207090408
BIRC6	Baculoviral IAP Repeat Containing 6	Protein Coding	43	GC02P032357	0.207090408
BLK	BLK Proto-Oncogene, Src Family Tyrosine Kinase	Protein Coding	51	GC08P011486	0.207090408
BMP2K	BMP2 Inducible Kinase	Protein Coding	40	GC04P078776	0.207090408
BMPR1B	Bone Morphogenetic Protein Receptor Type 1B	Protein Coding	51	GC04P094757	0.207090408
BMX	BMX Non-Receptor Tyrosine Kinase	Protein Coding	45	GC0XP015392	0.207090408
BRIP1	BRCA1 Interacting Helicase 1	Protein Coding	48	GC17M061679	0.207090408
BRSK1	BR Serine/Threonine Kinase 1	Protein Coding	43	GC19P056473	0.207090408
BRSK2	BR Serine/Threonine Kinase 2	Protein Coding	43	GC11P001389	0.207090408
BST1	Bone Marrow Stromal Cell Antigen 1	Protein Coding	43	GC04P015704	0.207090408
BTAF1	B-TFIID TATA-Box Binding Protein Associated Factor 1	Protein Coding	43	GC10P091923	0.207090408
BUB1	BUB1 Mitotic Checkpoint Serine/Threonine Kinase	Protein Coding	50	GC02M110637	0.207090408
CADPS	Calcium Dependent Secretion Activator	Protein Coding	41	GC03M062398	0.207090408
CALCR	Calcitonin Receptor	Protein Coding	48	GC07M093424	0.207090408
CALCRL	Calcitonin Receptor Like Receptor	Protein Coding	45	GC02M187341	0.207090408
CALR	Calreticulin	Protein Coding	51	GC19P012938	0.207090408

CAMK1D	Calcium/Calmodulin Dependent Protein Kinase ID	Protein Coding	42	GC10P012349	0.207090408
CAMK1G	Calcium/Calmodulin Dependent Protein Kinase IG	Protein Coding	42	GC01P209583	0.207090408
CAMK2B	Calcium/Calmodulin Dependent Protein Kinase II Beta	Protein Coding	50	GC07M044217	0.207090408
CAMK2D	Calcium/Calmodulin Dependent Protein Kinase II Delta	Protein Coding	50	GC04M113452	0.207090408
CAMKK1	Calcium/Calmodulin Dependent Protein Kinase Kinase 1	Protein Coding	45	GC17M003860	0.207090408
CANX	Calnexin	Protein Coding	45	GC05P179678	0.207090408
CAP1	Cyclase Associated Actin Cytoskeleton Regulatory Protein 1	Protein Coding	41	GC01P040050	0.207090408
CAPN1	Calpain 1	Protein Coding	50	GC11P065300	0.207090408
CAPN2	Calpain 2	Protein Coding	48	GC01P223701	0.207090408
CARNS1	Carnosine Synthase 1	Protein Coding	33	GC11P067414	0.207090408
CARS2	Cysteinyl-TRNA Synthetase 2, Mitochondrial	Protein Coding	41	GC13M110641	0.207090408
CARTPT	CART Prepropeptide	Protein Coding	44	GC05P071719	0.207090408
CASK	Calcium/Calmodulin Dependent Serine Protein Kinase	Protein Coding	49	GC0XM041514	0.207090408
CASP7	Caspase 7	Protein Coding	50	GC10P113679	0.207090408
CASR	Calcium Sensing Receptor	Protein Coding	51	GC03P122183	0.207090408
CAV2	Caveolin 2	Protein Coding	43	GC07P116287	0.207090408
CBLC	Cbl Proto-Oncogene C	Protein Coding	38	GC19P044777	0.207090408
CBR3	Carbonyl Reductase 3	Protein Coding	44	GC21P036134	0.207090408
CCL22	C-C Motif Chemokine Ligand 22	Protein Coding	38	GC16P057359	0.207090408
CD63	CD63 Molecule	Protein Coding	43	GC12M055725	0.207090408

CDC34	Cell Division Cycle 34, Ubiqiutin Conjugating Enzyme	Protein Coding	48	GC19P000532	0.207090408
CDC42BPG	CDC42 Binding Protein Kinase Gamma	Protein Coding	38	GC11M064823	0.207090408
CDK10	Cyclin Dependent Kinase 10	Protein Coding	45	GC16P089680	0.207090408
CDK12	Cyclin Dependent Kinase 12	Protein Coding	41	GC17P039461	0.207090408
CDK13	Cyclin Dependent Kinase 13	Protein Coding	42	GC07P040149	0.207090408
CDK14	Cyclin Dependent Kinase 14	Protein Coding	41	GC07P090471	0.207090408
CDK15	Cyclin Dependent Kinase 15	Protein Coding	40	GC02P201790	0.207090408
CDK18	Cyclin Dependent Kinase 18	Protein Coding	40	GC01P205504	0.207090408
CDK3	Cyclin Dependent Kinase 3	Protein Coding	40	GC17P076008	0.207090408
CDK8	Cyclin Dependent Kinase 8	Protein Coding	48	GC13P026254	0.207090408
CDKL2	Cyclin Dependent Kinase Like 2	Protein Coding	38	GC04M075576	0.207090408
CDKL3	Cyclin Dependent Kinase Like 3	Protein Coding	40	GC05M134242	0.207090408
CDKL5	Cyclin Dependent Kinase Like 5	Protein Coding	44	GC0XP018425	0.207090408
CDR1	Cerebellar Degeneration Related Protein 1	Protein Coding	31	GC0XM140782	0.207090408
CDT1	Chromatin Licensing And DNA Replication Factor 1	Protein Coding	44	GC16P088803	0.207090408
CENPE	Centromere Protein E	Protein Coding	45	GC04M103105	0.207090408
CERK	Ceramide Kinase	Protein Coding	43	GC22M046684	0.207090408
CGAS	Cyclic GMP-AMP Synthase	Protein Coding	30	GC06M073414	0.207090408
CGB3	Chorionic Gonadotropin Subunit Beta 3	Protein Coding	31	GC19M049024	0.207090408
CHD1	Chromodomain Helicase DNA Binding Protein 1	Protein Coding	45	GC05M098853	0.207090408

CHD1L	Chromodomain Helicase DNA Binding Protein 1 Like	Protein Coding	42	GC01P147203	0.207090408
CHD2	Chromodomain Helicase DNA Binding Protein 2	Protein Coding	44	GC15P092900	0.207090408
CHD3	Chromodomain Helicase DNA Binding Protein 3	Protein Coding	43	GC17P009121	0.207090408
CHD5	Chromodomain Helicase DNA Binding Protein 5	Protein Coding	41	GC01M006104	0.207090408
CHD6	Chromodomain Helicase DNA Binding Protein 6	Protein Coding	38	GC20M041402	0.207090408
CHD7	Chromodomain Helicase DNA Binding Protein 7	Protein Coding	46	GC08P060678	0.207090408
CHD8	Chromodomain Helicase DNA Binding Protein 8	Protein Coding	41	GC14M021385	0.207090408
CHD9	Chromodomain Helicase DNA Binding Protein 9	Protein Coding	38	GC16P053041	0.207090408
CHFR	Checkpoint With Forkhead And Ring Finger Domains	Protein Coding	41	GC12M132822	0.207090408
CHKB	Choline Kinase Beta	Protein Coding	46	GC22M050578	0.207090408
CHPT1	Choline Phosphotransferase 1	Protein Coding	40	GC12P101696	0.207090408
CIITA	Class II Major Histocompatibility Complex Transactivator	Protein Coding	45	GC16P010879	0.207090408
CILK1	Ciliogenesis Associated Kinase 1	Protein Coding	36	GC06M053002	0.207090408
CKMT1B	Creatine Kinase, Mitochondrial 1B	Protein Coding	38	GC15P043593	0.207090408

CLCA1	Chloride Channel Accessory 1	Protein Coding	41	GC01P086468	0.207090408
CLCN2	Chloride Voltage-Gated Channel 2	Protein Coding	47	GC03M184346	0.207090408
CLK1	CDC Like Kinase 1	Protein Coding	44	GC02M200853	0.207090408
CLK2	CDC Like Kinase 2	Protein Coding	41	GC01M155262	0.207090408
CLK4	CDC Like Kinase 4	Protein Coding	41	GC05M178602	0.207090408
CLN3	CLN3 Lysosomal/Endosomal Transmembrane Protein, Battenin	Protein Coding	45	GC16M028466	0.207090408
CLPB	Caseinolytic Mitochondrial Matrix Peptidase Chaperone Subunit B	Protein Coding	44	GC11M072286	0.207090408
CLPX	Caseinolytic Mitochondrial Matrix Peptidase Chaperone Subunit X	Protein Coding	41	GC15M065148	0.207090408
CLU	Clusterin	Protein Coding	47	GC08M027596	0.207090408
CMPK2	Cytidine/Uridine Monophosphate Kinase 2	Protein Coding	38	GC02M006834	0.207090408
CNC2	Carney Complex Type 2, Multiple Neoplasia And Lentiginosis	Genetic Locus	3	GC02U990267	0.207090408
CNGB1	Cyclic Nucleotide Gated Channel Subunit Beta 1	Protein Coding	43	GC16M057884	0.207090408
CNGB3	Cyclic Nucleotide Gated Channel Subunit Beta 3	Protein Coding	41	GC08M086553	0.207090408
CNOT4	CCR4-NOT Transcription Complex Subunit 4	Protein Coding	41	GC07M135361	0.207090408
COP1	COP1 E3 Ubiquitin Ligase	Protein Coding	34	GC01M175944	0.207090408
CORT	Cortistatin	Protein Coding	36	GC01P010449	0.207090408

CPQ	Carboxypeptidase Q	Protein Coding	34	GC08P096645	0.207090408
CR1	Complement C3b/C4b Receptor 1 (Knops Blood Group)	Protein Coding	45	GC01P207496	0.207090408
CREB3	CAMP Responsive Element Binding Protein 3	Protein Coding	39	GC09P035960	0.207090408
CREB3L1	CAMP Responsive Element Binding Protein 3 Like 1	Protein Coding	42	GC11P046299	0.207090408
CREB3L2	CAMP Responsive Element Binding Protein 3 Like 2	Protein Coding	40	GC07M137874	0.207090408
CREB3L3	CAMP Responsive Element Binding Protein 3 Like 3	Protein Coding	40	GC19P004153	0.207090408
CREB5	CAMP Responsive Element Binding Protein 5	Protein Coding	41	GC07P028305	0.207090408
CREM	CAMP Responsive Element Modulator	Protein Coding	43	GC10P035126	0.207090408
CRHR1	Corticotropin Releasing Hormone Receptor 1	Protein Coding	46	GC17P045784	0.207090408
CRHR2	Corticotropin Releasing Hormone Receptor 2	Protein Coding	43	GC07M030651	0.207090408
CRTC1	CREB Regulated Transcription Coactivator 1	Protein Coding	43	GC19P026660	0.207090408
CRTC2	CREB Regulated Transcription Coactivator 2	Protein Coding	43	GC01M153947	0.207090408
CRTC3	CREB Regulated Transcription Coactivator 3	Protein Coding	40	GC15P090529	0.207090408
CRYAA	Crystallin Alpha A	Protein Coding	45	GC21P043169	0.207090408
CRYM	Crystallin Mu	Protein Coding	45	GC16M021238	0.207090408
CSK	C-Terminal Src Kinase	Protein Coding	48	GC15P074782	0.207090408
CSNK1G2	Casein Kinase 1 Gamma 2	Protein Coding	47	GC19P001941	0.207090408
CTDSPL	CTD Small Phosphatase Like	Protein Coding	40	GC03P037861	0.207090408
CTPS2	CTP Synthase 2	Protein Coding	40	GC0XM016587	0.207090408

CUL1	Cullin 1	Protein Coding	45	GC07P148697	0.207090408
CXCL1	C-X-C Motif Chemokine Ligand 1	Protein Coding	43	GC04P073869	0.207090408
CYB5A	Cytochrome B5 Type A	Protein Coding	45	GC18M074250	0.207090408
CYC1	Cytochrome C1	Protein Coding	46	GC08P144095	0.207090408
CYP11A1	Cytochrome P450 Family 11 Subfamily A Member 1	Protein Coding	50	GC15M074337	0.207090408
CYP11B1	Cytochrome P450 Family 11 Subfamily B Member 1	Protein Coding	50	GC08M142872	0.207090408
CYP11B2	Cytochrome P450 Family 11 Subfamily B Member 2	Protein Coding	48	GC08M142910	0.207090408
CYP17A1	Cytochrome P450 Family 17 Subfamily A Member 1	Protein Coding	50	GC10M102830	0.207090408
CYP27A1	Cytochrome P450 Family 27 Subfamily A Member 1	Protein Coding	48	GC02P218781	0.207090408
CYP7A1	Cytochrome P450 Family 7 Subfamily A Member 1	Protein Coding	44	GC08M058476	0.207090408
DAPK2	Death Associated Protein Kinase 2	Protein Coding	42	GC15M063907	0.207090408
DAPK3	Death Associated Protein Kinase 3	Protein Coding	44	GC19M003958	0.207090408
DCLK2	Doublecortin Like Kinase 2	Protein Coding	41	GC04P150078	0.207090408
DDR2	Discoidin Domain Receptor Tyrosine Kinase 2	Protein Coding	51	GC01P162631	0.207090408
DDX10	DEAD-Box Helicase 10	Protein Coding	38	GC11P108569	0.207090408
DDX11	DEAD/H-Box Helicase 11	Protein Coding	44	GC12P031073	0.207090408
DDX11L8	DEAD/H-Box Helicase 11 Like 8 (Pseudogene)	Pseudogene	12	GC12P000012	0.207090408
DDX12P	DEAD/H-Box Helicase 12, Pseudogene	Pseudogene	19	GC12M009417	0.207090408

DDX17	DEAD-Box Helicase 17	Protein Coding	41	GC22M038483	0.207090408
DDX18	DEAD-Box Helicase 18	Protein Coding	40	GC02P117913	0.207090408
DDX19A	DEAD-Box Helicase 19A	Protein Coding	36	GC16P070346	0.207090408
DDX19B	DEAD-Box Helicase 19B	Protein Coding	38	GC16P070289	0.207090408
DDX20	DEAD-Box Helicase 20	Protein Coding	43	GC01P111755	0.207090408
DDX21	DExD-Box Helicase 21	Protein Coding	38	GC10P068956	0.207090408
DDX23	DEAD-Box Helicase 23	Protein Coding	40	GC12M048829	0.207090408
DDX24	DEAD-Box Helicase 24	Protein Coding	39	GC14M094048	0.207090408
DDX27	DEAD-Box Helicase 27	Protein Coding	36	GC20P049219	0.207090408
DDX28	DEAD-Box Helicase 28	Protein Coding	36	GC16M068023	0.207090408
DDX31	DEAD-Box Helicase 31	Protein Coding	38	GC09M132594	0.207090408
DDX39A	DExD-Box Helicase 39A	Protein Coding	38	GC19M014408	0.207090408
DDX39B	DExD-Box Helicase 39B	Protein Coding	40	GC06M031530	0.207090408
DDX3Y	DEAD-Box Helicase 3 Y-Linked	Protein Coding	39	GC0YP012903	0.207090408
DDX4	DEAD-Box Helicase 4	Protein Coding	41	GC05P055738	0.207090408
DDX42	DEAD-Box Helicase 42	Protein Coding	38	GC17P063773	0.207090408
DDX43	DEAD-Box Helicase 43	Protein Coding	35	GC06P073394	0.207090408
DDX49	DEAD-Box Helicase 49	Protein Coding	37	GC19P018919	0.207090408
DDX50	DExD-Box Helicase 50	Protein Coding	40	GC10P068901	0.207090408
DDX51	DEAD-Box Helicase 51	Protein Coding	36	GC12M132136	0.207090408
DDX52	DExD-Box Helicase 52	Protein Coding	36	GC17M037609	0.207090408
DDX54	DEAD-Box Helicase 54	Protein Coding	38	GC12M113157	0.207090408
DDX55	DEAD-Box Helicase 55	Protein Coding	36	GC12P123602	0.207090408
DDX56	DEAD-Box Helicase 56	Protein Coding	37	GC07M044565	0.207090408
DDX58	DExD/H-Box Helicase 58	Protein Coding	47	GC09M032455	0.207090408

DDX59	DEAD-Box Helicase 59	Protein Coding	38 GC01M200594	0.207090408
DDX6	DEAD-Box Helicase 6	Protein Coding	47 GC11M118748	0.207090408
DDX60	DExD/H-Box Helicase 60	Protein Coding	35 GC04M168216	0.207090408
DDX60L	DExD/H-Box 60 Like	Protein Coding	33 GC04M168356	0.207090408
DFFA	DNA Fragmentation Factor Subunit Alpha	Protein Coding	45 GC01M010456	0.207090408
DFFB	DNA Fragmentation Factor Subunit Beta	Protein Coding	43 GC01P003797	0.207090408
DGKA	Diacylglycerol Kinase Alpha	Protein Coding	45 GC12P055927	0.207090408
DGKB	Diacylglycerol Kinase Beta	Protein Coding	46 GC07M014145	0.207090408
DGKD	Diacylglycerol Kinase Delta	Protein Coding	44 GC02P233356	0.207090408
DGKE	Diacylglycerol Kinase Epsilon	Protein Coding	49 GC17P056834	0.207090408
DGKH	Diacylglycerol Kinase Eta	Protein Coding	42 GC13P042040	0.207090408
DGKI	Diacylglycerol Kinase Iota	Protein Coding	43 GC07M137381	0.207090408
DGKQ	Diacylglycerol Kinase Theta	Protein Coding	43 GC04M000942	0.207090408
DGKZ	Diacylglycerol Kinase Zeta	Protein Coding	45 GC11P046332	0.207090408
DHX16	DEAH-Box Helicase 16	Protein Coding	41 GC06M030653	0.207090408
DHX29	DExH-Box Helicase 29	Protein Coding	37 GC05M055256	0.207090408
DHX30	DExH-Box Helicase 30	Protein Coding	41 GC03P047802	0.207090408
DHX32	DEAH-Box Helicase 32 (Putative)	Protein Coding	37 GC10M125836	0.207090408
DHX33	DEAH-Box Helicase 33	Protein Coding	35 GC17M005440	0.207090408
DHX34	DExH-Box Helicase 34	Protein Coding	38 GC19P047349	0.207090408
DHX35	DEAH-Box Helicase 35	Protein Coding	36 GC20P038963	0.207090408
DHX36	DEAH-Box Helicase 36	Protein Coding	40 GC03M154272	0.207090408
DHX37	DEAH-Box Helicase 37	Protein Coding	38 GC12M124946	0.207090408

DHX38	DEAH-Box Helicase 38	Protein Coding	44	GC16P072127	0.207090408
DHX40	DEAH-Box Helicase 40	Protein Coding	35	GC17P059565	0.207090408
DHX57	DExH-Box Helicase 57	Protein Coding	36	GC02M038797	0.207090408
DHX58	DExH-Box Helicase 58	Protein Coding	39	GC17M042101	0.207090408
DHX8	DEAH-Box Helicase 8	Protein Coding	38	GC17P043483	0.207090408
DHX9	DExH-Box Helicase 9	Protein Coding	41	GC01P182839	0.207090408
DISC1	DISC1 Scaffold Protein	Protein Coding	44	GC01P231626	0.207090408
DLK1	Delta Like Non-Canonical Notch Ligand 1	Protein Coding	45	GC14P106102	0.207090408
DMC1	DNA Meiotic Recombinase 1	Protein Coding	42	GC22M048519	0.207090408
DMTN	Dematin Actin Binding Protein	Protein Coding	37	GC08P022048	0.207090408
DNA2	DNA Replication Helicase/Nuclease 2	Protein Coding	42	GC10M068414	0.207090408
DNAJA2	DnaJ Heat Shock Protein Family (Hsp40) Member A2	Protein Coding	41	GC16M046955	0.207090408
DNAJB1	DnaJ Heat Shock Protein Family (Hsp40) Member B1	Protein Coding	44	GC19M014514	0.207090408
DNAJB1P1	DnaJ Heat Shock Protein Family (Hsp40) Member B1 Pseudogene 1	Pseudogene	8	GC02M191879	0.207090408
DNM1L	Dynamin 1 Like	Protein Coding	48	GC12P032679	0.207090408
DPH6	Diphthamine Biosynthesis 6	Protein Coding	34	GC15M035217	0.207090408
DQX1	DEAQ-Box RNA Dependent ATPase 1	Protein Coding	33	GC02M074518	0.207090408
DRD3	Dopamine Receptor D3	Protein Coding	45	GC03M114128	0.207090408
DRD5	Dopamine Receptor D5	Protein Coding	48	GC04P009783	0.207090408
DSTN	Destrin, Actin Depolymerizing Factor	Protein Coding	40	GC20P017550	0.207090408

DSTYK	Dual Serine/Threonine And Tyrosine Protein Kinase	Protein Coding	42	GC01M205111	0.207090408
DUSP1	Dual Specificity Phosphatase 1	Protein Coding	48	GC05M172768	0.207090408
DYNC1H1	Dynein Cytoplasmic 1 Heavy Chain 1	Protein Coding	45	GC14P106032	0.207090408
DYRK1B	Dual Specificity Tyrosine Phosphorylation Regulated Kinase 1B	Protein Coding	47	GC19M039825	0.207090408
DYRK2	Dual Specificity Tyrosine Phosphorylation Regulated Kinase 2	Protein Coding	44	GC12P067558	0.207090408
DYRK3	Dual Specificity Tyrosine Phosphorylation Regulated Kinase 3	Protein Coding	41	GC01P206636	0.207090408
DYRK4	Dual Specificity Tyrosine Phosphorylation Regulated Kinase 4	Protein Coding	39	GC12P011798	0.207090408
DZIP3	DAZ Interacting Zinc Finger Protein 3	Protein Coding	37	GC03P108589	0.207090408
EARS2	Glutamyl-TRNA Synthetase 2, Mitochondrial	Protein Coding	43	GC16M023527	0.207090408
EIF2AK1	Eukaryotic Translation Initiation Factor 2 Alpha Kinase 1	Protein Coding	44	GC07M006022	0.207090408
EIF2AK3	Eukaryotic Translation Initiation Factor 2 Alpha Kinase 3	Protein Coding	50	GC02M088556	0.207090408
EIF2AK4	Eukaryotic Translation Initiation Factor 2 Alpha Kinase 4	Protein Coding	45	GC15P039934	0.207090408

EIF2S2	Eukaryotic Translation Initiation Factor 2 Subunit Beta	Protein Coding	41	GC20M034088	0.207090408
EIF2S3	Eukaryotic Translation Initiation Factor 2 Subunit Gamma	Protein Coding	46	GC0XP024054	0.207090408
EIF4H	Eukaryotic Translation Initiation Factor 4H	Protein Coding	42	GC07P074174	0.207090408
ENDOG	Endonuclease G	Protein Coding	43	GC09P128818	0.207090408
ENOPH1	Enolase-Phosphatase 1	Protein Coding	40	GC04P082430	0.207090408
EPB42	Erythrocyte Membrane Protein Band 4.2	Protein Coding	39	GC15M043271	0.207090408
EPHA1	EPH Receptor A1	Protein Coding	47	GC07M143390	0.207090408
EPHA2	EPH Receptor A2	Protein Coding	53	GC01M016124	0.207090408
EPHA4	EPH Receptor A4	Protein Coding	50	GC02M221418	0.207090408
EPHA7	EPH Receptor A7	Protein Coding	49	GC06M093240	0.207090408
EPHA8	EPH Receptor A8	Protein Coding	46	GC01P022563	0.207090408
EPHB1	EPH Receptor B1	Protein Coding	48	GC03P134598	0.207090408
EPHB2	EPH Receptor B2	Protein Coding	52	GC01P022710	0.207090408
EPHB3	EPH Receptor B3	Protein Coding	45	GC03P184561	0.207090408
EPHB6	EPH Receptor B6	Protein Coding	45	GC07P145806	0.207090408
ERBB3	Erb-B2 Receptor Tyrosine Kinase 3	Protein Coding	54	GC12P056376	0.207090408
ERBB4	Erb-B2 Receptor Tyrosine Kinase 4	Protein Coding	55	GC02M211375	0.207090408
ERCC5	ERCC Excision Repair 5, Endonuclease	Protein Coding	45	GC13P102845	0.207090408
ERCC6L	ERCC Excision Repair 6 Like, Spindle Assembly Checkpoint Helicase	Protein Coding	37	GC0XM072204	0.207090408
ERN2	Endoplasmic Reticulum To Nucleus Signaling 2	Protein Coding	38	GC16M023690	0.207090408
ESRRA	Estrogen Related Receptor Alpha	Protein Coding	48	GC11P064305	0.207090408
ETNK1	Ethanolamine Kinase 1	Protein Coding	41	GC12P022625	0.207090408

ETNK2	Ethanolamine Kinase 2	Protein Coding	41	GC01M204100	0.207090408
ETV4	ETS Variant Transcription Factor 4	Protein Coding	44	GC17M043527	0.207090408
EXO1	Exonuclease 1	Protein Coding	44	GC01P241847	0.207090408
F2R	Coagulation Factor II Thrombin Receptor	Protein Coding	47	GC05P076716	0.207090408
F8	Coagulation Factor VIII	Protein Coding	47	GC0XM154835	0.207090408
FANCL	FA Complementation Group L	Protein Coding	47	GC02M058127	0.207090408
FANCM	FA Complementation Group M	Protein Coding	43	GC14P045135	0.207090408
FARP1	FERM, ARH/RhoGEF And Pleckstrin Domain Protein 1	Protein Coding	38	GC13P098142	0.207090408
FARS2	Phenylalanyl-TRNA Synthetase 2, Mitochondrial	Protein Coding	44	GC06P005261	0.207090408
FARSA	Phenylalanyl-TRNA Synthetase Subunit Alpha	Protein Coding	41	GC19M012922	0.207090408
FARSB	Phenylalanyl-TRNA Synthetase Subunit Beta	Protein Coding	45	GC02M222570	0.207090408
FBH1	F-Box DNA Helicase 1	Protein Coding	27	GC10P005889	0.207090408
FCGR2A	Fc Fragment Of IgG Receptor IIa	Protein Coding	46	GC01P161505	0.207090408
FCSK	Fucose Kinase	Protein Coding	32	GC16P070454	0.207090408
FDFT1	Farnesyl-Diphosphate Farnesyltransferase 1	Protein Coding	46	GC08P011795	0.207090408
FDX1	Ferredoxin 1	Protein Coding	41	GC11P110429	0.207090408
FEN1	Flap Structure-Specific Endonuclease 1	Protein Coding	47	GC11P061793	0.207090408
FER	FER Tyrosine Kinase	Protein Coding	47	GC05P108747	0.207090408
FES	FES Proto-Oncogene, Tyrosine Kinase	Protein Coding	48	GC15P090883	0.207090408
FGFR4	Fibroblast Growth Factor Receptor 4	Protein Coding	52	GC05P177086	0.207090408

FHL1	Four And A Half LIM Domains 1	Protein Coding	47	GC0XP136146	0.207090408
FICD	FIC Domain Protein Adenylyltransferase	Protein Coding	34	GC12P108515	0.207090408
FIGNL1	Fidgetin Like 1	Protein Coding	40	GC07M050444	0.207090408
FKBP4	FKBP Prolyl Isomerase 4	Protein Coding	45	GC12P002795	0.207090408
FLII	FLII Actin Remodeling Protein	Protein Coding	43	GC17M018244	0.207090408
FLOT1	Flotillin 1	Protein Coding	42	GC06M046803	0.207090408
FLT1	Fms Related Receptor Tyrosine Kinase 1	Protein Coding	51	GC13M028300	0.207090408
FLT4	Fms Related Receptor Tyrosine Kinase 4	Protein Coding	52	GC05M180607	0.207090408
FMN1	Formin 1	Protein Coding	39	GC15M032765	0.207090408
FN3K	Fructosamine 3 Kinase	Protein Coding	41	GC17P082735	0.207090408
FOSL1	FOS Like 1, AP-1 Transcription Factor Subunit	Protein Coding	45	GC11M069434	0.207090408
FRK	Fyn Related Src Family Tyrosine Kinase	Protein Coding	45	GC06M115931	0.207090408
FTMT	Ferritin Mitochondrial	Protein Coding	38	GC05P121851	0.207090408
FUBP1	Far Upstream Element Binding Protein 1	Protein Coding	39	GC01M077944	0.207090408
FURIN	Furin, Paired Basic Amino Acid Cleaving Enzyme	Protein Coding	47	GC15P090868	0.207090408
G3BP1	G3BP Stress Granule Assembly Factor 1	Protein Coding	41	GC05P151771	0.207090408
GABBR1	Gamma-Aminobutyric Acid Type B Receptor Subunit 1	Protein Coding	48	GC06M029555	0.207090408
GAD2	Glutamate Decarboxylase 2	Protein Coding	47	GC10P026216	0.207090408
GAK	Cyclin G Associated Kinase	Protein Coding	45	GC04M000849	0.207090408

GALK1	Galactokinase 1	Protein Coding	48	GC17M075751	0.207090408
GALK2	Galactokinase 2	Protein Coding	41	GC15P049155	0.207090408
GATB	Glutamyl-TRNA Amidotransferase Subunit B	Protein Coding	37	GC04M151670	0.207090408
GATC	Glutamyl-TRNA Amidotransferase Subunit C	Protein Coding	39	GC12P120446	0.207090408
GCA	Grancalcin	Protein Coding	41	GC02P162318	0.207090408
GCLC	Glutamate-Cysteine Ligase Catalytic Subunit	Protein Coding	44	GC06M053497	0.207090408
GHRHR	Growth Hormone Releasing Hormone Receptor	Protein Coding	44	GC07P030938	0.207090408
GHRL	Ghrelin And Obestatin Prepropeptide	Protein Coding	44	GC03M010285	0.207090408
GIP	Gastric Inhibitory Polypeptide	Protein Coding	40	GC17M048958	0.207090408
GJA5	Gap Junction Protein Alpha 5	Protein Coding	46	GC01M147756	0.207090408
GK2	Glycerol Kinase 2	Protein Coding	41	GC04M079406	0.207090408
GK3P	Glycerol Kinase 3 Pseudogene	Pseudogene	23	GC04M165277	0.207090408
GK5	Glycerol Kinase 5	Protein Coding	37	GC03M142158	0.207090408
GLP1R	Glucagon Like Peptide 1 Receptor	Protein Coding	47	GC06P039048	0.207090408
GLYCTK	Glycerate Kinase	Protein Coding	43	GC03P052288	0.207090408
GMNN	Geminin DNA Replication Inhibitor	Protein Coding	46	GC06P024779	0.207090408
GNAT3	G Protein Subunit Alpha Transducin 3	Protein Coding	37	GC07M080458	0.207090408
GNE	Glucosamine (UDP-N-Acetyl)-2-Epimerase/N-Acetylmannosamine Kinase	Protein Coding	44	GC09M036214	0.207090408
GNRHR	Gonadotropin Releasing Hormone Receptor	Protein Coding	48	GC04M067737	0.207090408
GPBAR1	G Protein-Coupled Bile Acid Receptor 1	Protein Coding	40	GC02P218259	0.207090408

GPHA2	Glycoprotein Hormone Subunit Alpha 2	Protein Coding	36	GC11M069353	0.207090408
GPHB5	Glycoprotein Hormone Subunit Beta 5	Protein Coding	31	GC14M063312	0.207090408
GPR17	G Protein-Coupled Receptor 17	Protein Coding	41	GC02P127645	0.207090408
GRK3	G Protein-Coupled Receptor Kinase 3	Protein Coding	39	GC22P026762	0.207090408
GRK5	G Protein-Coupled Receptor Kinase 5	Protein Coding	44	GC10P119207	0.207090408
GRK7	G Protein-Coupled Receptor Kinase 7	Protein Coding	40	GC03P141778	0.207090408
GRM7	Glutamate Metabotropic Receptor 7	Protein Coding	46	GC03P006770	0.207090408
GSK3A	Glycogen Synthase Kinase 3 Alpha	Protein Coding	50	GC19M046852	0.207090408
GTF2H2	General Transcription Factor IIH Subunit 2	Protein Coding	40	GC05M071032	0.207090408
GTF2H4	General Transcription Factor IIH Subunit 4	Protein Coding	41	GC06P055185	0.207090408
GUCA1A	Guanylate Cyclase Activator 1A	Protein Coding	43	GC06P055395	0.207090408
GUCA2A	Guanylate Cyclase Activator 2A	Protein Coding	38	GC01M042162	0.207090408
H1-0	H1.0 Linker Histone	Protein Coding	32	GC22P037898	0.207090408
H6PD	Hexose-6-Phosphate Dehydrogenase/Glucose 1-Dehydrogenase	Protein Coding	43	GC01P009234	0.207090408
HARS1	Histidyl-TRNA Synthetase 1	Protein Coding	37	GC05M140673	0.207090408
HARS2	Histidyl-TRNA Synthetase 2, Mitochondrial	Protein Coding	44	GC05P143875	0.207090408

HBA2	Hemoglobin Subunit Alpha 2	Protein Coding	43	GC16P005491	0.207090408
HCK	HCK Proto-Oncogene, Src Family Tyrosine Kinase	Protein Coding	50	GC20P032052	0.207090408
HCN1	Hyperpolarization Activated Cyclic Nucleotide Gated Potassium Channel 1	Protein Coding	46	GC05M045260	0.207090408
HCN2	Hyperpolarization Activated Cyclic Nucleotide Gated Potassium And Sodium Channel 2	Protein Coding	47	GC19P000589	0.207090408
HCN3	Hyperpolarization Activated Cyclic Nucleotide Gated Potassium Channel 3	Protein Coding	40	GC01P155277	0.207090408
HCN4	Hyperpolarization Activated Cyclic Nucleotide Gated Potassium Channel 4	Protein Coding	48	GC15M073319	0.207090408
HDC	Histidine Decarboxylase	Protein Coding	45	GC15M050241	0.207090408
HELB	DNA Helicase B	Protein Coding	35	GC12P066302	0.207090408
HELQ	Helicase, POLQ Like	Protein Coding	37	GC04M083407	0.207090408
HERC1	HECT And RLD Domain Containing E3 Ubiquitin Protein Ligase Family Member 1	Protein Coding	42	GC15M063608	0.207090408
HERC2	HECT And RLD Domain Containing E3 Ubiquitin Protein Ligase 2	Protein Coding	45	GC15M028111	0.207090408
HFM1	Helicase For Meiosis 1	Protein Coding	40	GC01M091260	0.207090408
HIPK1	Homeodomain Interacting Protein Kinase 1	Protein Coding	41	GC01P113929	0.207090408

HK1	Hexokinase 1	Protein Coding	51	GC10P069269	0.207090408
HK3	Hexokinase 3	Protein Coding	44	GC05M176882	0.207090408
HKDC1	Hexokinase Domain Containing 1	Protein Coding	41	GC10P069220	0.207090408
HMGB2	High Mobility Group Box 2	Protein Coding	41	GC04M173331	0.207090408
HPR	Haptoglobin-Related Protein	Protein Coding	40	GC16P072097	0.207090408
HPX	Hemopexin	Protein Coding	40	GC11M006435	0.207090408
HRH4	Histamine Receptor H4	Protein Coding	46	GC18P024460	0.207090408
HSD3BP4	Hydroxy-Delta-5-Steroid Dehydrogenase, 3 Beta, Pseudogene 4	Pseudogene	12	GC01P119563	0.207090408
HSF1	Heat Shock Transcription Factor 1	Protein Coding	45	GC08P144291	0.207090408
HSPA6	Heat Shock Protein Family A (Hsp70) Member 6	Protein Coding	45	GC01P161524	0.207090408
HSPA9	Heat Shock Protein Family A (Hsp70) Member 9	Protein Coding	48	GC05M138554	0.207090408
HSPE1	Heat Shock Protein Family E (Hsp10) Member 1	Protein Coding	41	GC02P197501	0.207090408
HSPH1	Heat Shock Protein Family H (Hsp110) Member 1	Protein Coding	44	GC13M031134	0.207090408
HTN3	Histatin 3	Protein Coding	35	GC04P070028	0.207090408
HTR1D	5-Hydroxytryptamine Receptor 1D	Protein Coding	47	GC01M023191	0.207090408
HTR4	5-Hydroxytryptamine Receptor 4	Protein Coding	45	GC05M148451	0.207090408
HUWE1	HECT, UBA And WWE Domain Containing E3 Ubiquitin Protein Ligase 1	Protein Coding	44	GC0XM053532	0.207090408
HYOU1	Hypoxia Up-Regulated 1	Protein Coding	44	GC11M119045	0.207090408
IARS1	Isoleucyl-TRNA Synthetase 1	Protein Coding	36	GC09M092211	0.207090408

IARS2	Isoleucyl-TRNA Synthetase 2, Mitochondrial	Protein Coding	42	GC01P220094	0.207090408
IBSP	Integrin Binding Sialoprotein	Protein Coding	38	GC04P087799	0.207090408
IDNK	IDNK Gluconokinase	Protein Coding	36	GC09P083623	0.207090408
IFIH1	Interferon Induced With Helicase C Domain 1	Protein Coding	48	GC02M162267	0.207090408
IGFBP4	Insulin Like Growth Factor Binding Protein 4	Protein Coding	43	GC17P040443	0.207090408
IGHMBP2	Immunoglobulin Mu DNA Binding Protein 2	Protein Coding	42	GC11P068903	0.207090408
IKBKE	Inhibitor Of Nuclear Factor Kappa B Kinase Subunit Epsilon	Protein Coding	46	GC01P206470	0.207090408
IL7	Interleukin 7	Protein Coding	43	GC08M078689	0.207090408
ILK	Integrin Linked Kinase	Protein Coding	47	GC11P006604	0.207090408
INHA	Inhibin Subunit Alpha	Protein Coding	44	GC02P219569	0.207090408
INSL3	Insulin Like 3	Protein Coding	41	GC19M017816	0.207090408
INSRR	Insulin Receptor Related Receptor	Protein Coding	43	GC01M156840	0.207090408
IP6K3	Inositol Hexakisphosphate Kinase 3	Protein Coding	40	GC06M033721	0.207090408
IPMK	Inositol Polyphosphate Multikinase	Protein Coding	40	GC10M058191	0.207090408
IPPK	Inositol-Pentakisphosphate 2-Kinase	Protein Coding	38	GC09M092613	0.207090408
IRAK1	Interleukin 1 Receptor Associated Kinase 1	Protein Coding	50	GC0XM154010	0.207090408
IRAK2	Interleukin 1 Receptor Associated Kinase 2	Protein Coding	42	GC03P011014	0.207090408
IRAK3	Interleukin 1 Receptor Associated Kinase 3	Protein Coding	48	GC12P066188	0.207090408

IRAK4	Interleukin 1 Receptor Associated Kinase 4	Protein Coding	48	GC12P043758	0.207090408
ISG15	ISG15 Ubiquitin Like Modifier	Protein Coding	47	GC01P001001	0.207090408
ITGA2	Integrin Subunit Alpha 2	Protein Coding	47	GC05P052989	0.207090408
ITGB3	Integrin Subunit Beta 3	Protein Coding	50	GC17P047254	0.207090408
ITK	IL2 Inducible T Cell Kinase	Protein Coding	52	GC05P157158	0.207090408
ITPK1	Inositol-Tetrakisphosphate 1-Kinase	Protein Coding	42	GC14M092936	0.207090408
ITPKA	Inositol-Trisphosphate 3-Kinase A	Protein Coding	43	GC15P041493	0.207090408
ITPKB	Inositol-Trisphosphate 3-Kinase B	Protein Coding	45	GC01M226631	0.207090408
ITPKC	Inositol-Trisphosphate 3-Kinase C	Protein Coding	44	GC19P041025	0.207090408
JDP2	Jun Dimerization Protein 2	Protein Coding	41	GC14P075427	0.207090408
JUND	JunD Proto-Oncogene, AP-1 Transcription Factor Subunit	Protein Coding	43	GC19M018279	0.207090408
KATNA1	Katanin Catalytic Subunit A1	Protein Coding	40	GC06M149594	0.207090408
KATNAL1	Katanin Catalytic Subunit A1 Like 1	Protein Coding	38	GC13M030202	0.207090408
KATNAL2	Katanin Catalytic Subunit A1 Like 2	Protein Coding	36	GC18P046917	0.207090408
KATNB1	Katanin Regulatory Subunit B1	Protein Coding	41	GC16P057735	0.207090408
KCNA10	Potassium Voltage-Gated Channel Subfamily A Member 10	Protein Coding	38	GC01M110517	0.207090408

KCNE3	Potassium Voltage-Gated Channel Subfamily E Regulatory Subunit 3	Protein Coding	45	GC11M074454	0.207090408
KCNH1	Potassium Voltage-Gated Channel Subfamily H Member 1	Protein Coding	47	GC01M210678	0.207090408
KCNH3	Potassium Voltage-Gated Channel Subfamily H Member 3	Protein Coding	43	GC12P049539	0.207090408
KCNH4	Potassium Voltage-Gated Channel Subfamily H Member 4	Protein Coding	38	GC17M042160	0.207090408
KCNH5	Potassium Voltage-Gated Channel Subfamily H Member 5	Protein Coding	44	GC14M062699	0.207090408
KCNH6	Potassium Voltage-Gated Channel Subfamily H Member 6	Protein Coding	42	GC17P063523	0.207090408
KCNH7	Potassium Voltage-Gated Channel Subfamily H Member 7	Protein Coding	44	GC02M162371	0.207090408
KCNH8	Potassium Voltage-Gated Channel Subfamily H Member 8	Protein Coding	41	GC03P019165	0.207090408
KCNJ1	Potassium Inwardly Rectifying Channel Subfamily J Member 1	Protein Coding	48	GC11M128741	0.207090408

KCNJ6	Potassium Inwardly Rectifying Channel Subfamily J Member 6	Protein Coding	48	GC21M037607	0.207090408
KCNJ8	Potassium Inwardly Rectifying Channel Subfamily J Member 8	Protein Coding	45	GC12M021764	0.207090408
KCNN1	Potassium Calcium-Activated Channel Subfamily N Member 1	Protein Coding	40	GC19P026648	0.207090408
KHK	Ketohexokinase	Protein Coding	45	GC02P027086	0.207090408
KIF16B	Kinesin Family Member 16B	Protein Coding	38	GC20M016272	0.207090408
KIF22	Kinesin Family Member 22	Protein Coding	44	GC16P032374	0.207090408
KIF2C	Kinesin Family Member 2C	Protein Coding	44	GC01P044739	0.207090408
KIFC1	Kinesin Family Member C1	Protein Coding	40	GC06P033391	0.207090408
KIR3DL1	Killer Cell Immunoglobulin Like Receptor, Three Ig Domains And Long Cytoplasmic Tail 1	Protein Coding	41	GC19P056447	0.207090408
KLF6	Kruppel Like Factor 6	Protein Coding	44	GC10M003779	0.207090408
KRT15	Keratin 15	Protein Coding	40	GC17M041513	0.207090408
KRT19	Keratin 19	Protein Coding	46	GC17M041523	0.207090408
LALBA	Lactalbumin Alpha	Protein Coding	40	GC12M048567	0.207090408
LARS1	Leucyl-TRNA Synthetase 1	Protein Coding	37	GC05M146114	0.207090408
LARS2	Leucyl-TRNA Synthetase 2, Mitochondrial	Protein Coding	45	GC03P045606	0.207090408
LDHC	Lactate Dehydrogenase C	Protein Coding	43	GC11P018433	0.207090408
LGALS3	Galectin 3	Protein Coding	45	GC14P055124	0.207090408
LGALS4	Galectin 4	Protein Coding	38	GC19M046731	0.207090408
LIG3	DNA Ligase 3	Protein Coding	45	GC17P034980	0.207090408
LIG4	DNA Ligase 4	Protein Coding	48	GC13M108207	0.207090408

LIPE	Lipase E, Hormone Sensitive Type	Protein Coding	48	GC19M042401	0.207090408
LMNA	Lamin A/C	Protein Coding	48	GC01P156082	0.207090408
LMTK2	Lemur Tyrosine Kinase 2	Protein Coding	40	GC07P098106	0.207090408
LNX1	Ligand Of Numb-Protein X 1	Protein Coding	44	GC04M053459	0.207090408
LONP1	Lon Peptidase 1, Mitochondrial	Protein Coding	44	GC19M005691	0.207090408
LRAT	Lecithin Retinol Acyltransferase	Protein Coding	45	GC04P154626	0.207090408
LRRK1	Leucine Rich Repeat Kinase 1	Protein Coding	40	GC15P100919	0.207090408
LTB4R	Leukotriene B4 Receptor	Protein Coding	45	GC14P024311	0.207090408
LUC7L3	LUC7 Like 3 Pre-MRNA Splicing Factor	Protein Coding	37	GC17P050719	0.207090408
LYN	LYN Proto-Oncogene, Src Family Tyrosine Kinase	Protein Coding	50	GC08P055879	0.207090408
LZTS1	Leucine Zipper Tumor Suppressor 1	Protein Coding	39	GC08M020246	0.207090408
MAK	Male Germ Cell Associated Kinase	Protein Coding	44	GC06M010762	0.207090408
MAP2K2	Mitogen-Activated Protein Kinase Kinase 2	Protein Coding	54	GC19M004090	0.207090408
MAP2K3	Mitogen-Activated Protein Kinase Kinase 3	Protein Coding	50	GC17P029210	0.207090408
MAP2K4	Mitogen-Activated Protein Kinase Kinase 4	Protein Coding	47	GC17P012020	0.207090408
MAP2K5	Mitogen-Activated Protein Kinase Kinase 5	Protein Coding	47	GC15P077101	0.207090408
MAP3K10	Mitogen-Activated Protein Kinase Kinase Kinase 10	Protein Coding	43	GC19P040191	0.207090408

MAP3K11	Mitogen-Activated Protein Kinase Kinase Kinase 11	Protein Coding	48	GC11M069402	0.207090408
MAP3K13	Mitogen-Activated Protein Kinase Kinase Kinase 13	Protein Coding	43	GC03P185282	0.207090408
MAP3K14	Mitogen-Activated Protein Kinase Kinase Kinase 14	Protein Coding	45	GC17M045263	0.207090408
MAP3K2	Mitogen-Activated Protein Kinase Kinase Kinase 2	Protein Coding	45	GC02M127298	0.207090408
MAP3K20	Mitogen-Activated Protein Kinase Kinase Kinase 20	Protein Coding	40	GC02P173076	0.207090408
MAP3K3	Mitogen-Activated Protein Kinase Kinase Kinase 3	Protein Coding	48	GC17P063622	0.207090408
MAP3K4	Mitogen-Activated Protein Kinase Kinase Kinase 4	Protein Coding	44	GC06P160991	0.207090408
MAP3K5	Mitogen-Activated Protein Kinase Kinase Kinase 5	Protein Coding	48	GC06M136557	0.207090408
MAP3K6	Mitogen-Activated Protein Kinase Kinase Kinase 6	Protein Coding	45	GC01M027354	0.207090408
MAP3K7	Mitogen-Activated Protein Kinase Kinase Kinase 7	Protein Coding	51	GC06M090513	0.207090408
MAP3K9	Mitogen-Activated Protein Kinase Kinase Kinase 9	Protein Coding	44	GC14M070722	0.207090408
MAP4K2	Mitogen-Activated Protein Kinase Kinase Kinase Kinase 2	Protein Coding	47	GC11M069340	0.207090408

MAP4K3	Mitogen-Activated Protein Kinase Kinase Kinase Kinase 3	Protein Coding	43	GC02M039249	0.207090408
MAP4K4	Mitogen-Activated Protein Kinase Kinase Kinase Kinase 4	Protein Coding	48	GC02P101773	0.207090408
MAP4K5	Mitogen-Activated Protein Kinase Kinase Kinase Kinase 5	Protein Coding	45	GC14M050418	0.207090408
MAPK11	Mitogen-Activated Protein Kinase 11	Protein Coding	48	GC22M050263	0.207090408
MAPK12	Mitogen-Activated Protein Kinase 12	Protein Coding	49	GC22M050246	0.207090408
MAPK13	Mitogen-Activated Protein Kinase 13	Protein Coding	48	GC06P055340	0.207090408
MAPK15	Mitogen-Activated Protein Kinase 15	Protein Coding	41	GC08P143716	0.207090408
MAPK4	Mitogen-Activated Protein Kinase 4	Protein Coding	45	GC18P050564	0.207090408
MAPK6	Mitogen-Activated Protein Kinase 6	Protein Coding	45	GC15P051952	0.207090408
MAPK7	Mitogen-Activated Protein Kinase 7	Protein Coding	49	GC17P019379	0.207090408
MAPK8IP1	Mitogen-Activated Protein Kinase 8 Interacting Protein 1	Protein Coding	46	GC11P046089	0.207090408
MAPKAPK2	MAPK Activated Protein Kinase 2	Protein Coding	49	GC01P206684	0.207090408
MAPKAPK3	MAPK Activated Protein Kinase 3	Protein Coding	50	GC03P050611	0.207090408
MAPKAPK5	MAPK Activated Protein Kinase 5	Protein Coding	45	GC12P111842	0.207090408

MARCHF1	Membrane Associated Ring-CH-Type Finger 1	Protein Coding	28	GC04M163525	0.207090408
MARCHF10	Membrane Associated Ring-CH-Type Finger 10	Protein Coding	26	GC17M062704	0.207090408
MARCHF2	Membrane Associated Ring-CH-Type Finger 2	Protein Coding	28	GC19P008414	0.207090408
MARCHF3	Membrane Associated Ring-CH-Type Finger 3	Protein Coding	28	GC05M126869	0.207090408
MARCHF5	Membrane Associated Ring-CH-Type Finger 5	Protein Coding	32	GC10P092292	0.207090408
MARCHF6	Membrane Associated Ring-CH-Type Finger 6	Protein Coding	30	GC05P010372	0.207090408
MARCHF7	Membrane Associated Ring-CH-Type Finger 7	Protein Coding	27	GC02P159713	0.207090408
MARCHF8	Membrane Associated Ring-CH-Type Finger 8	Protein Coding	28	GC10M045662	0.207090408
MARCHF9	Membrane Associated Ring-CH-Type Finger 9	Protein Coding	26	GC12P057754	0.207090408
MARCKS	Myristoylated Alanine Rich Protein Kinase C Substrate	Protein Coding	40	GC06P113857	0.207090408
MARK1	Microtubule Affinity Regulating Kinase 1	Protein Coding	43	GC01P220528	0.207090408
MARK2	Microtubule Affinity Regulating Kinase 2	Protein Coding	45	GC11P063838	0.207090408
MARK3	Microtubule Affinity Regulating Kinase 3	Protein Coding	49	GC14P103385	0.207090408
MARK4	Microtubule Affinity Regulating Kinase 4	Protein Coding	45	GC19P045079	0.207090408
MARS1	Methionyl-TRNA Synthetase 1	Protein Coding	36	GC12P057476	0.207090408

MARS2	Methionyl-TRNA Synthetase 2, Mitochondrial	Protein Coding	43	GC02P197705	0.207090408
MAST1	Microtubule Associated Serine/Threonine Kinase 1	Protein Coding	41	GC19P012903	0.207090408
MAST2	Microtubule Associated Serine/Threonine Kinase 2	Protein Coding	41	GC01P045786	0.207090408
MAST4	Microtubule Associated Serine/Threonine Kinase Family Member 4	Protein Coding	40	GC05P066596	0.207090408
MASTL	Microtubule Associated Serine/Threonine Kinase Like	Protein Coding	43	GC10P027154	0.207090408
MATK	Megakaryocyte-Associated Tyrosine Kinase	Protein Coding	46	GC19M003777	0.207090408
MBL2	Mannose Binding Lectin 2	Protein Coding	48	GC10M052760	0.207090408
MC1R	Melanocortin 1 Receptor	Protein Coding	48	GC16P089912	0.207090408
MC2R	Melanocortin 2 Receptor	Protein Coding	48	GC18M019097	0.207090408
MC3R	Melanocortin 3 Receptor	Protein Coding	44	GC20P056248	0.207090408
MCAM	Melanoma Cell Adhesion Molecule	Protein Coding	41	GC11M119308	0.207090408
MCCC1	Methylcrotonyl-CoA Carboxylase Subunit 1	Protein Coding	44	GC03M183015	0.207090408
MCCC2	Methylcrotonyl-CoA Carboxylase Subunit 2	Protein Coding	45	GC05P071587	0.207090408
MCM2	Minichromosome Maintenance Complex Component 2	Protein Coding	48	GC03P127598	0.207090408
MCM4	Minichromosome Maintenance Complex Component 4	Protein Coding	48	GC08P047965	0.207090408

MCM5	Minichromosome Maintenance Complex Component 5	Protein Coding	46	GC22P035400	0.207090408
MCM6	Minichromosome Maintenance Complex Component 6	Protein Coding	44	GC02M135839	0.207090408
MCM7	Minichromosome Maintenance Complex Component 7	Protein Coding	45	GC07M100092	0.207090408
MCM8	Minichromosome Maintenance 8 Homologous Recombination Repair Factor	Protein Coding	41	GC20P005988	0.207090408
MCM9	Minichromosome Maintenance 9 Homologous Recombination Repair Factor	Protein Coding	40	GC06M118813	0.207090408
ME2	Malic Enzyme 2	Protein Coding	45	GC18P050879	0.207090408
MEIS1	Meis Homeobox 1	Protein Coding	43	GC02P066433	0.207090408
MELK	Maternal Embryonic Leucine Zipper Kinase	Protein Coding	46	GC09P036572	0.207090408
MET	MET Proto-Oncogene, Receptor Tyrosine Kinase	Protein Coding	54	GC07P116672	0.207090408
MGRN1	Mahogunin Ring Finger 1	Protein Coding	40	GC16P005659	0.207090408
MIB1	MIB E3 Ubiquitin Protein Ligase 1	Protein Coding	47	GC18P021704	0.207090408
MINK1	Misshapen Like Kinase 1	Protein Coding	43	GC17P004833	0.207090408
MKKS	MKKS Centrosomal Shuttling Protein	Protein Coding	40	GC20M010424	0.207090408
MKNK2	MAPK Interacting Serine/Threonine Kinase 2	Protein Coding	44	GC19M002037	0.207090408
MLKL	Mixed Lineage Kinase Domain Like Pseudokinase	Protein Coding	42	GC16M074672	0.207090408
MMAB	Metabolism Of Cobalamin Associated B	Protein Coding	46	GC12M109553	0.207090408

MMP13	Matrix Metallopeptidase 13	Protein Coding	51	GC11M102942	0.207090408
MMP14	Matrix Metallopeptidase 14	Protein Coding	52	GC14P026302	0.207090408
MMUT	Methylmalonyl-CoA Mutase	Protein Coding	36	GC06M049430	0.207090408
MOCS3	Molybdenum Cofactor Synthesis 3	Protein Coding	40	GC20P050958	0.207090408
MOK	MOK Protein Kinase	Protein Coding	36	GC14M102224	0.207090408
MOS	MOS Proto-Oncogene, Serine/Threonine Kinase	Protein Coding	39	GC08M056112	0.207090408
MOV10	Mov10 RISC Complex RNA Helicase	Protein Coding	41	GC01P112673	0.207090408
MOV10L1	Mov10 Like RISC Complex RNA Helicase 1	Protein Coding	38	GC22P050089	0.207090408
MPP3	Membrane Palmitoylated Protein 3	Protein Coding	39	GC17M043800	0.207090408
MRGPRD	MAS Related GPR Family Member D	Protein Coding	37	GC11M068980	0.207090408
MRGPRX2	MAS Related GPR Family Member X2	Protein Coding	38	GC11M019077	0.207090408
MST1R	Macrophage Stimulating 1 Receptor	Protein Coding	49	GC03M050077	0.207090408
MT-ND5	Mitochondrially Encoded NADH:Ubiquinone Oxidoreductase Core Subunit 5	Protein Coding	33	GCMTP012339	0.207090408
MTPAP	Mitochondrial Poly(A) Polymerase	Protein Coding	38	GC10M031114	0.207090408
MUSK	Muscle Associated Receptor Tyrosine Kinase	Protein Coding	47	GC09P110668	0.207090408
MVD	Mevalonate Diphosphate Decarboxylase	Protein Coding	46	GC16M088651	0.207090408
MVK	Mevalonate Kinase	Protein Coding	50	GC12P109573	0.207090408

MYBPC1	Myosin Binding Protein C1	Protein Coding	44	GC12P101568	0.207090408
MYBPC3	Myosin Binding Protein C3	Protein Coding	47	GC11M068997	0.207090408
MYCBP2	MYC Binding Protein 2	Protein Coding	38	GC13M077044	0.207090408
MYH1	Myosin Heavy Chain 1	Protein Coding	41	GC17M010492	0.207090408
MYH10	Myosin Heavy Chain 10	Protein Coding	47	GC17M008474	0.207090408
MYLIP	Myosin Regulatory Light Chain Interacting Protein	Protein Coding	40	GC06P016129	0.207090408
MYLK2	Myosin Light Chain Kinase 2	Protein Coding	49	GC20P031819	0.207090408
MYLK3	Myosin Light Chain Kinase 3	Protein Coding	43	GC16M046762	0.207090408
MYO1A	Myosin IA	Protein Coding	40	GC12M057028	0.207090408
MYO1C	Myosin IC	Protein Coding	44	GC17M001464	0.207090408
MYO3A	Myosin IIIA	Protein Coding	42	GC10P025934	0.207090408
MYO3B	Myosin IIIB	Protein Coding	41	GC02P170178	0.207090408
MYO5A	Myosin VA	Protein Coding	45	GC15M067802	0.207090408
MYO9B	Myosin IXB	Protein Coding	44	GC19P026629	0.207090408
NADK	NAD Kinase	Protein Coding	44	GC01M001751	0.207090408
NADK2	NAD Kinase 2, Mitochondrial	Protein Coding	38	GC05M036194	0.207090408
NADSYN1	NAD Synthetase 1	Protein Coding	42	GC11P071454	0.207090408
NAE1	NEDD8 Activating Enzyme E1 Subunit 1	Protein Coding	43	GC16M066803	0.207090408
NAGA	Alpha-N-Acetylgalactosaminidase	Protein Coding	44	GC22M042058	0.207090408
NAGK	N-Acetylglucosamine Kinase	Protein Coding	44	GC02P071064	0.207090408
NAPA	NSF Attachment Protein Alpha	Protein Coding	39	GC19M047916	0.207090408
NAPG	NSF Attachment Protein Gamma	Protein Coding	38	GC18P010516	0.207090408
NARS1	Asparaginyl-TRNA Synthetase 1	Protein Coding	36	GC18M057601	0.207090408
NARS2	Asparaginyl-TRNA Synthetase 2, Mitochondrial	Protein Coding	44	GC11M078435	0.207090408
NAV2	Neuron Navigator 2	Protein Coding	38	GC11P019345	0.207090408
NAXD	NAD(P)HX Dehydratase	Protein Coding	32	GC13P110616	0.207090408

NCOA2	Nuclear Receptor Coactivator 2	Protein Coding	45	GC08M070109	0.207090408
NCOR1	Nuclear Receptor Corepressor 1	Protein Coding	44	GC17M016029	0.207090408
NCOR2	Nuclear Receptor Corepressor 2	Protein Coding	45	GC12M124324	0.207090408
NEDD4	NEDD4 E3 Ubiquitin Protein Ligase	Protein Coding	47	GC15M055826	0.207090408
NEDD8	NEDD8 Ubiquitin Like Modifier	Protein Coding	41	GC14M024216	0.207090408
NEFL	Neurofilament Light Chain	Protein Coding	47	GC08M024950	0.207090408
NEK1	NIMA Related Kinase 1	Protein Coding	44	GC04M169393	0.207090408
NEK11	NIMA Related Kinase 11	Protein Coding	40	GC03P131026	0.207090408
NEK2	NIMA Related Kinase 2	Protein Coding	51	GC01M211658	0.207090408
NEK4	NIMA Related Kinase 4	Protein Coding	41	GC03M052708	0.207090408
NEK6	NIMA Related Kinase 6	Protein Coding	40	GC09P124259	0.207090408
NEK7	NIMA Related Kinase 7	Protein Coding	43	GC01P198156	0.207090408
NEK8	NIMA Related Kinase 8	Protein Coding	42	GC17P028725	0.207090408
NEK9	NIMA Related Kinase 9	Protein Coding	46	GC14M075079	0.207090408
NKX2-1	NK2 Homeobox 1	Protein Coding	47	GC14M036516	0.207090408
NMNAT2	Nicotinamide Nucleotide Adenylyltransferase 2	Protein Coding	42	GC01M183248	0.207090408
NMNAT3	Nicotinamide Nucleotide Adenylyltransferase 3	Protein Coding	38	GC03M139560	0.207090408
NMRK1	Nicotinamide Riboside Kinase 1	Protein Coding	36	GC09M075061	0.207090408
NMRK2	Nicotinamide Riboside Kinase 2	Protein Coding	38	GC19P003933	0.207090408
NNT	Nicotinamide Nucleotide Transhydrogenase	Protein Coding	46	GC05P043668	0.207090408
NPC1	NPC Intracellular Cholesterol Transporter 1	Protein Coding	47	GC18M023506	0.207090408

NPC1L1	NPC1 Like Intracellular Cholesterol Transporter 1	Protein Coding	44	GC07M044512	0.207090408
NPFF	Neuropeptide FF-Amide Peptide Precursor	Protein Coding	37	GC12M053516	0.207090408
NPVF	Neuropeptide VF Precursor	Protein Coding	34	GC07M025224	0.207090408
NPY1R	Neuropeptide Y Receptor Y1	Protein Coding	47	GC04M163323	0.207090408
NPY4R	Neuropeptide Y Receptor Y4	Protein Coding	37	GC10M046461	0.207090408
NPY5R	Neuropeptide Y Receptor Y5	Protein Coding	43	GC04P163343	0.207090408
NR0B1	Nuclear Receptor Subfamily 0 Group B Member 1	Protein Coding	47	GC0XM030304	0.207090408
NR1H3	Nuclear Receptor Subfamily 1 Group H Member 3	Protein Coding	48	GC11P047248	0.207090408
NR1I3	Nuclear Receptor Subfamily 1 Group I Member 3	Protein Coding	45	GC01M161229	0.207090408
NRG1	Neuregulin 1	Protein Coding	47	GC08P031639	0.207090408
NRP1	Neuropilin 1	Protein Coding	47	GC10M033177	0.207090408
NSF	N-Ethylmaleimide Sensitive Factor, Vesicle Fusing ATPase	Protein Coding	45	GC17P046590	0.207090408
NTN1	Netrin 1	Protein Coding	45	GC17P009021	0.207090408
NUAK1	NUAK Family Kinase 1	Protein Coding	44	GC12M106063	0.207090408
NUDT18	Nudix Hydrolase 18	Protein Coding	33	GC08M022106	0.207090408
NXF1	Nuclear RNA Export Factor 1	Protein Coding	42	GC11M069264	0.207090408
OLR1	Oxidized Low Density Lipoprotein Receptor 1	Protein Coding	44	GC12M015685	0.207090408
OPA1	OPA1 Mitochondrial Dynamin Like GTPase	Protein Coding	45	GC03P193594	0.207090408
OPRK1	Opioid Receptor Kappa 1	Protein Coding	46	GC08M053227	0.207090408
OPRL1	Opioid Related Nociceptin Receptor 1	Protein Coding	47	GC20P064080	0.207090408

OPRM1	Opioid Receptor Mu 1	Protein Coding	49	GC06P154075	0.207090408
OR4D2	Olfactory Receptor Family 4 Subfamily D Member 2	Protein Coding	33	GC17P058169	0.207090408
OXSR1	Oxidative Stress Responsive Kinase 1	Protein Coding	42	GC03P038183	0.207090408
P4HB	Prolyl 4-Hydroxylase Subunit Beta	Protein Coding	50	GC17M081843	0.207090408
PAK2	P21 (RAC1) Activated Kinase 2	Protein Coding	47	GC03P196739	0.207090408
PANK1	Pantothenate Kinase 1	Protein Coding	42	GC10M089579	0.207090408
PANK2	Pantothenate Kinase 2	Protein Coding	44	GC20P003887	0.207090408
PANK3	Pantothenate Kinase 3	Protein Coding	40	GC05M168549	0.207090408
PANK4	Pantothenate Kinase 4 (Inactive)	Protein Coding	39	GC01M002508	0.207090408
PAPOLA	Poly(A) Polymerase Alpha	Protein Coding	42	GC14P096501	0.207090408
PAPOLG	Poly(A) Polymerase Gamma	Protein Coding	39	GC02P060756	0.207090408
PARG	Poly(ADP-Ribose) Glycohydrolase	Protein Coding	40	GC10M049818	0.207090408
PARP10	Poly(ADP-Ribose) Polymerase Family Member 10	Protein Coding	38	GC08M143977	0.207090408
PARP2	Poly(ADP-Ribose) Polymerase 2	Protein Coding	47	GC14P020343	0.207090408
PARP4	Poly(ADP-Ribose) Polymerase Family Member 4	Protein Coding	44	GC13M024420	0.207090408
PARP6	Poly(ADP-Ribose) Polymerase Family Member 6	Protein Coding	38	GC15M072241	0.207090408
PARS2	Prolyl-TRNA Synthetase 2, Mitochondrial	Protein Coding	43	GC01M054756	0.207090408
PASK	PAS Domain Containing Serine/Threonine Kinase	Protein Coding	42	GC02M241106	0.207090408
PBK	PDZ Binding Kinase	Protein Coding	44	GC08M027809	0.207090408

PC	Pyruvate Carboxylase	Protein Coding	48	GC11M066848	0.207090408
PCCB	Propionyl-CoA Carboxylase Subunit Beta	Protein Coding	48	GC03P136250	0.207090408
PCSK2	Proprotein Convertase Subtilisin/Kexin Type 2	Protein Coding	41	GC20P017226	0.207090408
PDC	Phosducin	Protein Coding	39	GC01M186412	0.207090408
PDE4DIP	Phosphodiesterase 4D Interacting Protein	Protein Coding	41	GC01P148808	0.207090408
PDGFRA	Platelet Derived Growth Factor Receptor Alpha	Protein Coding	55	GC04P054229	0.207090408
PDIA4	Protein Disulfide Isomerase Family A Member 4	Protein Coding	43	GC07M149003	0.207090408
PDK1	Pyruvate Dehydrogenase Kinase 1	Protein Coding	47	GC02P172555	0.207090408
PDK2	Pyruvate Dehydrogenase Kinase 2	Protein Coding	44	GC17P050095	0.207090408
PDK3	Pyruvate Dehydrogenase Kinase 3	Protein Coding	48	GC0XP024465	0.207090408
PDK4	Pyruvate Dehydrogenase Kinase 4	Protein Coding	47	GC07M095583	0.207090408
PDP1	Pyruvate Dehydrogenase Phosphatase Catalytic Subunit 1	Protein Coding	47	GC08P093857	0.207090408
PENK	Proenkephalin	Protein Coding	38	GC08M056436	0.207090408
PEX5	Peroxisomal Biogenesis Factor 5	Protein Coding	43	GC12P011881	0.207090408
PEX6	Peroxisomal Biogenesis Factor 6	Protein Coding	43	GC06M042963	0.207090408
PFKFB1	6-Phosphofructo-2-Kinase/Fructose-2,6-Biphosphatase 1	Protein Coding	43	GC0XM054932	0.207090408
PFKL	Phosphofructokinase, Liver Type	Protein Coding	45	GC21P044300	0.207090408

PFKP	Phosphofructokinase, Platelet	Protein Coding	46	GC10P003066	0.207090408
PFN1	Profilin 1	Protein Coding	48	GC17M004945	0.207090408
PHB	Prohibitin	Protein Coding	48	GC17M049404	0.207090408
PHKA2	Phosphorylase Kinase Regulatory Subunit Alpha 2	Protein Coding	46	GC0XM018892	0.207090408
PHKG1	Phosphorylase Kinase Catalytic Subunit Gamma 1	Protein Coding	45	GC07M056080	0.207090408
PHKG2	Phosphorylase Kinase Catalytic Subunit Gamma 2	Protein Coding	47	GC16P032474	0.207090408
PHOX2B	Paired Like Homeobox 2B	Protein Coding	44	GC04M041746	0.207090408
PI4KA	Phosphatidylinositol 4-Kinase Alpha	Protein Coding	48	GC22M020707	0.207090408
PI4KB	Phosphatidylinositol 4-Kinase Beta	Protein Coding	45	GC01M151291	0.207090408
PIF1	PIF1 5'-To-3' DNA Helicase	Protein Coding	37	GC15M064815	0.207090408
PIKFYVE	Phosphoinositide Kinase, FYVE-Type Zinc Finger Containing	Protein Coding	48	GC02P208266	0.207090408
PIM1	Pim-1 Proto-Oncogene, Serine/Threonine Kinase	Protein Coding	51	GC06P055356	0.207090408
PIM2	Pim-2 Proto-Oncogene, Serine/Threonine Kinase	Protein Coding	45	GC0XM048913	0.207090408
PIM3	Pim-3 Proto-Oncogene, Serine/Threonine Kinase	Protein Coding	40	GC22P049960	0.207090408
PINK1	PTEN Induced Kinase 1	Protein Coding	48	GC01P020634	0.207090408
PIP4K2A	Phosphatidylinositol-5-Phosphate 4-Kinase Type 2 Alpha	Protein Coding	45	GC10M022484	0.207090408
PIP4K2B	Phosphatidylinositol-5-Phosphate 4-Kinase Type 2 Beta	Protein Coding	43	GC17M038765	0.207090408

PIP4K2C	Phosphatidylinositol-5-Phosphate 4-Kinase Type 2 Gamma	Protein Coding	40	GC12P057591	0.207090408
PIP5K1C	Phosphatidylinositol-4-Phosphate 5-Kinase Type 1 Gamma	Protein Coding	49	GC19M003631	0.207090408
PJA1	Praja Ring Finger Ubiquitin Ligase 1	Protein Coding	40	GC0XM069160	0.207090408
PKIB	CAMP-Dependent Protein Kinase Inhibitor Beta	Protein Coding	40	GC06P122472	0.207090408
PKIG	CAMP-Dependent Protein Kinase Inhibitor Gamma	Protein Coding	38	GC20P044531	0.207090408
PKMYT1	Protein Kinase, Membrane Associated Tyrosine/Threonine 1	Protein Coding	44	GC16M003694	0.207090408
PKN2	Protein Kinase N2	Protein Coding	46	GC01P088684	0.207090408
PKN3	Protein Kinase N3	Protein Coding	40	GC09P128702	0.207090408
PLA2G1B	Phospholipase A2 Group IB	Protein Coding	47	GC12M120322	0.207090408
PLA2G6	Phospholipase A2 Group VI	Protein Coding	48	GC22M049636	0.207090408
PLCB4	Phospholipase C Beta 4	Protein Coding	48	GC20P009024	0.207090408
PLCD3	Phospholipase C Delta 3	Protein Coding	41	GC17M045108	0.207090408
PLCG2	Phospholipase C Gamma 2	Protein Coding	52	GC16P081773	0.207090408
PLCL1	Phospholipase C Like 1 (Inactive)	Protein Coding	39	GC02P197804	0.207090408
PLG	Plasminogen	Protein Coding	49	GC06P160702	0.207090408
PLIN1	Perilipin 1	Protein Coding	45	GC15M089664	0.207090408
PLK3	Polo Like Kinase 3	Protein Coding	44	GC01P044799	0.207090408
PLK4	Polo Like Kinase 4	Protein Coding	48	GC04P127880	0.207090408
PMVK	Phosphomevalonate Kinase	Protein Coding	45	GC01M154924	0.207090408
PNOC	Prepronociceptin	Protein Coding	38	GC08P028316	0.207090408
POLR1A	RNA Polymerase I Subunit A	Protein Coding	44	GC02M086021	0.207090408

POLR1B	RNA Polymerase I Subunit B	Protein Coding	41	GC02P116682	0.207090408
POLR1D	RNA Polymerase I And III Subunit D	Protein Coding	45	GC13P027620	0.207090408
POLR1E	RNA Polymerase I Subunit E	Protein Coding	37	GC09P037604	0.207090408
POLR1F	RNA Polymerase I Subunit F	Protein Coding	29	GC07M019695	0.207090408
POLR1H	RNA Polymerase I Subunit H	Protein Coding	32	GC06P057843	0.207090408
POLR2B	RNA Polymerase II Subunit B	Protein Coding	43	GC04P056977	0.207090408
POLR2C	RNA Polymerase II Subunit C	Protein Coding	43	GC16P057462	0.207090408
POLR2D	RNA Polymerase II Subunit D	Protein Coding	43	GC02M128042	0.207090408
POLR2E	RNA Polymerase II, I And III Subunit E	Protein Coding	44	GC19M001086	0.207090408
POLR2F	RNA Polymerase II, I And III Subunit F	Protein Coding	42	GC22P037952	0.207090408
POLR2G	RNA Polymerase II Subunit G	Protein Coding	39	GC11P062762	0.207090408
POLR2I	RNA Polymerase II Subunit I	Protein Coding	40	GC19M036113	0.207090408
POLR2J	RNA Polymerase II Subunit J	Protein Coding	43	GC07M102473	0.207090408
POLR2J2	RNA Polymerase II Subunit J2	Protein Coding	29	GC07M102636	0.207090408
POLR2K	RNA Polymerase II, I And III Subunit K	Protein Coding	38	GC08P100150	0.207090408
POLR3A	RNA Polymerase III Subunit A	Protein Coding	44	GC10M077969	0.207090408
POLR3B	RNA Polymerase III Subunit B	Protein Coding	44	GC12P106357	0.207090408
POLR3C	RNA Polymerase III Subunit C	Protein Coding	38	GC01P145824	0.207090408
POLR3D	RNA Polymerase III Subunit D	Protein Coding	37	GC08P022245	0.207090408
POLR3E	RNA Polymerase III Subunit E	Protein Coding	37	GC16P022298	0.207090408
POLR3F	RNA Polymerase III Subunit F	Protein Coding	41	GC20P018475	0.207090408
POLR3G	RNA Polymerase III Subunit G	Protein Coding	36	GC05P090471	0.207090408
POLR3GL	RNA Polymerase III Subunit GL	Protein Coding	37	GC01P145959	0.207090408

POLR3H	RNA Polymerase III Subunit H	Protein Coding	38	GC22M041525	0.207090408
POLR3K	RNA Polymerase III Subunit K	Protein Coding	40	GC16M000046	0.207090408
PON3	Paraoxonase 3	Protein Coding	45	GC07M095359	0.207090408
POU1F1	POU Class 1 Homeobox 1	Protein Coding	44	GC03M087259	0.207090408
PPCS	Phosphopantothenoylcysteine Synthetase	Protein Coding	40	GC01P042456	0.207090408
PPIP5K1	Diphosphoinositol Pentakisphosphate Kinase 1	Protein Coding	34	GC15M043533	0.207090408
PPIP5K2	Diphosphoinositol Pentakisphosphate Kinase 2	Protein Coding	40	GC05P103120	0.207090408
PPP1R1A	Protein Phosphatase 1 Regulatory Inhibitor Subunit 1A	Protein Coding	43	GC12M054576	0.207090408
PPP5C	Protein Phosphatase 5 Catalytic Subunit	Protein Coding	45	GC19P046346	0.207090408
PRDX2	Peroxiredoxin 2	Protein Coding	47	GC19M012796	0.207090408
PRG2	Proteoglycan 2, Pro Eosinophil Major Basic Protein	Protein Coding	41	GC11M057386	0.207090408
PRKAR1B	Protein Kinase CAMP-Dependent Type I Regulatory Subunit Beta	Protein Coding	48	GC07M000549	0.207090408
PRKAR2A	Protein Kinase CAMP-Dependent Type II Regulatory Subunit Alpha	Protein Coding	47	GC03M048744	0.207090408
PRKCD	Protein Kinase C Delta	Protein Coding	54	GC03P053156	0.207090408
PRKCE	Protein Kinase C Epsilon	Protein Coding	50	GC02P045651	0.207090408
PRKCG	Protein Kinase C Gamma	Protein Coding	52	GC19P053879	0.207090408
PRKCH	Protein Kinase C Eta	Protein Coding	51	GC14P061187	0.207090408

PRKCQ	Protein Kinase C Theta	Protein Coding	50	GC10M006393	0.207090408
PRKD1	Protein Kinase D1	Protein Coding	51	GC14M029576	0.207090408
PRKD2	Protein Kinase D2	Protein Coding	47	GC19M046674	0.207090408
PRKD3	Protein Kinase D3	Protein Coding	46	GC02M037251	0.207090408
PRNP	Prion Protein	Protein Coding	47	GC20P004686	0.207090408
PRPF19	Pre-MRNA Processing Factor 19	Protein Coding	38	GC11M060890	0.207090408
PRPF4	Pre-MRNA Processing Factor 4	Protein Coding	43	GC09P113275	0.207090408
PRPF4B	Pre-MRNA Processing Factor 4B	Protein Coding	41	GC06P004021	0.207090408
PSKH1	Protein Serine Kinase H1	Protein Coding	40	GC16P067927	0.207090408
PTBP1	Polypyrimidine Tract Binding Protein 1	Protein Coding	43	GC19P000797	0.207090408
PTGDR	Prostaglandin D2 Receptor	Protein Coding	48	GC14P052267	0.207090408
PTGER1	Prostaglandin E Receptor 1	Protein Coding	41	GC19M014444	0.207090408
PTGER2	Prostaglandin E Receptor 2	Protein Coding	49	GC14P052314	0.207090408
PTGER3	Prostaglandin E Receptor 3	Protein Coding	47	GC01M070852	0.207090408
PTGER4	Prostaglandin E Receptor 4	Protein Coding	47	GC05P040679	0.207090408
PTGFR	Prostaglandin F Receptor	Protein Coding	45	GC01P078303	0.207090408
PTH1R	Parathyroid Hormone 1 Receptor	Protein Coding	50	GC03P046877	0.207090408
PTK6	Protein Tyrosine Kinase 6	Protein Coding	48	GC20M063528	0.207090408
PTK7	Protein Tyrosine Kinase 7 (Inactive)	Protein Coding	44	GC06P043076	0.207090408
PTPN1	Protein Tyrosine Phosphatase Non-Receptor Type 1	Protein Coding	51	GC20P050510	0.207090408
PTPN7	Protein Tyrosine Phosphatase Non-Receptor Type 7	Protein Coding	44	GC01M202147	0.207090408

PTPRU	Protein Tyrosine Phosphatase Receptor Type U	Protein Coding	43	GC01P029236	0.207090408
PTS	6-Pyruvoyltetrahydropterin Synthase	Protein Coding	48	GC11P112226	0.207090408
PVALB	Parvalbumin	Protein Coding	40	GC22M036800	0.207090408
PYGB	Glycogen Phosphorylase B	Protein Coding	45	GC20P025248	0.207090408
QRSL1	Glutaminyl-TRNA Amidotransferase Subunit QRSL1	Protein Coding	40	GC06P106629	0.207090408
RAB6A	RAB6A, Member RAS Oncogene Family	Protein Coding	41	GC11M073676	0.207090408
RAD17	RAD17 Checkpoint Clamp Loader Component	Protein Coding	43	GC05P069369	0.207090408
RAD50	RAD50 Double Strand Break Repair Protein	Protein Coding	50	GC05P132556	0.207090408
RAD54L	RAD54 Like	Protein Coding	47	GC01P046278	0.207090408
RAD54L2	RAD54 Like 2	Protein Coding	40	GC03P051538	0.207090408
RALBP1	RalA Binding Protein 1	Protein Coding	45	GC18P009465	0.207090408
RAP1B	RAP1B, Member Of RAS Oncogene Family	Protein Coding	45	GC12P068610	0.207090408
RAP2A	RAP2A, Member Of RAS Oncogene Family	Protein Coding	40	GC13P097436	0.207090408
RAPGEF2	Rap Guanine Nucleotide Exchange Factor 2	Protein Coding	42	GC04P159106	0.207090408
RAPGEF6	Rap Guanine Nucleotide Exchange Factor 6	Protein Coding	39	GC05M131423	0.207090408
RARS2	Arginyl-TRNA Synthetase 2, Mitochondrial	Protein Coding	43	GC06M087514	0.207090408
RBL1	RB Transcriptional Corepressor Like 1	Protein Coding	43	GC20M036996	0.207090408
RBL2	RB Transcriptional Corepressor Like 2	Protein Coding	44	GC16P053433	0.207090408

RBM8A	RNA Binding Motif Protein 8A	Protein Coding	41	GC01M145921	0.207090408
RECQL	RecQ Like Helicase	Protein Coding	41	GC12M021468	0.207090408
RECQL5	RecQ Like Helicase 5	Protein Coding	39	GC17M075626	0.207090408
RFC1	Replication Factor C Subunit 1	Protein Coding	47	GC04M039291	0.207090408
RFC2	Replication Factor C Subunit 2	Protein Coding	45	GC07M074231	0.207090408
RFC4	Replication Factor C Subunit 4	Protein Coding	41	GC03M186789	0.207090408
RFWD3	Ring Finger And WD Repeat Domain 3	Protein Coding	40	GC16M074621	0.207090408
RHPN2	Rhophilin Rho GTPase Binding Protein 2	Protein Coding	40	GC19M032978	0.207090408
RIMKLA	Ribosomal Modification Protein RimK Like Family Member A	Protein Coding	35	GC01P042380	0.207090408
RIMKLB	Ribosomal Modification Protein RimK Like Family Member B	Protein Coding	34	GC12P008681	0.207090408
RIOK2	RIO Kinase 2	Protein Coding	41	GC05M097160	0.207090408
RIPK1	Receptor Interacting Serine/Threonine Kinase 1	Protein Coding	50	GC06P003073	0.207090408
RIPK3	Receptor Interacting Serine/Threonine Kinase 3	Protein Coding	43	GC14M024336	0.207090408
RIPK4	Receptor Interacting Serine/Threonine Kinase 4	Protein Coding	44	GC21M041739	0.207090408
RNF128	Ring Finger Protein 128	Protein Coding	38	GC0XP106693	0.207090408
RNF138	Ring Finger Protein 138	Protein Coding	40	GC18P032091	0.207090408
RNF144B	Ring Finger Protein 144B	Protein Coding	39	GC06P018447	0.207090408

RNF2	Ring Finger Protein 2	Protein Coding	43	GC01P185045	0.207090408
RNF20	Ring Finger Protein 20	Protein Coding	38	GC09P101533	0.207090408
RNF5	Ring Finger Protein 5	Protein Coding	43	GC06P055228	0.207090408
RNF8	Ring Finger Protein 8	Protein Coding	42	GC06P055359	0.207090408
ROR1	Receptor Tyrosine Kinase Like Orphan Receptor 1	Protein Coding	48	GC01P063774	0.207090408
ROS1	ROS Proto-Oncogene 1, Receptor Tyrosine Kinase	Protein Coding	45	GC06M117287	0.207090408
RPL19	Ribosomal Protein L19	Protein Coding	43	GC17P039200	0.207090408
RPS6KA3	Ribosomal Protein S6 Kinase A3	Protein Coding	53	GC0XM020149	0.207090408
RPS6KA4	Ribosomal Protein S6 Kinase A4	Protein Coding	48	GC11P064360	0.207090408
RPS6KA6	Ribosomal Protein S6 Kinase A6	Protein Coding	44	GC0XM084058	0.207090408
RPS6KB2	Ribosomal Protein S6 Kinase B2	Protein Coding	47	GC11P067428	0.207090408
RTCA	RNA 3'-Terminal Phosphate Cyclase	Protein Coding	37	GC01P100266	0.207090408
RTCB	RNA 2',3'-Cyclic Phosphate And 5'-OH Ligase	Protein Coding	36	GC22M032387	0.207090408
RTEL1	Regulator Of Telomere Elongation Helicase 1	Protein Coding	41	GC20P063658	0.207090408
RUVBL2	RuvB Like AAA ATPase 2	Protein Coding	45	GC19P048993	0.207090408
RXFP1	Relaxin Family Peptide Receptor 1	Protein Coding	45	GC04P158315	0.207090408
RXFP2	Relaxin Family Peptide Receptor 2	Protein Coding	43	GC13P031739	0.207090408
RXFP3	Relaxin Family Peptide Receptor 3	Protein Coding	42	GC05P033937	0.207090408

RXFP4	Relaxin Family Peptide/INSL5 Receptor 4	Protein Coding	38	GC01P155959	0.207090408
RYK	Receptor Like Tyrosine Kinase	Protein Coding	43	GC03M134065	0.207090408
S100A11	S100 Calcium Binding Protein A11	Protein Coding	43	GC01M152032	0.207090408
S1PR3	Sphingosine-1-Phosphate Receptor 3	Protein Coding	45	GC09P088991	0.207090408
SACM1L	SAC1 Like Phosphatidylinositide Phosphatase	Protein Coding	40	GC03P045692	0.207090408
SAE1	SUMO1 Activating Enzyme Subunit 1	Protein Coding	45	GC19P047115	0.207090408
SARS2	Seryl-TRNA Synthetase 2, Mitochondrial	Protein Coding	43	GC19M046734	0.207090408
SCG5	Secretogranin V	Protein Coding	38	GC15P032641	0.207090408
SCN4A	Sodium Voltage-Gated Channel Alpha Subunit 4	Protein Coding	45	GC17M063938	0.207090408
SCTR	Secretin Receptor	Protein Coding	41	GC02M119439	0.207090408
SEC14L2	SEC14 Like Lipid Binding 2	Protein Coding	40	GC22P030396	0.207090408
SEPHS1	Selenophosphate Synthetase 1	Protein Coding	43	GC10M013317	0.207090408
SEPHS2	Selenophosphate Synthetase 2	Protein Coding	38	GC16M031271	0.207090408
SERPINB2	Serpin Family B Member 2	Protein Coding	44	GC18P063871	0.207090408
SERPINH1	Serpin Family H Member 1	Protein Coding	46	GC11P075562	0.207090408
SF1	Splicing Factor 1	Protein Coding	39	GC11M064764	0.207090408
SFTPC	Surfactant Protein C	Protein Coding	44	GC08P022156	0.207090408
SFTPD	Surfactant Protein D	Protein Coding	44	GC10M079937	0.207090408
SGK2	Serum/Glucocorticoid Regulated Kinase 2	Protein Coding	41	GC20P043558	0.207090408
SHPK	Sedoheptulokinase	Protein Coding	38	GC17M003608	0.207090408
SIAH1	Siah E3 Ubiquitin Protein Ligase 1	Protein Coding	47	GC16M048357	0.207090408

SIAH2	Siah E3 Ubiquitin Protein Ligase 2	Protein Coding	45	GC03M150741	0.207090408
SIRT1	Sirtuin 1	Protein Coding	50	GC10P067884	0.207090408
SIRT2	Sirtuin 2	Protein Coding	48	GC19M038878	0.207090408
SIRT3	Sirtuin 3	Protein Coding	48	GC11M000215	0.207090408
SIRT4	Sirtuin 4	Protein Coding	40	GC12P120291	0.207090408
SIRT6	Sirtuin 6	Protein Coding	47	GC19M004174	0.207090408
SIRT7	Sirtuin 7	Protein Coding	42	GC17M081911	0.207090408
SLC12A2	Solute Carrier Family 12 Member 2	Protein Coding	48	GC05P128083	0.207090408
SLC16A1	Solute Carrier Family 16 Member 1	Protein Coding	49	GC01M112926	0.207090408
SLC17A6	Solute Carrier Family 17 Member 6	Protein Coding	41	GC11P022359	0.207090408
SLC18A3	Solute Carrier Family 18 Member A3	Protein Coding	44	GC10P049610	0.207090408
SLC1A3	Solute Carrier Family 1 Member 3	Protein Coding	51	GC05P036620	0.207090408
SLC25A17	Solute Carrier Family 25 Member 17	Protein Coding	40	GC22M048533	0.207090408
SLC25A5	Solute Carrier Family 25 Member 5	Protein Coding	45	GC0XP119468	0.207090408
SLC25A6	Solute Carrier Family 25 Member 6	Protein Coding	44	GC0XM001386	0.207090408
SLC32A1	Solute Carrier Family 32 Member 1	Protein Coding	44	GC20P038724	0.207090408
SLC4A2	Solute Carrier Family 4 Member 2	Protein Coding	44	GC07P151057	0.207090408
SLC4A7	Solute Carrier Family 4 Member 7	Protein Coding	43	GC03M027372	0.207090408
SLC8A2	Solute Carrier Family 8 Member A2	Protein Coding	41	GC19M047428	0.207090408
SLC8A3	Solute Carrier Family 8 Member A3	Protein Coding	44	GC14M070044	0.207090408
SLC9A3R1	SLC9A3 Regulator 1	Protein Coding	46	GC17P074749	0.207090408

SLC9A3R2	SLC9A3 Regulator 2	Protein Coding	40	GC16P005577	0.207090408
SLCO2A1	Solute Carrier Organic Anion Transporter Family Member 2A1	Protein Coding	43	GC03M133932	0.207090408
SLCO4C1	Solute Carrier Organic Anion Transporter Family Member 4C1	Protein Coding	37	GC05M102233	0.207090408
SLK	STE20 Like Kinase	Protein Coding	44	GC10P103967	0.207090408
SMARCAD1	SWI/SNF-Related, Matrix-Associated Actin-Dependent Regulator Of Chromatin, Subfamily A, Containing DEAD/H Box 1	Protein Coding	46	GC04P094207	0.207090408
SMC3	Structural Maintenance Of Chromosomes 3	Protein Coding	46	GC10P110567	0.207090408
SMPD1	Sphingomyelin Phosphodiesterase 1	Protein Coding	48	GC11P006390	0.207090408
SMURF2	SMAD Specific E3 Ubiquitin Protein Ligase 2	Protein Coding	43	GC17M064542	0.207090408
SNAP23	Synaptosome Associated Protein 23	Protein Coding	45	GC15P042491	0.207090408
SNAP25	Synaptosome Associated Protein 25	Protein Coding	51	GC20P010218	0.207090408
SNRK	SNF Related Kinase	Protein Coding	40	GC03P043303	0.207090408
SORD	Sorbitol Dehydrogenase	Protein Coding	47	GC15P045023	0.207090408
SOX9	SRY-Box Transcription Factor 9	Protein Coding	48	GC17P072121	0.207090408
SPAST	Spastin	Protein Coding	40	GC02P032063	0.207090408
SPHK1	Sphingosine Kinase 1	Protein Coding	48	GC17P076376	0.207090408
SPHK2	Sphingosine Kinase 2	Protein Coding	45	GC19P048619	0.207090408

SPHKAP	SPHK1 Interactor, AKAP Domain Containing	Protein Coding	32	GC02M227979	0.207090408
SPTAN1	Spectrin Alpha, Non-Erythrocytic 1	Protein Coding	48	GC09P128552	0.207090408
SREBF1	Sterol Regulatory Element Binding Transcription Factor 1	Protein Coding	46	GC17M017810	0.207090408
SRL	Sarcalumenin	Protein Coding	34	GC16M004189	0.207090408
SRMS	Src-Related Kinase Lacking C-Terminal Regulatory Tyrosine And N-Terminal Myristylation Sites	Protein Coding	39	GC20M063539	0.207090408
SRPK3	SRSF Protein Kinase 3	Protein Coding	40	GC0XP153776	0.207090408
SSB	Small RNA Binding Exonuclease Protection Factor La	Protein Coding	43	GC02P169791	0.207090408
SSBP3	Single Stranded DNA Binding Protein 3	Protein Coding	38	GC01M054225	0.207090408
SSTR1	Somatostatin Receptor 1	Protein Coding	44	GC14P038207	0.207090408
ST13	ST13 Hsp70 Interacting Protein	Protein Coding	40	GC22M048534	0.207090408
STAT6	Signal Transducer And Activator Of Transcription 6	Protein Coding	50	GC12M057095	0.207090408
STC1	Stanniocalcin 1	Protein Coding	41	GC08M023841	0.207090408
STK10	Serine/Threonine Kinase 10	Protein Coding	44	GC05M172042	0.207090408
STK17A	Serine/Threonine Kinase 17a	Protein Coding	41	GC07P043582	0.207090408
STK17B	Serine/Threonine Kinase 17b	Protein Coding	43	GC02M196133	0.207090408
STK24	Serine/Threonine Kinase 24	Protein Coding	45	GC13M098445	0.207090408
STK26	Serine/Threonine Kinase 26	Protein Coding	40	GC0XP132023	0.207090408

STK32A	Serine/Threonine Kinase 32A	Protein Coding	37	GC05P147234	0.207090408
STK32B	Serine/Threonine Kinase 32B	Protein Coding	33	GC04P005053	0.207090408
STK36	Serine/Threonine Kinase 36	Protein Coding	44	GC02P218672	0.207090408
STK38	Serine/Threonine Kinase 38	Protein Coding	44	GC06M036493	0.207090408
STK38L	Serine/Threonine Kinase 38 Like	Protein Coding	44	GC12P027243	0.207090408
STK39	Serine/Threonine Kinase 39	Protein Coding	44	GC02M167954	0.207090408
STK4	Serine/Threonine Kinase 4	Protein Coding	50	GC20P044966	0.207090408
STX1A	Syntaxin 1A	Protein Coding	48	GC07M073700	0.207090408
STX2	Syntaxin 2	Protein Coding	38	GC12M130789	0.207090408
STX5	Syntaxin 5	Protein Coding	40	GC11M062806	0.207090408
STYK1	Serine/Threonine /Tyrosine Kinase 1	Protein Coding	41	GC12M015695	0.207090408
SUPV3L1	Suv3 Like RNA Helicase	Protein Coding	38	GC10P069182	0.207090408
SYK	Spleen Associated Tyrosine Kinase	Protein Coding	51	GC09P091373	0.207090408
SYNGAP1	Synaptic Ras GTPase Activating Protein 1	Protein Coding	45	GC06P033419	0.207090408
SYT1	Synaptotagmin 1	Protein Coding	48	GC12P078863	0.207090408
TACR1	Tachykinin Receptor 1	Protein Coding	47	GC02M075010	0.207090408
TAF9	TATA-Box Binding Protein Associated Factor 9	Protein Coding	40	GC05M069364	0.207090408
TAFAZZIN	Tafazzin, Phospholipid-Lysophospholipid Transacylase	Protein Coding	34	GC0XP154413	0.207090408
TALDO1	Transaldolase 1	Protein Coding	47	GC11P000775	0.207090408
TAOK1	TAO Kinase 1	Protein Coding	45	GC17P029654	0.207090408
TAOK2	TAO Kinase 2	Protein Coding	41	GC16P032386	0.207090408
TAOK3	TAO Kinase 3	Protein Coding	41	GC12M118149	0.207090408
TAP1	Transporter 1, ATP Binding Cassette Subfamily B Member	Protein Coding	48	GC06M046946	0.207090408
TARS1	Threonyl-TRNA Synthetetase 1	Protein Coding	37	GC05P033441	0.207090408

TARS2	Threonyl-TRNA Synthetase 2, Mitochondrial	Protein Coding	43	GC01P150502	0.207090408
TARS3	Threonyl-TRNA Synthetase 3	Protein Coding	29	GC15M108668	0.207090408
TDRD9	Tudor Domain Containing 9	Protein Coding	40	GC14P106050	0.207090408
TEC	Tec Protein Tyrosine Kinase	Protein Coding	46	GC04M048137	0.207090408
TENT2	Terminal Nucleotidyltransferase 2	Protein Coding	29	GC05P079613	0.207090408
TESK1	Testis Associated Actin Remodelling Kinase 1	Protein Coding	43	GC09P035605	0.207090408
TGFB3	Transforming Growth Factor Beta 3	Protein Coding	47	GC14M075958	0.207090408
TGIF1	TGFB Induced Factor Homeobox 1	Protein Coding	47	GC18P003411	0.207090408
TGM2	Transglutaminase 2	Protein Coding	48	GC20M038127	0.207090408
THG1L	TRNA-Histidine Guanylyltransferase 1 Like	Protein Coding	41	GC05P157731	0.207090408
THPO	Thrombopoietin	Protein Coding	43	GC03M184371	0.207090408
THTPA	Thiamine Triphosphatase	Protein Coding	38	GC14P026310	0.207090408
TIAL1	TIA1 Cytotoxic Granule Associated RNA Binding Protein Like 1	Protein Coding	41	GC10M119571	0.207090408
TIMM17A	Translocase Of Inner Mitochondrial Membrane 17A	Protein Coding	41	GC01P201955	0.207090408
TIMM44	Translocase Of Inner Mitochondrial Membrane 44	Protein Coding	38	GC19M007926	0.207090408
TJP1	Tight Junction Protein 1	Protein Coding	44	GC15M029699	0.207090408
TJP2	Tight Junction Protein 2	Protein Coding	47	GC09P069121	0.207090408
TLK2	Tousled Like Kinase 2	Protein Coding	46	GC17P062458	0.207090408
TMEM132A	Transmembrane Protein 132A	Protein Coding	32	GC11P060924	0.207090408

TMSB4X	Thymosin Beta 4 X-Linked	Protein Coding	40	GC0XP012975	0.207090408
TNFRSF1B	TNF Receptor Superfamily Member 1B	Protein Coding	48	GC01P012167	0.207090408
TNIK	TRAF2 And NCK Interacting Kinase	Protein Coding	45	GC03M171061	0.207090408
TNK1	Tyrosine Kinase Non Receptor 1	Protein Coding	43	GC17P007380	0.207090408
TNK2	Tyrosine Kinase Non Receptor 2	Protein Coding	46	GC03M195863	0.207090408
TOMM20	Translocase Of Outer Mitochondrial Membrane 20	Protein Coding	40	GC01M235109	0.207090408
TOMM40	Translocase Of Outer Mitochondrial Membrane 40	Protein Coding	41	GC19P044890	0.207090408
TOP2B	DNA Topoisomerase II Beta	Protein Coding	47	GC03M025598	0.207090408
TOPORS	TOP1 Binding Arginine/Serine Rich Protein, E3 Ubiquitin Ligase	Protein Coding	43	GC09M032540	0.207090408
TP53RK	TP53 Regulating Kinase	Protein Coding	41	GC20M046684	0.207090408
TPH1	Tryptophan Hydroxylase 1	Protein Coding	45	GC11M018040	0.207090408
TPK1	Thiamin Pyrophosphokinase 1	Protein Coding	47	GC07M144451	0.207090408
TPP1	Tripeptidyl Peptidase 1	Protein Coding	43	GC11M006620	0.207090408
TPX2	TPX2 Microtubule Nucleation Factor	Protein Coding	42	GC20P031739	0.207090408
TRAP1	TNF Receptor Associated Protein 1	Protein Coding	44	GC16M003667	0.207090408
TRIM11	Tripartite Motif Containing 11	Protein Coding	40	GC01M228393	0.207090408
TRIM23	Tripartite Motif Containing 23	Protein Coding	38	GC05M065589	0.207090408
TRIM25	Tripartite Motif Containing 25	Protein Coding	44	GC17M056836	0.207090408
TRIM32	Tripartite Motif Containing 32	Protein Coding	44	GC09P116687	0.207090408

TRIM33	Tripartite Motif Containing 33	Protein Coding	44	GC01M114392	0.207090408
TRIM5	Tripartite Motif Containing 5	Protein Coding	42	GC11M005753	0.207090408
TRIM63	Tripartite Motif Containing 63	Protein Coding	41	GC01M026062	0.207090408
TRIP12	Thyroid Hormone Receptor Interactor 12	Protein Coding	43	GC02M229763	0.207090408
TRPC3	Transient Receptor Potential Cation Channel Subfamily C Member 3	Protein Coding	48	GC04M121879	0.207090408
TRPC4	Transient Receptor Potential Cation Channel Subfamily C Member 4	Protein Coding	45	GC13M037636	0.207090408
TRPM6	Transient Receptor Potential Cation Channel Subfamily M Member 6	Protein Coding	47	GC09M074725	0.207090408
TRPT1	TRNA Phosphotransferase 1	Protein Coding	34	GC11M064223	0.207090408
TRPV2	Transient Receptor Potential Cation Channel Subfamily V Member 2	Protein Coding	43	GC17P016415	0.207090408
TSSK2	Testis Specific Serine Kinase 2	Protein Coding	36	GC22P020052	0.207090408
TSSK3	Testis Specific Serine Kinase 3	Protein Coding	33	GC01P032351	0.207090408
TSSK4	Testis Specific Serine Kinase 4	Protein Coding	37	GC14P024205	0.207090408
TTBK1	Tau Tubulin Kinase 1	Protein Coding	36	GC06P043243	0.207090408
TTBK2	Tau Tubulin Kinase 2	Protein Coding	44	GC15M042738	0.207090408
TTL	Tubulin Tyrosine Ligase	Protein Coding	40	GC02P116681	0.207090408
TTPA	Alpha Tocopherol Transfer Protein	Protein Coding	41	GC08M063048	0.207090408
TUBA1B	Tubulin Alpha 1b	Protein Coding	43	GC12M049127	0.207090408

TUBG1	Tubulin Gamma 1	Protein Coding	48	GC17P042609	0.207090408
TUT1	Terminal Uridylyl Transferase 1, U6 SnRNA-Specific	Protein Coding	36	GC11M069245	0.207090408
TWNK	Twinkle MtDNA Helicase	Protein Coding	34	GC10P100993	0.207090408
TXK	TXK Tyrosine Kinase	Protein Coding	46	GC04M048069	0.207090408
TXN2	Thioredoxin 2	Protein Coding	46	GC22M036467	0.207090408
TYK2	Tyrosine Kinase 2	Protein Coding	53	GC19M010350	0.207090408
TYRO3	TYRO3 Protein Tyrosine Kinase	Protein Coding	48	GC15P041557	0.207090408
UBA2	Ubiquitin Like Modifier Activating Enzyme 2	Protein Coding	45	GC19P034428	0.207090408
UBA6	Ubiquitin Like Modifier Activating Enzyme 6	Protein Coding	41	GC04M067612	0.207090408
UBA7	Ubiquitin Like Modifier Activating Enzyme 7	Protein Coding	43	GC03M049805	0.207090408
UBE2A	Ubiquitin Conjugating Enzyme E2 A	Protein Coding	45	GC0XP119574	0.207090408
UBE2B	Ubiquitin Conjugating Enzyme E2 B	Protein Coding	46	GC05P134371	0.207090408
UBE2D2	Ubiquitin Conjugating Enzyme E2 D2	Protein Coding	45	GC05P139526	0.207090408
UBE2D3	Ubiquitin Conjugating Enzyme E2 D3	Protein Coding	47	GC04M102794	0.207090408
UBE2D4	Ubiquitin Conjugating Enzyme E2 D4 (Putative)	Protein Coding	41	GC07P043926	0.207090408
UBE2E1	Ubiquitin Conjugating Enzyme E2 E1	Protein Coding	41	GC03P023805	0.207090408
UBE2E2	Ubiquitin Conjugating Enzyme E2 E2	Protein Coding	41	GC03P023221	0.207090408

UBE2E3	Ubiquitin Conjugating Enzyme E2 E3	Protein Coding	43	GC02P180967	0.207090408
UBE2F	Ubiquitin Conjugating Enzyme E2 F (Putative)	Protein Coding	43	GC02P237988	0.207090408
UBE2G1	Ubiquitin Conjugating Enzyme E2 G1	Protein Coding	44	GC17M004270	0.207090408
UBE2G2	Ubiquitin Conjugating Enzyme E2 G2	Protein Coding	45	GC21M044768	0.207090408
UBE2H	Ubiquitin Conjugating Enzyme E2 H	Protein Coding	44	GC07M129830	0.207090408
UBE2I	Ubiquitin Conjugating Enzyme E2 I	Protein Coding	48	GC16P005523	0.207090408
UBE2J1	Ubiquitin Conjugating Enzyme E2 J1	Protein Coding	41	GC06M089326	0.207090408
UBE2J2	Ubiquitin Conjugating Enzyme E2 J2	Protein Coding	42	GC01M002692	0.207090408
UBE2K	Ubiquitin Conjugating Enzyme E2 K	Protein Coding	41	GC04P039700	0.207090408
UBE2L3	Ubiquitin Conjugating Enzyme E2 L3	Protein Coding	46	GC22P021549	0.207090408
UBE2L6	Ubiquitin Conjugating Enzyme E2 L6	Protein Coding	41	GC11M069079	0.207090408
UBE2M	Ubiquitin Conjugating Enzyme E2 M	Protein Coding	42	GC19M058555	0.207090408
UBE2N	Ubiquitin Conjugating Enzyme E2 N	Protein Coding	47	GC12M093406	0.207090408
UBE2O	Ubiquitin Conjugating Enzyme E2 O	Protein Coding	41	GC17M076389	0.207090408
UBE2Q1	Ubiquitin Conjugating Enzyme E2 Q1	Protein Coding	39	GC01M154521	0.207090408
UBE2Q2	Ubiquitin Conjugating Enzyme E2 Q2	Protein Coding	43	GC15P075843	0.207090408

UBE2R2	Ubiquitin Conjugating Enzyme E2 R2	Protein Coding	44	GC09P033817	0.207090408
UBE2T	Ubiquitin Conjugating Enzyme E2 T	Protein Coding	44	GC01M202332	0.207090408
UBE2U	Ubiquitin Conjugating Enzyme E2 U	Protein Coding	36	GC01P064231	0.207090408
UBE2W	Ubiquitin Conjugating Enzyme E2 W	Protein Coding	39	GC08M073780	0.207090408
UBE2Z	Ubiquitin Conjugating Enzyme E2 Z	Protein Coding	42	GC17P048908	0.207090408
UBE3A	Ubiquitin Protein Ligase E3A	Protein Coding	48	GC15M025333	0.207090408
UBE3B	Ubiquitin Protein Ligase E3B	Protein Coding	44	GC12P109477	0.207090408
UBE3C	Ubiquitin Protein Ligase E3C	Protein Coding	41	GC07P157138	0.207090408
UBE4A	Ubiquitination Factor E4A	Protein Coding	40	GC11P118359	0.207090408
UBE4B	Ubiquitination Factor E4B	Protein Coding	41	GC01P010032	0.207090408
UBR1	Ubiquitin Protein Ligase E3 Component N-Recognin 1	Protein Coding	44	GC15M042942	0.207090408
UBR2	Ubiquitin Protein Ligase E3 Component N-Recognin 2	Protein Coding	40	GC06P055398	0.207090408
UBR4	Ubiquitin Protein Ligase E3 Component N-Recognin 4	Protein Coding	39	GC01M019074	0.207090408
UBR5	Ubiquitin Protein Ligase E3 Component N-Recognin 5	Protein Coding	43	GC08M102252	0.207090408
UCKL1	Uridine-Cytidine Kinase 1 Like 1	Protein Coding	38	GC20M063939	0.207090408
UCN	Urocortin	Protein Coding	38	GC02M027308	0.207090408
UHRF1	Ubiquitin Like With PHD And Ring Finger Domains 1	Protein Coding	40	GC19P004910	0.207090408

ULK1	Unc-51 Like Autophagy Activating Kinase 1	Protein Coding	45	GC12P131894	0.207090408
ULK2	Unc-51 Like Autophagy Activating Kinase 2	Protein Coding	41	GC17M021706	0.207090408
ULK3	Unc-51 Like Kinase 3	Protein Coding	40	GC15M074836	0.207090408
USO1	USO1 Vesicle Transport Factor	Protein Coding	39	GC04P075724	0.207090408
VAMP3	Vesicle Associated Membrane Protein 3	Protein Coding	43	GC01P007765	0.207090408
VARS1	Valyl-TRNA Synthetase 1	Protein Coding	37	GC06M046896	0.207090408
VARS2	Valyl-TRNA Synthetase 2, Mitochondrial	Protein Coding	43	GC06P055186	0.207090408
VCP	Valosin Containing Protein	Protein Coding	50	GC09M035056	0.207090408
VDAC2	Voltage Dependent Anion Channel 2	Protein Coding	44	GC10P075210	0.207090408
VIPR1	Vasoactive Intestinal Peptide Receptor 1	Protein Coding	47	GC03P042490	0.207090408
VPS4A	Vacuolar Protein Sorting 4 Homolog A	Protein Coding	43	GC16P069311	0.207090408
VPS4B	Vacuolar Protein Sorting 4 Homolog B	Protein Coding	43	GC18M063389	0.207090408
VRK1	VRK Serine/Threonine Kinase 1	Protein Coding	49	GC14P096797	0.207090408
VRK2	VRK Serine/Threonine Kinase 2	Protein Coding	43	GC02P057907	0.207090408
VRK3	VRK Serine/Threonine Kinase 3	Protein Coding	40	GC19M049976	0.207090408
VTI1B	Vesicle Transport Through Interaction With T-SNAREs 1B	Protein Coding	43	GC14M067647	0.207090408

VTN	Vitronectin	Protein Coding	44	GC17M031079	0.207090408
WARS2	Tryptophanyl TRNA Synthetase 2, Mitochondrial	Protein Coding	45	GC01M119031	0.207090408
WAS	WASP Actin Nucleation Promoting Factor	Protein Coding	50	GC0XP048676	0.207090408
WNK1	WNK Lysine Deficient Protein Kinase 1	Protein Coding	47	GC12P000733	0.207090408
WNK2	WNK Lysine Deficient Protein Kinase 2	Protein Coding	40	GC09P093184	0.207090408
WNK3	WNK Lysine Deficient Protein Kinase 3	Protein Coding	40	GC0XM054194	0.207090408
WNK4	WNK Lysine Deficient Protein Kinase 4	Protein Coding	44	GC17P044667	0.207090408
WWP2	WW Domain Containing E3 Ubiquitin Protein Ligase 2	Protein Coding	45	GC16P069796	0.207090408
XBP1	X-Box Binding Protein 1	Protein Coding	46	GC22M028794	0.207090408
XYLB	Xylulokinase	Protein Coding	40	GC03P038363	0.207090408
YARS1	Tyrosyl-TRNA Synthetase 1	Protein Coding	37	GC01M032776	0.207090408
YARS2	Tyrosyl-TRNA Synthetase 2	Protein Coding	45	GC12M032725	0.207090408
YES1	YES Proto-Oncogene 1, Src Family Tyrosine Kinase	Protein Coding	49	GC18M000721	0.207090408
YME1L1	YME1 Like 1 ATPase	Protein Coding	43	GC10M027110	0.207090408
YTHDC2	YTH Domain Containing 2	Protein Coding	36	GC05P113513	0.207090408
ZNF202	Zinc Finger Protein 202	Protein Coding	40	GC11M123724	0.207090408
ZNRF1	Zinc And Ring Finger 1	Protein Coding	39	GC16P075033	0.207090408
ZNRF2	Zinc And Ring Finger 2	Protein Coding	34	GC07P030284	0.207090408
AQP3	Aquaporin 3 (Gill Blood Group)	Protein Coding	48	GC09M033431	0.1853358
ASL	Argininosuccinate Lyase	Protein Coding	47	GC07P066075	0.1853358

CEBPE	CCAAT Enhancer Binding Protein Epsilon	Protein Coding	43	GC14M023117	0.1853358
CORO1A	Coronin 1A	Protein Coding	44	GC16P032415	0.1853358
DNASE2B	Deoxyribonuclease 2 Beta	Protein Coding	34	GC01P084398	0.1853358
EIF1	Eukaryotic Translation Initiation Factor 1	Protein Coding	38	GC17P041688	0.1853358
ERI1	Exoribonuclease 1	Protein Coding	38	GC08P008892	0.1853358
GLUD2	Glutamate Dehydrogenase 2	Protein Coding	43	GC0XP121047	0.1853358
H1-1	H1.1 Linker Histone, Cluster Member	Protein Coding	31	GC06M026018	0.1853358
H1-2	H1.2 Linker Histone, Cluster Member	Protein Coding	33	GC06M026056	0.1853358
H1-3	H1.3 Linker Histone, Cluster Member	Protein Coding	30	GC06M047205	0.1853358
H2AC1	H2A Clustered Histone 1	Protein Coding	30	GC06M025873	0.1853358
H2AC11	H2A Clustered Histone 11	Protein Coding	31	GC06P055577	0.1853358
H2AC12	H2A Clustered Histone 12	Protein Coding	28	GC06P055578	0.1853358
H2AC13	H2A Clustered Histone 13	Protein Coding	30	GC06P055045	0.1853358
H2AC14	H2A Clustered Histone 14	Protein Coding	27	GC06M046627	0.1853358
H2AC15	H2A Clustered Histone 15	Protein Coding	29	GC06M046630	0.1853358
H2AC16	H2A Clustered Histone 16	Protein Coding	29	GC06P055054	0.1853358
H2AC17	H2A Clustered Histone 17	Protein Coding	29	GC06M047209	0.1853358
H2AC20	H2A Clustered Histone 20	Protein Coding	31	GC01P149978	0.1853358
H2AC21	H2A Clustered Histone 21	Protein Coding	27	GC01M150139	0.1853358
H2AC6	H2A Clustered Histone 6	Protein Coding	27	GC06P055579	0.1853358
H2AC7	H2A Clustered Histone 7	Protein Coding	29	GC06M047210	0.1853358
H2AC8	H2A Clustered Histone 8	Protein Coding	27	GC06P055580	0.1853358
H2AW	H2A.W Histone	Protein Coding	29	GC01M228478	0.1853358

H2BC1	H2B Clustered Histone 1	Protein Coding	26	GC06P025897	0.1853358
H2BC10	H2B Clustered Histone 10	Protein Coding	27	GC06P055582	0.1853358
H2BC11	H2B Clustered Histone 11	Protein Coding	29	GC06M046548	0.1853358
H2BC12	H2B Clustered Histone 12	Protein Coding	27	GC06M046553	0.1853358
H2BC13	H2B Clustered Histone 13	Protein Coding	27	GC06M046624	0.1853358
H2BC14	H2B Clustered Histone 14	Protein Coding	27	GC06P055046	0.1853358
H2BC15	H2B Clustered Histone 15	Protein Coding	28	GC06P055050	0.1853358
H2BC17	H2B Clustered Histone 17	Protein Coding	26	GC06P055057	0.1853358
H2BC19P	H2B Clustered Histone 19, Pseudogene	Pseudogene	11	GC01P149964	0.1853358
H2BC20P	H2B Clustered Histone 20, Pseudogene	Pseudogene	12	GC01M150125	0.1853358
H2BC3	H2B Clustered Histone 3	Protein Coding	29	GC06M026044	0.1853358
H2BC4	H2B Clustered Histone 4	Protein Coding	30	GC06M047212	0.1853358
H2BC5	H2B Clustered Histone 5	Protein Coding	29	GC06P055583	0.1853358
H2BC6	H2B Clustered Histone 6	Protein Coding	27	GC06P055584	0.1853358
H2BC7	H2B Clustered Histone 7	Protein Coding	26	GC06P055585	0.1853358
H2BC8	H2B Clustered Histone 8	Protein Coding	27	GC06M047213	0.1853358
H2BC9	H2B Clustered Histone 9	Protein Coding	30	GC06P055586	0.1853358
H2BU1	H2B.U Histone 1	Protein Coding	25	GC01P228467	0.1853358
H3-4	H3.4 Histone	Protein Coding	33	GC01M228427	0.1853358
H3C1	H3 Clustered Histone 1	Protein Coding	33	GC06P054980	0.1853358
H3C10	H3 Clustered Histone 10	Protein Coding	29	GC06P055047	0.1853358
H3C11	H3 Clustered Histone 11	Protein Coding	28	GC06M047214	0.1853358
H3C12	H3 Clustered Histone 12	Protein Coding	29	GC06M047215	0.1853358
H3C15	H3 Clustered Histone 15	Protein Coding	27	GC01P149970	0.1853358
H3C2	H3 Clustered Histone 2	Protein Coding	31	GC06M026032	0.1853358

H3C3	H3 Clustered Histone 3	Protein Coding	31	GC06P054978	0.1853358
H3C4	H3 Clustered Histone 4	Protein Coding	31	GC06M047216	0.1853358
H3C6	H3 Clustered Histone 6	Protein Coding	28	GC06P055588	0.1853358
H3C7	H3 Clustered Histone 7	Protein Coding	28	GC06M047217	0.1853358
H3C8	H3 Clustered Histone 8	Protein Coding	29	GC06M047218	0.1853358
HBD	Hemoglobin Subunit Delta	Protein Coding	39	GC11M005232	0.1853358
HBE1	Hemoglobin Subunit Epsilon 1	Protein Coding	40	GC11M005268	0.1853358
LOC106867047	Origin Of Replication Upstream Of MYC	Biological Region	1	GC08P127733	0.1853358
MPV17	Mitochondrial Inner Membrane Protein MPV17	Protein Coding	42	GC02M027309	0.1853358
NEXMIF	Neurite Extension And Migration Factor	Protein Coding	27	GC0XM074733	0.1853358
NRGN	Neurogranin	Protein Coding	39	GC11P124739	0.1853358
PAIP1	Poly(A) Binding Protein Interacting Protein 1	Protein Coding	38	GC05M043526	0.1853358
PITPNC1	Phosphatidylinositol Transfer Protein Cytoplasmic 1	Protein Coding	36	GC17P067377	0.1853358
PITPNM1	Phosphatidylinositol Transfer Protein Membrane Associated 1	Protein Coding	39	GC11M069524	0.1853358
POLN	DNA Polymerase Nu	Protein Coding	34	GC04M002073	0.1853358
RAD52	RAD52 Homolog, DNA Repair Protein	Protein Coding	42	GC12M000912	0.1853358
RFX1	Regulatory Factor X1	Protein Coding	38	GC19M013961	0.1853358
SLC11A1	Solute Carrier Family 11 Member 1	Protein Coding	48	GC02P218382	0.1853358
TLR7	Toll Like Receptor 7	Protein Coding	48	GC0XP012867	0.1853358
UOX	Urate Oxidase (Pseudogene)	Pseudogene	16	GC01M084364	0.1853358

ACTB	Actin Beta	Protein Coding	50	GC07M005527	0.151326045
AGL	Amylo-Alpha-1, 6-Glucosidase, 4-Alpha-Glucanotransferase	Protein Coding	45	GC01P099850	0.151326045
AKR1C1	Aldo-Keto Reductase Family 1 Member C1	Protein Coding	44	GC10P004963	0.151326045
AKR1C2	Aldo-Keto Reductase Family 1 Member C2	Protein Coding	47	GC10M004987	0.151326045
APOC4	Apolipoprotein C4	Protein Coding	36	GC19P044943	0.151326045
APOD	Apolipoprotein D	Protein Coding	43	GC03M195568	0.151326045
ARHGAP26	Rho GTPase Activating Protein 26	Protein Coding	45	GC05P142770	0.151326045
ARID1B	AT-Rich Interaction Domain 1B	Protein Coding	45	GC06P156777	0.151326045
CALM1	Calmodulin 1	Protein Coding	45	GC14P090396	0.151326045
CD36	CD36 Molecule	Protein Coding	49	GC07P080369	0.151326045
CEACAM5	CEA Cell Adhesion Molecule 5	Protein Coding	42	GC19P041709	0.151326045
CENPB	Centromere Protein B	Protein Coding	38	GC20M003783	0.151326045
CLASP1	Cytoplasmic Linker Associated Protein 1	Protein Coding	41	GC02M121337	0.151326045
COL2A1	Collagen Type II Alpha 1 Chain	Protein Coding	49	GC12M047972	0.151326045
CSF2RA	Colony Stimulating Factor 2 Receptor Subunit Alpha	Protein Coding	46	GC0XP001333	0.151326045
CXCL2	C-X-C Motif Chemokine Ligand 2	Protein Coding	41	GC04M074097	0.151326045
DBNL	Drebrin Like	Protein Coding	41	GC07P044044	0.151326045
DONSON	DNA Replication Fork Stabilization Factor DONSON	Protein Coding	35	GC21M033559	0.151326045
EHF	ETS Homologous Factor	Protein Coding	41	GC11P034621	0.151326045
EML3	EMAP Like 3	Protein Coding	34	GC11M062602	0.151326045

EPOR	Erythropoietin Receptor	Protein Coding	48	GC19M011377	0.151326045
FBXL7	F-Box And Leucine Rich Repeat Protein 7	Protein Coding	36	GC05P015553	0.151326045
FOXK1	Forkhead Box K1	Protein Coding	35	GC07P004682	0.151326045
FRAXA	Fragile Site, Folic Acid Type, Rare, Fra(X)(Q27.3) A (Macroorchidism, Mental Retardation)	Biological Region	6	GC0XP147926	0.151326045
GRIN2A	Glutamate Ionotropic Receptor NMDA Type Subunit 2A	Protein Coding	52	GC16M009753	0.151326045
GYG1	Glycogenin 1	Protein Coding	47	GC03P148991	0.151326045
H1-8	H1.8 Linker Histone	Protein Coding	29	GC03P131510	0.151326045
H2AC18	H2A Clustered Histone 18	Protein Coding	26	GC01M150124	0.151326045
H2AC19	H2A Clustered Histone 19	Protein Coding	22	GC01P149980	0.151326045
H3C13	H3 Clustered Histone 13	Protein Coding	26	GC01M150118	0.151326045
HAPLN1	Hyaluronan And Proteoglycan Link Protein 1	Protein Coding	43	GC05M083637	0.151326045
HCLS1	Hematopoietic Cell-Specific Lyn Substrate 1	Protein Coding	43	GC03M121631	0.151326045
HEXB	Hexosaminidase Subunit Beta	Protein Coding	48	GC05P074640	0.151326045
HNRNPA2B1	Heterogeneous Nuclear Ribonucleoprotein A2/B1	Protein Coding	47	GC07M026174	0.151326045
HOXA13	Homeobox A13	Protein Coding	44	GC07M027347	0.151326045
INTS11	Integrator Complex Subunit 11	Protein Coding	30	GC01M002704	0.151326045
MAPKAP1	MAPK Associated Protein 1	Protein Coding	44	GC09M125437	0.151326045
MED1	Mediator Complex Subunit 1	Protein Coding	42	GC17M039404	0.151326045
MIR34A	MicroRNA 34a	RNA Gene	22	GC01M009151	0.151326045
MOCS2	Molybdenum Cofactor Synthesis 2	Protein Coding	43	GC05M053095	0.151326045

MTARC2	Mitochondrial Amidoxime Reducing Component 2	Protein Coding	30	GC01P220748	0.151326045
NKPD1	NTPase KAP Family P-Loop Domain Containing 1	Protein Coding	30	GC19M046964	0.151326045
NR1H2	Nuclear Receptor Subfamily 1 Group H Member 2	Protein Coding	49	GC19P050329	0.151326045
NR4A3	Nuclear Receptor Subfamily 4 Group A Member 3	Protein Coding	47	GC09P099821	0.151326045
ORC5	Origin Recognition Complex Subunit 5	Protein Coding	37	GC07M104126	0.151326045
PAH	Phenylalanine Hydroxylase	Protein Coding	49	GC12M102836	0.151326045
PF4V1	Platelet Factor 4 Variant 1	Protein Coding	36	GC04P073853	0.151326045
PLCB2	Phospholipase C Beta 2	Protein Coding	47	GC15M040278	0.151326045
PMAIP1	Phorbol-12-Myristate-13-Acetate-Induced Protein 1	Protein Coding	40	GC18P059899	0.151326045
PRIM1	DNA Primase Subunit 1	Protein Coding	43	GC12M056731	0.151326045
PRIMPOL	Primase And DNA Directed Polymerase	Protein Coding	33	GC04P184649	0.151326045
RNASE4	Ribonuclease A Family Member 4	Protein Coding	34	GC14P021584	0.151326045
RNU4-5P	RNA, U4 Small Nuclear 5, Pseudogene	Pseudogene	9	GC10P109869	0.151326045
RPE	Ribulose-5-Phosphate-3-Epimerase	Protein Coding	43	GC02P210002	0.151326045
RPL29	Ribosomal Protein L29	Protein Coding	39	GC03M052012	0.151326045
SAMHD1	SAM And HD Domain Containing Deoxynucleoside Triphosphate Triphosphohydrolase 1	Protein Coding	43	GC20M036890	0.151326045

SCLY	Selenocysteine Lyase	Protein Coding	41	GC02P238061	0.151326045
SFXN1	Sideroflexin 1	Protein Coding	40	GC05P175477	0.151326045
SFXN2	Sideroflexin 2	Protein Coding	40	GC10P102714	0.151326045
SFXN3	Sideroflexin 3	Protein Coding	39	GC10P101031	0.151326045
SFXN4	Sideroflexin 4	Protein Coding	41	GC10M119140	0.151326045
SFXN5	Sideroflexin 5	Protein Coding	40	GC02M072943	0.151326045
SMUG1	Single-Strand-Selective Monofunctional Uracil-DNA Glycosylase 1	Protein Coding	43	GC12M054121	0.151326045
SYNCRIP	Synaptotagmin Binding Cytoplasmic RNA Interacting Protein	Protein Coding	39	GC06M085607	0.151326045
TAS1R3	Taste 1 Receptor Member 3	Protein Coding	40	GC01P001331	0.151326045
TCTE1	T-Complex-Associated-Testis-Expressed 1	Protein Coding	36	GC06M044278	0.151326045
TEAD1	TEA Domain Transcription Factor 1	Protein Coding	48	GC11P012674	0.151326045
TFCP2	Transcription Factor CP2	Protein Coding	41	GC12M051093	0.151326045
TSN	Translin	Protein Coding	41	GC02P121737	0.151326045
UPF1	UPF1 RNA Helicase And ATPase	Protein Coding	41	GC19P018831	0.151326045
USP9X	Ubiquitin Specific Peptidase 9 X-Linked	Protein Coding	48	GC0XP041085	0.151326045
WDR1	WD Repeat Domain 1	Protein Coding	41	GC04M010075	0.151326045
XIST	X Inactive Specific Transcript	RNA Gene	24	GC0XM073820	0.151326045
YAP1	Yes1 Associated Transcriptional Regulator	Protein Coding	48	GC11P102110	0.151326045
YTHDC1	YTH Domain Containing 1	Protein Coding	37	GC04M068310	0.151326045
ACRV1	Acrosomal Vesicle Protein 1	Protein Coding	34	GC11M125671	0.107003674
ACTN4	Actinin Alpha 4	Protein Coding	47	GC19P038647	0.107003674

ALG2	ALG2 Alpha-1,3/1,6-Mannosyltransferase	Protein Coding	42	GC09M099216	0.107003674
ANP32A	Acidic Nuclear Phosphoprotein 32 Family Member A	Protein Coding	43	GC15M068778	0.107003674
ANXA10	Annexin A10	Protein Coding	37	GC04P168081	0.107003674
APEX2	Apurinic/Apyrimidinic Endodeoxyribonuclease 2	Protein Coding	38	GC0XP055000	0.107003674
APOL1	Apolipoprotein L1	Protein Coding	43	GC22P036253	0.107003674
AQP1	Aquaporin 1 (Colton Blood Group)	Protein Coding	47	GC07P030911	0.107003674
AQP10	Aquaporin 10	Protein Coding	37	GC01P154321	0.107003674
AQP11	Aquaporin 11	Protein Coding	36	GC11P077589	0.107003674
AQP4	Aquaporin 4	Protein Coding	47	GC18M026852	0.107003674
AQP5	Aquaporin 5	Protein Coding	47	GC12P049961	0.107003674
AQP7	Aquaporin 7	Protein Coding	45	GC09M033384	0.107003674
AQP8	Aquaporin 8	Protein Coding	37	GC16P026741	0.107003674
ARHGEF12	Rho Guanine Nucleotide Exchange Factor 12	Protein Coding	44	GC11P120336	0.107003674
ARHGEF16	Rho Guanine Nucleotide Exchange Factor 16	Protein Coding	40	GC01P003454	0.107003674
ARHGEF7	Rho Guanine Nucleotide Exchange Factor 7	Protein Coding	44	GC13P111114	0.107003674
ARL14	ADP Ribosylation Factor Like GTPase 14	Protein Coding	31	GC03P160677	0.107003674
ASS1P7	Argininosuccinate Synthetase 1 Pseudogene 7	Pseudogene	8	GC03M177296	0.107003674
ASTN2	Astrotactin 2	Protein Coding	37	GC09M116425	0.107003674
BBC3	BCL2 Binding Component 3	Protein Coding	41	GC19M047220	0.107003674
BCAR3	BCAR3 Adaptor Protein, NSP Family Member	Protein Coding	40	GC01M093561	0.107003674
BTN1A1	Butyrophilin Subfamily 1 Member A1	Protein Coding	40	GC06P026500	0.107003674

BZW2	Basic Leucine Zipper And W2 Domains 2	Protein Coding	40	GC07P016646	0.107003674
C19orf48	Chromosome 19 Open Reading Frame 48	Pseudogene	33	GC19M050797	0.107003674
CACNA1S	Calcium Voltage-Gated Channel Subunit Alpha1 S	Protein Coding	49	GC01M201008	0.107003674
CAPZB	Capping Actin Protein Of Muscle Z-Line Subunit Beta	Protein Coding	41	GC01M019339	0.107003674
CBR1	Carbonyl Reductase 1	Protein Coding	47	GC21P036069	0.107003674
CCL20	C-C Motif Chemokine Ligand 20	Protein Coding	44	GC02P227814	0.107003674
CCL27	C-C Motif Chemokine Ligand 27	Protein Coding	38	GC09M034662	0.107003674
CCR6	C-C Motif Chemokine Receptor 6	Protein Coding	45	GC06P167111	0.107003674
CD80	CD80 Molecule	Protein Coding	42	GC03M119524	0.107003674
CD86	CD86 Molecule	Protein Coding	44	GC03P122055	0.107003674
CDC42BPB	CDC42 Binding Protein Kinase Beta	Protein Coding	41	GC14M102932	0.107003674
CDH10	Cadherin 10	Protein Coding	40	GC05M024522	0.107003674
CEP170	Centrosomal Protein 170	Protein Coding	40	GC01M243124	0.107003674
CLC	Charcot-Leyden Crystal Galectin	Protein Coding	40	GC19M046744	0.107003674
CLCN5	Chloride Voltage-Gated Channel 5	Protein Coding	44	GC0XP049922	0.107003674
CLCNKA	Chloride Voltage-Gated Channel Ka	Protein Coding	41	GC01P016018	0.107003674
CLTC	Clathrin Heavy Chain	Protein Coding	47	GC17P059619	0.107003674
CNN2	Calponin 2	Protein Coding	39	GC19P001026	0.107003674
CNOT6L	CCR4-NOT Transcription Complex Subunit 6 Like	Protein Coding	35	GC04M077713	0.107003674
CUL5	Cullin 5	Protein Coding	45	GC11P108008	0.107003674
CUX1	Cut Like Homeobox 1	Protein Coding	44	GC07P101815	0.107003674

CUX2	Cut Like Homeobox 2	Protein Coding	41	GC12P111034	0.107003674
CYTH1	Cytohesin 1	Protein Coding	43	GC17M078674	0.107003674
DCDC2	Doublecortin Domain Containing 2	Protein Coding	40	GC06M024171	0.107003674
DEFA1	Defensin Alpha 1	Protein Coding	41	GC08M006977	0.107003674
DEFB1	Defensin Beta 1	Protein Coding	40	GC08M006870	0.107003674
DEK	DEK Proto-Oncogene	Protein Coding	41	GC06M018224	0.107003674
DGUOK-AS1	DGUOK Antisense RNA 1	RNA Gene	16	GC02M073947	0.107003674
DHFRP1	Dihydrofolate Reductase Pseudogene 1	Pseudogene	9	GC18M026167	0.107003674
DNPEP	Aspartyl Aminopeptidase	Protein Coding	40	GC02M219373	0.107003674
DOCK6	Dedicator Of Cytokinesis 6	Protein Coding	40	GC19M011199	0.107003674
DOCK7	Dedicator Of Cytokinesis 7	Protein Coding	42	GC01M062454	0.107003674
EDC3	Enhancer Of MRNA Decapping 3	Protein Coding	43	GC15M074631	0.107003674
EEF1B2	Eukaryotic Translation Elongation Factor 1 Beta 2	Protein Coding	43	GC02P206159	0.107003674
EEF1D	Eukaryotic Translation Elongation Factor 1 Delta	Protein Coding	43	GC08M143579	0.107003674
EEF1G	Eukaryotic Translation Elongation Factor 1 Gamma	Protein Coding	41	GC11M069243	0.107003674
EFNA1	Ephrin A1	Protein Coding	44	GC01P155127	0.107003674
EGR3	Early Growth Response 3	Protein Coding	40	GC08M022687	0.107003674
ELF5	E74 Like ETS Transcription Factor 5	Protein Coding	38	GC11M034500	0.107003674
EPHX2	Epoxide Hydrolase 2	Protein Coding	48	GC08P027490	0.107003674
ERG	ETS Transcription Factor ERG	Protein Coding	46	GC21M038367	0.107003674
EXOSC4	Exosome Component 4	Protein Coding	38	GC08P144079	0.107003674
EXOSC9	Exosome Component 9	Protein Coding	40	GC04P121801	0.107003674

FAH	Fumarylacetoacetate Hydrolase	Protein Coding	47	GC15P080152	0.107003674
FBXW7	F-Box And WD Repeat Domain Containing 7	Protein Coding	45	GC04M152321	0.107003674
FLNB	Filamin B	Protein Coding	47	GC03P058008	0.107003674
FN1	Fibronectin 1	Protein Coding	50	GC02M215360	0.107003674
FN3KRP	Fructosamine 3 Kinase Related Protein	Protein Coding	40	GC17P082716	0.107003674
FOXN1	Forkhead Box N1	Protein Coding	42	GC17P028506	0.107003674
FUS	FUS RNA Binding Protein	Protein Coding	45	GC16P031180	0.107003674
GAA	Alpha Glucosidase	Protein Coding	48	GC17P080101	0.107003674
GAN	Gigaxonin	Protein Coding	40	GC16P081319	0.107003674
GATA5	GATA Binding Protein 5	Protein Coding	40	GC20M062464	0.107003674
GATA6	GATA Binding Protein 6	Protein Coding	48	GC18P022169	0.107003674
GCLM	Glutamate-Cysteine Ligase Modifier Subunit	Protein Coding	43	GC01M093885	0.107003674
GFM1	G Elongation Factor Mitochondrial 1	Protein Coding	45	GC03P158644	0.107003674
GH-LCR	Growth Hormone Locus Control Region	Biological Region	2	GC17P063917	0.107003674
GIT1	GIT ArfGAP 1	Protein Coding	44	GC17M029573	0.107003674
GMDS	GDP-Mannose 4,6-Dehydratase	Protein Coding	45	GC06M001624	0.107003674
GNL3	G Protein Nucleolar 3	Protein Coding	41	GC03P052681	0.107003674
GPD1	Glycerol-3-Phosphate Dehydrogenase 1	Protein Coding	46	GC12P050105	0.107003674
GRB10	Growth Factor Receptor Bound Protein 10	Protein Coding	43	GC07M050590	0.107003674
GRIA1	Glutamate Ionotropic Receptor AMPA Type Subunit 1	Protein Coding	50	GC05P153467	0.107003674
H2BC18	H2B Clustered Histone 18	Protein Coding	27	GC01M150113	0.107003674
H3-2	H3.2 Histone (Putative)	Protein Coding	17	GC01M143894	0.107003674
HAO1	Hydroxyacid Oxidase 1	Protein Coding	44	GC20M007863	0.107003674

HDAC4	Histone Deacetylase 4	Protein Coding	52	GC02M239048	0.107003674
HDGF	Heparin Binding Growth Factor	Protein Coding	41	GC01M156786	0.107003674
HNRNPDL	Heterogeneous Nuclear Ribonucleoprotein D Like	Protein Coding	40	GC04M082422	0.107003674
HNRNPK	Heterogeneous Nuclear Ribonucleoprotein K	Protein Coding	47	GC09M086093	0.107003674
HNRNPL	Heterogeneous Nuclear Ribonucleoprotein L	Protein Coding	40	GC19M038836	0.107003674
HSD17B3	Hydroxysteroid 17-Beta Dehydrogenase 3	Protein Coding	48	GC09M096240	0.107003674
HSD3B2	Hydroxy-Delta-5-Steroid Dehydrogenase, 3 Beta- And Steroid Delta-Isomerase 2	Protein Coding	47	GC01P119414	0.107003674
HSPA13	Heat Shock Protein Family A (Hsp70) Member 13	Protein Coding	37	GC21M014372	0.107003674
IGKC	Immunoglobulin Kappa Constant	Protein Coding	33	GC02M090112	0.107003674
IL21	Interleukin 21	Protein Coding	43	GC04M122612	0.107003674
IL23A	Interleukin 23 Subunit Alpha	Protein Coding	40	GC12P056440	0.107003674
IL3	Interleukin 3	Protein Coding	44	GC05P132060	0.107003674
IL5RA	Interleukin 5 Receptor Subunit Alpha	Protein Coding	47	GC03M003066	0.107003674
IPO5	Importin 5	Protein Coding	40	GC13P097953	0.107003674
IRS1	Insulin Receptor Substrate 1	Protein Coding	48	GC02M226731	0.107003674
ISCA1P1	Iron-Sulfur Cluster Assembly 1 Pseudogene 1	Pseudogene	9	GC05M062775	0.107003674
IVNS1ABP	Influenza Virus NS1A Binding Protein	Protein Coding	40	GC01M185299	0.107003674

KCTD3	Potassium Channel Tetramerization Domain Containing 3	Protein Coding	37	GC01P215567	0.107003674
KHSRP	KH-Type Splicing Regulatory Protein	Protein Coding	43	GC19M006413	0.107003674
KLC2	Kinesin Light Chain 2	Protein Coding	43	GC11P066257	0.107003674
KLHL5	Kelch Like Family Member 5	Protein Coding	36	GC04P039044	0.107003674
KLHL6	Kelch Like Family Member 6	Protein Coding	36	GC03M183487	0.107003674
KLRC3	Killer Cell Lectin Like Receptor C3	Protein Coding	35	GC12M015690	0.107003674
LAMA1	Laminin Subunit Alpha 1	Protein Coding	47	GC18M006941	0.107003674
LASP1	LIM And SH3 Protein 1	Protein Coding	43	GC17P038869	0.107003674
LCN2	Lipocalin 2	Protein Coding	44	GC09P128149	0.107003674
LDHB	Lactate Dehydrogenase B	Protein Coding	47	GC12M021635	0.107003674
LGALS3BP	Galectin 3 Binding Protein	Protein Coding	41	GC17M078971	0.107003674
LGALS7	Galectin 7	Protein Coding	38	GC19M038770	0.107003674
LINC01428	Long Intergenic Non-Protein Coding RNA 1428	RNA Gene	10	GC20M007146	0.107003674
LMNB1	Lamin B1	Protein Coding	48	GC05P126776	0.107003674
LOC102724058	Uncharacterized LOC102724058	RNA Gene	6	GC02P165958	0.107003674
LOC106694315	MPO Proximal Enhancer And Promoter Region	Biological Region	2	GC17P058279	0.107003674
LOC107133510	Origin Of Replication At HBB	Biological Region	2	GC11P005222	0.107003674
LOC108510657	Friedreich Ataxia Repeat Instability Region	Biological Region	2	GC09P069037	0.107003674
LOC110973015	NOS3 5' Regulatory Region	Biological Region	2	GC07P150988	0.107003674
LORICRIN	Loricrin Cornified Envelope Precursor Protein	Protein Coding	28	GC01P153262	0.107003674

LPIN1	Lipin 1	Protein Coding	48	GC02P011677	0.107003674
MAP1B	Microtubule Associated Protein 1B	Protein Coding	44	GC05P072107	0.107003674
MIR181A1	MicroRNA 181a-1	RNA Gene	19	GC01M198860	0.107003674
MIR203A	MicroRNA 203a	RNA Gene	19	GC14P106054	0.107003674
MMP16	Matrix Metallopeptidase 16	Protein Coding	45	GC08M088032	0.107003674
MPRIP	Myosin Phosphatase Rho Interacting Protein	Protein Coding	38	GC17P017042	0.107003674
MRS2	Magnesium Transporter MRS2	Protein Coding	38	GC06P024402	0.107003674
MT2A	Metallothionein 2A	Protein Coding	43	GC16P056658	0.107003674
MT-TI	Mitochondrially Encoded TRNA-Ile (AUU/C)	RNA Gene	13	GCMTP004265	0.107003674
MYF6	Myogenic Factor 6	Protein Coding	43	GC12P080707	0.107003674
MYH3	Myosin Heavy Chain 3	Protein Coding	44	GC17M010628	0.107003674
MYO18B	Myosin XVIIIB	Protein Coding	39	GC22P025742	0.107003674
MYOC	Myocilin	Protein Coding	43	GC01M171604	0.107003674
NEAT1	Nuclear Paraspeckle Assembly Transcript 1	RNA Gene	23	GC11P066222	0.107003674
NEIL3	Nei Like DNA Glycosylase 3	Protein Coding	38	GC04P177309	0.107003674
NFIC	Nuclear Factor I C	Protein Coding	41	GC19P003314	0.107003674
NFU1	NFU1 Iron-Sulfur Cluster Scaffold	Protein Coding	43	GC02M069395	0.107003674
NMB	Neuromedin B	Protein Coding	42	GC15M084655	0.107003674
NPTX2	Neuronal Pentraxin 2	Protein Coding	40	GC07P098620	0.107003674
NR5A2	Nuclear Receptor Subfamily 5 Group A Member 2	Protein Coding	47	GC01P199996	0.107003674
NUDC	Nuclear Distribution C, Dynein Complex Regulator	Protein Coding	43	GC01P026925	0.107003674
NUFIP1	Nuclear FMR1 Interacting Protein 1	Protein Coding	35	GC13M044939	0.107003674
OGFR	Opioid Growth Factor Receptor	Protein Coding	38	GC20P062804	0.107003674

OPTN	Optineurin	Protein Coding	45	GC10P013099	0.107003674
ORC1	Origin Recognition Complex Subunit 1	Protein Coding	44	GC01M052372	0.107003674
ORC2	Origin Recognition Complex Subunit 2	Protein Coding	40	GC02M200908	0.107003674
ORC4	Origin Recognition Complex Subunit 4	Protein Coding	43	GC02M147930	0.107003674
OTUB1	OTU Deubiquitinase, Ubiquitin Aldehyde Binding 1	Protein Coding	40	GC11P063985	0.107003674
OXT	Oxytocin/Neurophysin I Prepropeptide	Protein Coding	41	GC20P003068	0.107003674
PARK7	Parkinsonism Associated Deglycase	Protein Coding	47	GC01P008012	0.107003674
PCBP1	Poly(RC) Binding Protein 1	Protein Coding	43	GC02P070087	0.107003674
PCSK7	Proprotein Convertase Subtilisin/Kexin Type 7	Protein Coding	44	GC11M117199	0.107003674
PDIA6	Protein Disulfide Isomerase Family A Member 6	Protein Coding	41	GC02M010784	0.107003674
PEF1	Penta-EF-Hand Domain Containing 1	Protein Coding	38	GC01M031630	0.107003674
PGLS	6-Phosphogluconolactonase	Protein Coding	39	GC19P026640	0.107003674
PLAT	Plasminogen Activator, Tissue Type	Protein Coding	50	GC08M042174	0.107003674
PLEKHB1	Pleckstrin Homology Domain Containing B1	Protein Coding	37	GC11P073647	0.107003674
PLIN3	Perilipin 3	Protein Coding	41	GC19M004839	0.107003674
POLM	DNA Polymerase Mu	Protein Coding	40	GC07M044143	0.107003674

POMGNT2	Protein O-Linked Mannose N-Acetylglucosaminyltransferase 2 (Beta 1,4-)	Protein Coding	37	GC03M043121	0.107003674
POU4F1	POU Class 4 Homeobox 1	Protein Coding	39	GC13M078598	0.107003674
PPM1L	Protein Phosphatase, Mg2+/Mn2+ Dependent 1L	Protein Coding	38	GC03P160755	0.107003674
PPME1	Protein Phosphatase Methylesterase 1	Protein Coding	39	GC11P074170	0.107003674
PPP1CA	Protein Phosphatase 1 Catalytic Subunit Alpha	Protein Coding	49	GC11M069512	0.107003674
PPP2CB	Protein Phosphatase 2 Catalytic Subunit Beta	Protein Coding	46	GC08M030762	0.107003674
PPP2R1B	Protein Phosphatase 2 Scaffold Subunit Abeta	Protein Coding	47	GC11M111695	0.107003674
PRDX1	Peroxiredoxin 1	Protein Coding	50	GC01M045511	0.107003674
PRF1	Perforin 1	Protein Coding	45	GC10M070597	0.107003674
PRICKLE4	Prickle Planar Cell Polarity Protein 4	Protein Coding	36	GC06P041780	0.107003674
PRL	Prolactin	Protein Coding	44	GC06M022287	0.107003674
PSMB9	Proteasome 20S Subunit Beta 9	Protein Coding	47	GC06P055233	0.107003674
PSMC1	Proteasome 26S Subunit, ATPase 1	Protein Coding	43	GC14P090256	0.107003674
PSMC2	Proteasome 26S Subunit, ATPase 2	Protein Coding	41	GC07P103344	0.107003674
PSMC5	Proteasome 26S Subunit, ATPase 5	Protein Coding	43	GC17P063827	0.107003674
PSMD1	Proteasome 26S Subunit, Non-ATPase 1	Protein Coding	40	GC02P231056	0.107003674
PSMD12	Proteasome 26S Subunit, Non-ATPase 12	Protein Coding	43	GC17M067337	0.107003674

PSMD14	Proteasome 26S Subunit, Non-ATPase 14	Protein Coding	44	GC02P161308	0.107003674
PSME1	Proteasome Activator Subunit 1	Protein Coding	43	GC14P024136	0.107003674
PSME2	Proteasome Activator Subunit 2	Protein Coding	43	GC14M024143	0.107003674
PSPH	Phosphoserine Phosphatase	Protein Coding	47	GC07M056010	0.107003674
PSTPIP1	Proline-Serine-Threonine Phosphatase Interacting Protein 1	Protein Coding	46	GC15P076993	0.107003674
PTPN12	Protein Tyrosine Phosphatase Non-Receptor Type 12	Protein Coding	48	GC07P077537	0.107003674
PTPRG	Protein Tyrosine Phosphatase Receptor Type G	Protein Coding	43	GC03P061561	0.107003674
PTPRM	Protein Tyrosine Phosphatase Receptor Type M	Protein Coding	43	GC18P007557	0.107003674
PUDP	Pseudouridine 5'-Phosphatase	Protein Coding	31	GC0XM006668	0.107003674
RANGAP1	Ran GTPase Activating Protein 1	Protein Coding	43	GC22M041244	0.107003674
RAPSN	Receptor Associated Protein Of The Synapse	Protein Coding	43	GC11M069002	0.107003674
RBM7	RNA Binding Motif Protein 7	Protein Coding	38	GC11P114401	0.107003674
RBMX	RNA Binding Motif Protein X-Linked	Protein Coding	43	GC0XM136848	0.107003674
RCBTB1	RCC1 And BTB Domain Containing Protein 1	Protein Coding	40	GC13M049531	0.107003674
RCC2	Regulator Of Chromosome Condensation 2	Protein Coding	40	GC01M017406	0.107003674
REEP1	Receptor Accessory Protein 1	Protein Coding	41	GC02M086213	0.107003674

RELB	RELB Proto-Oncogene, NF-KB Subunit	Protein Coding	46	GC19P045002	0.107003674
REXO1	RNA Exonuclease 1 Homolog	Protein Coding	36	GC19M001815	0.107003674
RFX4	Regulatory Factor X4	Protein Coding	36	GC12P106583	0.107003674
RFX5	Regulatory Factor X5	Protein Coding	42	GC01M151340	0.107003674
RHBDD1	Rhomboid Domain Containing 1	Protein Coding	36	GC02P226805	0.107003674
RHCE	Rh Blood Group CcEe Antigens	Protein Coding	41	GC01M025360	0.107003674
RP2	RP2 Activator Of ARL3 GTPase	Protein Coding	42	GC0XP046836	0.107003674
RPA4	Replication Protein A4	Protein Coding	37	GC0XP096883	0.107003674
RPIA	Ribose 5-Phosphate Isomerase A	Protein Coding	44	GC02P088691	0.107003674
RPS15	Ribosomal Protein S15	Protein Coding	41	GC19P001438	0.107003674
RPS16	Ribosomal Protein S16	Protein Coding	41	GC19M039433	0.107003674
RPS18	Ribosomal Protein S18	Protein Coding	41	GC06P055262	0.107003674
RPSAP11	Ribosomal Protein SA Pseudogene 11	Pseudogene	7	GC03P032252	0.107003674
SAFB2	Scaffold Attachment Factor B2	Protein Coding	37	GC19M005587	0.107003674
SCN1A	Sodium Voltage-Gated Channel Alpha Subunit 1	Protein Coding	47	GC02M165989	0.107003674
SEC23A	SEC23 Homolog A, COPII Coat Complex Component	Protein Coding	44	GC14M039031	0.107003674
SEC23B	SEC23 Homolog B, COPII Coat Complex Component	Protein Coding	44	GC20P018507	0.107003674
SEC24C	SEC24 Homolog C, COPII Coat Complex Component	Protein Coding	45	GC10P073744	0.107003674
SEMA3C	Semaphorin 3C	Protein Coding	43	GC07M080742	0.107003674
SERPINB1	Serpin Family B Member 1	Protein Coding	40	GC06M002833	0.107003674

SET	SET Nuclear Proto-Oncogene	Protein Coding	47	GC09P128684	0.107003674
SF3B1	Splicing Factor 3b Subunit 1	Protein Coding	45	GC02M197393	0.107003674
SIK2	Salt Inducible Kinase 2	Protein Coding	44	GC11P111633	0.107003674
SLC25A14	Solute Carrier Family 25 Member 14	Protein Coding	41	GC0XP130339	0.107003674
SLC25A27	Solute Carrier Family 25 Member 27	Protein Coding	39	GC06P046652	0.107003674
SLC26A5	Solute Carrier Family 26 Member 5	Protein Coding	44	GC07M103352	0.107003674
SLC2A4	Solute Carrier Family 2 Member 4	Protein Coding	47	GC17P009071	0.107003674
SLC2A6	Solute Carrier Family 2 Member 6	Protein Coding	42	GC09M133471	0.107003674
SMC4	Structural Maintenance Of Chromosomes 4	Protein Coding	40	GC03P160399	0.107003674
SMYD1	SET And MYND Domain Containing 1	Protein Coding	40	GC02P088068	0.107003674
SND1	Staphylococcal Nuclease And Tudor Domain Containing 1	Protein Coding	43	GC07P127652	0.107003674
SON	SON DNA And RNA Binding Protein	Protein Coding	40	GC21P033542	0.107003674
SPICE1	Spindle And Centriole Associated Protein 1	Protein Coding	34	GC03M113442	0.107003674
SRGAP2	SLIT-ROBO Rho GTPase Activating Protein 2	Protein Coding	37	GC01P206203	0.107003674
SRP19	Signal Recognition Particle 19	Protein Coding	40	GC05P112862	0.107003674
SRP54	Signal Recognition Particle 54	Protein Coding	44	GC14P034981	0.107003674
SRSF4	Serine And Arginine Rich Splicing Factor 4	Protein Coding	39	GC01M029147	0.107003674

STAT5B	Signal Transducer And Activator Of Transcription 5B	Protein Coding	50	GC17M042199	0.107003674
STAU1	Staufen Double-Stranded RNA Binding Protein 1	Protein Coding	38	GC20M049113	0.107003674
SYNE1	Spectrin Repeat Containing Nuclear Envelope Protein 1	Protein Coding	41	GC06M152121	0.107003674
TACR2	Tachykinin Receptor 2	Protein Coding	44	GC10M069403	0.107003674
TAGLN2	Transgelin 2	Protein Coding	41	GC01M159918	0.107003674
TBC1D4	TBC1 Domain Family Member 4	Protein Coding	44	GC13M075284	0.107003674
TCEA1	Transcription Elongation Factor A1	Protein Coding	38	GC08M053966	0.107003674
TFG	Trafficking From ER To Golgi Regulator	Protein Coding	45	GC03P100709	0.107003674
TNS1	Tensin 1	Protein Coding	40	GC02M217799	0.107003674
TOMM6	Translocase Of Outer Mitochondrial Membrane 6	Protein Coding	31	GC06P041787	0.107003674
TPM3	Tropomyosin 3	Protein Coding	47	GC01M154127	0.107003674
TRIM28	Tripartite Motif Containing 28	Protein Coding	44	GC19P058544	0.107003674
TUBA1A	Tubulin Alpha 1a	Protein Coding	49	GC12M049184	0.107003674
TUBB3	Tubulin Beta 3 Class III	Protein Coding	50	GC16P089919	0.107003674
TXNDC12	Thioredoxin Domain Containing 12	Protein Coding	38	GC01M052020	0.107003674
TXNDC5	Thioredoxin Domain Containing 5	Protein Coding	39	GC06M007893	0.107003674
TXNL1	Thioredoxin Like 1	Protein Coding	40	GC18M056597	0.107003674
U2AF1	U2 Small Nuclear RNA Auxiliary Factor 1	Protein Coding	44	GC21M043092	0.107003674
UBXN1	UBX Domain Protein 1	Protein Coding	36	GC11M069259	0.107003674
UGDH	UDP-Glucose 6-Dehydrogenase	Protein Coding	47	GC04M039502	0.107003674

UGGT1	UDP-Glucose Glycoprotein Glucosyltransferase 1	Protein Coding	38	GC02P128091	0.107003674
UGT2B15	UDP Glucuronosyltransferase Family 2 Member B15	Protein Coding	43	GC04M068646	0.107003674
UGT2B28	UDP Glucuronosyltransferase Family 2 Member B28	Protein Coding	40	GC04P069280	0.107003674
USP11	Ubiquitin Specific Peptidase 11	Protein Coding	43	GC0XP047232	0.107003674
USP47	Ubiquitin Specific Peptidase 47	Protein Coding	39	GC11P011819	0.107003674
VCX3A	Variable Charge X-Linked 3A	Protein Coding	29	GC0XM006533	0.107003674
VPS13A	Vacuolar Protein Sorting 13 Homolog A	Protein Coding	41	GC09P077177	0.107003674
VPS33A	VPS33A Core Subunit Of CORVET And HOPS Complexes	Protein Coding	41	GC12M122229	0.107003674
VPS33B	VPS33B Late Endosome And Lysosome Associated	Protein Coding	42	GC15M090998	0.107003674
VPS45	Vacuolar Protein Sorting 45 Homolog	Protein Coding	40	GC01P150068	0.107003674
VSTM4	V-Set And Transmembrane Domain Containing 4	Protein Coding	32	GC10M049014	0.107003674
XPR1	Xenotropic And Polytropic Retrovirus Receptor 1	Protein Coding	44	GC01P180632	0.107003674
YBX3	Y-Box Binding Protein 3	Protein Coding	40	GC12M015696	0.107003674
YWHAE	Tyrosine 3-Monooxygenase/Tryptophan 5-Monooxygenase Activation Protein Epsilon	Protein Coding	50	GC17M001346	0.107003674

ZC3H11A	Zinc Finger CCCH-Type Containing 11A	Protein Coding	37	GC01P203795	0.107003674
ZEB1	Zinc Finger E-Box Binding Homeobox 1	Protein Coding	49	GC10P031318	0.107003674
ZYX	Zyxin	Protein Coding	46	GC07P143381	0.107003674
