# Supplementary material for: NEK6 dampens FOXO3 nuclear translocation to stabilize C-MYC and promotes subsequent de novo purine synthesis to support ovarian cancer chemoresistance
Source: Cell Death Dis. 2024 Sep 10;15(9):661. doi: 10.1038/s41419-024-07045-2 (PMC11387829; doi:10.1038/s41419-024-07045-2)
Supplement: Supplementary file 5 — Supplementary Table 4 [file 41419_2024_7045_MOESM5_ESM.pdf]

Supplementary Table 4. Characteristics of ovarian cancer patients

Patient	Age	Pathology	Stage	Drug treatments (post-initial cytoreductive surgery)	Chemotherapy cycle (post-initial cytoreductive Surgery)
Patient #S1	46	High grade serous carcinoma	IIIc	Paclitaxel/cisplatin	5
Patient #S2	52	High grade serous carcinoma	IIIb	Paclitaxel/cisplatin	5
Patient #S3	54	High grade clear cell carcinoma	Ia	Paclitaxel/carboplatin	6
Patient #S4	56	High grade serous carcinoma	IIIc	Paclitaxel/carboplatin	4
Patient #S5	68	High grade serous carcinoma	IIc	Paclitaxel/carboplatin	5
Patient #S6	62	Mucinous carcinoma	Ic	Paclitaxel/carboplatin	5
Patient #R1	45	High grade serous carcinoma	IIIc	Paclitaxel/carboplatin	6
Patient #R2	55	High grade serous carcinoma	IIIc	Paclitaxel/cisplatin	8
Patient #R3	60	High grade serous carcinoma	IVA	Paclitaxel/cisplatin	7
Patient #R4	63	High grade serous carcinoma	IIIc	Paclitaxel/carboplatin	6
Patient #R5	53	High grade clear cell carcinoma	IIIc	Paclitaxel/cisplatin	6
Patient #R6	38	High grade papillary serous carcinoma	IIIc	Paclitaxel/cisplatin	6
