# Supplementary material for: NEK6 dampens FOXO3 nuclear translocation to stabilize C-MYC and promotes subsequent de novo purine synthesis to support ovarian cancer chemoresistance
Source: Cell Death Dis. 2024 Sep 10;15(9):661. doi: 10.1038/s41419-024-07045-2 (PMC11387829; doi:10.1038/s41419-024-07045-2)
Supplement: Supplementary file 6 — Supplementary Table 5 [file 41419_2024_7045_MOESM6_ESM.pdf]

Supplementary Table 5. Purine metabolite abundance in chemosensitive and chemoresistant ovarian cancer tissues

#group	Chemosensitive	Chemosensitive	Chemosensitive	Chemosensitive	Chemoresistanc	Chemoresistanc	Chemoresistanc	Chemoresistanc
#sample	S1	S2	S3	S4	R1	R2	R3	R4
2’-Deoxyguanosine 5’-phosphate	508878198	296790443	934651647	579575583	1606669410	2330924963	1391921535	2182102836
2-hydroxy-dAMP	483296944	209267051	601042025	320272185	968317443	1306104514	831841166	1209593804
8-Oxo-dGMP	86060529	34834104	122949301	45320430	220168456	318771274	195347021	304266636
Adenosine	32053312	19922666	47043812	26562880	83767264	72638238	67977748	94632432
Adenosine phosphosulfate	148986983	126751535	107565923	94833918	152160865	100832710	137820525	129002368
ADP	53505784	43887020	35421838	22743716	33668541	36832832	40369279	43429761
AMP	31448953	22266498	65891768	41417969	129415158	156598900	106163146	151346545
Arabinosylhypoxanthine	173228081	67526726	229534187	334569260	188885166	55534709	301275798	307640156
GMP	59866267	28888566	124979424	48374381	202040729	305586594	197813948	303711812
Guanosine	20680305	16764016	22160825	22469187	22739694	26534247	26479174	23963506
Inosine	26060781	36847008	25861538	20686032	30932121	76998223	130069070	160013570
Vidarabine	26440353	21243546	65404413	26642599	6993453	893831605	2263597848	1527176711
IMP	10847139	20814988	18653996	28907594	43031679	48105836	29532707	29207604
Zeatin riboside	4875250	1581849	6954765	9531293	17585770	41276711	5587811	6332644
