# Supplementary material for: NEK6 dampens FOXO3 nuclear translocation to stabilize C-MYC and promotes subsequent de novo purine synthesis to support ovarian cancer chemoresistance
Source: Cell Death Dis. 2024 Sep 10;15(9):661. doi: 10.1038/s41419-024-07045-2 (PMC11387829; doi:10.1038/s41419-024-07045-2)
Supplement: Supplementary file 7 — Supplementary Table 6 [file 41419_2024_7045_MOESM7_ESM.pdf]

Supplementary Table 6. Purine metabolite abundance in OVCAR8 and NCI/ADR-RES cells

#group	OVCAR8	OVCAR8	OVCAR8	OVCAR8	OVCAR8	OVCAR8	NCI/ADR-RES	NCI/ADR-RES	NCI/ADR-RES	NCI/ADR-RES	NCI/ADR-RES	NCI/ADR-RES
#sample	1	2	3	4	5	6	1	2	3	4	5	6
6-Mercaptopurine_ribonucleoside 5’-diphosphate	1144	4608	3047	1456	2961	2479	7218	6152	5816	4294	4754	5623
6-Thioinosine-5’-monophosphate	8439	20636	12027	5136	11932	14513	4771	9073	10926	5995	11550	10386
7-Methylguanosine_5’-diphosphate	294	523	100	169	453	110	3757	4172	2649	1751	3448	2988
Adenosine	1458	3583	1847	3198	3751	2456	4976	3014	4668	2905	6297	8585
AMP	11143	14104	10883	1485	11594	12387	14108	13845	13463	14520	19290	12949
Adenosine_phosphosulfate	1756	3326	2296	1496	1839	2010	4582	4518	3249	2342	2711	3755
ADP-ribose 2’-phosphate	5600	10848	9721	10239	9465	11096	10941	10310	9112	9466	9165	8133
Clofarabine	10391	62174	22985	11816	24691	22957	23469	51822	65529	19066	38542	54096
dADP	1382	9022	4711	3220	4951	9158	1433	14729	11188	3203	16241	5314
dAMP	9495	47862	25001	21571	10981	31074	13271	25650	55789	12351	28104	47421
IMP	2139	5719	3392	2882	2537	4455	11665	13368	18104	6824	9393	11754
Guanosine	1729	1749	1887	1716	1291	1550	2341	2485	2016	2307	2056	1772
IDP	2057	7733	3753	1667	3669	3017	3328	7095	6580	3534	5190	6881
Inosine	3443	9966	7204	9559	7836	9524	16123	8617	10633	10084	17464	9552
GMP	563	1348	1132	780	1473	3144	4330	2818	2809	2043	1287	3084
