# Supplementary material for: NEK6 dampens FOXO3 nuclear translocation to stabilize C-MYC and promotes subsequent de novo purine synthesis to support ovarian cancer chemoresistance
Source: Cell Death Dis. 2024 Sep 10;15(9):661. doi: 10.1038/s41419-024-07045-2 (PMC11387829; doi:10.1038/s41419-024-07045-2)
Supplement: Supplementary file 8 — Supplementary Table 7 [file 41419_2024_7045_MOESM8_ESM.pdf]

Supplementary Table 7. Purine metabolite abundance in SKOV3 and SKOV3/DDP cells

#group	SKOV3	SKOV3	SKOV3	SKOV3	SKOV3	SKOV3	SKOV3/DDP	SKOV3/DDP	SKOV3/DDP	SKOV3/DDP	SKOV3/DDP	SKOV3/DDP
#sample	1	2	3	4	5	6	1	2	3	4	5	6
2-Hydroxy-6-Aminopurine	60509	54338	38775	130060	30520	20822	112680	126590	194270	253720	136570	183650
5'-Deoxy-5'-(Methylthio) Adenosine	271120	230780	335050	229450	273510	182790	3557800	2695500	1615400	2118500	1900300	1997500
5'-deoxy-5'-fluoroadenosine	465610	332880	237560	406660	400240	194480	2114900	2385300	2261500	2744000	1514000	2147700
5'-Deoxyadenosine	244250	148150	14603	39755	78726	16654	679070	996200	696960	594060	497500	762780
AMP	97298	72325	17483	55706	68215	38553	69432	375680	294940	390610	118140	199900
Adenosine	65078000	52212000	39163000	57742000	63035000	27882000	269800000	273850000	262410000	295650000	203250000	271390000
cAmp	5160	9041	11010	7466	11616	5962	98066	93314	80400	127370	57737	59006
Cyclic ADP ribose	30901	19143	147280	21964	39570	33793	468870	513490	345920	561040	269320	436150
Deoxyadenosine	244250	148150	14603	39755	78726	16654	679070	996200	696960	594060	497500	762780
GMP	60509	54338	38775	130060	30520	20822	112680	126590	194270	253720	136570	183650
Guanosine	298140	152710	226740	255580	217260	146640	998330	2632700	2404800	2612100	1504100	2140200
IMP	32259	15350	6566	14604	17622	10211	54167	1042300	406250	1075800	159420	193850
Inosine	4377900	3630800	2690500	3836800	3852100	2013900	21414000	23962000	22285000	24776000	16833000	22663000
Nicotinic Acid Adenine Dinucleotide	6610.5	2926.6	82861	4376.3	13891	20401	206620	231110	254610	375940	100760	140580
Xanthosine	18268	20410	24022	19363	17113	11382	79336	217250	181290	201050	116730	187040
