# Supplementary material for: NEK6 dampens FOXO3 nuclear translocation to stabilize C-MYC and promotes subsequent de novo purine synthesis to support ovarian cancer chemoresistance
Source: Cell Death Dis. 2024 Sep 10;15(9):661. doi: 10.1038/s41419-024-07045-2 (PMC11387829; doi:10.1038/s41419-024-07045-2)
Supplement: Supplementary file 9 — Supplementary Table 8 [file 41419_2024_7045_MOESM9_ESM.pdf]

Supplementary Table 8. Purine metabolite abundance in NCI/ADR-RES cells after introduction of NEK6 knockdown and C-MYC overexpression

#group	shCtrl	shCtrl	shCtrl	shCtrl	shCtrl	shCtrl	shNEK6#3	shNEK6#3	shNEK6#3	shNEK6#3	shNEK6#3	shNEK6#3	shNEK6#3+ C-MYC	shNEK6#3+ C-MYC	shNEK6#3+ C-MYC	shNEK6#3+ C-MYC	shNEK6#3+ C-MYC	shNEK6#3+ C-MYC
#sample	1	2	3	4	5	6	1	2	3	4	5	6	1	2	3	4	5	6
5’-Deoxyadenosine	7414366	7311228	4902282	4575020	5456918	5439351	1493388	1736053	3180474	2299683	1693836	2306613	13792866	25175494	15570551	10037660	27102966	15779057
5’-Methylthioadenosine	177758530	110545960	209224248	212953646	273947124	213129680	25279346	17080345	38139672	73495938	56090218	112168490	65629629	89229478	61567968	38829559	47568354	46834215
7-methylguanosine	808679	711828	814870	1024238	1558725	1192454	451852	717655	731852	1724775	796301	2564034	1625729	1674517	990360	997658	909919	1311524
Adenosine	653760902	658812822	557964154	362538886	379548274	629357607	127251743	173727814	151594172	169044211	172120807	234050448	254042424	305460773	253615413	207224477	276225456	265142108
AMP	24211	21296	36589	24938	29036	28943	22651	28519	16475	12428	10246	15893	48427	57882	64442	53150	36411	34951
Deoxyguanosine	90292	286152	119788	108879	131787	758445	97583	104862	165665	169673	117242	174042	1046806	1071930	876410	809041	1287818	1054438
GMP	25123	39688	24210	42966	29856	55345	18569	25220	20389	17479	27869	26409	251963	218469	197341	178774	280003	300025
Guanosine	9511717	8481284	7601370	9651128	8418055	7791511	5696451	4922803	6171517	5925752	5440105	6222326	6516342	7248983	9587535	13777922	6023417	12483211
Inosine	406498217	496777466	406982367	412006943	340635028	407478954	76296754	135002514	187126442	226044466	102941949	266154845	305444641	293709550	364483941	260571215	359306552	355936934
N2,N2-Dimethylguanosine	62391	1400885	885362	954303	859383	1193523	723113	865850	647388	674516	1041793	1277638	7780530	6923096	3946914	4786165	4941289	6079589
N6-methyladenosine	862097	777004	653172	1014369	1364242	1292776	1320337	1292102	2317311	1754864	1630339	1691372	3432440	3113552	2364079	3230810	2804189	3109577
NAD	2347727	2657193	1468251	1668427	2598953	1485339	1013298	2002952	2505162	1916834	1570257	1577676	1697113	1866778	1580895	1271725	1200115	1281603
IMP	321282	254774	310303	285936	379000	353514	209871	280865	212372	246460	283212	212416	323331	345900	503306	315569	313498	310678
