# Supplementary material for: NEK6 dampens FOXO3 nuclear translocation to stabilize C-MYC and promotes subsequent de novo purine synthesis to support ovarian cancer chemoresistance
Source: Cell Death Dis. 2024 Sep 10;15(9):661. doi: 10.1038/s41419-024-07045-2 (PMC11387829; doi:10.1038/s41419-024-07045-2)
Supplement: Supplementary file 10 — Original western blots [file 41419_2024_7045_MOESM10_ESM.pdf]

Fig. 3B

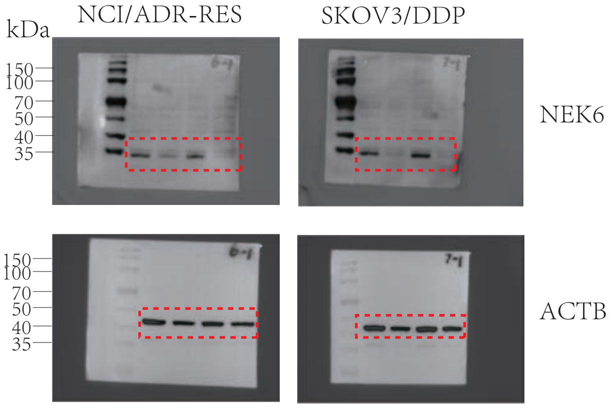

Fig. 4A

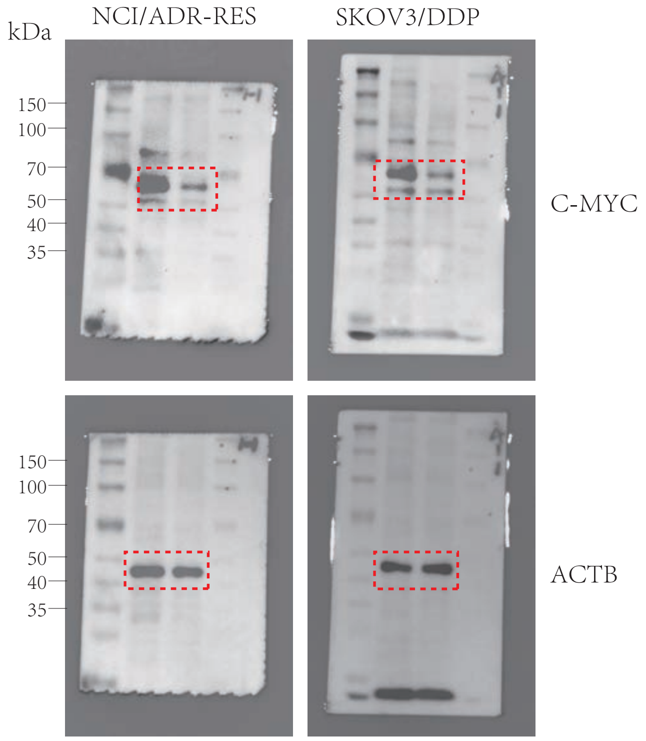

Fig. 4H

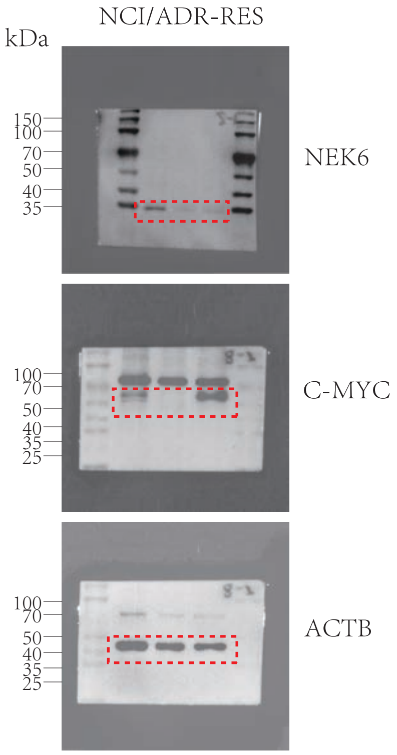

Fig. 5A

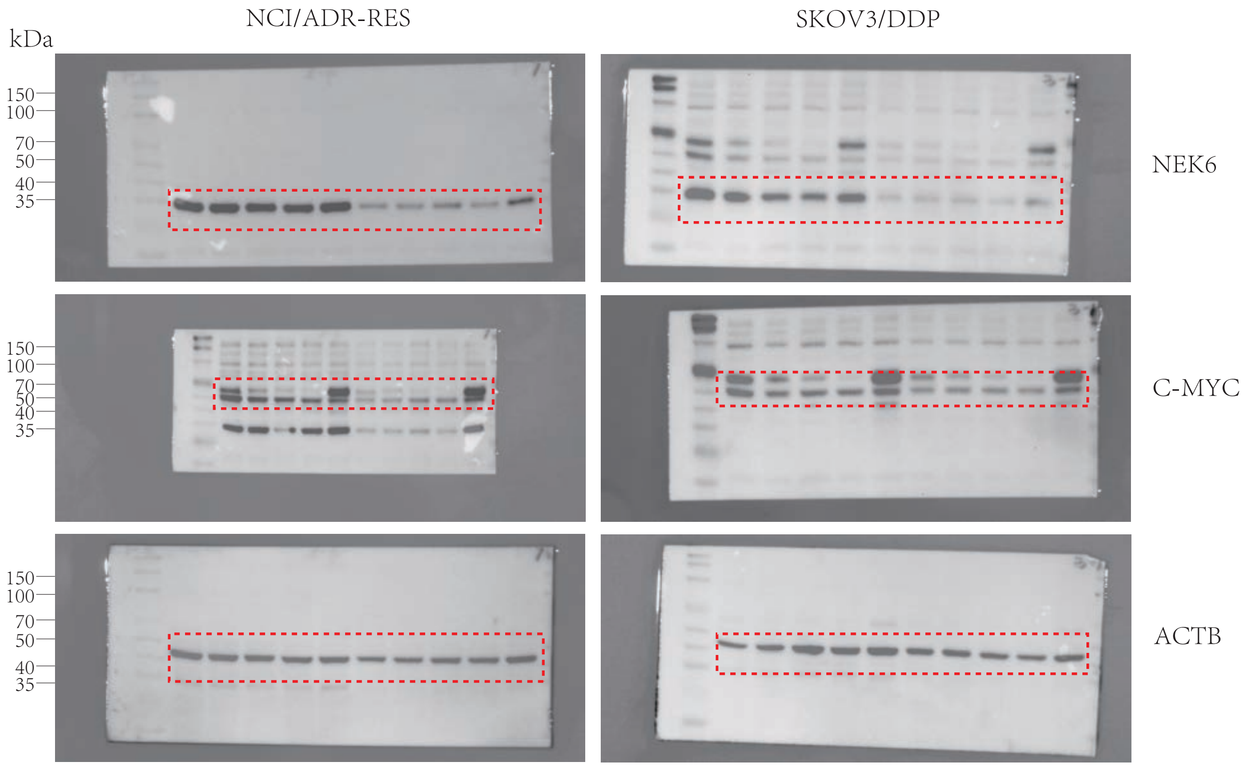

Fig. 5B

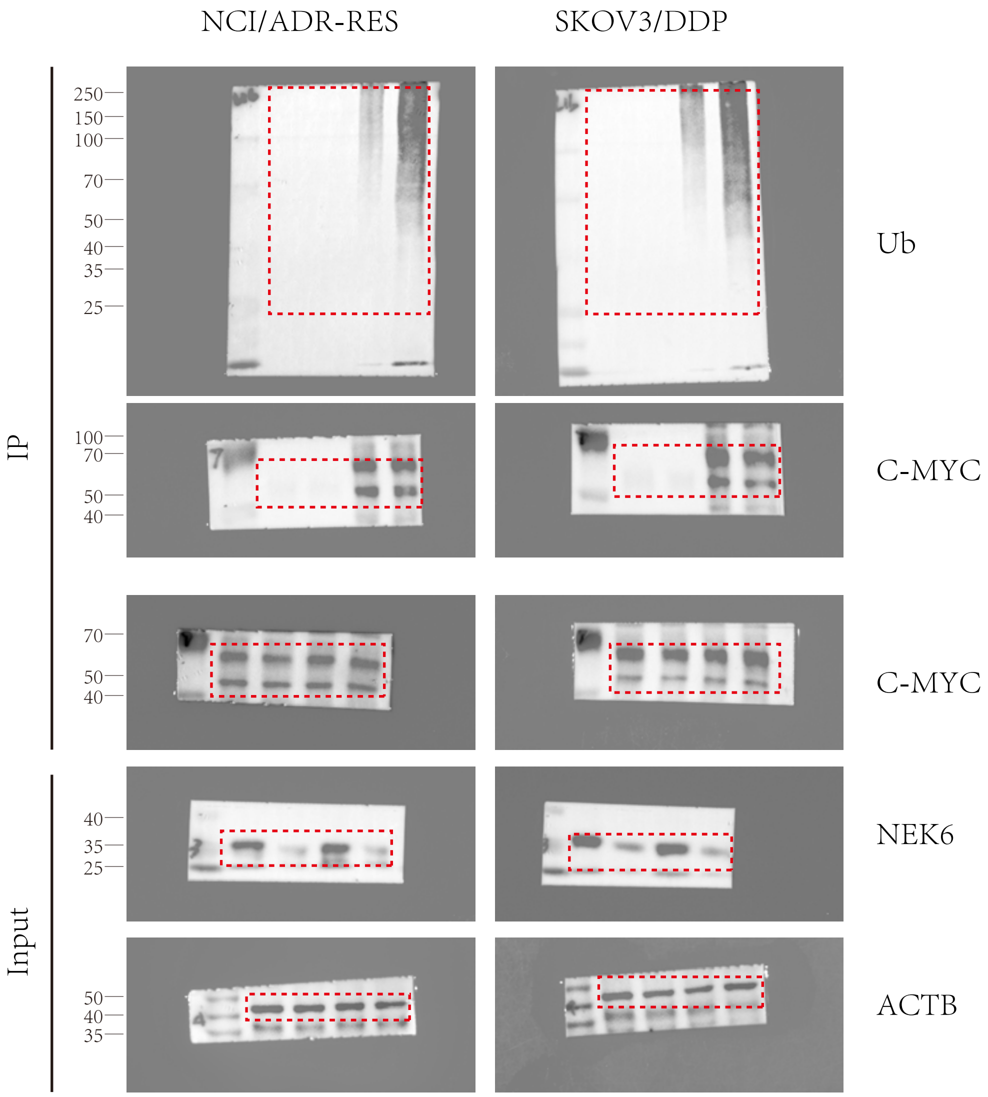

Fig. 5C

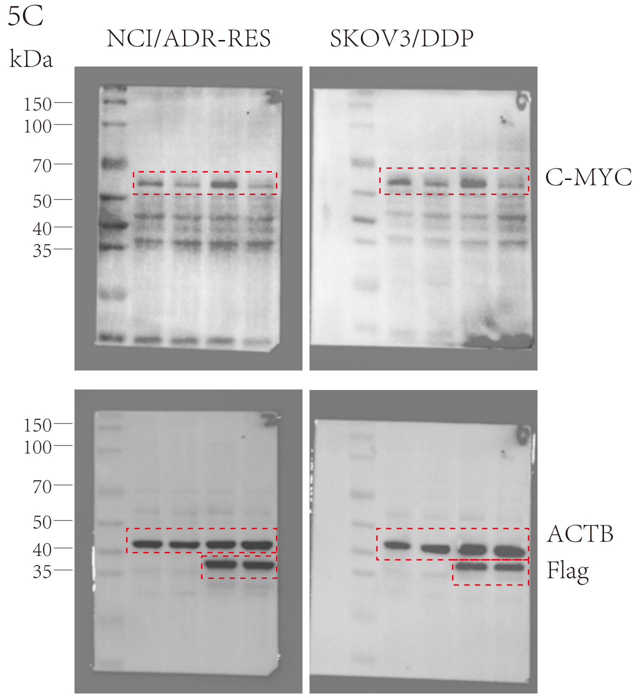

Fig. 6C

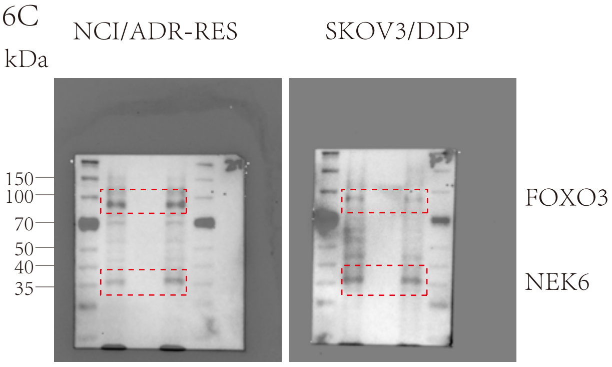

Fig. 6D

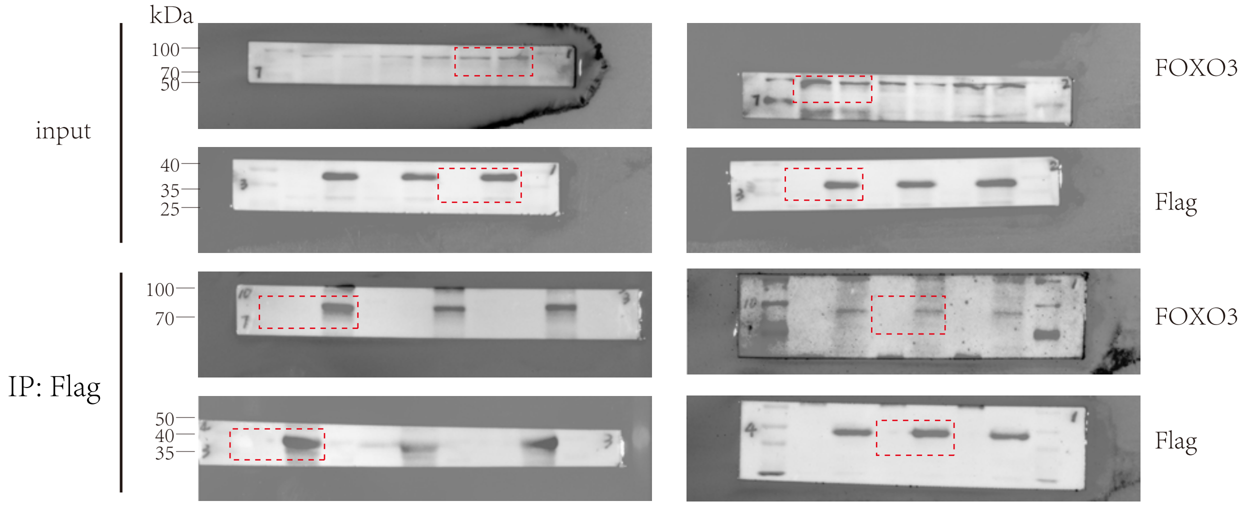

Fig. 6E

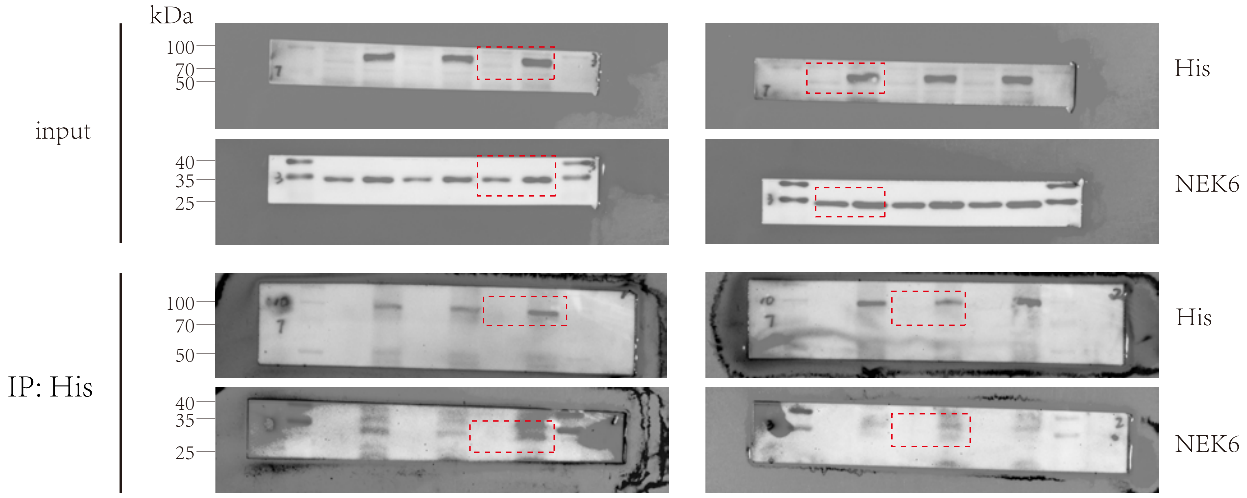

Fig. 6H

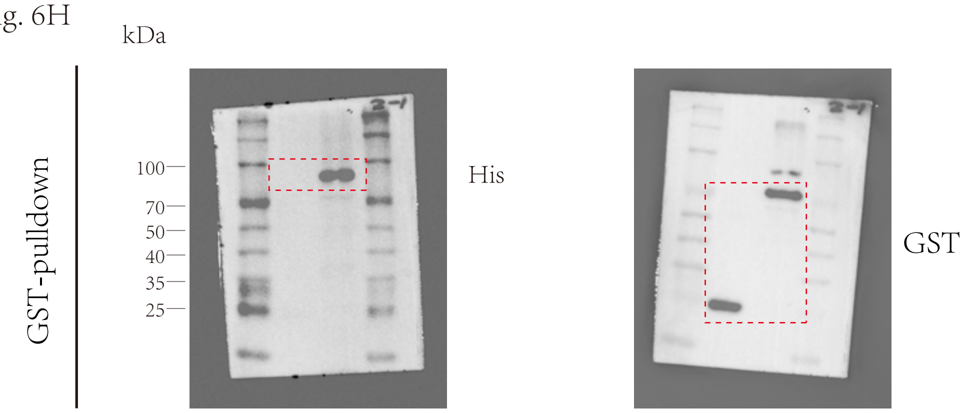

Fig. 6K

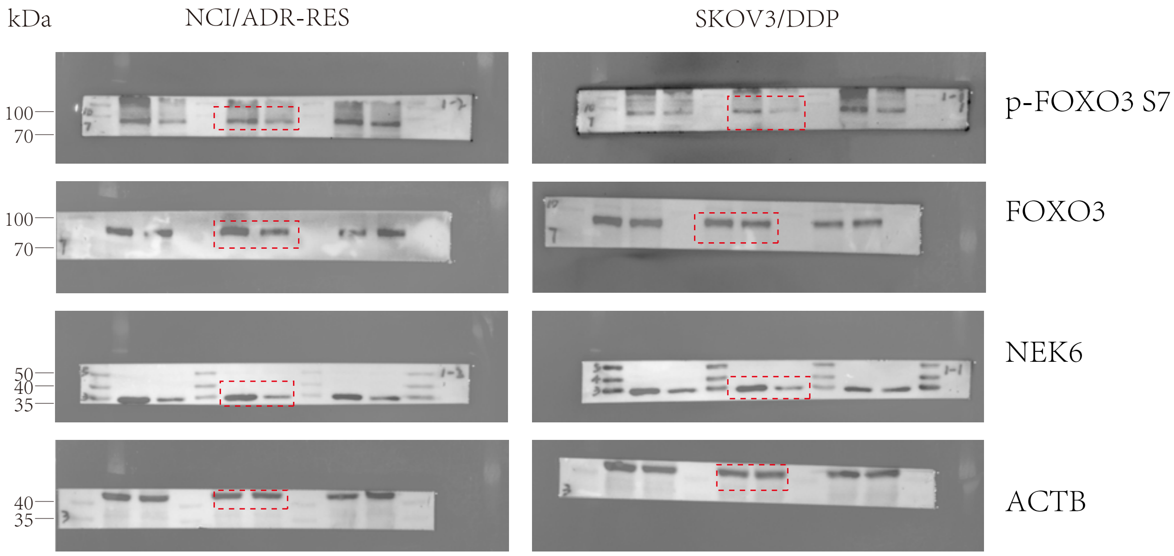

Fig. 6L

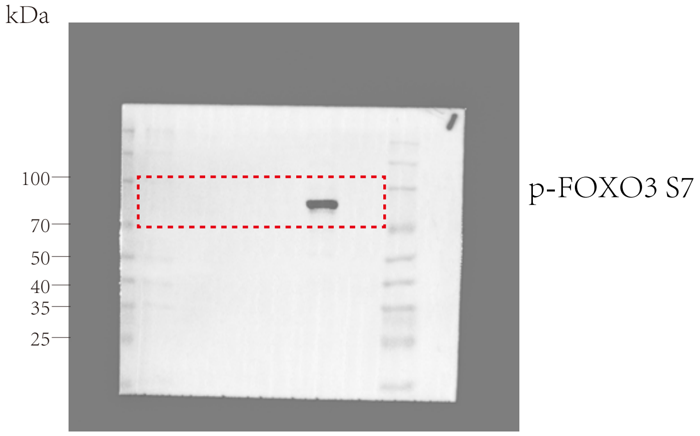
kDa
100
70
50
40
35
25
p-FOXO3 S7

Fig. 6M

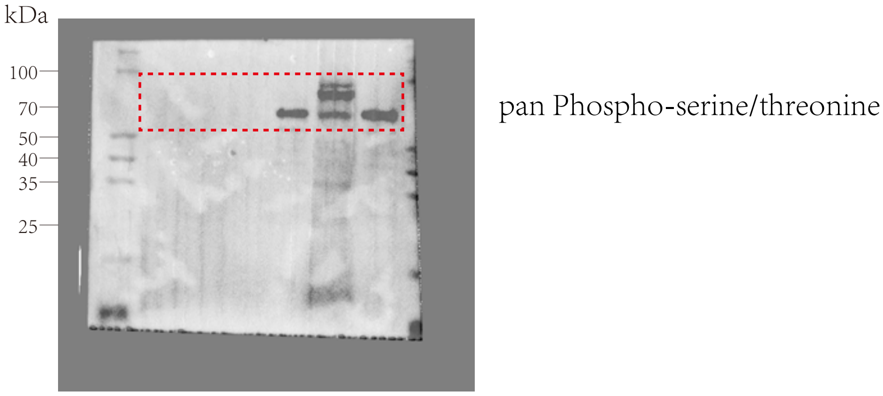
kDa
100
70
50
40
35
25
pan Phospho-serine/threonine

Fig. 7A

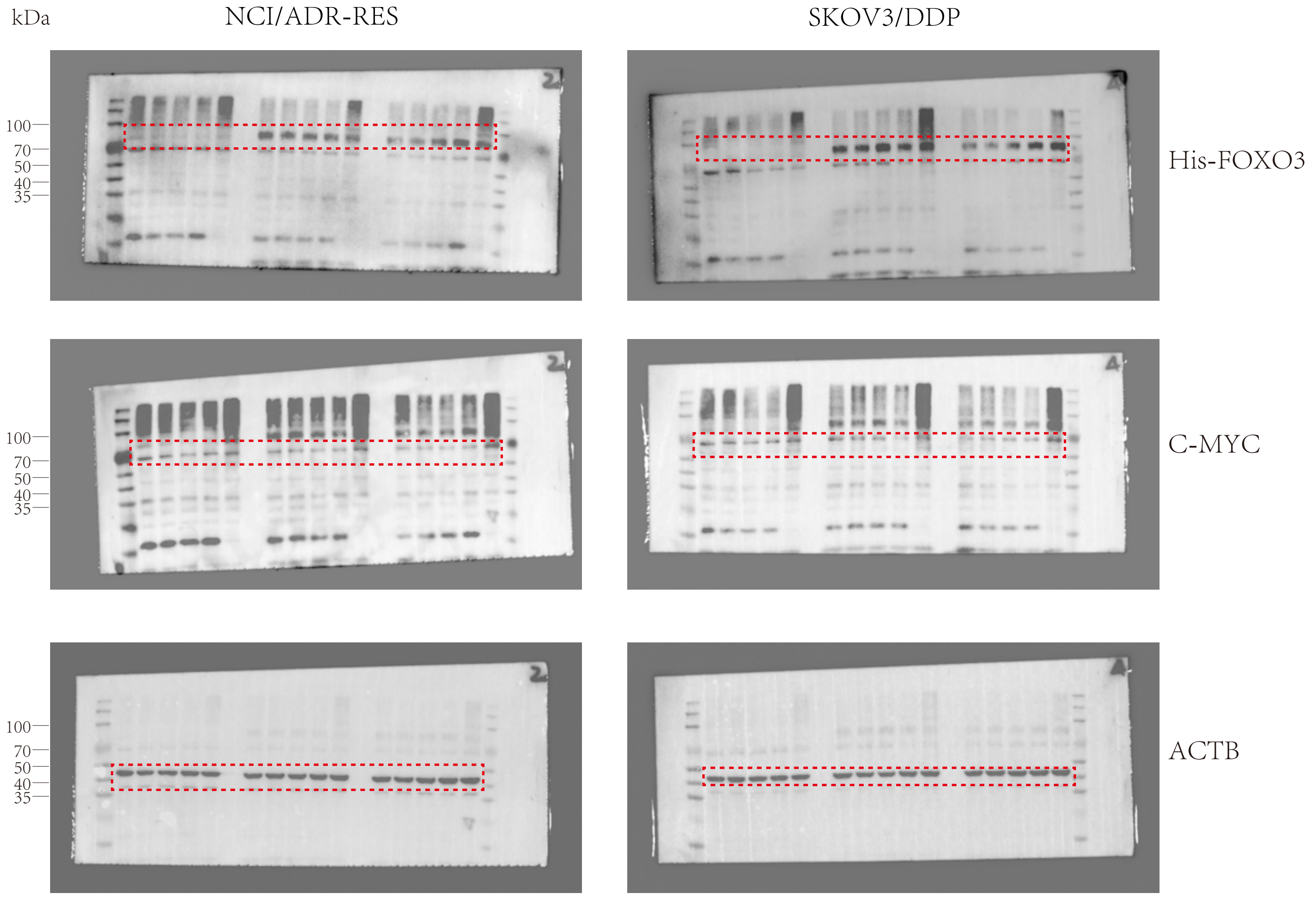
kDa
NCI/ADR-RES
SKOV3/DDP
100
70
50
40
35
His-FOXO3
C-MYC
ACTB

Fig. 7B

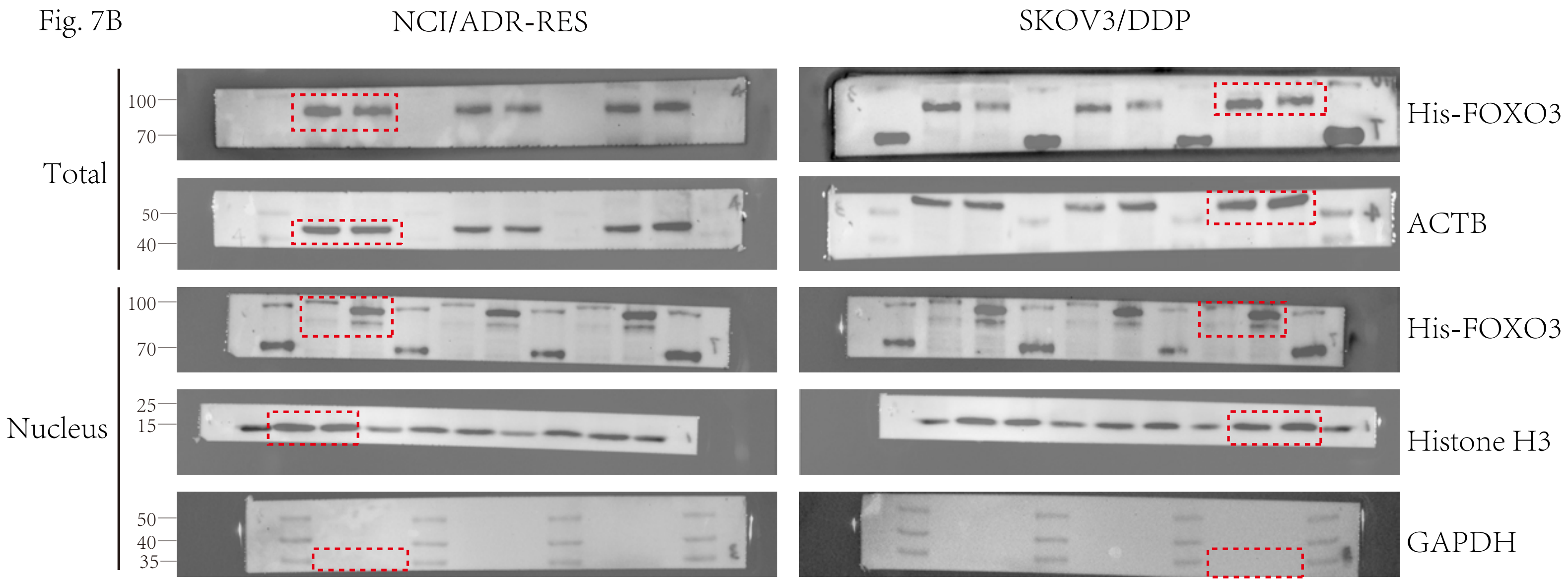
NCI/ADR-RES
SKOV3/DDP
Total
Nucleus
100
70
50
40
25
15
35
His-FOXO3
ACTB
His-FOXO3
Histone H3
GAPDH

Fig. 7H

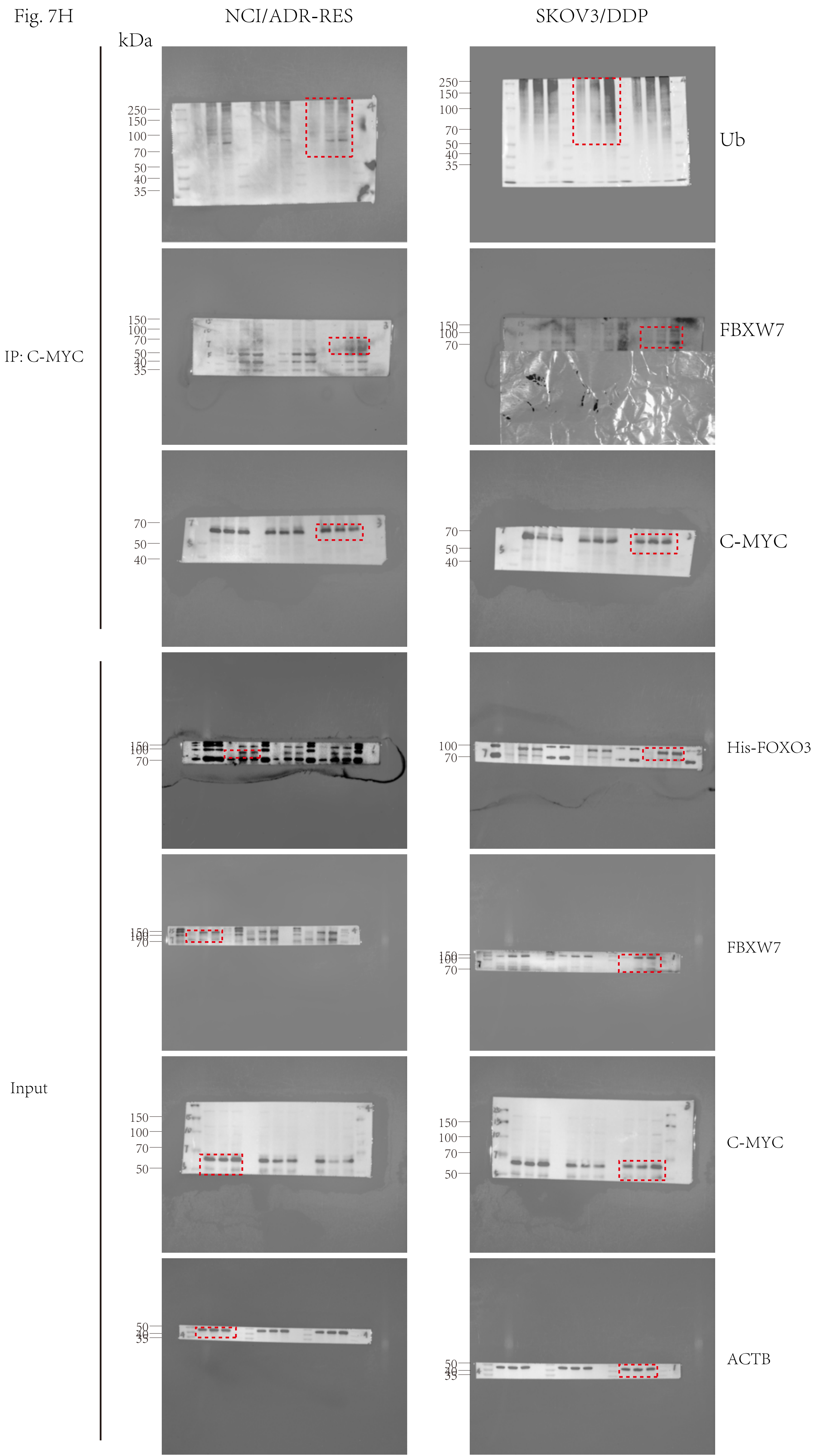
NCI/ADR-RES
SKOV3/DDP
kDa
IP: C-MYC
Input
Ub
FBXW7
C-MYC
His-FOXO3
FBXW7
C-MYC
ACTB
250
150
100
70
50
40
35

Fig. 8B

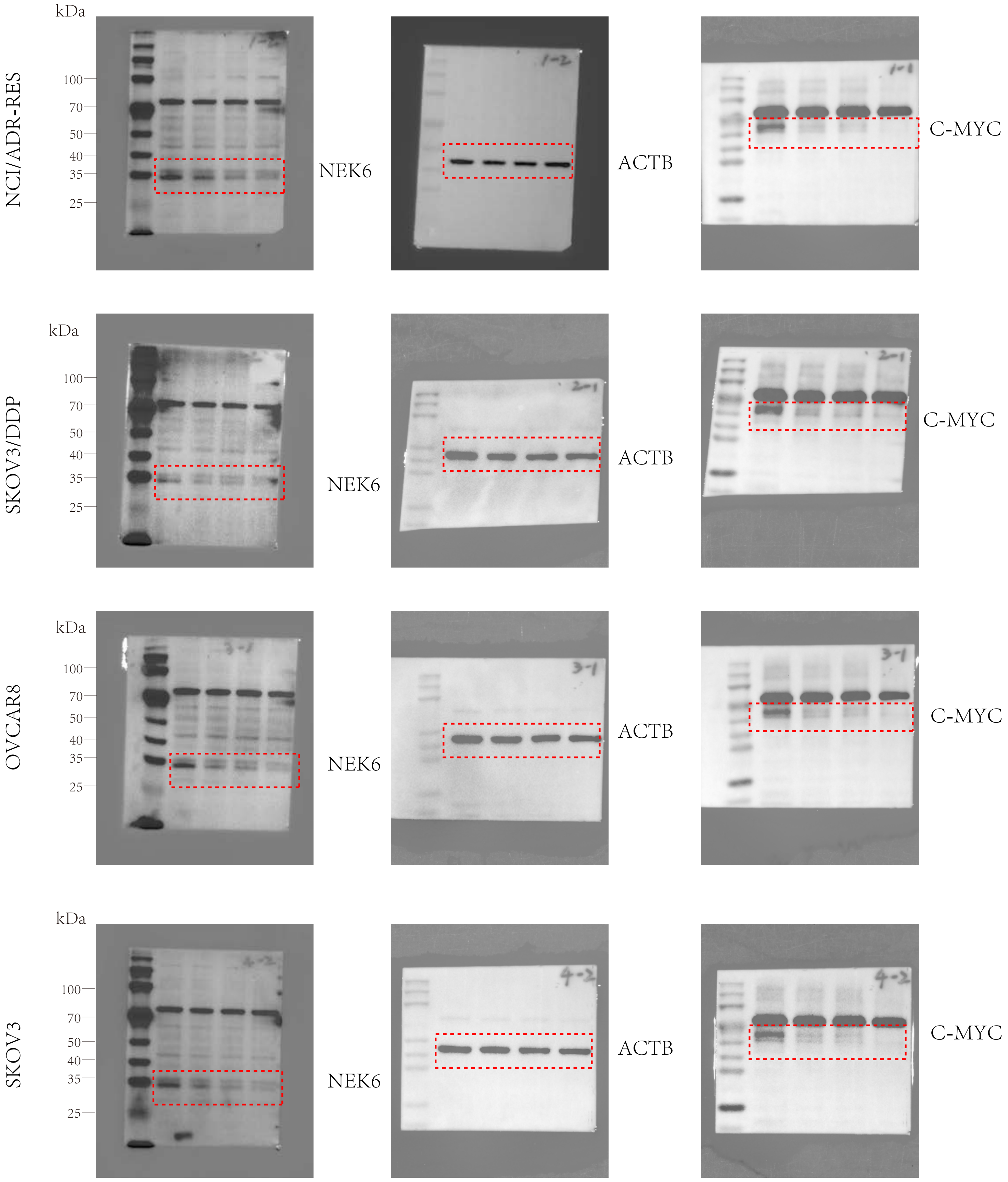
kDa
100
70
50
40
35
25
NCI/ADR-RES
NEK6
ACTB
C-MYC
SKOV3/DDP
OVCAR8
SKOV3

Fig. 8J

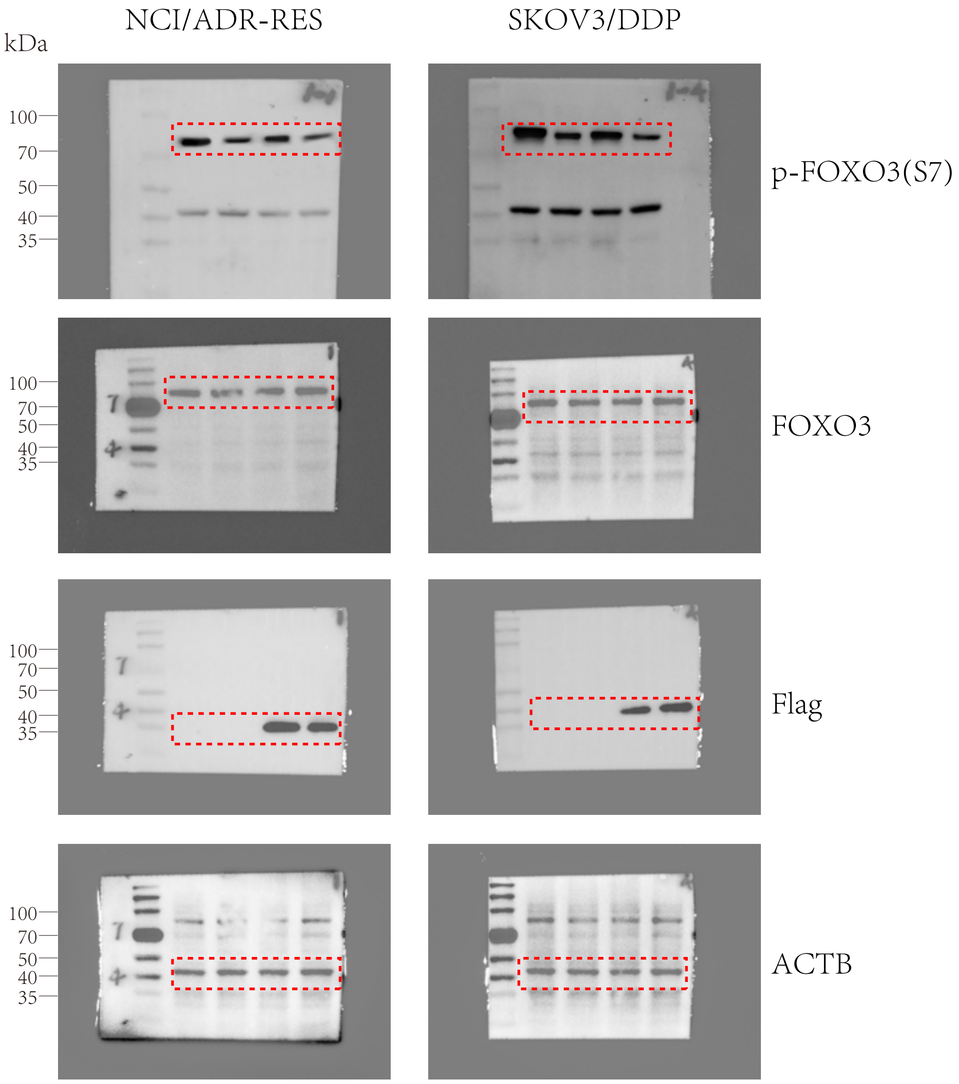

Fig. S2C

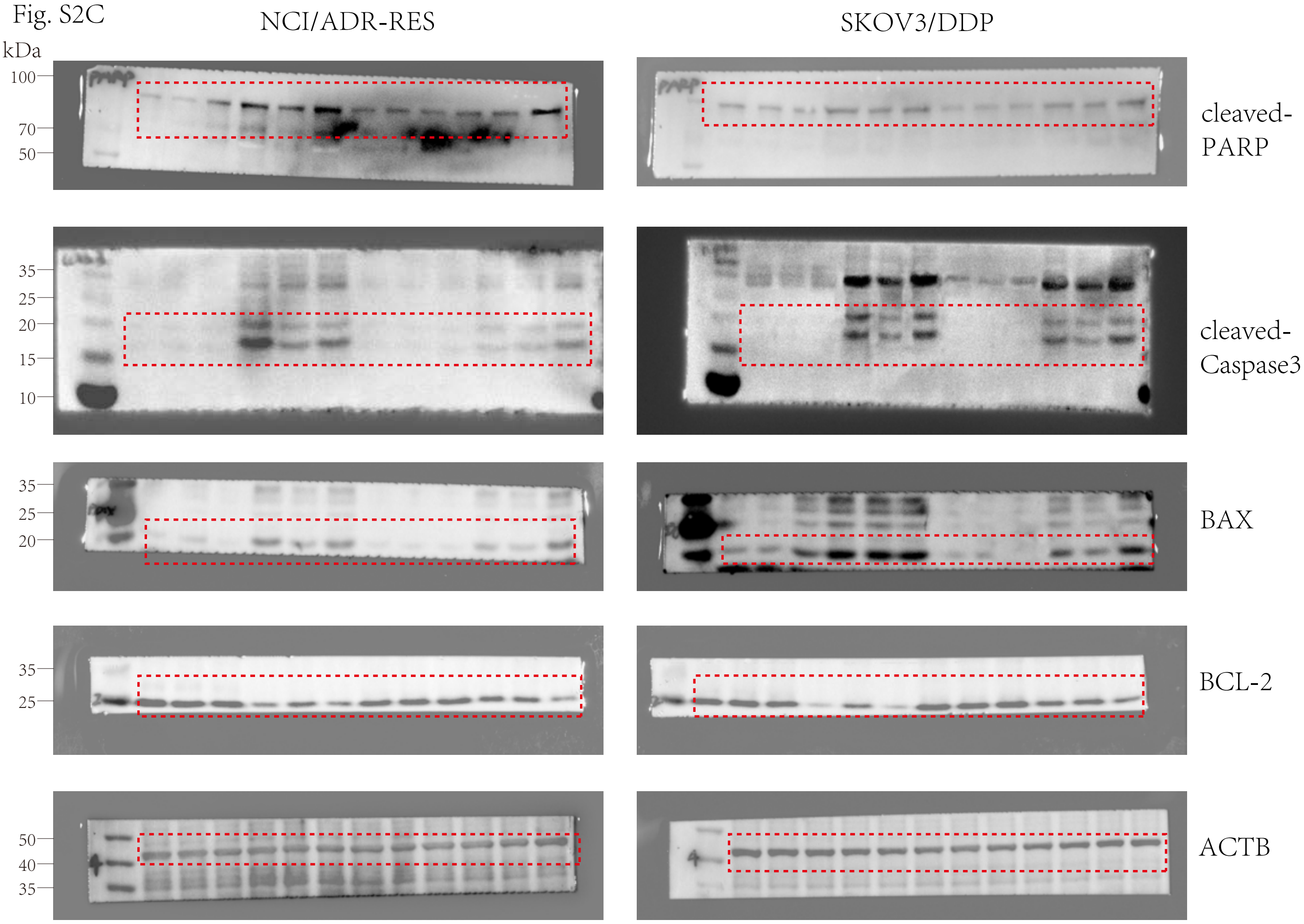

Fig.S3C

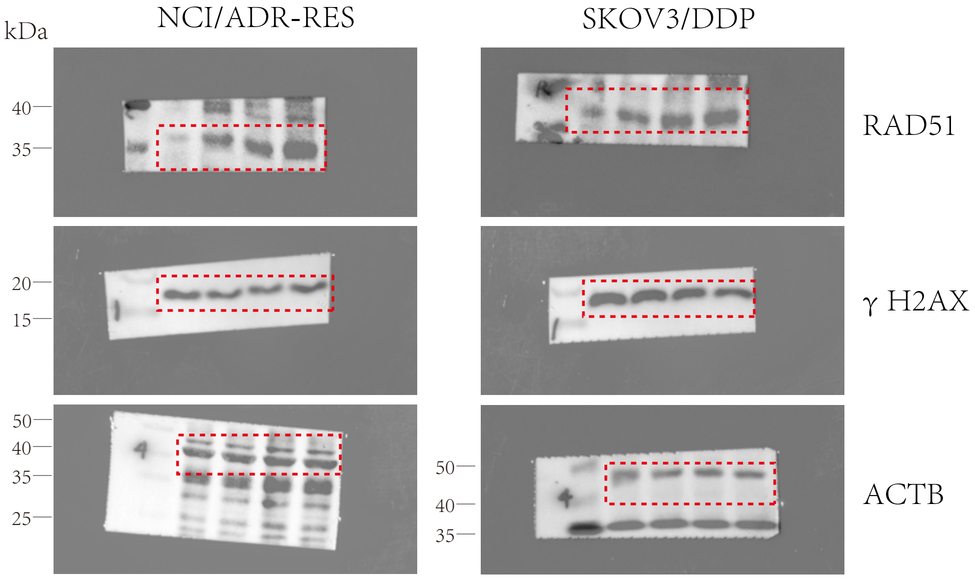

Fig. S6B

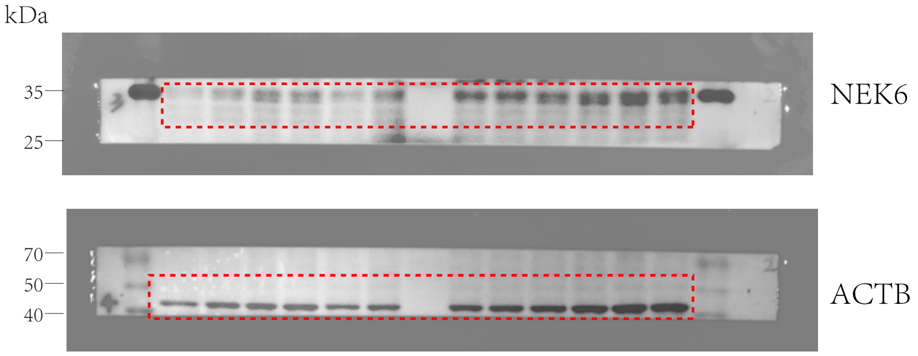

Fig. S6C

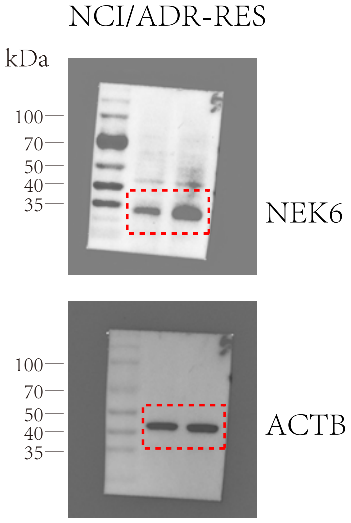

Fig. S6D

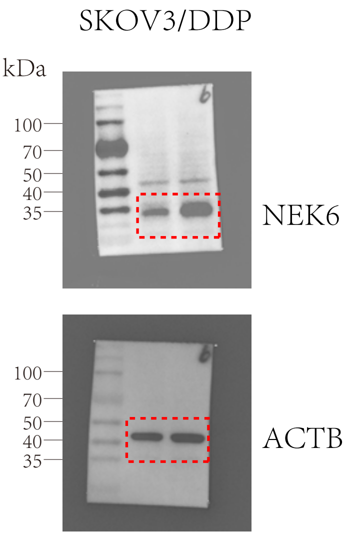

Fig. S7D

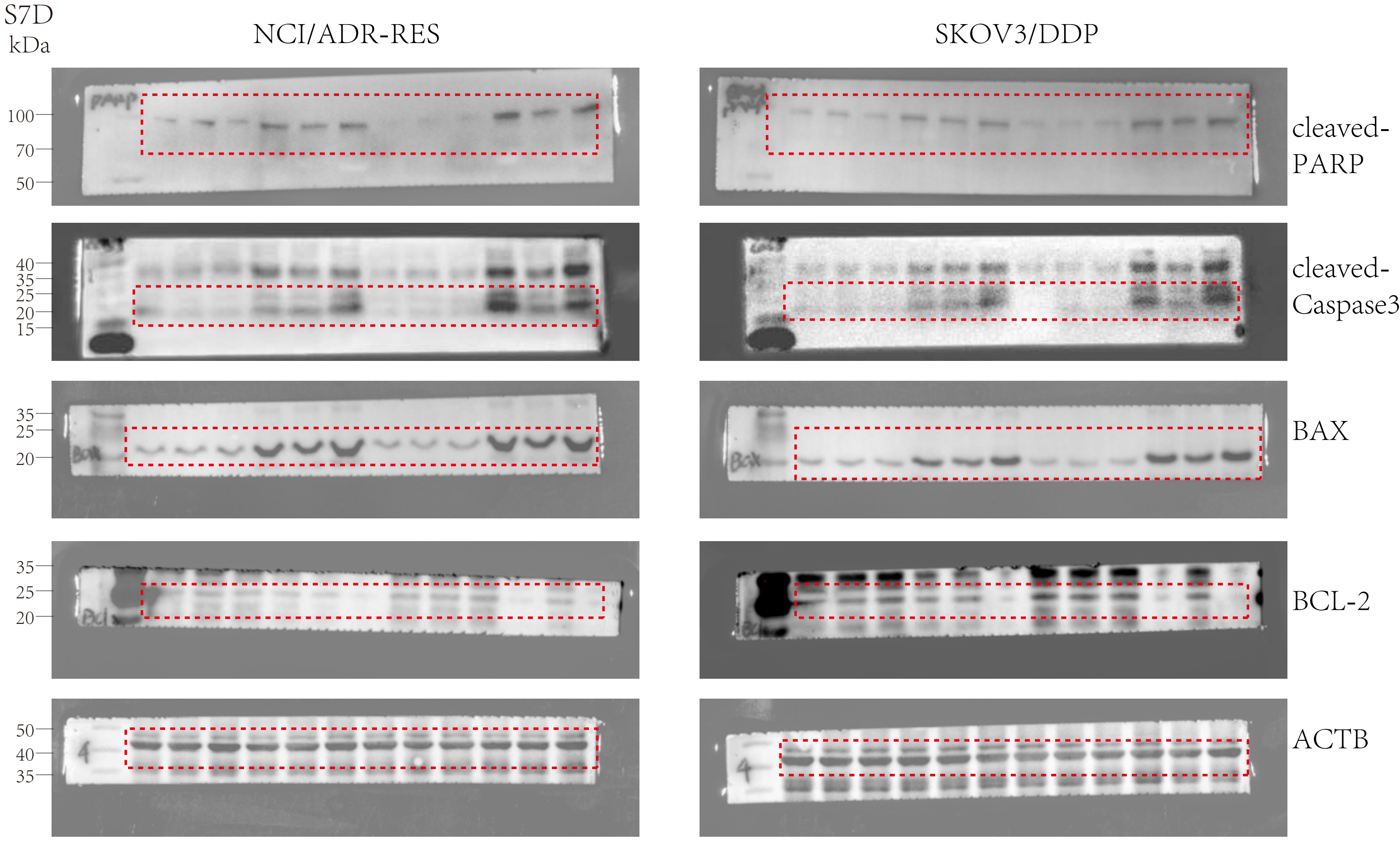

Fig. S7F

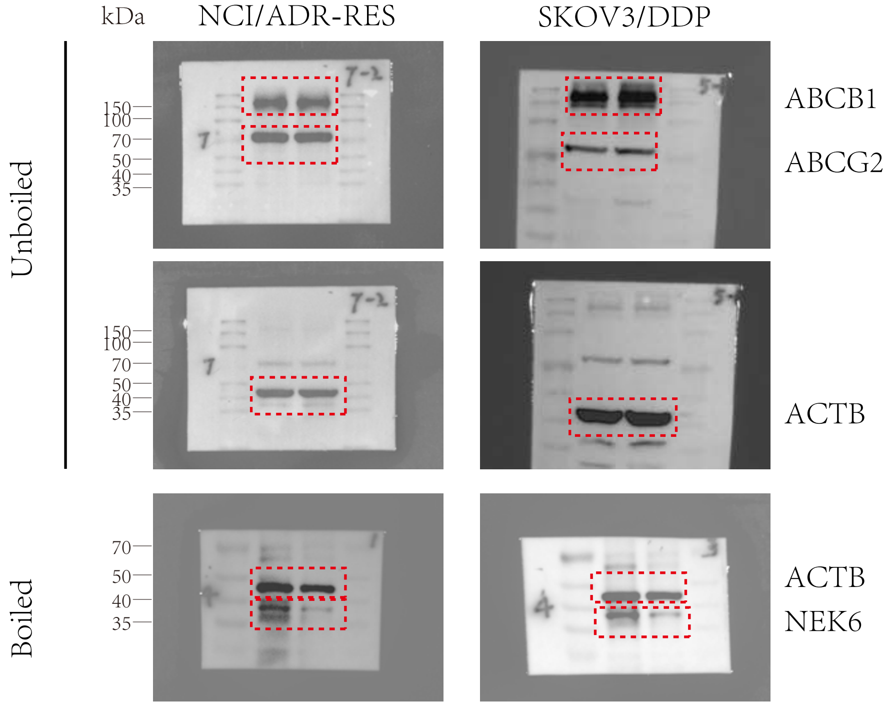

Fig. S8A

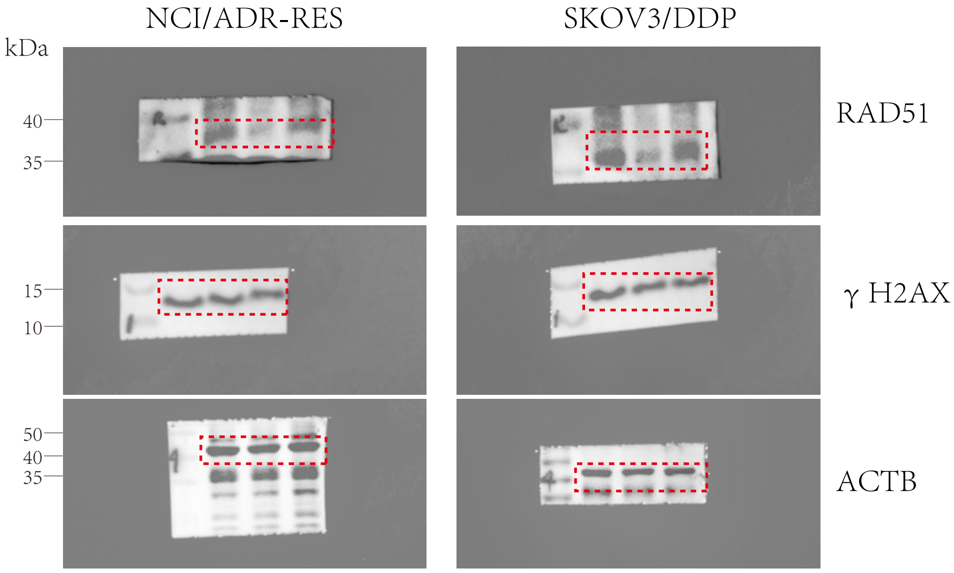

Fig. S8B

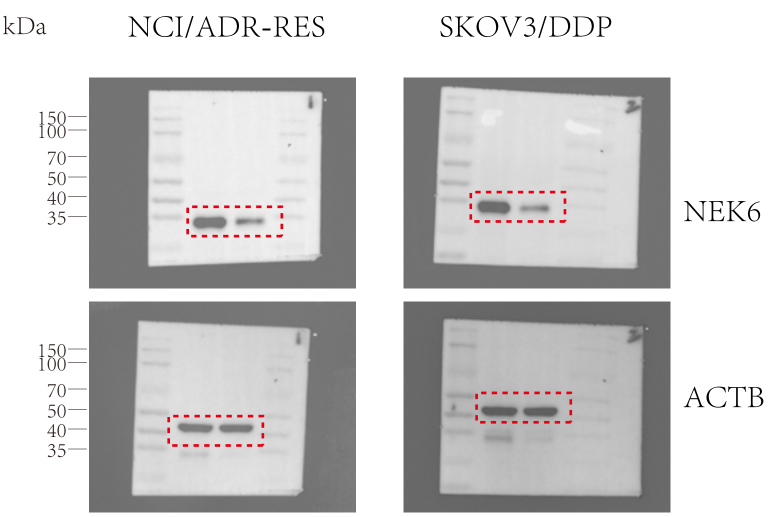

Fig. S9B

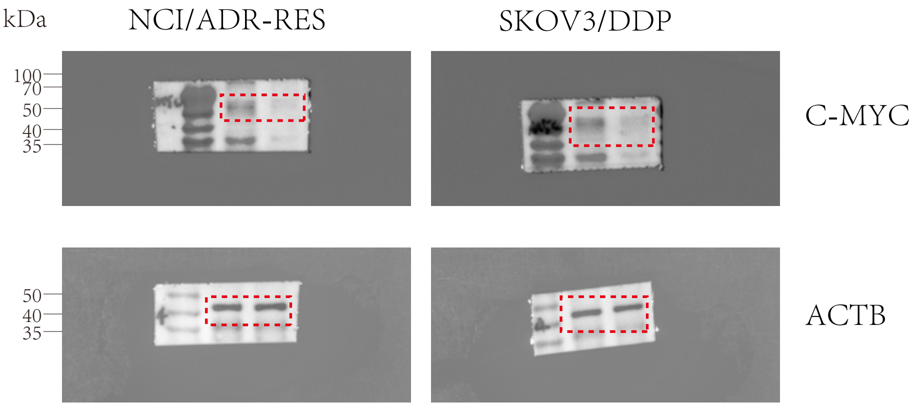

Fig. S9E

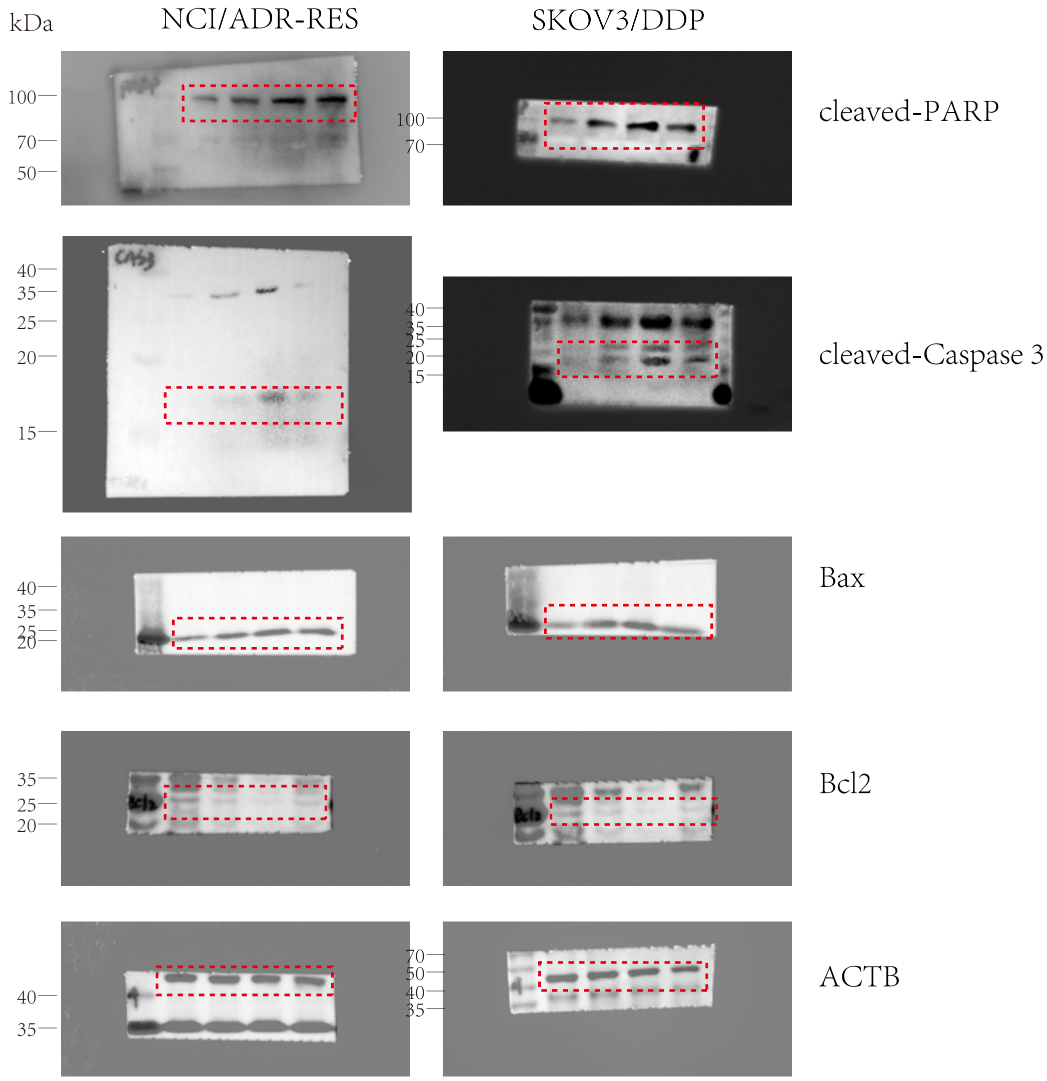

Fig. S9F

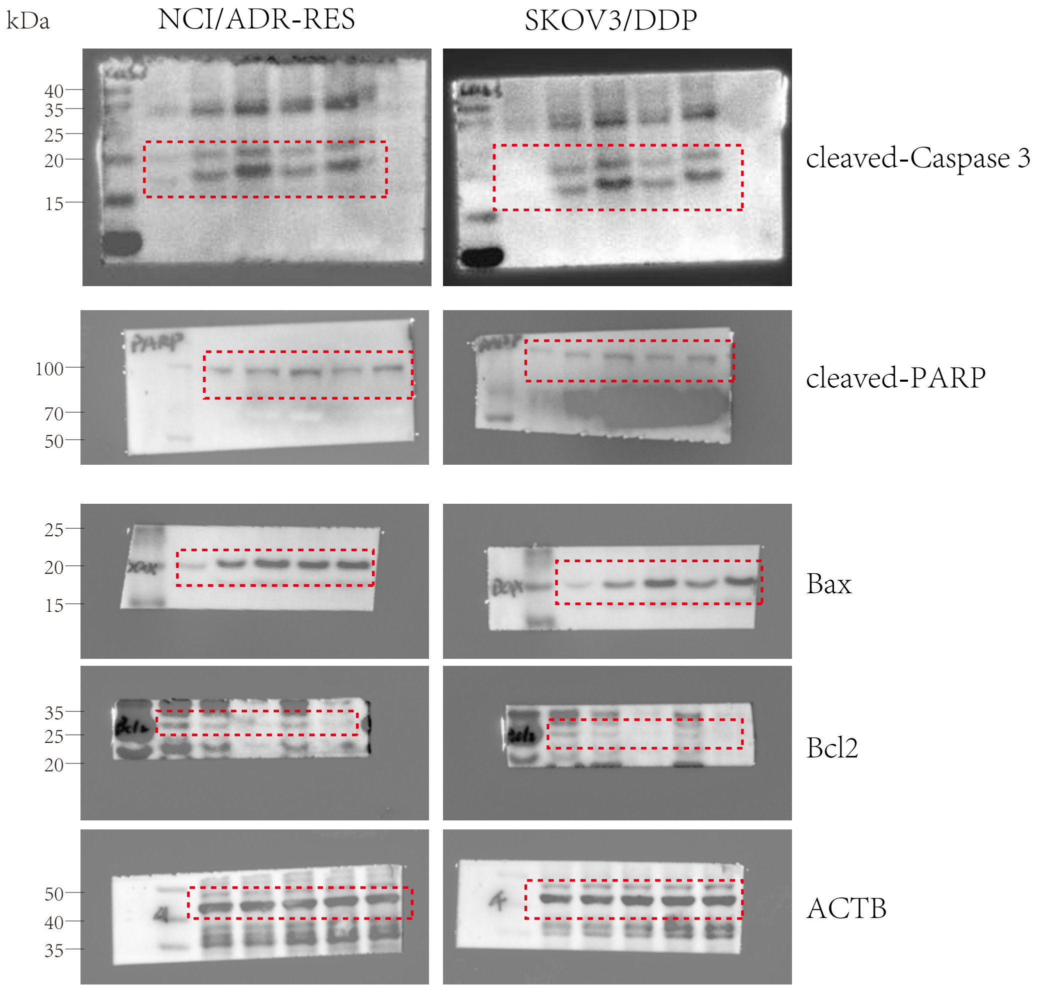
kDa
NCI/ADR-RES
SKOV3/DDP
40
35
25
20
15
cleaved-Caspase 3
100
70
50
cleaved-PARP
25
20
15
Bax
35
25
20
Bcl2
50
40
35
ACTB

Fig. S10C

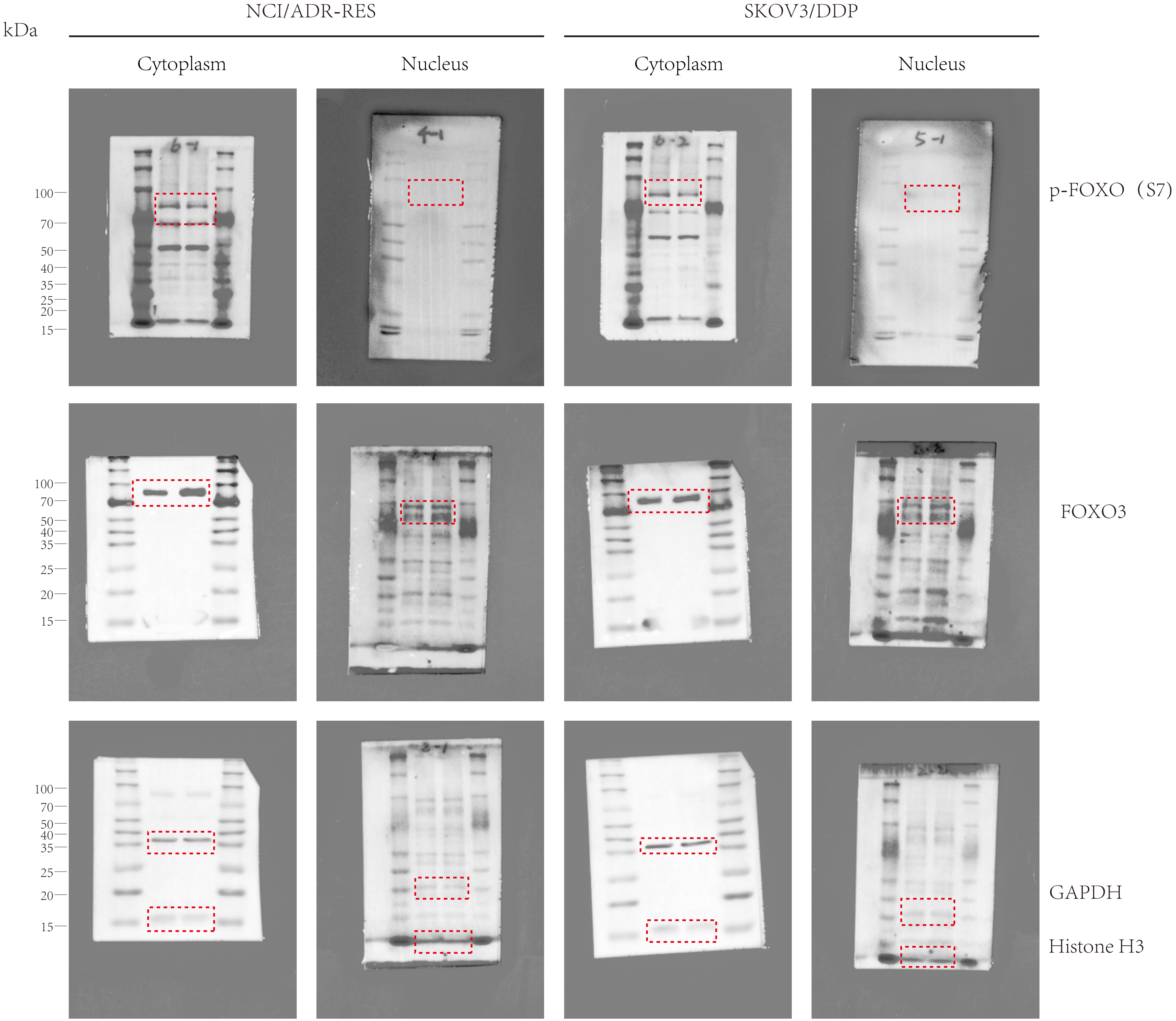
kDa
NCI/ADR-RES
SKOV3/DDP
Cytoplasm
Nucleus
Cytoplasm
Nucleus
100
70
50
40
35
25
20
15
p-FOXO（S7)
FOXO3
GAPDH
Histone H3

Fig. S10E

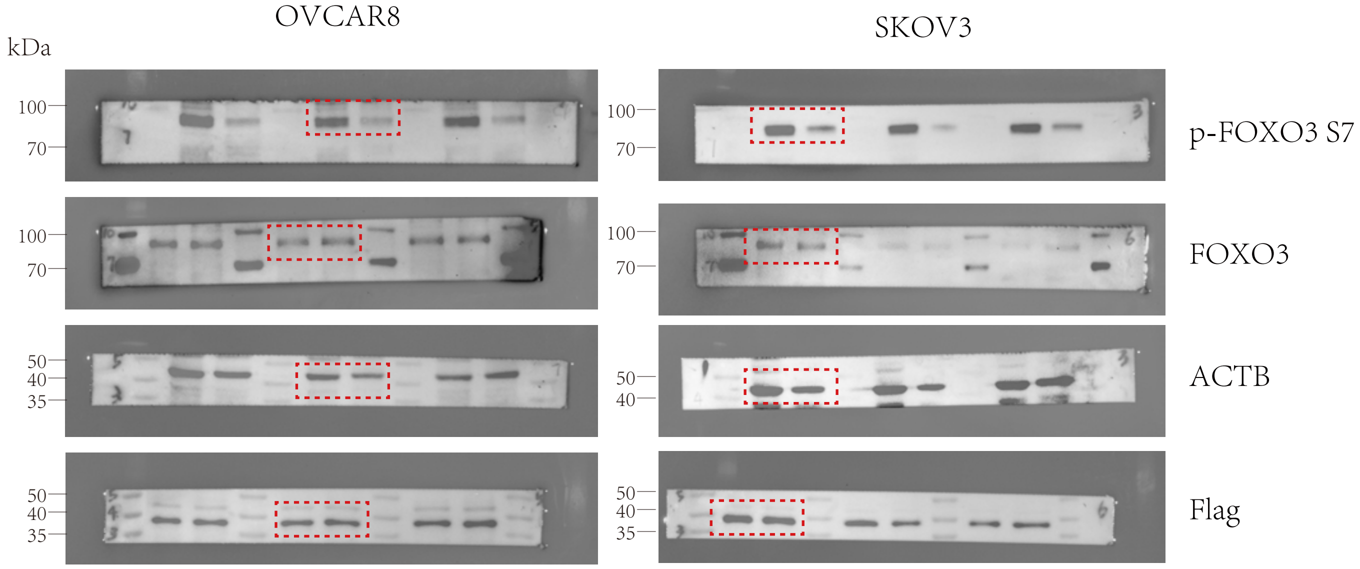

Fig. S10F

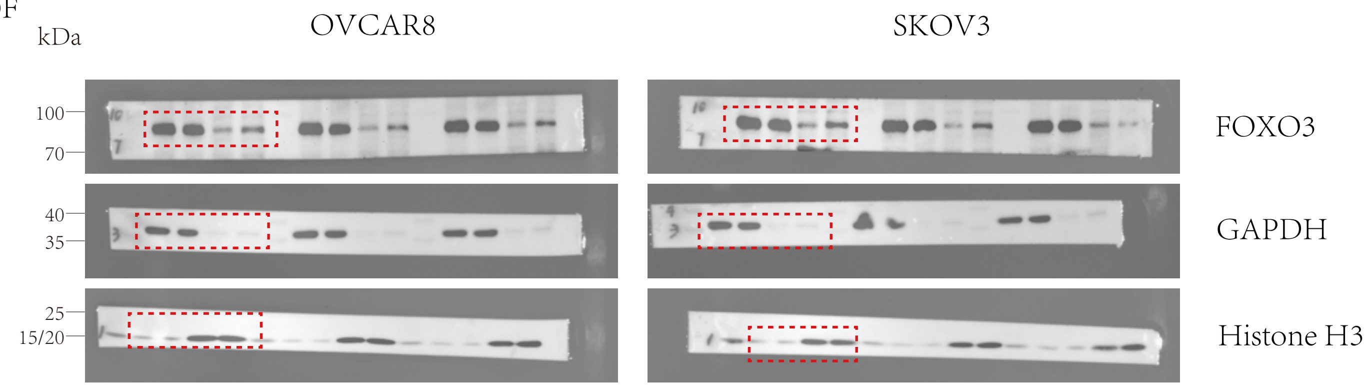

Fig. S12D

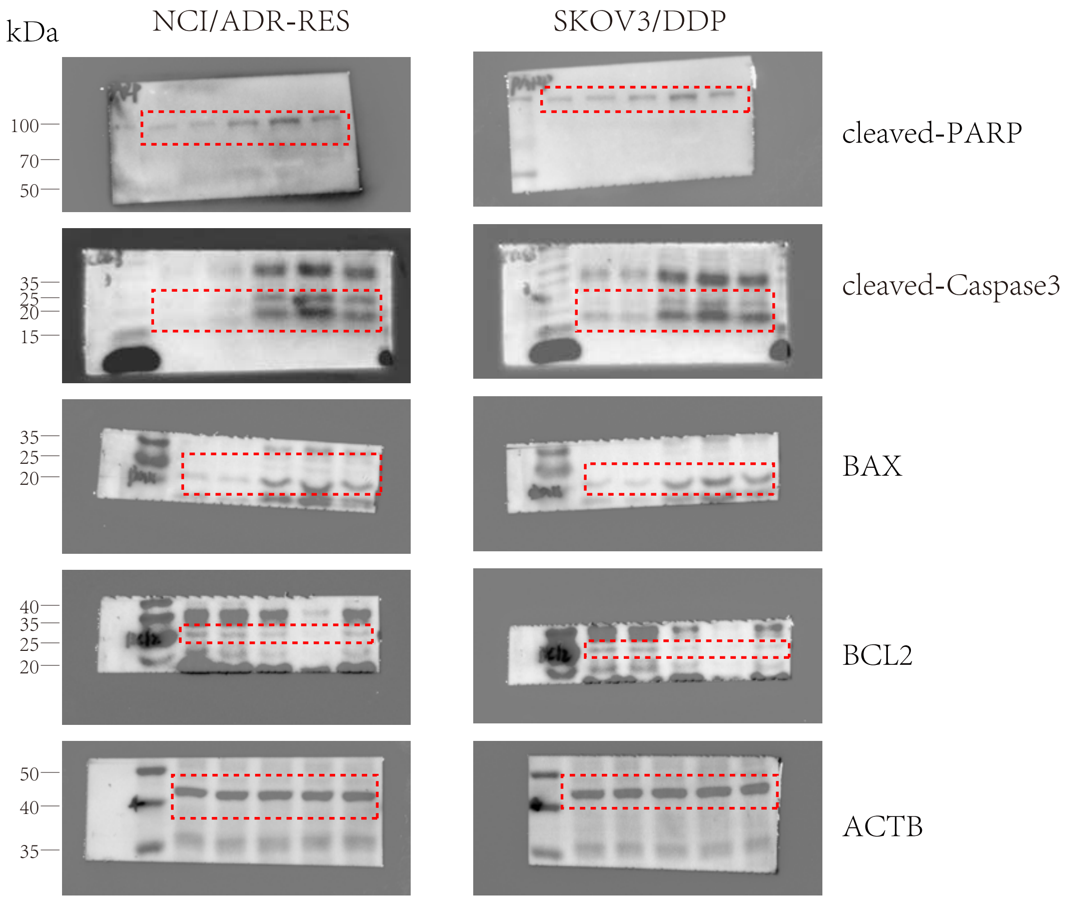

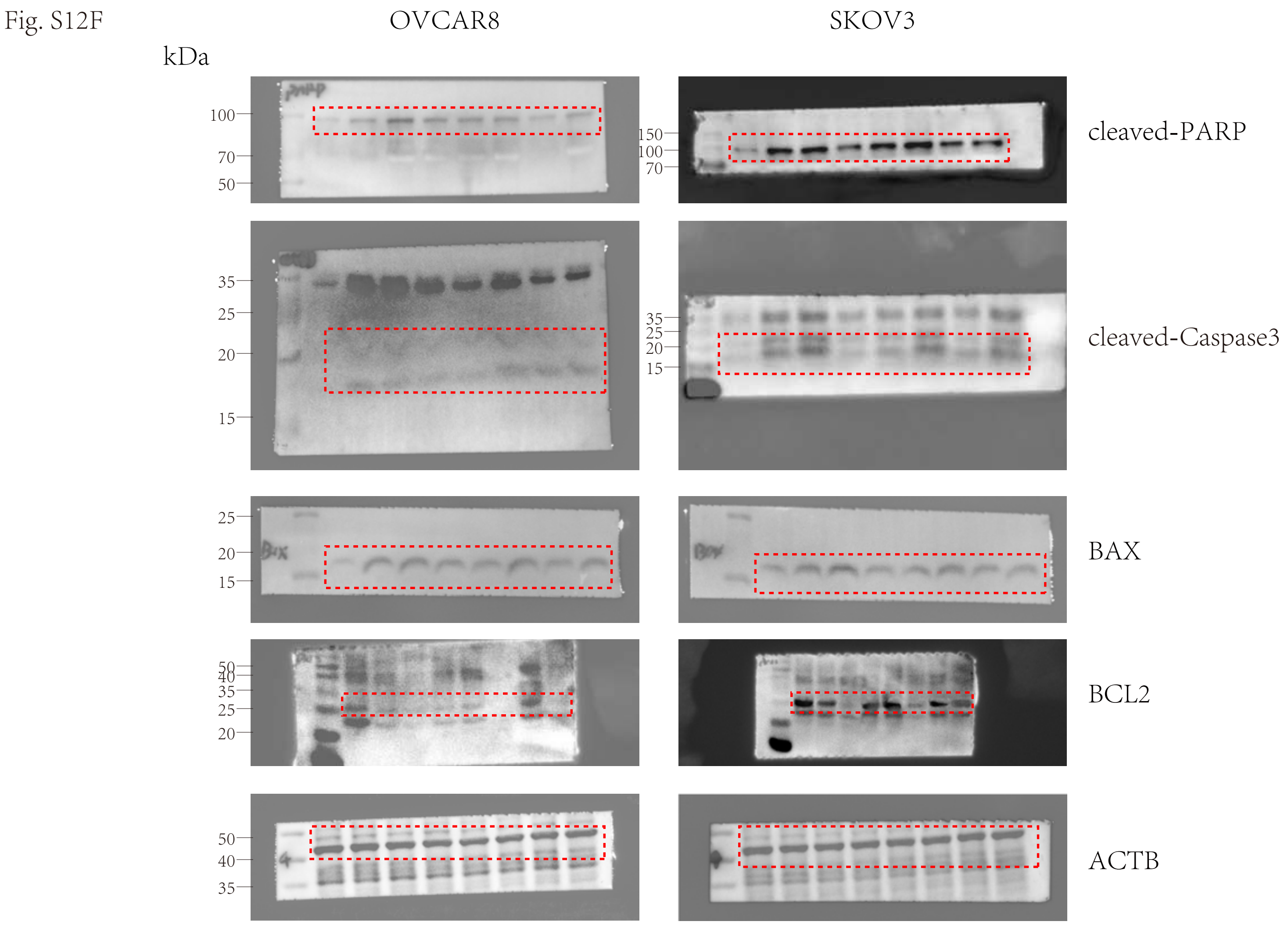

Fig. S12F
OVCAR8
SKOV3
kDa
100
70
50
150
100
70
cleaved-PARP
35
25
20
15
35
25
20
15
cleaved-Caspase3
25
20
15
BAX
50
40
35
25
20
BCL2
50
40
35
ACTB
